# Associations between the use of psychedelics and other recreational drugs with mental health and resilience during the COVID-19 pandemic

**DOI:** 10.3389/fpsyt.2023.1184681

**Published:** 2023-06-15

**Authors:** Maria Bălăeț, William Trender, Peter J. Hellyer, Adam Hampshire

**Affiliations:** ^1^Department of Brain Sciences, Imperial College London, London, United Kingdom; ^2^Engineering and Physical Sciences Research Council CDT Neurotechnology, Imperial College London, London, United Kingdom; ^3^Centre for Neuroimaging Sciences, Institute of Psychiatry, Psychology and Neuroscience, King’s College London, London, United Kingdom

**Keywords:** COVID-19, psychedelics, cannabis, mental health, resilience

## Abstract

The large-scale disruption to peoples’ daily lives during the COVID-19 pandemic provides a context for examining whether use of substances such as psychedelics in a naturalistic (outside of a controlled environment) setting, is associated with better mental wellbeing and resilience relative to those who use other drugs, or who do not use drugs at all. We interrogate data from the Great British Intelligence Test and identify that 7.8% out of *N* = 30,598 unique respondents used recreational drugs inclusive of psychedelics, cannabis, cocaine, and MDMA during the COVID-19 pandemic. Recruitment materials did not mention drug use would be surveyed, thereby enabling us to model the relationship with mood and resilience in people who had not specifically self-selected themselves for a ‘drug’ study. We report that people form clusters, characterized by different real-world patterns of drug use, and the majority of psychedelics users also use cannabis. However, a subset of cannabis users do not use psychedelics, enabling a subtractive comparison. Those who primarily used psychedelics and cannabis during the COVID-19 pandemic had worse mood self-assessment and resilience scores compared to those who never used drugs or primarily used cannabis. This pattern was also evident for other recreational drug use clusters, except for those who primarily used MDMA and cannabis, who had better mood but were of too low incidence to have confidence in this estimate. These findings cast light on the significant differences in mental wellbeing between users of different drugs and the non-user population during a global-crisis and call for future research to explore the pharmacological, contextual and cultural variables associated with these differences, their generalisability and causal links with greater precision.

## 1. Introduction

The large-scale disruption of peoples’ lives during pandemic provides a unique context for studying the population variables that underpin individual differences in mental health vulnerability and resilience. Sociodemographic characteristics, dimensions of personality, and lifestyle choices such as exercise, meditation and social media use have all received substantial attention as predictors of mental health and/or resilience and have been extensively modeled in large datasets (1–7). Notably, early predictions were made about a prospective increase in drug use in order to cope with pandemic-induced stress (8–10). However, naturalistic drug use (drug use outside of controlled settings, regardless of underlying reasoning) has been largely overlooked in the majority of large-scale studies surveying the general population. Instead, the effects of drug use have mostly been quantified in studies specifically looking at vulnerable groups, such as people with existing substance use problems (11), or adolescents (12). Consequently, it remains unclear whether members of the broader general population who used drugs during the COVID-19 pandemic were more or less resilient during the global crisis than those who have not used drugs in their lifetime, and whether this varied depending on which drugs they used.

Indeed, investigating the relationship between naturalistic drug use, mental health and resilience in the general population is well-justified; with a recent survey showing that 1 in 11 individuals aged 16–59 in the UK declared drug use within the past year (13), it is likely to be a significant modulator of psychological wellbeing. Cannabis, cocaine, and MDMA/ecstasy are the most common choices in the UK (13), and a recent review of epidemiological data suggests increased global use of psychedelics (14). However, as the current literature provides conflicting evidence, the direction of this modulation is difficult to predict. On the one hand, concerns have been raised about a negative association between drug use and mental health outcomes independently of the COVID-19 pandemic (15–17). On the other hand, a raft of other studies have argued that certain drugs could be used to counter mental health problems (18–20). A pre-pandemic review (21) discusses cannabis, the most widely used recreational drug across the world, as a good example where two competing views dominate the narrative: either it is considered a contributor to poor mental health, or a therapeutic agent. 3, 4-Methylenedioxymethamphetamine (MDMA) is another prime example, where despite past studies illustrating an association between its use and the incidence of mental health problems (22), the recent narrative has been shifted in the light of clinical trials recommending its use for treating post-traumatic stress disorder (20).

In particular, psychedelics have recently received a tremendous increase in positive attention relative to other drugs, with therapeutic effects noted in clinical studies conducted with patients suffering from depression (18), anxiety (19), alcoholism (23), tobacco addiction (24) and obsessive–compulsive disorder (25). Furthermore, these positive effects have also been found to extend to instances where individuals chose to use psychedelics in naturalistic settings (26–29). They have been studied not only at full doses, but also reported by users at microdoses, which are sub-threshold doses that allegedly do not entail a psychoactive manifestation but have been argued to carry benefits pertaining to wellbeing and cognitive performance (30). However, some recent prospective studies have largely attributed these perceived benefits to a placebo effect (31, 32). Despite this recent interest, the relationship between naturalistic drug use and mental health in the general population remains unclear.

A major contributor to the complexity of the problem is that a significant proportion of individuals who consume drugs are polydrug users – they tend to consume more than one drug (33). Therefore, modeling the effects of specific drugs in isolation does not reflect naturalistic behavior. Nor does analysis of drug use under controlled conditions necessarily reflect their effects in different, naturalistic settings. Moreover, there has been little research into whether the use of drugs engenders greater mental health resilience or vulnerability when dealing with real-life stressors. Understanding this complexity requires sampling the general population to capture data from individuals who use drugs in naturalistic settings on a large enough scale to analyse psychological wellbeing changes in response to environmental stressors whilst accounting for different real-world patterns of use.

The aim of the present study was to test the hypothesis that naturalistic use of psychedelics during the COVID-19 pandemic would be associated with better mental health and resilience than for users of other drugs, or non-drug users. For this purpose we analysed data from the Great British Intelligence Test, which surveyed tens of thousands of individuals longitudinally at 6-monthly timepoints between 2020 and 2023. We did not specifically advertise that the study would contain drug-use related questions, thus mitigating recruitment bias caused by self-selection characteristic to other studies of this kind. In particular, we analyse data collected at two timepoints: during December 2020 when national-level restrictions were enforced, and during June 2021 when restrictions were lifted for the first time in the UK. At both of these timepoints participants were presented with questionnaires assessing their personality, compulsivity, lifestyle, mental health and resilience. Without prior advertisement of the study content, participants were surveyed on whether they used certain ‘recreational drugs’ during the COVID-19 pandemic. We first use clustering analysis to categorize people according to their patterns of drug use choices. Then we compare these clusters in terms of mood and resilience. Lastly, subtractive analyses are applied to test whether individuals who use psychedelics in combination with other drugs had better mood self-assessment and resilience scores during the pandemic relative to those who use other drugs or are not drug users.

## 2. Methods

### 2.1. Recruitment and study design

Participants were recruited as part of the Great British Intelligence Test in two waves facilitated by advertisement through the BBC main page website. The first wave was between December 2019 and January 2020, and the second wave in May 2020. In May 2020 the recruitment phase was supplemented with a bespoke pandemic resilience questionnaire titled The Pandemic General Impact Scale (PD-GIS) (2), select items from the Patient Health Questionnaire [PHQ, (34)] and GAD-7 (35), a reduced version of the Big5 questionnaire (36) and a compulsivity questionnaire (37) to assess the impact that the onset of the pandemic and the first couple of months of lockdown in the UK had on the participants.

This study was run in accordance with the Helsinki Declaration of 1975, as revised in 2008. All procedures were approved by the Imperial College Research Ethics Committee (17IC4009). All participants provided informed consent prior to completing the survey.

### 2.2. Mental health assessment

We based our mood assessment on items from the extensively validated self-assessment scales: the Patient Health Questionnaire (PHQ) and the complete Generalized Anxiety Disorder Assessment (GAD-7) (34, 35). We selected 5 items from the PHQ questionnaire and the 7 items from the GAD-7 scale (See Supplementary material Part I for full questionnaire items). To capture mood over a longer period of time, we asked the participants to answer these questions pertaining to their mood in the month prior to the assessment (rather than 2 weeks as per the original scales). Additionally, we modified the scoring in order to capture a higher degree of granularity in their overall mood. Specifically, participants were asked to report symptoms over the preceding month from the time of assessment scored on a continuous scale from 0 to 6, as follows: ‘0-Never’, ‘1-Almost never’, ‘2-Once or twice a week’, ‘3-Several times a week’, ‘4-Daily’, ‘5-Hourly’, ‘6-More often’.

### 2.3. Personality and compulsivity assessment

Personality traits were quantified using an abbreviated scale comprising 18 (see Supplementary material Part I) out of the 44 items of the extensively validated Big-5 (36). Each item was a short phrase answered on a 5-point rating scale from −2 (strongly disagree) to 2 (strongly agree). Aspects of personality measured by this questionnaire classically reflect five factors: extraversion, agreeableness, conscientiousness, neuroticism and openness to experience. Compulsivity was quantified using a previously validated 15 item questionnaire (37). Each item was a short phrase answered on a 5-point rating scale from −2 (strongly disagree) to 2 (strongly agree). The two factors measured by the compulsivity questionnaire were perfectionism and reward drive.

### 2.4. Impact of the pandemic assessment

In May 2020 during our second stage of recruitment for the Great British Intelligence Test we were motivated by the pandemic context to develop in collaboration with psychiatrists, psychologists and neuroscientists a bespoke scale to quantify the self-perceived negative and positive impacts of the COVID-19 pandemic on daily life, as well as outlook, on multiple levels of psycho-socio-economic investigation. The Pandemic General Impact Scale (2) (PD-GIS) aimed to quantify self-reported feelings and behavior toward aspects of daily living that were specific to COVID-19 rather than general mental health, quality of life, optimism or resilience metrics. The seven factors of this questionnaire pertain to: disrupted lifestyle, health concerns, optimism, conflict at home, improved environment, more time for loved ones, a more relaxed lifestyle.

The scale quantifies three key aspects: (1) Aspects of positive impact. (2) Aspects of negative impact. (3) Outlook across 47 questions that map onto a 7-factor structure (see Supplementary material Part II). Each item is answered on a 5-point scale ranging from −2 (strongly disagree) to 2 (strongly agree).

### 2.5. Classifying drug use

In December 2020 and January 2021 participants were given the option to answer questions about their recreational drug use. These questions referred to recreational drugs that are illegal in the UK as opposed to the use of alcohol or tobacco. Based on whether they chose to answer this section or not, participants were split in to a number of drug use categories:

- Unknown/unwilling to disclose: this category encompasses participants that did not wish to disclose whether they have used drugs in the past.
- Non-drug users: this category encompasses those participants who reported they have never used a recreational drug in their lives.
- Drug users: this category encompasses those participants who reported they have used a recreational drug in their lives. This group was further split based on their exact drug use history.

### 2.6. Statistical analysis

#### 2.6.1. Gaussian mixture modeling

Gaussian Mixture Modeling (GMM) was used to determine the clusters of people by their self-reported drug use in a data-driven way. The clusters of different shapes and sizes, where each cluster is represented by a Gaussian distribution, can be accommodated by GMM, which is a flexible clustering modeling method. A probability score of belonging to a certain cluster is assigned by this algorithm to each datapoint (individual) characterised by N features (drugs they used during the pandemic). Then, the cluster with the highest probability defining that datapoint is assigned as the dominant cluster. In the present study, an individual drug user represented the datapoint, and uses of different drugs during the pandemic coded in binary terms, as well as whether the individuals were users before not during the pandemic, or not at all during their lifetime, were the features.

A 5-fold cross-validation method was used on a 80% train 20% test split (38) of the total data to identify the ideal number of clusters that could be modeled with the highest accuracy. The optimal number for the data was identified as 10 clusters (highest accuracy score), and the trained model was used to assign cluster probabilities and dominant cluster labels to all data points. The clusters were used as groups in the statistical analysis of mood and resilience.

#### 2.6.2. Factor structure of PD-GIS, little big-5, the compulsivity scale

Confirmatory factor analysis (CFA) was run in python using the factor analyzer package (39). The following factor structure was previously reported in publications as 7-factors for PD-GIS, and 2-factors for the compulsivity scale (37). For the reduced big-5 we employed an exploratory factor analysis with varimax rotation. The full set of questions, factor loadings and feature correlations are provided in the Supplementary material Part I.

#### 2.6.3. Mood self-assessment composite score

A factor analysis with one factor was run on the standardized mood self-assessment scores containing all questions asked. The first component identified this way was kept as the mood self-assessment composite score. This was done with the factor-analyser package in Python (39).

#### 2.6.4. Statistical differences between clusters

Chi-2 statistics were run to analyse demographic differences between clusters at different timepoints (See Supplementary material Part III). Differences between clusters were tested with 2-way ANOVA for group effects, dimension of mood/resilience effects, and their interactions; and subsequently where effects were identified cluster differences were tested with Tukey post-hoc tests. Ordinary Least Squares Regression (OLS) regression was used to model data whilst accounting for sociodemographic, lifestyle (inclusive of tobacco and alcohol use) and personality factors. All statistical analysis was performed using the statsmodels python package (40).

#### 2.6.5. Inferring effect size differences in mood/resilience based on belonging to a certain cluster

A linear regression with binary cluster labels as predictors was fitted to predict the standardized mood self-assessment/resilience scores after controlling for timepoint, sociodemographics, personality and lifestyle (inclusive of tobacco and alcohol use) variables. The model was then run through a type 2 ANOVA to infer the significance of each predictor contribution. The beta coefficients (SD units) are plotted alongside significance levels inferred via this latter analysis.

## 3. Results

Out of the *N* = 243,875 recruited participants who completed the Great British Intelligence Test between December 2019 and May 2020 (2), by December 2020, *N* = 95,441 provided their emails and gave permission to be recontacted for research purposes. These participants were recontacted in December 2020 and June 2021. At those subsequent timepoints the questionnaire delivered to participants was extended to include questions related to recreational drug use, which were not part of either the recruitment materials or follow up emails. A total of *N* = 22,633 participants responded in December 2020 and *N* = 17,231. Out of these, *N* = 22,304 and *N* = 16,903 participants completed all questionnaires of interest at the December 2020 and June 2021 timepoints respectively, totalling *N* = 30,598 unique respondents and *N* = 8,609 returning respondents. The original cohort sociodemographic characteristics are presented in Supplementary Figure S7.

### 3.1. Clustering recreational drug users by choice of drug

**Figure 1:**
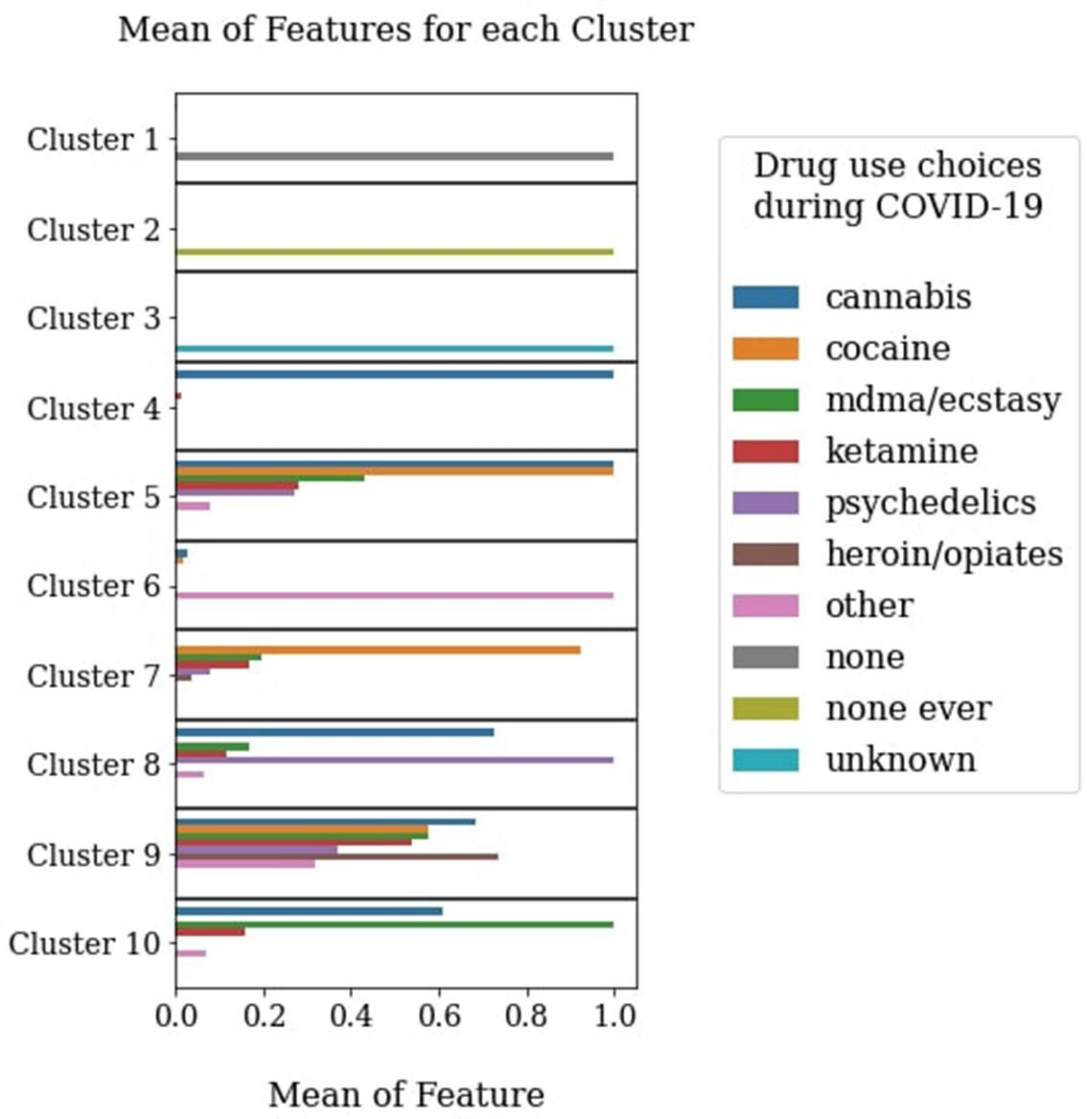
Data-driven clusters based on choices of recreational drug use during the COVID-19 pandemic. The *y* axis represents the feature loadings onto different clusters. The different clusters have been interpreted as follows: Cluster 1 (*N* = 6,161) – Drug use history but no pandemic use, Cluster 2 (*N* = 22,653) – Never used drugs, Cluster 3 (*N* = 7,593) – Unknown/Unwilling to disclose use, Cluster 4 (*N* = 1,538) – Cannabis users, Cluster 5 (*N* = 317) – Cannabis and cocaine users, Cluster 6 (*N* = 248) – users of ‘other’ drugs not covered in the list, Cluster 7 (*N* = 301) – Cocaine users, Cluster 8 (*N* = 216) – Psychedelics and cannabis users, Cluster 9 (*N* = 34) – Extreme polydrug users, Cluster 10 (*N* = 90) – MDMA/ecstasy and cannabis users.

10 clusters were identified as optimal when performing a 5-fold cross validation on the data with a 80% train set and 20% test set split. The Gaussian Mixture Model was applied to all data at this model order, to identify the loading of each feature onto each cluster and the probability of belonging to each of the clusters for each participant. The cluster with the highest probability was assigned as the dominant cluster for each participant (Figure 1). In Supplementary material Part III we illustrate that these clusters also vary significantly in their sociodemographics, lifestyle (inclusive of tobacco and alcohol use) and personality.

**Figure 2:**
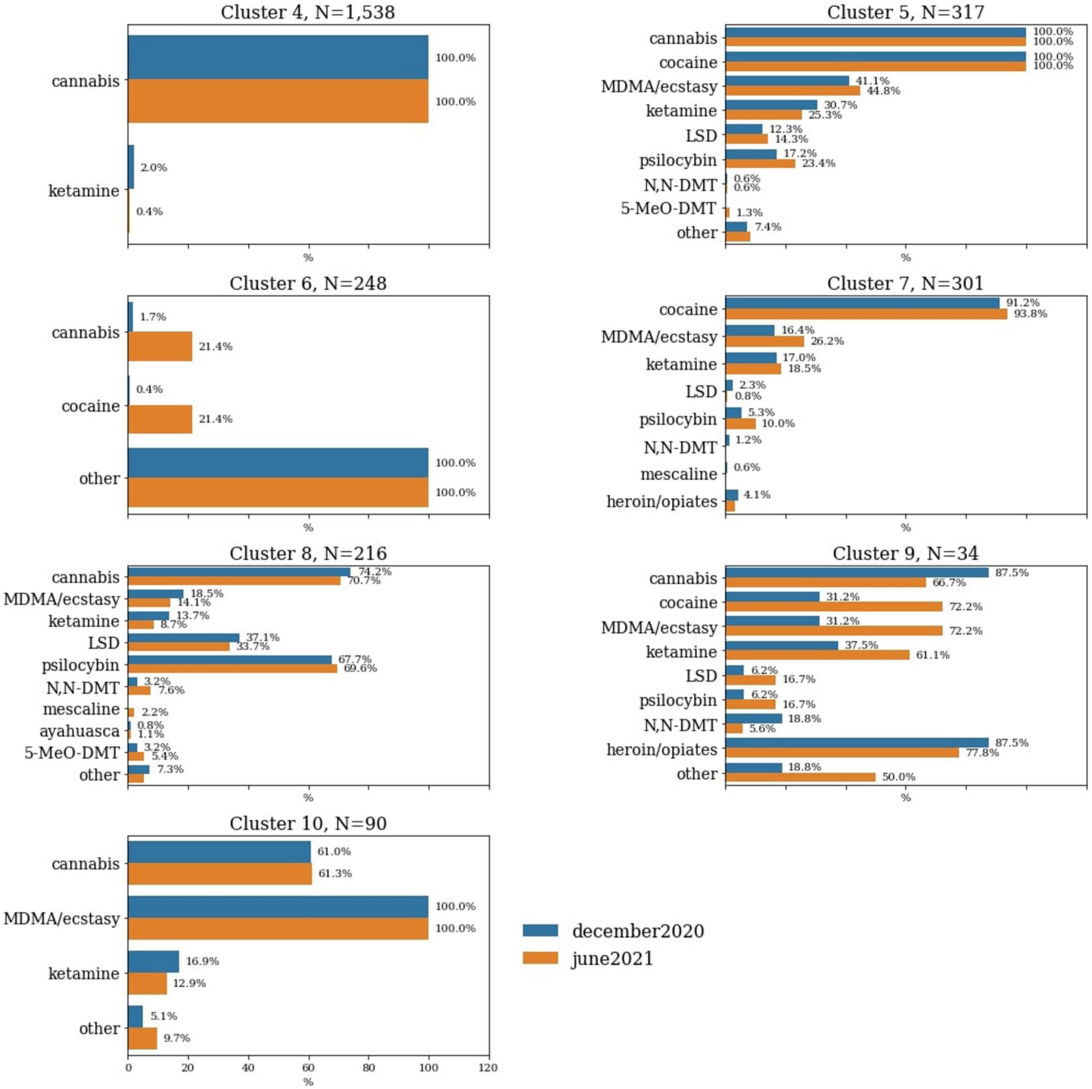
Percentage of choices of recreational drugs used during the pandemic within each of the clusters that represent active users. Only the clusters characterised by drug use features are represented in this figure. Percentages of individuals within specific clusters who have used certain drugs during the pandemic are illustrated based on the timepoint of assessment.

In Figure 2, we present a breakdown of all of the recreational drugs participants within each of the clusters reported using at different timepoints. We find that only for the under sampled cluster, Cluster 9, proportions of drugs use prevalence become notably shifted over time. More participants in Cluster 9 reported using cocaine, MDMA/ecstasy, ketamine and ‘other’ drugs by June 2021 than by December 2020.

Cannabis was by far the most prevalent recreational drug, being represented in multiple clusters. This was closely followed by cocaine, which had a cluster of people who primarily used only this drug, as well as a cluster paired with cannabis. The cluster representative for psychedelics users also had a strong cannabis use co-incidence, with over two thirds of members of this cluster having reported using it since the pandemic began. Notably, there was only a small proportion of the respondents (*N* = 46) who reported using psychedelics and no other drugs (Figure 2).

**Figure 3:**
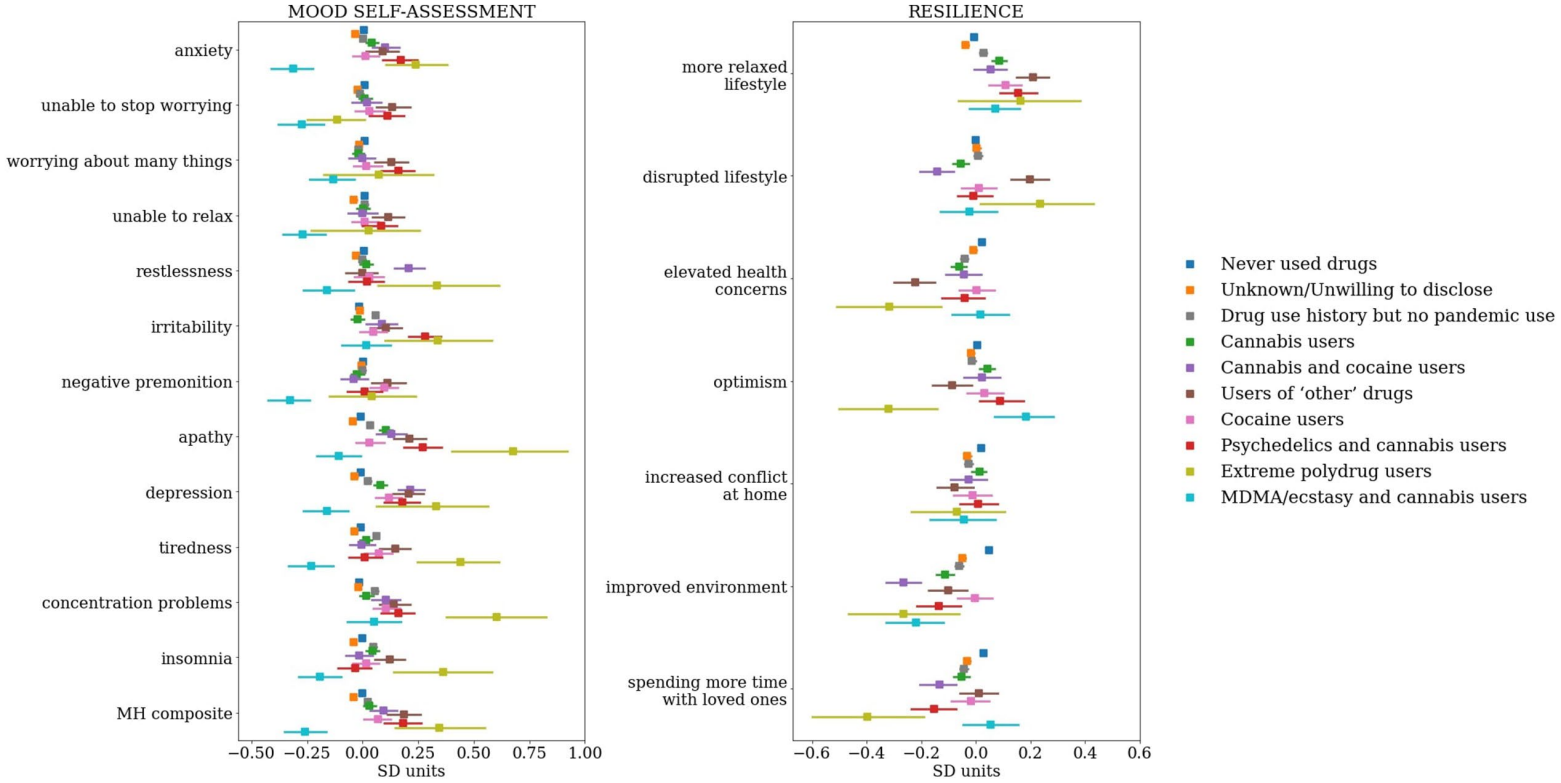
Group differences in individual mood self-assessment items and resilience latent factors. The answers received to mood questions were turned to scores, then using a linear regression model adjusted to timepoint, sociodemographic, lifestyle and personality factors. The resulting residuals were then standardized (*z*-scored). The latent factors derived from the PD-GIS questionnaire (2) have been adjusted to using a linear regression model with timepoint, sociodemographic, lifestyle and personality factors as predictors. The resulting residuals were then standardized (*z*-scored) and plotted. On both panels, points represent the cluster mean, error bars represent standard error of mean (SEM). The left panel illustrates group differences in individual dimensions of mood, whereas the right panel illustrates group differences in individual dimensions of resilience.

### 3.2. Mental health and resilience differences between clusters

We used a 2-way ANOVA to test for significant differences in mood self-assessment and resilience (Figure 3). On mood self-assessment scores we found a significant effect of cluster (*F*_(9,485,680)_ = 35.15, *p* < 0.001) and an interaction between cluster and variable (*F*_(108,485,680)_ = 1.95, *p* < 0.001). By applying Tukey post-hoc tests, prior to correction for multiple comparisons, we found that some of these effects were driven by group differences between the psychedelics and cannabis cluster and other groups. In particular, differences between psychedelics and cannabis users vs. MDMA/ecstasy and cannabis users on anxiety, differences between psychedelics and cannabis users and cannabis only users/those who never used drugs/those unwilling to disclose their drug use on irritability, psychedelics and cannabis users vs. those with drug use history but no pandemic use/those who never used drugs/those unwilling to disclose their use on apathy, and psychedelics and cannabis users vs. MDMA/ecstasy and cannabis users on their overall mood scores. However, we note that none of these survived correction for multiple comparisons. Full *post-hoc* analysis can be found in Supplementary material Part III.

On resilience scores we also found a significant effect of cluster (*F*_(9,261,051)_ = 9.32, *p* < 0.001) and an interaction between cluster and variable (*F*_(54,261,051)_ = 4.77, *p* < 0.001). However, we did not see differences pertaining to the resilience of psychedelics and cannabis/MDMA and cannabis users driving any main cluster effects (full post-hoc analysis can be found in Supplementary material Part III).

**Figure 4:**
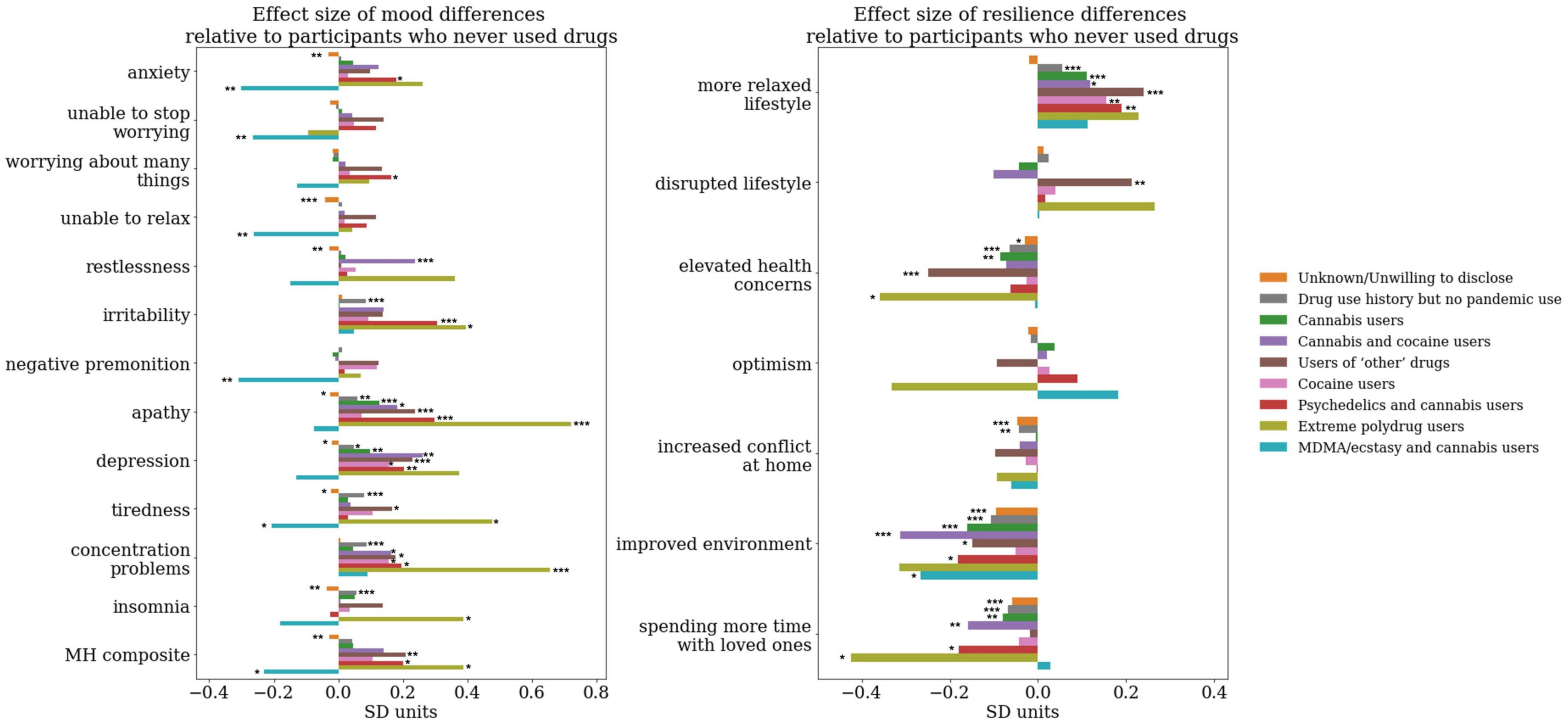
Effect size difference relative to the cluster representing participants who never used any drug whilst accounting for the effect of timepoint, sociodemographic, lifestyle and personality factors. A linear regression model was run on the adjusted MH scores/resilience latent factors with each binary dummy-variable representing cluster labels as predictors. The cluster of participants who reported never having used drugs has been kept as the reference. The y axis represents the beta coefficients resulting from the regression associated with the effect size of each of the groups, whereas the significance star annotations represents the statistical significance of this effect size derived from running an ANOVA on the linear regression model. *, *p* < 0.05; **, *p* < 0.01; ***, *p* < 0.001.

First, using linear modeling, we adjusted every single mood self-assessment and resilience factor score to timepoint, demographics, lifestyle (inclusive of use of tobacco and alcohol) and personality. Specifically, for use of tobacco and alcohol, we adjust to the number of alcohol units consumed in a week and the number of cigarettes smoked in a day. Use of tobacco and alcohol was evident in each of the data-driven clusters we present (see Supplementary material Part III for group level differences), therefore justifying the need to account for the confounding effects of using these substances in conjunction with other recreational drugs. We then run a linear regression on the adjusted smood self-assessment and resilience scores with the cluster labels as binary predictors (except the cluster indicating participants who never used drugs in their lifetime, which acted as a reference) in order to identify effect size differences in the reports of each cluster relative to those who never used drugs. We use Sawilowsky’s updated version of Cohen’s notion of effect sizes (0.1 SD = very small, 0.2 SD = small, 0.5 SD = medium, 0.8 SD = large, 1.2 SD = very large and 2.0 SD = huge) for interpreting the magnitude of the effects observed in our data (41, 42). We identify a range of effects in the small to medium range of different drugs/associations of drugs on mood, as well as resilience. Most notably, for mood dimensions, the MDMA and cannabis users and the participants unwilling to disclose their use were the only clusters displaying an association with better mood (lower mood-self assessment scores) relative to those who never used drugs (Figure 4).

For the MDMA and cannabis users we observe significantly less anxiety (effect size −0.32SD, *F*_(1,37,373)_ = 8.97, *p* < 0.001), inability to stop worrying (effect size −0.28SD, *F*_(1,37,378)_ = 7.07, *p* = 0.01), inability to relax (effect size −0.28SD, *F*_(1,37,374)_ = 6.85, *p* = 0.01), and negative premonition (effect size −0.33SD, *F*_(1,37,379)_ = 9.57, *p* < 0.001), tiredness (effect size −0.23SD, F_(1,37,379)_ = 4.5, *p* = 0.03), and overall better mood (effect size −0.26SD, *F*_(1,37,360)_ = 5.87, *p* = 0.02). We observe an opposite pattern for psychedelics and cannabis users, with significantly higher anxiety (effect size 0.17SD, *F*_(1,37,373)_ = 5.59, *p* = 0.02), worrying about too many things (effect size 0.15SD, *F*_(1,37,379)_ = 4.45, *p* = 0.03), higher irritability (effect size 0.3SD, F_(1,37,379)_ = 17.41, *p* < 0.001), apathy (effect size 0.28SD, *F*_(1,37,370)_ = 15.53, *p* < 0.001), depression (effect size 0.19SD, F_(1,37,379)_ = 6.97, *p* = 0.01), concentration problems (effect size 0.18SD, F_(1,37,379)_ = 6.22, *p* = 0.01) and overall mood problems (effect size 0.18SD, *F*_(1,37,360)_ = 6.57, *p* = 0.01).

Relative to individuals who never used drugs in their lifetime we observe less agreement to statements suggesting the pandemic led to an improved environment in both psychedelics and cannabis (effect size −0.18SD, *F*_(1,37,293)_ = 6.07, *p* = 0.01) and MDMA and cannabis users (effect size −0.27SD, *F*_(1,37,293)=_5.75, *p* = 0.02). Psychedelics and cannabis users also reported a more relaxed lifestyle (effect size 0.19SD, F_(1,37,293)_ = 7.05, *p* = 0.01) and spending less time with loved ones (effect size −0.18SD, F_(1,37,293)_ = 5.82, *p* = 0.02).

**Figure 5:**
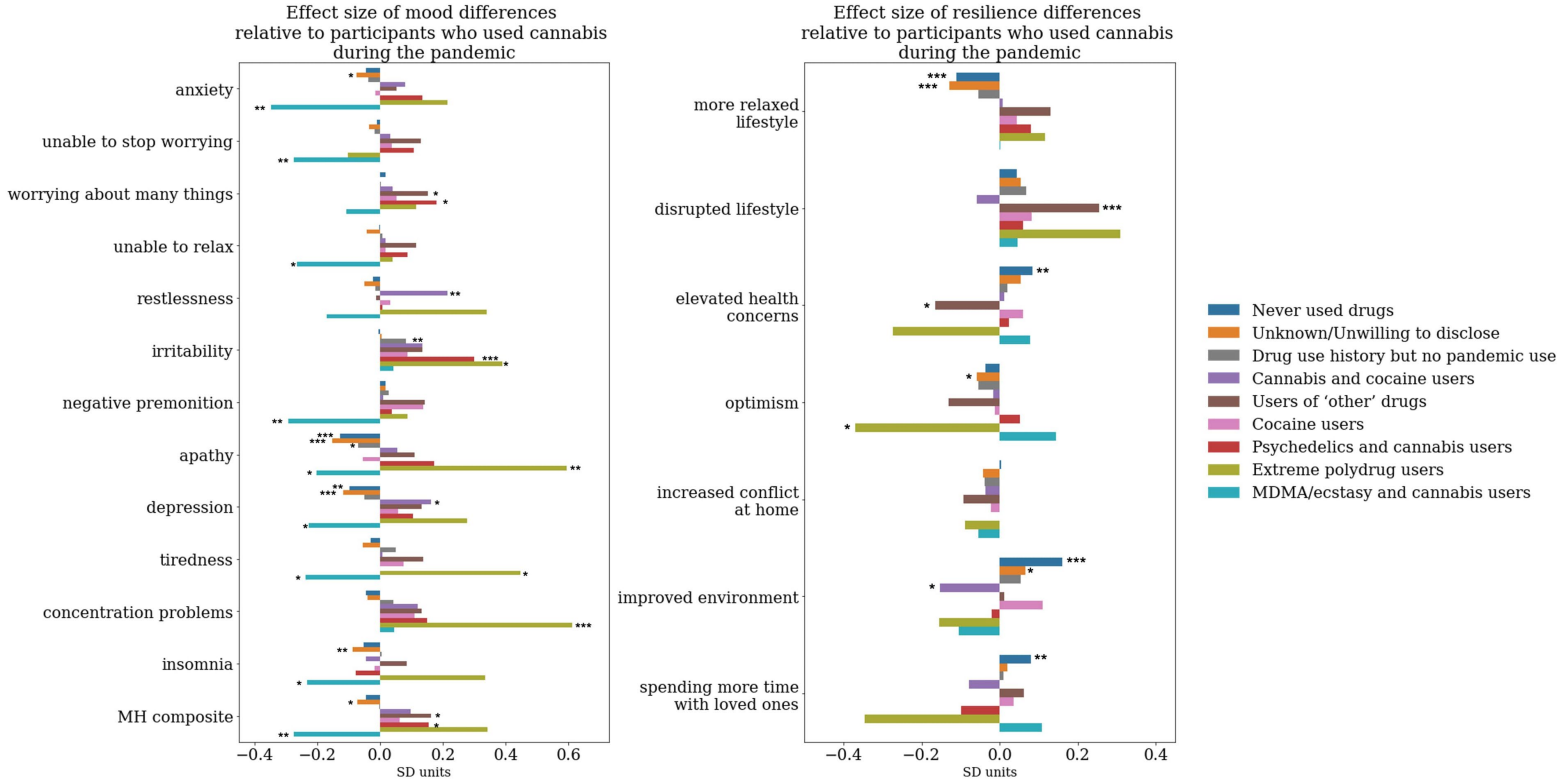
Effect size difference relative to the cluster representing participants who took cannabis during the pandemic whilst accounting for the effect of timepoint, sociodemographic, lifestyle and personality factors. A linear regression model has been run on the adjusted MH scores/resilience latent factors with each binary dummy-variable representing cluster labels as predictors. The cluster of participants who reported using cannabis during the pandemic has been kept as the reference. The y axis represents the beta coefficients resulting from the regression associated with the effect size of each of the groups, whereas the significance star annotations represents the statistical significance of this effect size derived from running an ANOVA on the linear regression model. *, *p* < 0.05; **, *p* < 0.01; ***, *p* < 0.001.

To investigate the magnitude of the effect size differences of the clusters of psychedelics and cannabis, and MDMA and cannabis users respectively, relative to the cluster composed of cannabis users only, we repeated the analysis illustrated above with all other clusters as predictors apart from the cannabis users only cluster (Figure 5).

To a large extent the effects on mood dimensions relative to cannabis users were similar to the effects we observe relative to non-users. MDMA and cannabis users, relative to cannabis only users, had significantly lower levels of anxiety (effect size −0.36SD, *F*_(1,37,373)_ = 10.57, *p* < 0.01), inability to stop worrying (effect size −0.28SD, *F*_(1,37,378)_ = 6.73, *p* = 0.01), inability to relax (effect size −0.27SD, *F*_(1,37,374)_ = 6.17, *p* = 0.01), negative premonition (effect size −0.28SD, *F*_(1,37,379)_ = 7.61, *p* = 0.01), apathy (effect size −0.21SD, *F*_(1,37,370)_ = 3.86, *p* = 0.049), depression (effect size −0.3SD, F_(1,37,379)_ = 4.76, *p* = 0.03), tiredness (effect size −0.25SD, F_(1,37,379)_ = 5.08,*p* = 0.02), insomnia (effect size −0.24SD, *F*_(1,37,378)_ = 4.65, *p* = 0.03) and overall mood problems (effect size −0.29SD, *F*_(1,37,360)_ = 6.98, *p* = 0.01). Psychedelics users, on the other hand, had significantly higher levels, relative to cannabis only users, of worrying about too many different things (effect size 0.18SD, *F*_(1,37,379)_ = 5.63, *p* = 0.02), irritability (effect size 0.3SD, F_(1,37,379)_ = 16.07, *p* < 0.001), and overall mood problems (effect size 0.15SD, *F*_(1,37,360)_ = 3.99, *p* = 0.049).

Neither psychedelics and cannabis or MDMA and cannabis users displayed significant effect size differences relative to users of cannabis only in any domains of pandemic-specific resilience.

## 4. Discussion

Our results illustrate that recreational drug use during the March 2020–June2021 period can be framed by 10 distinct clusters defined in a data-driven way on the basis of the combinations of drugs participants reported using (Figure 1). These distinct clusters indicate preferences for certain substances over others (eg primarily cannabis or primarily cocaine users), or a preference for an associated pattern of use (eg psychedelics and cannabis, cocaine and cannabis, MDMA and cannabis, extreme polydrug use). Most importantly, they are characterised by different levels of mood and resilience profiles during the COVID-19 pandemic.

Perhaps most strikingly, all but one drug cluster had consistently negative associations with mood, the exception being the MDMA plus cannabis cluster, where we observed positive associations with mood. Conversely, while we expected to see those who chose to use psychedelics as having better mood and higher resilience than users of other drugs or participants who never used drugs, this was not the case. In fact, we observed, contrary to our hypothesis, that those who used psychedelics indicated higher levels of anxiety, worrying about different things, irritability, apathy, depression and overall mood problems relative to individuals who never used drugs. We also found that while they reported a more relaxed lifestyle as a result of the pandemic, they disagreed that the pandemic effects could be associated with an improvement in the environment and spent less time with loved ones. Considering almost all psychedelics users in our sample were polydrug users, especially in association with cannabis, we carried out the same analysis but this time calculating effect size differences relative to the cannabis only cluster. This produced similar results; specifically, relative to those who only used cannabis, those who used psychedelics plus cannabis reported higher levels of anxiety, worrying about different things, irritability and overall mood problems; though depression levels were not significantly different.

Our findings concerning the naturalistic use of psychedelics diverge from the anticipated outcomes, but in doing so help contextualize the optimistic landscape painted by prior investigations. Prior to the COVID-19 pandemic the overwhelming majority of studies found a positive association between naturalistic psychedelics use and dimensions of mental wellbeing. For example, in a prospective naturalistic study of depressed participants, reductions in depressive symptoms were observed for up to a month after using a psychedelic (43). A cohort of Indigenous people in Canada and the United States reported the incidence of fewer depressive symptoms, anxiety and stress within a month after taking a psychedelic relative to a previous baseline (44). Subjective improvements in depression and anxiety were also reported to be associated with the naturalistic use of mescaline (26). This effect has been observed to increase as a function of higher psychedelics exposure, up to a ceiling (45). Ceremonial use of psychedelics such as ayahuasca has also been linked to improvements in depression lasting for up to 6 months (46). Interestingly, significant reductions in anxiety and depression were also identified in ayahuasca-naive participants who underwent such a ceremony (29). Using psilocybin truffles in supportive group settings has been linked to a reduction in anxiety (47, 48). Those who microdosed psilocybin also report short to medium term improvements in mood and mental health (49). It is worth noting that all of these investigations have demonstrated enhancements in various dimensions of mental wellbeing relative to participants’ own baseline, that is, in a within-subjects experimental design, instead of comparing those who used psychedelics versus those who used other drugs, or never used drugs in their lifetime. While prior findings offer valuable insights into the potential of psychedelics to ameliorate low mood following experiences in naturalistic settings, it remains unclear whether the magnitude of these improvements was substantial enough to match (or go beyond) the mood levels of non-drug users or users of other drugs, thus potentially resulting in psychedelics users as having better mood than other subsets of the population.

Findings concerning naturalistic psychedelics use during COVID-19 pandemic offer a more particular basis for drawing direct comparisons with our results. A few studies did attempt to explore the relationship between use of psychedelics and mental health outcomes during the COVID-19 pandemic and reported that people did indeed use psychedelics with the intention of better coping with pandemic stresses (50, 51) and that lifetime use of psychedelics was associated with better mental health indicators (52). Psychedelics have even been proposed for the purpose of treating mental health conditions due to or aggravated by COVID-19 viral infection (53). (50) indicated two thirds of their sample claimed that pandemic use of psychedelics helped them deal with the global situation better. (51) reported those who used psychedelics (and also MDMA) used problem-focused coping strategies in response to the global crisis more often than non-users and (54) (on a different cross-sectional analysis of data from the same study as (51)) reported that users of psychedelics, especially regular ones, reported less psychological distress than non-users. However, a survey carried out in the United States of America (USA) found an association between psychedelics use, namely between past use of psilocybin mushrooms, and worse mood self-assessment scores at the time of assessment (55). Our findings agree with those of Matzopoulos et al. (55), who employed similar mood self-assessment scales as ours to survey the USA general population, but differ to those of (53) who used different mental wellbeing assessments and carried out the survey mainly in Spain and Brazil through snowball sampling on social media.

The obvious question prompted by our results is - why is it that we are not seeing, in the light of the positive effects demonstrated by clinical studies on psychedelics as well as naturalistic surveys, a positive association between naturalistic psychedelics use and mental health during times of crisis? Some of the earliest theories aimed at explaining the variability in the effects of psychedelics have converged around the concept of set and setting (56), and their potential for acting as non-specific context amplifiers (57). *Set and setting* refer to the mindset and intention of the individual experiencing the psychedelic, and the characteristics of the environment where the experience is taking place, respectively, (58, 59). In modern clinical studies, for example, in order to ensure an optimal “set,” careful attention is given to provide participants with psychological support before, during and after the experience, and to ensure an optimal “setting” the same attention is paid to the environment in which the psychedelic intervention takes place (60). Given the importance that this notion of *set and setting* is given in the clinical environment, it is potentially unsurprising that the benefits seen there are not transferred to the naturalistic setting where these variables may not be controlled optimally. Not only was the context of the experiences captured in our study not explicitly therapeutic, but it was also heavily marked by a global mental health crisis as well as significant disruptions in the immediate environment of individuals as a result of the COVID-19 pandemic. As demonstrated by our findings, lifestyle disruptions were common in the majority of drug use clusters, as was spending less time with loved ones, and in certain instances an increase in conflict at home. These factors undoubtedly had imminent effects on the *set and setting* of psychedelic experiences during that period, and it is reasonable to infer that the disruptive context would have likely contributed to the associations we observe between use of psychedelics and mood self-assessment scores.

Another major difference between the present study and the previous literature is the socio-cultural context in which the psychedelics have been taken. Different countries will have different cultural acceptance, cultural significance and general stigma around psychedelic drug use to that of the UK. Adding to this, local regulation due to the pandemic may have produced differences in access to drugs. Since context can influence psychedelic experiences in a naturalistic setting (61), it cannot be ruled out that these factors contributed to the outcomes we report. More specifically, it is not excluded that psychedelics use in the UK (where strict lockdown and infection control guidelines were employed) during the COVID-19 pandemic could have led to the amplification of general distress, which would have in turn influenced the outcome of psychedelic experiences. It is worth noting, in support of this perspective, that our findings align with those emerging from the USA during a similar timeframe (54), but not to findings from Spain or Brazil (53). While political climate, healthcare access and infection control guidelines differed in these countries and undoubtedly affected their population in differential ways, the specific socio-cultural nuances related to the use of psychedelics also need to be considered. Neither the USA or the UK have a recent history of general cultural acceptance of psychedelics use, nor were these substances legal for recreational use at the time of assessment. Given the above, there is a question as to what extent our findings generalize outside the borders of the UK or the USA.

Despite the issues with naturalistic psychedelic drug use studies with regards to uncontrolled set and setting, the majority of these studies (both the within-subjects design pre-pandemic studies as well as the cross-sectional study of (53) carried out during the pandemic) still report a positive association with mental wellbeing. Another key difference between our study and most of the other studies mentioned above that could explain the reported outcomes is the nature of participant recruitment methodology. With few exceptions, the above mentioned studies advertised psychedelics-related research in psychedelic-profiled social media groups. Members of these groups who respond to such advertisements may not be representative of those groups or of the people who use drugs more generally. Recruitment bias has been previously called out to be a confound in surveys specifically recruiting psychedelics users, since openly advertising such studies opens the door for self-selecting participants who, on the basis of enthusiasm, positive experiences, and/or desire to contribute to research to advance a global societal movement, might not yield objective datasets and consequently allow scientists to draw the right conclusions (62–65). In particular, biases in sampling related to positive past experiences, but also as pertains to a prospective study-related expectation that there may be a mental health benefit associated with psychedelic use, could confound the results.

There are other reasons why we might observe different outcomes. None of the past studies looking at naturalistic use of psychedelics used a data-driven approach to cluster the use of psychedelics with the use of other drugs alongside to expose effects of common drug interactions. Additionally, none directly compared the data of psychedelics users from clusters derived in this way with data from users of other drugs who do not use psychedelics. Notwithstanding the debate on what constitutes a psychedelic to begin with (66), in past studies classical and non-classical psychedelics were often assessed concomitantly under the umbrella term of ‘psychedelics’, regardless of the use of other drugs. This is an important consideration as drugs with different pharmacology and subjective effects could produce varying outcomes. Our own data, for example, reveals that within the cluster of individuals who primarily used psychedelics and cannabis during the pandemic a small proportion have also used MDMA. (53) also included MDMA users as part of their ‘psychedelics users’ group in a cross-sectional analysis of users vs. non users of psychedelics. Notably though, MDMA users were more prevalent in their sample compared to ours. The implications of this observation are significant, particularly considering that our results demonstrate a contrasting association between the use of primarily MDMA and cannabis (in the absence of psychedelics) and dimensions of mental wellbeing. Specifically, individuals within this cluster exhibited better mood relative to those who never used drugs in their lifetime at the time of assessment, drawing further attention to the question pertaining to whether the proportion of MDMA experiences captured within the ‘psychedelics’ label is what could be driving the differential outcomes.

Our analysis focuses on modeling the choices of drugs used specifically during the pandemic timeframe, rather than the frequency or underlying motivations for these choices. It is not excluded that using psychedelics in conjunction with other substances (e.g., as was evident in our sample that the majority of those who chose to use psychedelics were polydrug users and also chose to use cannabis, whereas a minority used other substances too) during a global mental health crisis cancels out therapeutic effects that could potentially be derived from naturalistic experiences with psychedelics. The effects on mental wellbeing would be contingent on prior experiences, the frequency of use of each individual substance, on their dosage, whether they were consumed together or separately and under which circumstances (as discussed above). Within our data-driven clusters, we anticipate capturing a diverse range of such patterns. For instance, some individuals may use cannabis daily while only occasionally using psychedelics, whereas others may frequently engage in microdosing psychedelics while rarely using cannabis. These patterns might have been in place prior to the pandemic or adopted because of it. Furthermore, the dosage of these substances is expected to have varied among the individuals surveyed, in turn leading to varying acute and long-term effects mediated by different degrees neurobiological changes and the resulting intensity of those experiences. Additionally, it is reasonable to assume that the individuals we surveyed would have used psychedelics or cannabis for a variety of reasons not limited to self-medication, recreationally, as a social catalyst, or for spiritual purposes. These motivations would have differentially influenced their mood post-experience. Moreover, whether therapeutic effects even exist subsequent to naturalistic use, they might be short lived [maximum documented has been 6 months post experience by Ruffell et al. (46)] and our participants might have had their experiences months apart from answering our survey, and/or modulated by subsequent use of other substances (or even prescription medications) not limited to the ones captured in our assessment.

A core difficulty in interpreting our findings is to do with potential causal links between psychedelics use, mood self-assessment and resilience metrics in the general population. Contextual variability affecting the outcome of drug-induced experiences aside, it might also be that people with poor mental health to begin with take psychedelics, and therefore their perceived improvements in mood might be visible only in a within-subjects study design rather than something comparable across large segments of non-drug using population. It has been previously documented that people with poor mental health indeed engage in drug use to self-medicate (67), and it is possible that individuals experiencing low mood during the pandemic turned to psychedelics for this reason. Drawing upon prior research findings, psychedelics could have indeed contributed to improvements in the mood of our participants, suggesting that our results could be driven by baseline differences in mood that preceded the experiences rather than a lack of benefits derived from psychedelics use. However, this observation raises the possibility that the improvements in mood resulting from psychedelic experiences in naturalistic settings may not reach a level that equals or surpasses the mood levels of individuals with no history of drug use. This could be the case particularly during times of global crisis but also beyond. On the other hand, it is also plausible that our results are influenced by fundamental differences in dimensions other than mood between individuals who choose to use psychedelics and those who use other drugs or have no history of drug use, which were not captured in our assessment –genetics, brain and body health status, co-morbidities and psychological history to name a few. To add to the complexity, there might be differences between people who chose to use psychedelics during the pandemic versus people who chose to use psychedelics before the pandemic, or those who will choose to use psychedelics after the pandemic. These underlying differences could potentially contribute to baseline variations in mental health levels that persist regardless of any potential positive effects on mood resulting from psychedelic experiences in naturalistic settings. It is also possible that a combination of these factors – limited magnitude of psychedelic effects on mood as well as fundamental differences between user groups – might be at play in determining cross-sectional differences. Importantly, the severity of the pandemic impact highlights that it is crucial to consider these factors when evaluating survey data on psychedelic use collected as the world recovers from disruption.

Our results reflecting a positive association between MDMA/ecstasy and cannabis use are interesting and surprising, though we would like to exert caution interpreting them and their generalisability to the wider spectrum of MDMA users. The within group variation was high and the sample size of this cluster low (*N* = 90) relative to the other clusters, consistent with previous work reporting a decrease in typical ‘party’ drugs such as MDMA/ecstasy during the COVID-19 pandemic (68), thus the effects we report reflect a bias toward more extreme values in this particular case. Additionally, as this is a self-report sample we cannot exclude that some mood self-assessments might be inaccurate either due to dishonesty or as a function of personal awareness, and in lower samples this raises higher levels of skepticism in interpretation. However, if these results were to be replicated in a larger sample, a possible interpretation would be that since acute MDMA effects are associated with feelings of bonding, love and social connection (69), MDMA-catalyzed social interactions during the pandemic could have acted as a protective mechanism on the mood of users, relative to those who did not have these experiences.

Our study has a number of strengths which confers us the ability to draw the present inferences with confidence. First and foremost, we present one of the few studies assessing effects of naturalistic use of psychedelics, MDMA and other drugs in the absence of recruitment bias toward social media groups discussing psychedelics, since the questions which participants were asked were not advertised either at the time of recruitment or prior to the follow-ups, and our study was never advertised on social media channels pertaining to drug use/related activities. What’s more, in our analysis we employ a diverse population inclusive of a very large control population who has never used drugs but has answered the same questions during the same timeframe. We also acknowledge that drug use behavior is dynamic, and that psychedelics are most often not used in isolation, with cannabis specifically being the substance of choice that participants might use during the same period of time, thus making it generally difficult to disentangle specific drug effects when these are being used naturalistically.

There are also certain limitations our work possesses, in part owed to the main reason our study is unique - that the study design was not drug-use specific. Recreational drug use is a prevalent lifestyle decision that covers choices of both licit and illicit drugs. In our study we only model choices of recreational drugs that are illicit in the UK, and control for the frequency of otherwise licit drugs alcohol and tobacco in the multivariate analysis. This distinction in our analysis is not to imply that licit drugs are less harmful than illicit drugs. While characterization of harm based on this dichotomy is beyond the scope of this manuscript, we note the extensive evidence to suggest that alcohol and tobacco use is at least as harmful as is the use of the other recreational drugs analysed in this study (70–77). Furthermore, due to a variety of reasons not limited to stigma, it is difficult to recruit drug users from the general population, since this category of individuals, particularly if they have a high level of polydrug use, are less likely to engage with traditional survey methods (78). Therefore, we might have undersampled the population with problematic use (as reflected by the extreme poly-drug users cluster having the lowest number (*N* = 35) of respondents). With regards to polydrug use, at the point when the present data were collected we have not collected extensive details about participants (who declared having used a drug at least once) drug use history prior to the COVID-19 pandemic, thus diminishing our ability to draw inferences about an accumulation of experiences throughout their lifetime potentially influencing mood and/or resilience during the pandemic timeframe. We expect that within our data-driven clusters people would have had different frequencies of specific drug use prior as well as during the pandemic. However, we have not collected extensive details about participants drug use frequencies during the pandemic timeframe itself, and are therefore not able to assess the relationship between frequency of use and effects on mental wellbeing in the present study. These dimensions of mental wellbeing, too, were studied independent of clinical diagnoses of either psychiatric or neurological disorders, and given the large dataset it is not excluded that some participants might have had clinical levels of anxiety, depression, post-traumatic stress disorder or even diagnosed substance use problems. Other factors such as living conditions, quality of interpersonal relationships, and various ramifications within the umbrella term of ‘social misery’ have not been addressed in the present analysis, and it is not excluded that there would potentially be significant differences in these aspects between the different drug use clusters, with effects on mood and resilience. Lastly, we highlight that our sample, although large, is >90% represented by members of the British population, and therefore it is possible that associations between naturalistic use of psychedelics (as well as other recreational drugs) might look different for individuals residing in other parts of the world.

The effects of naturalistic use of recreational drugs on mood, and mental wellbeing more generally, warrant further attention and research since these substances are growing increasingly more popular and available beyond clinical setups. In particular, we advise for other studies to try to replicate our findings in existent/future large datasets that also collected data on naturalistic drug use. Concerning our results on MDMA and cannabis use, this is particularly important, since unintended “hype” might arise as a result of the noted positive associations, which if it results in increased naturalistic use to self-medicate for mood disturbances is of concern due to potential toxicity of prolonged MDMA exposure (79). Concerning our results related to psychedelics use, the effect of different contexts and more granular investigations of how particular set and setting features influence the quality of the experience and its long term outcomes might prove useful in advancing our understanding of psychedelics effects in naturalistic settings and inform the development of harm reduction guidelines. We note special attention might be given to cultural contexts, as perceptions of psychedelics are tied to ethnographic backgrounds (80). Amongst key survey/experimental design approaches we highlight the importance of selecting participants as agnostically as possible in relation to the study outcome, *and where possible not disclosing the study hypothesis*. Aiming to recruit control-participants and even users of other drugs, too, and test them on objective mental health metrics alongside psychedelics users would also strengthen and contextualize conclusions. Perhaps even more importantly, collecting non-psychedelic drug use history, lifestyle and personality data in such studies and then adequately accounting for these variables at the analysis stage might yield more holistic insights as to what exactly psychedelics modulate and in relation to/independent of what other factors. The underlying effects on brain chemistry and activity where drug interactions of potent pharmacological agents are used in conjunction with one another is also something that might be worth exploring in association with behavioural metrics. Specifically, attention ought to be given to the intersection of different patterns of using distinct drugs in parallel, both licit and illicit.

### 4.1. Conclusion

In the present study we found a positive association of MDMA and cannabis use, but did not find a positive association of psychedelics and cannabis use with better mood and resilience during the COVID-19 pandemic in users relative to those who only used cannabis during the same timeframe or never used drugs in their lifetime. Mapping out with precision how sociodemographic and lifestyle factors (inclusive of socio-cultural context, use of alcohol or tobacco, being a single drug or polydrug user, history of drug use, personality type, compulsivity) or drug experience factors (set and setting during the acute experience, dosage, interaction between drugs used at the same time/subsequently) are driving the associations observed between data-driven clusters of drug use choices and mood is challenging due to the complex interplay among all of these variables. While we find positive associations with mood in the MDMA and cannabis use cluster, we indicate these results must be replicated before stronger conclusions can be drawn. In the case of psychedelics, it is reasonable to infer based on past studies that in appropriate conditions (set and setting, clear intention, appropriate dose) psychedelics taken in naturalistic settings could potentially lead to improved mood and wellbeing in specific individuals. However, contrary to our initial hypothesis, our analysis provided evidence that individuals who used psychedelics during a global crisis did not exhibit better mood and resilience compared to those who used other drugs or did not use drugs in their lifetime. We posit that psychedelics effects and associations with mood and resilience in naturalistic settings are variable rather than unequivocally positive, and future research should aim to map out with greater precision what factors predict outcomes at either end of the positive–negative spectrum, and how these outcomes inform cross-sectional differences between users and non-users.

## Data availability statement

Data presented are part of a longitudinal study that is still ongoing, and can only be shared via institutional agreements in compliance with local GDPR guidelines. Data sharing inquiries should be directed to Prof. Adam Hampshire: a.hampshire@imperial.ac.uk

## Ethics statement

This study was run in accordance with the Helsinki Declaration of 1975, as revised in 2008. All procedures were approved by the Imperial College Research Ethics Committee (17IC4009). All participants provided informed consent prior to completing the survey.

## Author contributions

MB, WT, and AH: conceptualisation. MB and AH: methodology. MB: investigation, visualization, and writing—original draft. AH: supervision. WT, PH, and AH: software. MB, WT, PH, and AH: writing—review and editing. All authors contributed to the article and approved the submitted version.

## Funding

MB is supported by the Medical Research Council Doctorate Training Programme at Imperial College London. WT is supported by the EPSRC Centre for Doctoral Training in Neurotechnology. PJH is, in part, supported by the National Institute for Health Research (NIHR) Biomedical Research Centre at South London and Maudsley NHS Foundation Trust and King's College London. AH is supported by the Biomedical Research Centre at Imperial College London.

## Conflict of interest

AH is owner and director of Future Cognition LTD and H2 Cognitive Designs LTD, which support online studies and develop custom cognitive assessment software respectively. PH is co-owner and director of H2 Cognitive Designs LTD and reports personal fees from H2 Cognitive Designs LTD outside the submitted work.

The remaining authors declare that the research was conducted in the absence of any commercial or financial relationships that could be construed as a potential conflict of interest.

## Publisher’s note

All claims expressed in this article are solely those of the authors and do not necessarily represent those of their affiliated organizations, or those of the publisher, the editors and the reviewers. Any product that may be evaluated in this article, or claim that may be made by its manufacturer, is not guaranteed or endorsed by the publisher.
