# Supplementary material for: Associations between the use of psychedelics and other recreational drugs with mental health and resilience during the COVID-19 pandemic
Source: Front Psychiatry. 2023 Jun 15;14:1184681. doi: 10.3389/fpsyt.2023.1184681 (PMC10307955; doi:10.3389/fpsyt.2023.1184681)
Supplement: Supplementary file 1 [file Data_Sheet_1.docx]

Supplementary Material

Associations between the use of psychedelics and other recreational drugs with mental health and resilience during the COVID-19 pandemic

**Maria Bălăeț*, William Trender, Peter J. Hellyer, Adam Hampshire**

**Correspondence:** Corresponding Author: [m.balaet17@imperial.ac.uk](mailto:m.balaet17@imperial.ac.uk)

These supplementary materials are split into six parts:

- **Part I.** detailing the questions asked in the study, and where applicable the factor structure of the questionnaires
- **Part II –** sociodemographics of the GBIT cohort
- **Part III.** Supplementary tables of statistical analyses outputs
- **Part IV.** Supplementary post-hoc analysis of main results
- **Part V –** Full regression output – removing the effects of confounds
- **Part VI –** Full regression output – investigating the effects of drug use

**PART 1.**

***Demographics***

1. How old are you?
   1. From 0 to 100
2. What sex have you been assigned?
   1. Male
   2. Female
   3. Other
3. To ensure we have a representative sample of the population, please indicate your ethnicity:
   1. White
   2. East Asian
   3. Indian/S/SE Asian
   4. Mixed ethnicity
   5. Other
   6. Unknown
4. What is your residence?
   1. United Kingdom
   2. Abroad
5. What is your level of education?
   1. No schooling
   2. Primary/elementary school
   3. Secondary school/High school diploma
   4. University Degree
   5. PhD
6. What is your occupational status?
   1. Worker
   2. Student
   3. Homemaker
   4. Retired
   5. Unemployed/looking for work
   6. Disabled/unapplicable/sheltered employment
   7. Unknown

***Drug use***

**December 2020**

1. If you have never taken another recreational drug, or do not want to answer these questions, please indicate below (Note your data will be analysed in anonymous format)
   1. I would like to answer questions about recreational drug use
   2. Skip this section
2. Have you taken any of the following drugs DURING the pandemic
3. Cannabis
4. Cocaine
5. MDMA/ecstasy
6. Ketamine
7. Heroin/opiates
8. LSD
9. Magic mushrooms (psilocybin)
10. Crystal N,N-DMT
11. 5-MeO-DMT
12. Ayahuasca
13. Mescaline
14. Other

**June 2021**

1. Have you taken a recreational drug before in your life
   1. Yes
   2. No
2. Would you like to answer the questions about recreational drug use
   1. I would like to answer questions about recreational drug use
   2. Skip this section
3. Have you taken any of the following drugs DURING the pandemic
   1. Cannabis
   2. Cocaine
   3. MDMA/ecstasy
   4. Ketamine
   5. Heroin/opiates
   6. LSD
   7. Magic mushrooms (psilocybin)
   8. Crystal N,N-DMT
   9. 5-MeO-DMT
   10. Ayahuasca
   11. Mescaline
   12. Other

***Mental Health***

*GAD-7 items*

1.Feeling nervous, anxious or on edge?

2. Not being able to stop or control worrying?

3.Worrying too much about different things?

4.Trouble relaxing?/// (over the last month)

5.Being so restless that it is hard to sit still?

6.Becoming easily annoyed or irritable?

7.Feeling afraid as if something awful might happen?

*PHQ items*

8.Little interest or pleasure in doing things

9.feeling down or depressed

10.feeling tired or having little energy

11.trouble concentrating on things, such as reading the newspaper or watching television

12.not being able to get to sleep or stay asleep

***Resilience***


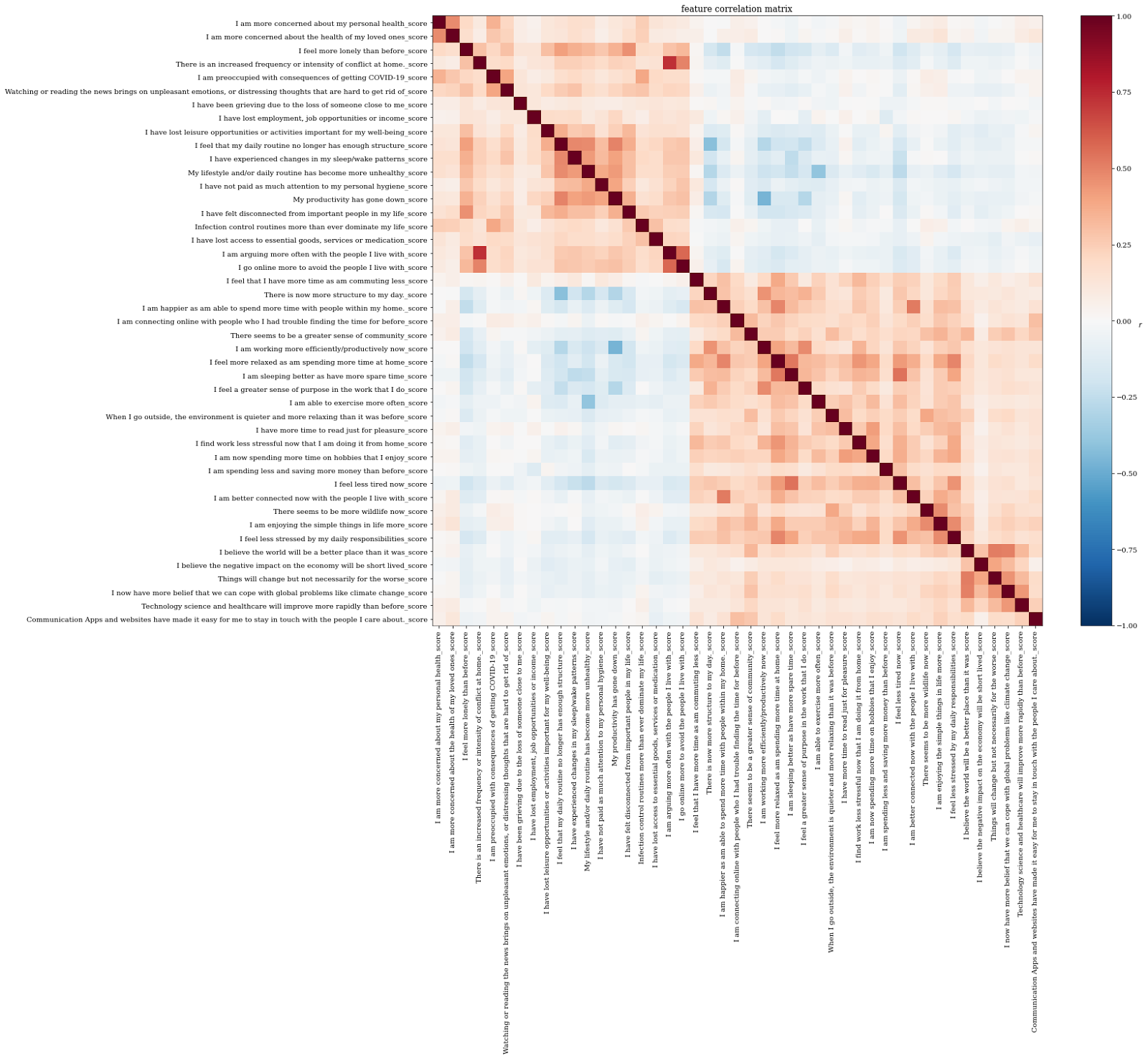


**Supp Fig 1. Feature correlations PDGIS**


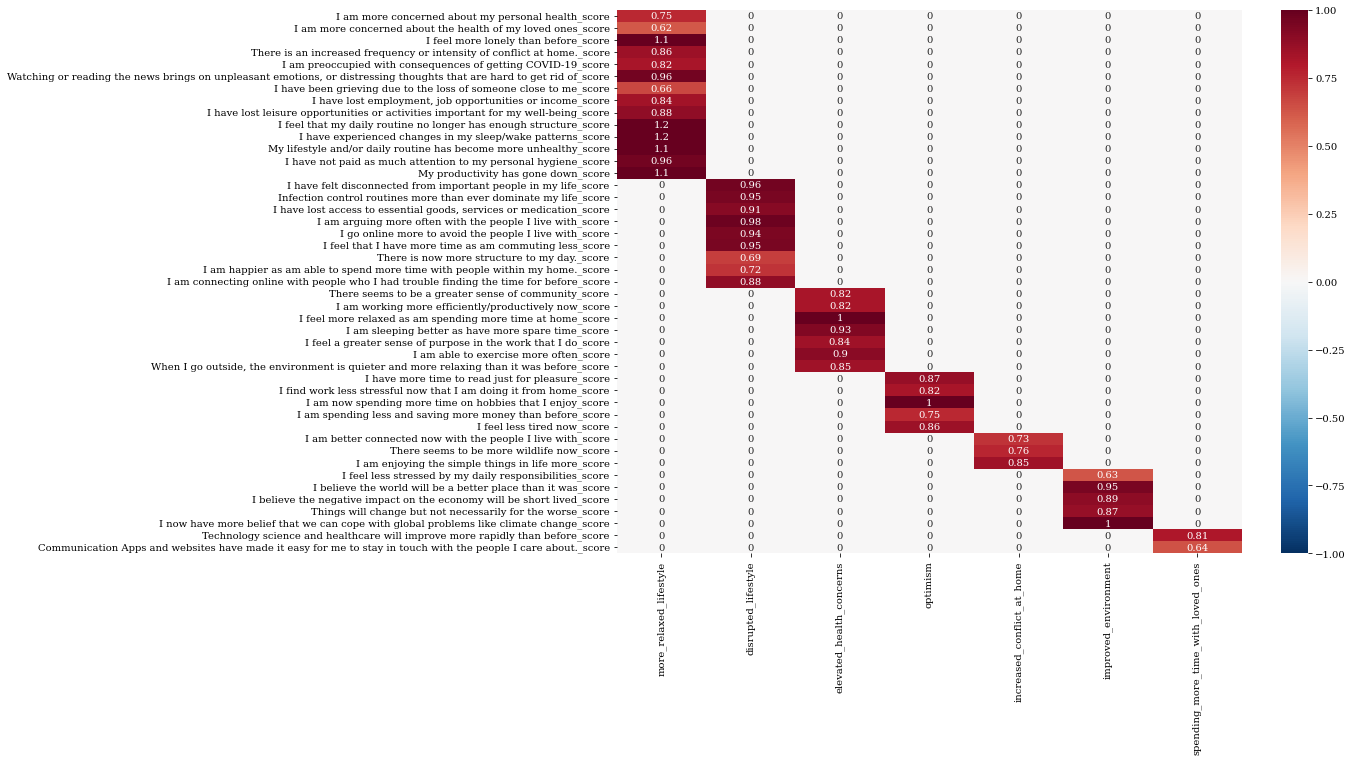


**Supp Fig 2. Factor loadings PDGIS**

*Cue- Please indicate how well the following statements describe the impact of lockdown on you*

*Negative aspects*

1. I am more concerned about my personal health
2. I am more concerned about the health of my loved ones
3. I feel more lonely than before
4. There is an increased frequency or intensity of conflict at home
5. I am preoccupied with consequences of getting COVID-19
6. Watching or reading the news brings on unpleasant emotions, or distressing thoughts that are hard to get rid of
7. I have been grieving due to the loss of someone close to me
8. I have lost employment, job opportunities or income
9. I have lost leisure opportunities or activities important for my well-being
10. I feel that my daily routine no longer has enough structure
11. I have experienced changes in my sleep/wake patterns
12. My lifestyle and/or daily routine has become more unhealthy
13. I have not paid as much attention to my personal hygiene
14. My productivity has gone down
15. I have felt disconnected from important people in my life
16. Infection control routines more than ever dominate my life
17. I have lost access to essential goods, services or medication
18. I am arguing more often with the people I live with
19. I go online more to avoid the people I live with
20. Are you drinking more alcohol since lockdown?
21. Are you smoking more cigarettes since lockdown?

*Positive aspects*

1. I feel that I have more time as am commuting less
2. There is now more structure to my day
3. I am happier as am able to spend more time with people within my home
4. I am connecting online with people who I had trouble finding the time for before
5. There is a greater sense of shared community
6. I am working more efficiently/productively now
7. I feel more relaxed as am spending more time at home
8. I am sleeping better as have more spare time
9. I feel a greater sense of purpose in the work that I do
10. I am able to exercise more often
11. When I go outside, the environment is quieter and more relaxing than it was before
12. I have more time to read just for pleasure
13. I find work less stressful now that I am doing it from home
14. I am now spending more time on hobbies that I enjoy
15. I am spending less and saving more money than before
16. I have more social contact outside of my home
17. I feel less tired now I am better connected now with the people I live with
18. There seems to be more wildlife now
19. I am enjoying the simple things in life more
20. I feel less stressed by my daily responsibilities
21. Communication Apps and websites have made it easy for me to stay in touch with the people I care about

*Cue - How will things change in the long term?*

*Optimism*

1. I believe the world will be a better place than it was
2. I believe the negative impact on the economy will be short lived
3. Things will change but not necessarily for the worse
4. I have more belief that we can cope with global problems like climate change
5. Technology science and healthcare will improve more rapidly than before

***Personality***


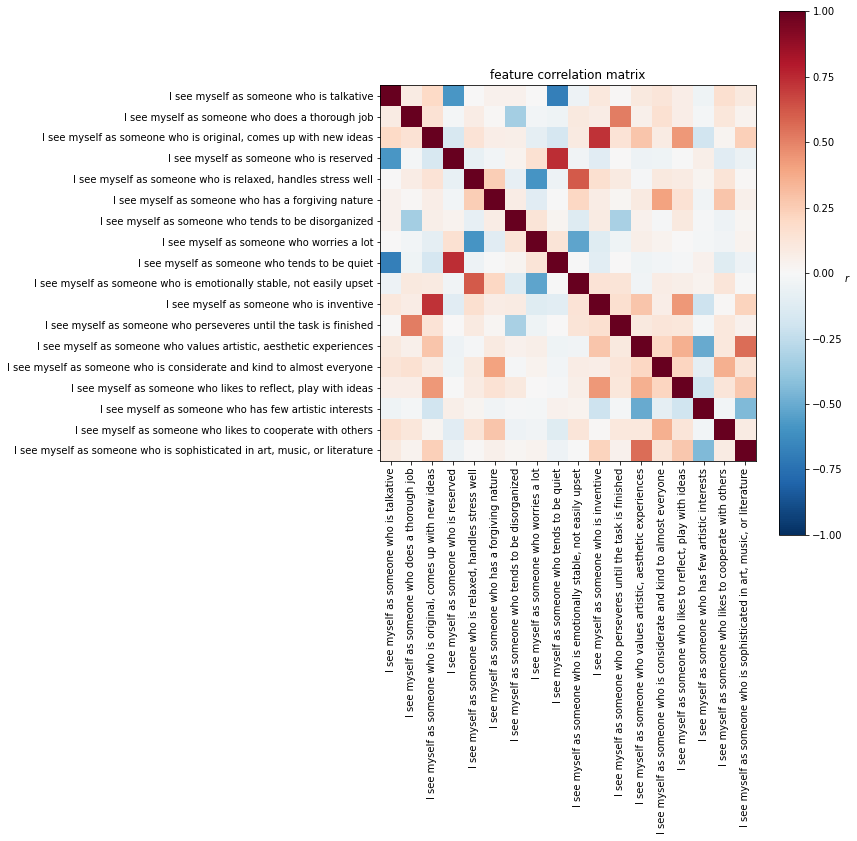


**Supp Fig 3. Feature correlations little Big5**


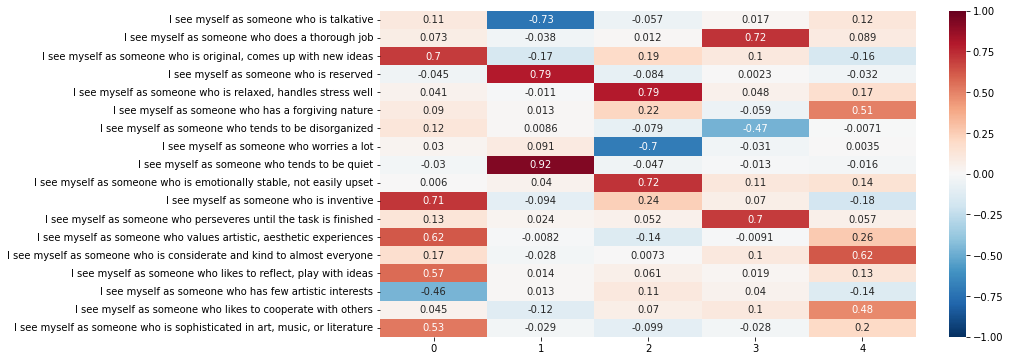


**Supp Fig 4. Factor loadings little big5.** Factors 1 and 2 have been *(-1) in the analysis to convert to extraversion and neuroticism.

Little Big 5

1. I see myself as someone who is talkative
2. I see myself as someone who does a thorough job
3. I see myself as someone who is original, comes up with new ideas,
4. I see myself as someone who is reserved
5. I see myself as someone who is relaxed, handles stress well
6. I see myself as someone who has a forgiving nature
7. I see myself as someone who tends to be disorganised
8. I see myself as someone who worries a lot
9. I see myself as someone who tends to be quiet
10. I see myself as someone who is emotionally stable, not easily upset
11. I see myself as someone who is inventive
12. I see myself as someone who perseveres until the task is finished
13. I see myself as someone who values artistic, aesthetic experiences
14. I see myself as someone who is considerate and kind to almost everyone
15. I see myself as someone who likes to reflect, play with ideas
16. I see myself as someone who has few artistic interests
17. I see myself as someone who likes to cooperate with others
18. I see myself as someone who is sophisticated in art, music, or literature

***Compulsivity***


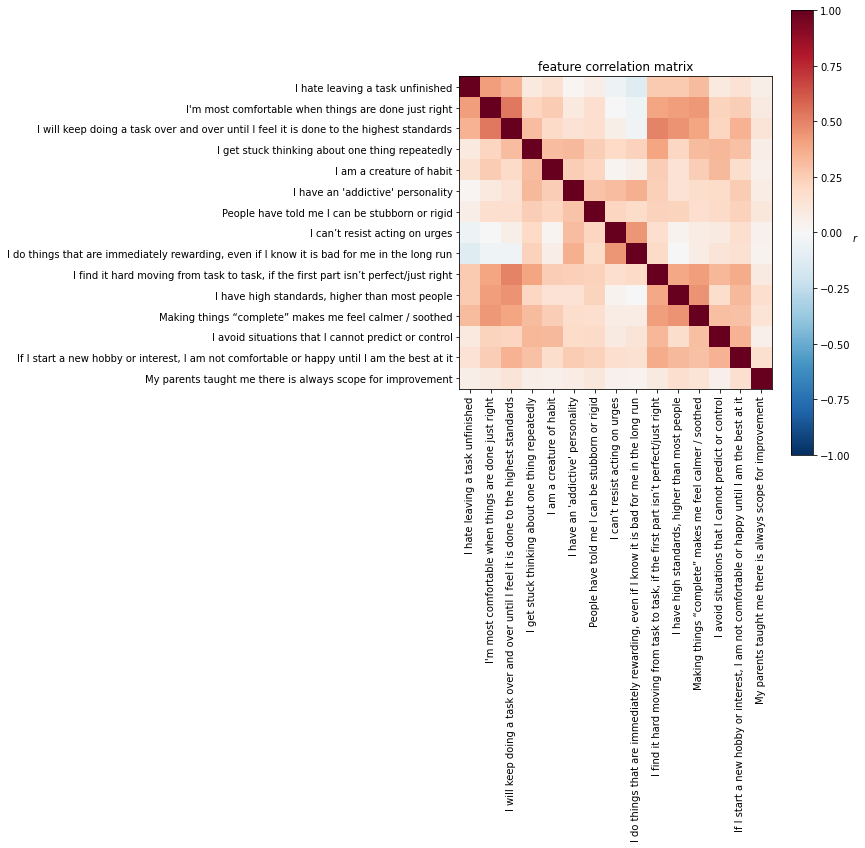


**Supp Fig 5. Feature correlations compulsivity questionnaire**


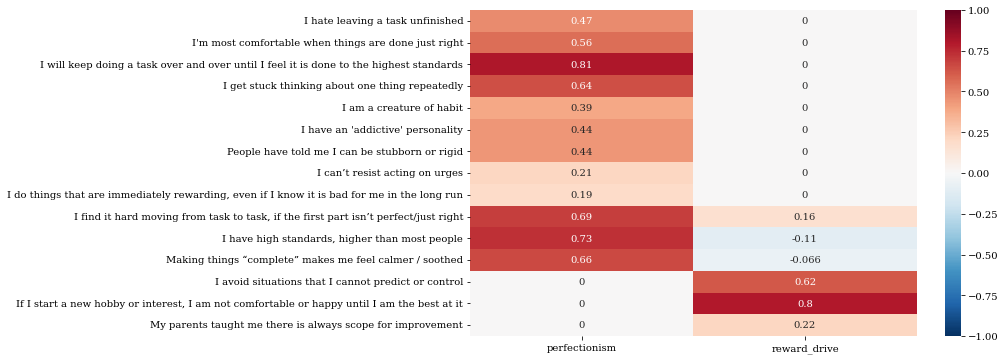


**Supp Fig 6. Factor loadings compulsivity questionnaire**

Compulsivity questionnaire

1. I hate leaving a task unfinished
2. I'm most comfortable when things are done just right
3. I will keep doing a task over and over until I feel it is done to the highest standards
4. I get stuck thinking about one thing repeatedly
5. I am a creature of habit
6. I have an 'addictive' personality
7. People have told me I can be stubborn or rigid
8. I can’t resist acting on urges
9. I do things that are immediately rewarding, even if I know it is bad for me in the long run
10. I find it hard moving from task to task, if the first part isn’t perfect/just right
11. I have high standards, higher than most people
12. Making things “complete” makes me feel calmer / soothed
13. I avoid situations that I cannot predict or control
14. If I start a new hobby or interest, I am not comfortable or happy until I am the best at it
15. My parents taught me there is always scope for improvement

**Part II. Sociodemographics of the GBIT cohort**

**
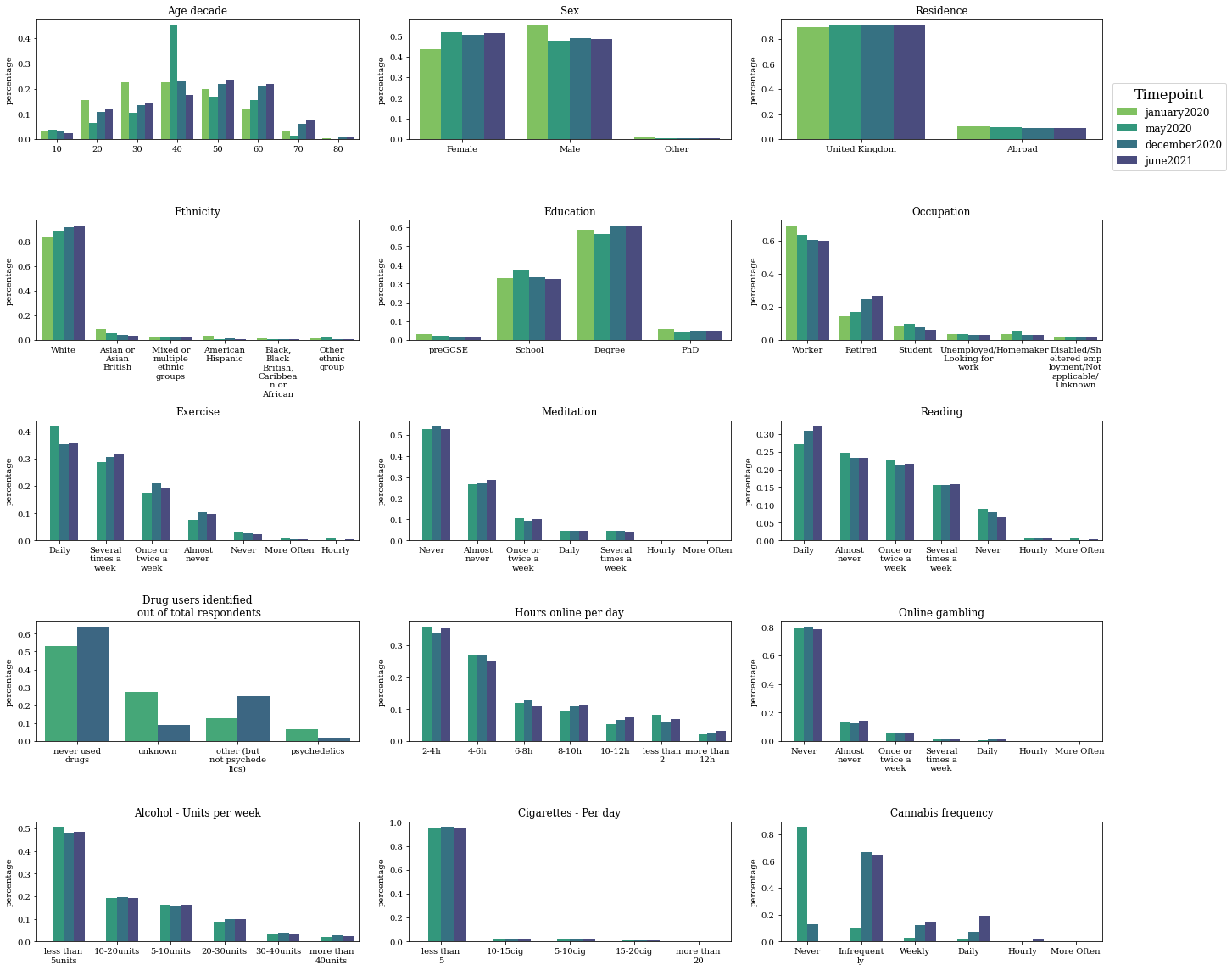
**

**Supp Figure 7. Sociodemographic characteristics of the entire cohort.**

**Part III - Sociodemographics, lifestyle and personality differences amongst clusters of drug use**

People who use different substances/combinations of different substances also vary significantly in their sociodemographic characteristics (Supp Table 1), other lifestyle choices (Supp Table 2), as well as personality (Supp Figure 7), highlighting the importance of taking these into account when evaluating associations with drug use.

For personality and compulsivity, 2-way ANOVA with cluster, personality/compulsivity dimension, and the interaction between cluster and dimension as predictors shows a significant effect of cluster on all dimensions of personality and compulsivity (F_(9,273987_=24.09,p<0.001) and a significant interaction (F_(54,273987)_=46.97,p<0.001). To investigate what drives these differences we performed Tukey post-hoc tests between all clusters for each personality and compulsivity dimension (Supp Table 3).

***Differences in demographics, lifestyle and personality between clusters***

|  |  | Age_decade | | | | | | | | Sex | | | Residence | | Ethnicity | | | | | | Education | | | | Occupation | | | | | |
| --- | --- | --- | --- | --- | --- | --- | --- | --- | --- | --- | --- | --- | --- | --- | --- | --- | --- | --- | --- | --- | --- | --- | --- | --- | --- | --- | --- | --- | --- | --- |
| timepoint | cluster | 10 | 20 | 30 | 40 | 50 | 60 | 70 | 80 | Female | Male | Other | Abroad | United Kingdom | American Hispanic | Asian or Asian British | Black, Black British, Caribbean or African | Mixed or multiple ethnic groups | Other ethnic group | White | Degree | PhD | School | preGCSE | Disabled/Sheltered employment/Not applicable/Unknown | Homemaker | Retired | Student | Unemployed/Looking for work | Worker |
| Dec-20 | Drug use history but no pandemic use | 1.4 | 12.7 | 18.3 | 28 | 21.1 | 15.7 | 2.8 | 0.1 | 37.9 | 61.5 | 0.6 | 8 | 92 | 1.2 | 3.2 | 0.7 | 2.6 | 0.3 | 91.9 | 65.7 | 5.7 | 27.1 | 1.4 | 1.5 | 1.8 | 15.7 | 5.7 | 3.4 | 71.9 |
|  | MDMA/ecstasy and cannabis users | 10.2 | 32.2 | 8.5 | 32.2 | 15.3 | 1.7 | 0 | 0 | 44.1 | 55.9 | 0 | 13.6 | 86.4 | 0 | 3.4 | 0 | 5.1 | 0 | 91.5 | 76.3 | 1.7 | 22 | 0 | 0 | 0 | 0 | 22 | 3.4 | 74.6 |
|  | Never used drugs | 3 | 10.2 | 12.3 | 19.8 | 23.1 | 23.2 | 7.4 | 1 | 52.8 | 46.6 | 0.6 | 8.1 | 91.9 | 1 | 4 | 0.4 | 2.3 | 0.3 | 92 | 59.9 | 5.4 | 33.2 | 1.5 | 1.1 | 3 | 28.8 | 6.5 | 2.5 | 58 |
|  | Unknown/Unwilling to disclose | 4 | 8.5 | 12 | 26.4 | 21 | 21.6 | 5.9 | 0.5 | 55.9 | 43.7 | 0.4 | 9.5 | 90.5 | 0.7 | 3.4 | 0.4 | 2.6 | 0.6 | 92.3 | 58.9 | 4 | 35.7 | 1.4 | 1.4 | 3.4 | 25 | 8.5 | 3 | 58.7 |
|  | Cannabis users | 7.2 | 19.6 | 20.8 | 22.8 | 16.6 | 11 | 1.8 | 0.1 | 37.7 | 61.7 | 0.6 | 14.5 | 85.5 | 2 | 3.5 | 0.5 | 5.3 | 0.5 | 88.3 | 55.3 | 4.8 | 37.8 | 2.2 | 3.2 | 2.6 | 9.8 | 14.8 | 5.6 | 63.9 |
|  | Cannabis and cocaine users | 14.1 | 30.1 | 24.5 | 24.5 | 5.5 | 0.6 | 0.6 | 0 | 33.7 | 66.3 | 0 | 4.9 | 95.1 | 1.2 | 2.5 | 0 | 6.1 | 1.2 | 89 | 62.6 | 3.7 | 33.1 | 0.6 | 1.8 | 0 | 0.6 | 26.4 | 9.2 | 62 |
|  | Users of ‘other’ drugs | 3.8 | 9.8 | 17.1 | 29.9 | 20.9 | 15.4 | 3 | 0 | 38.5 | 60.3 | 1.3 | 9 | 91 | 0.4 | 1.3 | 0.4 | 2.6 | 0.9 | 94.4 | 58.1 | 5.1 | 32.5 | 4.3 | 3 | 0 | 16.7 | 10.7 | 6 | 63.7 |
|  | Cocaine users | 5.8 | 25.7 | 21.6 | 31 | 11.7 | 4.1 | 0 | 0 | 33.3 | 66.1 | 0.6 | 8.8 | 91.2 | 1.2 | 2.3 | 0.6 | 2.9 | 0.6 | 92.4 | 68.4 | 1.2 | 29.8 | 0.6 | 1.8 | 0 | 1.8 | 14.6 | 3.5 | 78.4 |
|  | Psychedelics and cannabis users | 9.7 | 37.9 | 17.7 | 19.4 | 9.7 | 5.6 | 0 | 0 | 33.9 | 66.1 | 0 | 12.1 | 87.9 | 1.6 | 6.5 | 0 | 7.3 | 0 | 84.7 | 66.1 | 1.6 | 31.5 | 0.8 | 1.6 | 1.6 | 3.2 | 27.4 | 9.7 | 56.5 |
|  | Extreme polydrug users | 12.5 | 18.8 | 12.5 | 18.8 | 31.2 | 6.2 | 0 | 0 | 50 | 50 | 0 | 25 | 75 | 0 | 0 | 0 | 12.5 | 6.2 | 81.2 | 62.5 | 0 | 31.2 | 6.2 | 0 | 12.5 | 6.2 | 18.8 | 12.5 | 50 |
| Jun-21 | Drug use history but no pandemic use | 0.6 | 10.9 | 19.4 | 22.7 | 23.7 | 18.4 | 4.2 | 0.1 | 41.2 | 58.4 | 0.4 | 8.7 | 91.3 | 1 | 2.4 | 0.4 | 2.3 | 0.3 | 93.7 | 64.1 | 5.4 | 29.1 | 1.4 | 1.9 | 2.6 | 19.1 | 3.9 | 3.8 | 68.7 |
|  | MDMA/ecstasy and cannabis users | 22.6 | 25.8 | 22.6 | 12.9 | 12.9 | 3.2 | 0 | 0 | 32.3 | 64.5 | 3.2 | 16.1 | 83.9 | 0 | 3.2 | 0 | 12.9 | 0 | 83.9 | 58.1 | 0 | 41.9 | 0 | 0 | 0 | 3.2 | 22.6 | 6.5 | 67.7 |
|  | Never used drugs | 2.5 | 10.4 | 11.4 | 14.5 | 25.1 | 25.1 | 9.9 | 1.1 | 55.8 | 43.7 | 0.5 | 8.5 | 91.5 | 0.7 | 3.3 | 0.4 | 1.9 | 0.4 | 93.4 | 59.3 | 4.9 | 34.3 | 1.5 | 1.1 | 3.1 | 32 | 5.8 | 2.6 | 55.4 |
|  | Unknown/Unwilling to disclose | 2.1 | 14.2 | 18.4 | 24.6 | 19.6 | 17 | 3.9 | 0.1 | 52.3 | 47.1 | 0.6 | 9.2 | 90.8 | 1.1 | 2.6 | 0.3 | 2.2 | 0.5 | 93.3 | 68 | 4.5 | 26.1 | 1.4 | 1.8 | 2.8 | 17.9 | 7.4 | 2.7 | 67.4 |
|  | Cannabis users | 7.3 | 22.2 | 21.5 | 19.1 | 17.5 | 10.3 | 2 | 0.1 | 37.5 | 61.5 | 1 | 15.8 | 84.2 | 1.7 | 3.8 | 0.7 | 4.3 | 0.1 | 89.3 | 58.4 | 4.1 | 35.6 | 1.9 | 3 | 2.7 | 10 | 13.4 | 5.4 | 65.5 |
|  | Cannabis and cocaine users | 11 | 37.7 | 21.4 | 20.8 | 7.8 | 1.3 | 0 | 0 | 31.8 | 67.5 | 0.6 | 5.2 | 94.8 | 0.6 | 1.9 | 0 | 7.8 | 0 | 89.6 | 65.6 | 3.2 | 28.6 | 2.6 | 2.6 | 1.9 | 1.3 | 26.6 | 6.5 | 61 |
|  | Users of ‘other’ drugs | 7.1 | 42.9 | 35.7 | 7.1 | 7.1 | 0 | 0 | 0 | 57.1 | 42.9 | 0 | 7.1 | 92.9 | 0 | 14.3 | 0 | 0 | 0 | 85.7 | 57.1 | 7.1 | 35.7 | 0 | 7.1 | 0 | 0 | 14.3 | 7.1 | 71.4 |
|  | Cocaine users | 3.1 | 38.5 | 29.2 | 16.9 | 9.2 | 2.3 | 0.8 | 0 | 36.2 | 63.1 | 0.8 | 8.5 | 91.5 | 0.8 | 0.8 | 0.8 | 5.4 | 0.8 | 91.5 | 61.5 | 3.1 | 33.8 | 1.5 | 1.5 | 0 | 0.8 | 11.5 | 4.6 | 81.5 |
|  | Psychedelics and cannabis users | 1.1 | 38 | 20.7 | 22.8 | 12 | 5.4 | 0 | 0 | 34.8 | 65.2 | 0 | 22.8 | 77.2 | 2.2 | 4.3 | 0 | 9.8 | 2.2 | 81.5 | 67.4 | 4.3 | 27.2 | 1.1 | 5.4 | 0 | 3.3 | 15.2 | 9.8 | 66.3 |
|  | Extreme polydrug users | 16.7 | 50 | 11.1 | 16.7 | 5.6 | 0 | 0 | 0 | 22.2 | 77.8 | 0 | 5.6 | 94.4 | 0 | 5.6 | 0 | 11.1 | 0 | 83.3 | 33.3 | 5.6 | 61.1 | 0 | 0 | 11.1 | 11.1 | 11.1 | 16.7 | 50 |
| Chi2, p | | chi2=2479.6,p<0.001 | | | | | | | | chi2=702.8,p<0.001 | | | chi2=114.8,  p<0.001 | | chi2=208.6,p<0.001 | | | | | | chi2=137.2,p<0.001 | | | | chi2=1656.8,p<0.001 | | | | | |

**Supp Table 1. Demographics characteristics of different clusters of drug use at different timepoints.**

|  | | Exercise | | | | | | | Meditation | | | | | | | Reading | | | | | | | Hours online | | | | | | | Online gambling | | | | | | | Alcohol (units/week) | Cigarettes (cig/per day) |
| --- | --- | --- | --- | --- | --- | --- | --- | --- | --- | --- | --- | --- | --- | --- | --- | --- | --- | --- | --- | --- | --- | --- | --- | --- | --- | --- | --- | --- | --- | --- | --- | --- | --- | --- | --- | --- | --- | --- |
| timepoint | cluster | Almost never | Daily | Hourly | More Often | Never | Once or twice a week | Several times a week | Almost never | Daily | Hourly | More Often | Never | Once or twice a week | Several times a week | Almost never | Daily | Hourly | More Often | Never | Once or twice a week | Several times a week | 10-12h | 2-4h | 4-6h | 6-8h | 8-10h | less than 2 | more than 12h | Almost never | Daily | Hourly | More Often | Never | Once or twice a week | Several times a week |  |  |
| Dec-20 | Drug use history but no pandemic use | 11.5 | 32.3 | 0.1 | 0.3 | 2.9 | 22.5 | 30.4 | 28.9 | 4.2 | 0 | 0 | 53.5 | 9.4 | 3.9 | 24.3 | 28.9 | 0.4 | 0 | 8.5 | 21.3 | 16.6 | 7.6 | 30.3 | 28 | 14.5 | 13.1 | 4 | 2.3 | 16 | 1.4 | 0 | 0 | 73.5 | 7.1 | 2 | 12.77 ¬± 15.57 | 1.15 ¬± 4.16 |
|  | MDMA/ecstasy and cannabis users | 6.8 | 28.8 | 0 | 0 | 3.4 | 32.2 | 28.8 | 28.8 | 0 | 0 | 0 | 55.9 | 11.9 | 3.4 | 27.1 | 11.9 | 0 | 0 | 8.5 | 32.2 | 20.3 | 18.6 | 13.6 | 30.5 | 15.3 | 13.6 | 1.7 | 6.8 | 11.9 | 1.7 | 0 | 0 | 78 | 8.5 | 0 | 10.71 ¬± 8.85 | 2.34 ¬± 8.63 |
|  | Never used drugs | 9.8 | 36.2 | 0.2 | 0.3 | 2.4 | 20.4 | 30.6 | 26.3 | 4.8 | 0.1 | 0.1 | 55.2 | 9.3 | 4.3 | 22.2 | 33.1 | 0.7 | 0.2 | 7.4 | 20.6 | 15.8 | 6.3 | 36.3 | 26.3 | 12.5 | 9.6 | 7.2 | 1.9 | 10.7 | 0.7 | 0 | 0 | 82.8 | 4.7 | 1.1 | 8.66 ¬± 11.72 | 0.43 ¬± 2.80 |
|  | Unknown/Unwilling to disclose | 10.1 | 36 | 0.2 | 0.2 | 2.7 | 20.5 | 30.1 | 27 | 4.1 | 0.1 | 0 | 54.8 | 9.4 | 4.6 | 23.3 | 30.9 | 0.5 | 0.2 | 8.6 | 21.5 | 15 | 6.3 | 34.5 | 27.4 | 12.6 | 10.9 | 6 | 2.4 | 11.9 | 0.9 | 0.1 | 0 | 81 | 4.9 | 1.3 | 9.04 ¬± 11.96 | 0.67 ¬± 3.17 |
|  | Cannabis users | 12.6 | 30.9 | 0.5 | 0.2 | 3.6 | 21.1 | 31.2 | 32.3 | 3.9 | 0 | 0 | 44.6 | 12.6 | 6.6 | 29.4 | 21.7 | 0.2 | 0.2 | 9.7 | 23.8 | 15 | 8.9 | 27.2 | 27.5 | 16.7 | 13.3 | 2.3 | 4.1 | 16.7 | 1.2 | 0.2 | 0.1 | 73.1 | 6.9 | 1.7 | 12.51 ¬± 16.74 | 2.64 ¬± 5.42 |
|  | Cannabis and cocaine users | 9.8 | 30.1 | 0 | 0 | 1.8 | 20.2 | 38 | 27.6 | 3.7 | 0 | 0 | 53.4 | 11 | 4.3 | 29.4 | 11.7 | 0 | 0 | 10.4 | 30.1 | 18.4 | 7.3 | 17.2 | 32.5 | 17.9 | 17.9 | 2.6 | 4.6 | 20.2 | 2.5 | 0 | 0.6 | 69.3 | 4.3 | 3.1 | 18.73 ¬± 17.36 | 2.67 ¬± 4.93 |
|  | Users of ‘other’ drugs | 17.5 | 33.3 | 0.4 | 0.4 | 4.3 | 22.2 | 21.8 | 33.8 | 1.3 | 0 | 0 | 54.7 | 5.1 | 5.1 | 25.2 | 20.5 | 0.9 | 0 | 13.7 | 22.6 | 17.1 | 11 | 22.8 | 26 | 12.3 | 19.6 | 1.8 | 6.4 | 19.7 | 1.7 | 0 | 0 | 68.8 | 7.7 | 2.1 | 13.62 ¬± 16.37 | 1.81 ¬± 5.37 |
|  | Cocaine users | 12.9 | 29.8 | 0 | 0 | 1.2 | 24 | 32.2 | 26.3 | 4.1 | 0 | 0 | 52 | 12.9 | 4.7 | 32.7 | 18.1 | 0 | 0 | 9.4 | 25.7 | 14 | 7.5 | 19.9 | 33.5 | 18.6 | 14.9 | 2.5 | 3.1 | 26.3 | 1.8 | 0 | 0 | 57.3 | 12.3 | 2.3 | 17.19 ¬± 15.73 | 2.09 ¬± 4.91 |
|  | Psychedelics and cannabis users | 10.5 | 22.6 | 0 | 0 | 2.4 | 25.8 | 38.7 | 35.5 | 8.1 | 0 | 0 | 31.5 | 17.7 | 7.3 | 29.8 | 17.7 | 0 | 0 | 5.6 | 28.2 | 18.5 | 10.5 | 15.8 | 25.4 | 17.5 | 17.5 | 5.3 | 7.9 | 10.5 | 2.4 | 0 | 0 | 83.9 | 2.4 | 0.8 | 10.17 ¬± 11.97 | 1.00 ¬± 3.55 |
|  | Extreme polydrug users | 18.8 | 18.8 | 0 | 0 | 6.2 | 31.2 | 25 | 25 | 6.2 | 0 | 0 | 43.8 | 18.8 | 6.2 | 56.2 | 0 | 0 | 0 | 6.2 | 18.8 | 18.8 | 13.3 | 6.7 | 26.7 | 33.3 | 6.7 | 0 | 13.3 | 18.8 | 0 | 0 | 0 | 81.2 | 0 | 0 | 15.94 ¬± 13.32 | 2.25 ¬± 6.03 |
| Jun-21 | Drug use history but no pandemic use | 11.3 | 34 | 0.2 | 0.3 | 2.3 | 20.8 | 31.1 | 28.7 | 3.6 | 0 | 0 | 54.1 | 10 | 3.5 | 25.1 | 30 | 0.4 | 0.3 | 6.7 | 21.8 | 15.8 | 9.1 | 30.3 | 27.7 | 12.2 | 12.9 | 4.6 | 3.2 | 16.6 | 1.3 | 0.1 | 0 | 72.9 | 7.2 | 1.9 | 12.18 ¬± 15.11 | 1.21 ¬± 4.31 |
|  | MDMA/ecstasy and cannabis users | 9.7 | 25.8 | 0 | 0 | 3.2 | 32.3 | 29 | 35.5 | 6.5 | 0 | 0 | 41.9 | 16.1 | 0 | 35.5 | 29 | 0 | 0 | 9.7 | 22.6 | 3.2 | 6.7 | 30 | 33.3 | 16.7 | 10 | 0 | 3.3 | 19.4 | 0 | 0 | 0 | 71 | 9.7 | 0 | 12.74 ¬± 12.84 | 1.55 ¬± 4.30 |
|  | Never used drugs | 9.1 | 37.1 | 0.3 | 0.4 | 2.4 | 18.9 | 31.7 | 27.9 | 4.5 | 0 | 0 | 53.9 | 9.6 | 3.9 | 22 | 34.3 | 0.5 | 0.3 | 6.1 | 21 | 15.9 | 6.6 | 38.9 | 23.8 | 9.9 | 9.9 | 8.2 | 2.7 | 12.2 | 0.7 | 0 | 0 | 81.9 | 4.3 | 0.9 | 7.91 ¬± 11.24 | 0.39 ¬± 2.56 |
|  | Unknown/Unwilling to disclose | 8.1 | 36.6 | 0.3 | 0.2 | 1.7 | 18.2 | 34.9 | 29.5 | 5.4 | 0.1 | 0 | 48.7 | 10.7 | 5.7 | 24.2 | 32.2 | 0.4 | 0.1 | 6.7 | 21.2 | 15.2 | 8.6 | 31.6 | 25.4 | 13.5 | 11.7 | 6.1 | 3.2 | 15.9 | 0.4 | 0.1 | 0 | 76.1 | 6.3 | 1.1 | 9.85 ¬± 12.03 | 0.87 ¬± 3.48 |
|  | Cannabis users | 12.8 | 30.6 | 0.3 | 1 | 3.3 | 19.2 | 32.8 | 32.5 | 3.7 | 0.3 | 0.3 | 43.9 | 12.8 | 6.6 | 27.6 | 21.1 | 0.4 | 0.1 | 9.8 | 24.1 | 16.8 | 9.4 | 23.9 | 26.5 | 14.5 | 16.5 | 3.3 | 5.9 | 18.8 | 1 | 0 | 0 | 71.5 | 7 | 1.7 | 12.27 ¬± 16.55 | 2.57 ¬± 5.38 |
|  | Cannabis and cocaine users | 11.7 | 24.7 | 0 | 1.3 | 1.9 | 22.1 | 38.3 | 33.8 | 0.6 | 0 | 0 | 44.8 | 15.6 | 5.2 | 26.6 | 14.9 | 0 | 0 | 8.4 | 37.7 | 12.3 | 9.4 | 14.8 | 28.2 | 16.8 | 21.5 | 3.4 | 6 | 27.3 | 1.3 | 0 | 0.6 | 59.1 | 9.1 | 2.6 | 19.99 ¬± 19.99 | 2.61 ¬± 5.19 |
|  | Users of ‘other’ drugs | 14.3 | 35.7 | 0 | 0 | 0 | 14.3 | 35.7 | 42.9 | 7.1 | 0 | 0 | 42.9 | 7.1 | 0 | 21.4 | 14.3 | 0 | 0 | 7.1 | 50 | 7.1 | 21.4 | 7.1 | 21.4 | 14.3 | 21.4 | 7.1 | 7.1 | 28.6 | 0 | 0 | 0 | 71.4 | 0 | 0 | 12.71 ¬± 10.22 | 2.71 ¬± 6.39 |
|  | Cocaine users | 10.8 | 23.1 | 0 | 0.8 | 1.5 | 24.6 | 39.2 | 36.9 | 3.8 | 0 | 0 | 46.2 | 8.5 | 4.6 | 32.3 | 17.7 | 0 | 0 | 12.3 | 25.4 | 12.3 | 11.9 | 19.8 | 27.8 | 16.7 | 18.3 | 2.4 | 3.2 | 18.5 | 2.3 | 0 | 0 | 63.1 | 13.1 | 3.1 | 13.24 ¬± 14.24 | 1.52 ¬± 4.08 |
|  | Psychedelics and cannabis users | 9.8 | 27.2 | 1.1 | 0 | 1.1 | 22.8 | 38 | 33.7 | 5.4 | 0 | 0 | 37 | 18.5 | 5.4 | 23.9 | 23.9 | 0 | 0 | 4.3 | 23.9 | 23.9 | 15.9 | 18.2 | 23.9 | 11.4 | 19.3 | 5.7 | 5.7 | 17.4 | 0 | 0 | 0 | 75 | 4.3 | 3.3 | 14.80 ¬± 15.27 | 2.07 ¬± 5.42 |
|  | Extreme polydrug users | 22.2 | 38.9 | 5.6 | 0 | 0 | 16.7 | 16.7 | 27.8 | 11.1 | 0 | 0 | 38.9 | 22.2 | 0 | 16.7 | 22.2 | 0 | 0 | 5.6 | 33.3 | 22.2 | 16.7 | 11.1 | 55.6 | 0 | 5.6 | 0 | 11.1 | 27.8 | 0 | 0 | 0 | 61.1 | 11.1 | 0 | 15.56 ¬± 12.15 | 6.56 ¬± 12.31 |
| Chi2, p | | chi2=170.6, p<0.001 | | | | | | | chi2=189.5, p<0.001 | | | | | | | chi2=343, p<0.001 | | | | | | | chi2=725, p<0.001 | | | | | | | chi2=536.9, p<0.001 | | | | | | |  |  |

**Supp Table 2. Lifestyle** **characteristics of different clusters of drug use at different timepoints.**

Differences in cigarettes per day across clusters&timepoints:


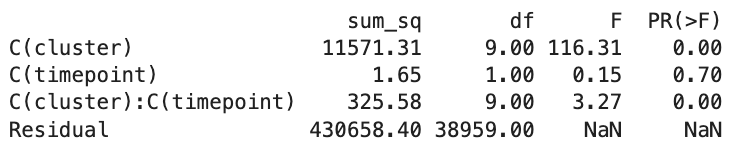


Differences in alcohol units per week across clusters&timepoints:


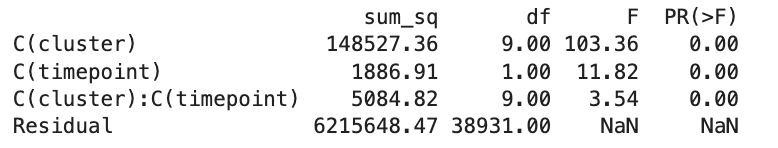


**

**

**Supp Fig 8. Personality and compulsivity differences between clusters.** Personality factors have been derived from the mini-Big5 and the compulsivity questionnaire (Tiego et al., 2023) using a factor analysis with varimax rotation. The five personality factors emerging from the mini-Big5 questionnaire are openness, extraversion, neuroticism, conscientiousness and agreeableness. The two compulsivity factors emerging from the compulsivity questionnaire are perfectionism and reward drive.  Residuals resulting from linear regressions where all personality and compulsivity factors have been adjusted to timepoint of collection (but not other variables) are plotted.

| group1 | group2 | meandiff | p | lower | upper | sig | variable | sig_corrected |
| --- | --- | --- | --- | --- | --- | --- | --- | --- |
| Cluster 1 | Cluster 10 | 0.0777 | 0.9993 | -0.2558 | 0.4112 | FALSE | openness_adj_z | FALSE |
| Cluster 1 | Cluster 2 | -0.2395 | 0 | -0.2846 | -0.1943 | TRUE | openness_adj_z | FALSE |
| Cluster 1 | Cluster 3 | -0.1453 | 0 | -0.1992 | -0.0915 | TRUE | openness_adj_z | FALSE |
| Cluster 1 | Cluster 4 | 0.1534 | 0 | 0.0638 | 0.2429 | TRUE | openness_adj_z | FALSE |
| Cluster 1 | Cluster 5 | 0.1144 | 0.5989 | -0.0665 | 0.2953 | FALSE | openness_adj_z | FALSE |
| Cluster 1 | Cluster 6 | 0.0407 | 0.9998 | -0.1628 | 0.2441 | FALSE | openness_adj_z | FALSE |
| Cluster 1 | Cluster 7 | -0.0652 | 0.9836 | -0.2506 | 0.1203 | FALSE | openness_adj_z | FALSE |
| Cluster 1 | Cluster 8 | 0.3895 | 0 | 0.172 | 0.6069 | TRUE | openness_adj_z | FALSE |
| Cluster 1 | Cluster 9 | 0.37 | 0.4797 | -0.1702 | 0.9102 | FALSE | openness_adj_z | FALSE |
| Cluster 10 | Cluster 2 | -0.3172 | 0.075 | -0.6489 | 0.0146 | FALSE | openness_adj_z | FALSE |
| Cluster 10 | Cluster 3 | -0.223 | 0.514 | -0.5561 | 0.11 | FALSE | openness_adj_z | FALSE |
| Cluster 10 | Cluster 4 | 0.0757 | 0.9995 | -0.265 | 0.4163 | FALSE | openness_adj_z | FALSE |
| Cluster 10 | Cluster 5 | 0.0367 | 1 | -0.3385 | 0.4118 | FALSE | openness_adj_z | FALSE |
| Cluster 10 | Cluster 6 | -0.037 | 1 | -0.4236 | 0.3495 | FALSE | openness_adj_z | FALSE |
| Cluster 10 | Cluster 7 | -0.1429 | 0.9729 | -0.5202 | 0.2345 | FALSE | openness_adj_z | FALSE |
| Cluster 10 | Cluster 8 | 0.3118 | 0.2667 | -0.0823 | 0.7059 | FALSE | openness_adj_z | FALSE |
| Cluster 10 | Cluster 9 | 0.2923 | 0.9068 | -0.34 | 0.9246 | FALSE | openness_adj_z | FALSE |
| Cluster 2 | Cluster 3 | 0.0941 | 0 | 0.0525 | 0.1358 | TRUE | openness_adj_z | FALSE |
| Cluster 2 | Cluster 4 | 0.3928 | 0 | 0.31 | 0.4756 | TRUE | openness_adj_z | FALSE |
| Cluster 2 | Cluster 5 | 0.3538 | 0 | 0.1762 | 0.5315 | TRUE | openness_adj_z | FALSE |
| Cluster 2 | Cluster 6 | 0.2801 | 0.0004 | 0.0796 | 0.4807 | TRUE | openness_adj_z | FALSE |
| Cluster 2 | Cluster 7 | 0.1743 | 0.0748 | -0.008 | 0.3565 | FALSE | openness_adj_z | FALSE |
| Cluster 2 | Cluster 8 | 0.6289 | 0 | 0.4142 | 0.8437 | TRUE | openness_adj_z | FALSE |
| Cluster 2 | Cluster 9 | 0.6095 | 0.0129 | 0.0704 | 1.1486 | TRUE | openness_adj_z | FALSE |
| Cluster 3 | Cluster 4 | 0.2987 | 0 | 0.2109 | 0.3865 | TRUE | openness_adj_z | FALSE |
| Cluster 3 | Cluster 5 | 0.2597 | 0.0002 | 0.0796 | 0.4398 | TRUE | openness_adj_z | FALSE |
| Cluster 3 | Cluster 6 | 0.186 | 0.1045 | -0.0167 | 0.3887 | FALSE | openness_adj_z | FALSE |
| Cluster 3 | Cluster 7 | 0.0802 | 0.9353 | -0.1044 | 0.2648 | FALSE | openness_adj_z | FALSE |
| Cluster 3 | Cluster 8 | 0.5348 | 0 | 0.3181 | 0.7516 | TRUE | openness_adj_z | FALSE |
| Cluster 3 | Cluster 9 | 0.5154 | 0.076 | -0.0246 | 1.0553 | FALSE | openness_adj_z | FALSE |
| Cluster 4 | Cluster 5 | -0.039 | 0.9998 | -0.2327 | 0.1548 | FALSE | openness_adj_z | FALSE |
| Cluster 4 | Cluster 6 | -0.1127 | 0.8186 | -0.3276 | 0.1023 | FALSE | openness_adj_z | FALSE |
| Cluster 4 | Cluster 7 | -0.2185 | 0.0173 | -0.4165 | -0.0205 | TRUE | openness_adj_z | FALSE |
| Cluster 4 | Cluster 8 | 0.2361 | 0.0357 | 0.0079 | 0.4644 | TRUE | openness_adj_z | FALSE |
| Cluster 4 | Cluster 9 | 0.2167 | 0.9625 | -0.328 | 0.7613 | FALSE | openness_adj_z | FALSE |
| Cluster 5 | Cluster 6 | -0.0737 | 0.9972 | -0.34 | 0.1926 | FALSE | openness_adj_z | FALSE |
| Cluster 5 | Cluster 7 | -0.1795 | 0.4243 | -0.4323 | 0.0733 | FALSE | openness_adj_z | FALSE |
| Cluster 5 | Cluster 8 | 0.2751 | 0.0536 | -0.002 | 0.5522 | FALSE | openness_adj_z | FALSE |
| Cluster 5 | Cluster 9 | 0.2556 | 0.9191 | -0.3112 | 0.8225 | FALSE | openness_adj_z | FALSE |
| Cluster 6 | Cluster 7 | -0.1058 | 0.9654 | -0.3752 | 0.1635 | FALSE | openness_adj_z | FALSE |
| Cluster 6 | Cluster 8 | 0.3488 | 0.0062 | 0.0565 | 0.6411 | TRUE | openness_adj_z | FALSE |
| Cluster 6 | Cluster 9 | 0.3293 | 0.7266 | -0.2451 | 0.9038 | FALSE | openness_adj_z | FALSE |
| Cluster 7 | Cluster 8 | 0.4546 | 0 | 0.1745 | 0.7347 | TRUE | openness_adj_z | FALSE |
| Cluster 7 | Cluster 9 | 0.4352 | 0.3121 | -0.1331 | 1.0035 | FALSE | openness_adj_z | FALSE |
| Cluster 8 | Cluster 9 | -0.0195 | 1 | -0.599 | 0.5601 | FALSE | openness_adj_z | FALSE |
| Cluster 1 | Cluster 10 | -0.0163 | 1 | -0.3512 | 0.3187 | FALSE | extraversion_adj_z | FALSE |
| Cluster 1 | Cluster 2 | -0.1107 | 0 | -0.156 | -0.0654 | TRUE | extraversion_adj_z | FALSE |
| Cluster 1 | Cluster 3 | -0.0616 | 0.0117 | -0.1157 | -0.0075 | TRUE | extraversion_adj_z | FALSE |
| Cluster 1 | Cluster 4 | 0.1252 | 0.0004 | 0.0353 | 0.2152 | TRUE | extraversion_adj_z | FALSE |
| Cluster 1 | Cluster 5 | 0.3554 | 0 | 0.1737 | 0.5371 | TRUE | extraversion_adj_z | FALSE |
| Cluster 1 | Cluster 6 | 0.0583 | 0.9964 | -0.146 | 0.2626 | FALSE | extraversion_adj_z | FALSE |
| Cluster 1 | Cluster 7 | 0.2892 | 0 | 0.103 | 0.4754 | TRUE | extraversion_adj_z | FALSE |
| Cluster 1 | Cluster 8 | 0.1293 | 0.6868 | -0.089 | 0.3477 | FALSE | extraversion_adj_z | FALSE |
| Cluster 1 | Cluster 9 | 0.2366 | 0.9336 | -0.3059 | 0.7791 | FALSE | extraversion_adj_z | FALSE |
| Cluster 10 | Cluster 2 | -0.0944 | 0.9966 | -0.4276 | 0.2388 | FALSE | extraversion_adj_z | FALSE |
| Cluster 10 | Cluster 3 | -0.0453 | 1 | -0.3798 | 0.2892 | FALSE | extraversion_adj_z | FALSE |
| Cluster 10 | Cluster 4 | 0.1415 | 0.952 | -0.2006 | 0.4836 | FALSE | extraversion_adj_z | FALSE |
| Cluster 10 | Cluster 5 | 0.3717 | 0.0569 | -0.0051 | 0.7484 | FALSE | extraversion_adj_z | FALSE |
| Cluster 10 | Cluster 6 | 0.0746 | 0.9999 | -0.3136 | 0.4628 | FALSE | extraversion_adj_z | FALSE |
| Cluster 10 | Cluster 7 | 0.3055 | 0.242 | -0.0735 | 0.6845 | FALSE | extraversion_adj_z | FALSE |
| Cluster 10 | Cluster 8 | 0.1456 | 0.9776 | -0.2502 | 0.5414 | FALSE | extraversion_adj_z | FALSE |
| Cluster 10 | Cluster 9 | 0.2529 | 0.9623 | -0.3822 | 0.8879 | FALSE | extraversion_adj_z | FALSE |
| Cluster 2 | Cluster 3 | 0.0491 | 0.0078 | 0.0073 | 0.0909 | TRUE | extraversion_adj_z | FALSE |
| Cluster 2 | Cluster 4 | 0.2359 | 0 | 0.1528 | 0.3191 | TRUE | extraversion_adj_z | FALSE |
| Cluster 2 | Cluster 5 | 0.4661 | 0 | 0.2877 | 0.6445 | TRUE | extraversion_adj_z | FALSE |
| Cluster 2 | Cluster 6 | 0.169 | 0.1925 | -0.0324 | 0.3704 | FALSE | extraversion_adj_z | FALSE |
| Cluster 2 | Cluster 7 | 0.3999 | 0 | 0.2169 | 0.5829 | TRUE | extraversion_adj_z | FALSE |
| Cluster 2 | Cluster 8 | 0.24 | 0.0156 | 0.0244 | 0.4557 | TRUE | extraversion_adj_z | FALSE |
| Cluster 2 | Cluster 9 | 0.3473 | 0.578 | -0.1941 | 0.8887 | FALSE | extraversion_adj_z | FALSE |
| Cluster 3 | Cluster 4 | 0.1868 | 0 | 0.0986 | 0.275 | TRUE | extraversion_adj_z | FALSE |
| Cluster 3 | Cluster 5 | 0.417 | 0 | 0.2362 | 0.5978 | TRUE | extraversion_adj_z | FALSE |
| Cluster 3 | Cluster 6 | 0.1199 | 0.6938 | -0.0837 | 0.3235 | FALSE | extraversion_adj_z | FALSE |
| Cluster 3 | Cluster 7 | 0.3508 | 0 | 0.1654 | 0.5362 | TRUE | extraversion_adj_z | FALSE |
| Cluster 3 | Cluster 8 | 0.1909 | 0.1448 | -0.0267 | 0.4086 | FALSE | extraversion_adj_z | FALSE |
| Cluster 3 | Cluster 9 | 0.2982 | 0.7726 | -0.244 | 0.8404 | FALSE | extraversion_adj_z | FALSE |
| Cluster 4 | Cluster 5 | 0.2302 | 0.007 | 0.0356 | 0.4247 | TRUE | extraversion_adj_z | FALSE |
| Cluster 4 | Cluster 6 | -0.0669 | 0.9933 | -0.2828 | 0.1489 | FALSE | extraversion_adj_z | FALSE |
| Cluster 4 | Cluster 7 | 0.164 | 0.2131 | -0.0349 | 0.3628 | FALSE | extraversion_adj_z | FALSE |
| Cluster 4 | Cluster 8 | 0.0041 | 1 | -0.2251 | 0.2333 | FALSE | extraversion_adj_z | FALSE |
| Cluster 4 | Cluster 9 | 0.1114 | 0.9998 | -0.4356 | 0.6583 | FALSE | extraversion_adj_z | FALSE |
| Cluster 5 | Cluster 6 | -0.2971 | 0.016 | -0.5645 | -0.0296 | TRUE | extraversion_adj_z | FALSE |
| Cluster 5 | Cluster 7 | -0.0662 | 0.9982 | -0.3201 | 0.1877 | FALSE | extraversion_adj_z | FALSE |
| Cluster 5 | Cluster 8 | -0.226 | 0.2322 | -0.5044 | 0.0523 | FALSE | extraversion_adj_z | FALSE |
| Cluster 5 | Cluster 9 | -0.1188 | 0.9997 | -0.6881 | 0.4505 | FALSE | extraversion_adj_z | FALSE |
| Cluster 6 | Cluster 7 | 0.2309 | 0.1734 | -0.0396 | 0.5014 | FALSE | extraversion_adj_z | FALSE |
| Cluster 6 | Cluster 8 | 0.071 | 0.999 | -0.2226 | 0.3646 | FALSE | extraversion_adj_z | FALSE |
| Cluster 6 | Cluster 9 | 0.1783 | 0.9935 | -0.3986 | 0.7552 | FALSE | extraversion_adj_z | FALSE |
| Cluster 7 | Cluster 8 | -0.1598 | 0.7369 | -0.4412 | 0.1215 | FALSE | extraversion_adj_z | FALSE |
| Cluster 7 | Cluster 9 | -0.0526 | 1 | -0.6234 | 0.5182 | FALSE | extraversion_adj_z | FALSE |
| Cluster 8 | Cluster 9 | 0.1073 | 0.9999 | -0.4748 | 0.6893 | FALSE | extraversion_adj_z | FALSE |
| Cluster 1 | Cluster 10 | 0.0842 | 0.9987 | -0.2516 | 0.42 | FALSE | neuroticism_adj_z | FALSE |
| Cluster 1 | Cluster 2 | 0.0035 | 1 | -0.042 | 0.0489 | FALSE | neuroticism_adj_z | FALSE |
| Cluster 1 | Cluster 3 | 0.0759 | 0.0004 | 0.0217 | 0.1301 | TRUE | neuroticism_adj_z | FALSE |
| Cluster 1 | Cluster 4 | 0.054 | 0.6713 | -0.0361 | 0.1442 | FALSE | neuroticism_adj_z | FALSE |
| Cluster 1 | Cluster 5 | 0.0839 | 0.9086 | -0.0982 | 0.2661 | FALSE | neuroticism_adj_z | FALSE |
| Cluster 1 | Cluster 6 | 0.1519 | 0.3587 | -0.0529 | 0.3567 | FALSE | neuroticism_adj_z | FALSE |
| Cluster 1 | Cluster 7 | 0.0768 | 0.9535 | -0.1099 | 0.2635 | FALSE | neuroticism_adj_z | FALSE |
| Cluster 1 | Cluster 8 | 0.0135 | 1 | -0.2055 | 0.2324 | FALSE | neuroticism_adj_z | FALSE |
| Cluster 1 | Cluster 9 | 0.1027 | 0.9999 | -0.4412 | 0.6466 | FALSE | neuroticism_adj_z | FALSE |
| Cluster 10 | Cluster 2 | -0.0807 | 0.999 | -0.4148 | 0.2533 | FALSE | neuroticism_adj_z | FALSE |
| Cluster 10 | Cluster 3 | -0.0083 | 1 | -0.3436 | 0.327 | FALSE | neuroticism_adj_z | FALSE |
| Cluster 10 | Cluster 4 | -0.0302 | 1 | -0.3731 | 0.3128 | FALSE | neuroticism_adj_z | FALSE |
| Cluster 10 | Cluster 5 | -0.0003 | 1 | -0.378 | 0.3774 | FALSE | neuroticism_adj_z | FALSE |
| Cluster 10 | Cluster 6 | 0.0677 | 0.9999 | -0.3215 | 0.4569 | FALSE | neuroticism_adj_z | FALSE |
| Cluster 10 | Cluster 7 | -0.0074 | 1 | -0.3873 | 0.3726 | FALSE | neuroticism_adj_z | FALSE |
| Cluster 10 | Cluster 8 | -0.0707 | 0.9999 | -0.4675 | 0.326 | FALSE | neuroticism_adj_z | FALSE |
| Cluster 10 | Cluster 9 | 0.0185 | 1 | -0.6181 | 0.6552 | FALSE | neuroticism_adj_z | FALSE |
| Cluster 2 | Cluster 3 | 0.0724 | 0 | 0.0305 | 0.1144 | TRUE | neuroticism_adj_z | FALSE |
| Cluster 2 | Cluster 4 | 0.0506 | 0.6554 | -0.0328 | 0.1339 | FALSE | neuroticism_adj_z | FALSE |
| Cluster 2 | Cluster 5 | 0.0804 | 0.9205 | -0.0984 | 0.2593 | FALSE | neuroticism_adj_z | FALSE |
| Cluster 2 | Cluster 6 | 0.1484 | 0.372 | -0.0535 | 0.3504 | FALSE | neuroticism_adj_z | FALSE |
| Cluster 2 | Cluster 7 | 0.0733 | 0.9614 | -0.1102 | 0.2568 | FALSE | neuroticism_adj_z | FALSE |
| Cluster 2 | Cluster 8 | 0.01 | 1 | -0.2062 | 0.2262 | FALSE | neuroticism_adj_z | FALSE |
| Cluster 2 | Cluster 9 | 0.0992 | 0.9999 | -0.4435 | 0.642 | FALSE | neuroticism_adj_z | FALSE |
| Cluster 3 | Cluster 4 | -0.0219 | 0.9988 | -0.1103 | 0.0666 | FALSE | neuroticism_adj_z | FALSE |
| Cluster 3 | Cluster 5 | 0.008 | 1 | -0.1733 | 0.1893 | FALSE | neuroticism_adj_z | FALSE |
| Cluster 3 | Cluster 6 | 0.076 | 0.9757 | -0.1281 | 0.2801 | FALSE | neuroticism_adj_z | FALSE |
| Cluster 3 | Cluster 7 | 0.0009 | 1 | -0.185 | 0.1868 | FALSE | neuroticism_adj_z | FALSE |
| Cluster 3 | Cluster 8 | -0.0624 | 0.9963 | -0.2807 | 0.1558 | FALSE | neuroticism_adj_z | FALSE |
| Cluster 3 | Cluster 9 | 0.0268 | 1 | -0.5168 | 0.5704 | FALSE | neuroticism_adj_z | FALSE |
| Cluster 4 | Cluster 5 | 0.0299 | 1 | -0.1652 | 0.2249 | FALSE | neuroticism_adj_z | FALSE |
| Cluster 4 | Cluster 6 | 0.0979 | 0.9178 | -0.1185 | 0.3143 | FALSE | neuroticism_adj_z | FALSE |
| Cluster 4 | Cluster 7 | 0.0228 | 1 | -0.1766 | 0.2221 | FALSE | neuroticism_adj_z | FALSE |
| Cluster 4 | Cluster 8 | -0.0406 | 0.9999 | -0.2704 | 0.1892 | FALSE | neuroticism_adj_z | FALSE |
| Cluster 4 | Cluster 9 | 0.0487 | 1 | -0.4997 | 0.597 | FALSE | neuroticism_adj_z | FALSE |
| Cluster 5 | Cluster 6 | 0.068 | 0.9986 | -0.2001 | 0.3361 | FALSE | neuroticism_adj_z | FALSE |
| Cluster 5 | Cluster 7 | -0.0071 | 1 | -0.2616 | 0.2474 | FALSE | neuroticism_adj_z | FALSE |
| Cluster 5 | Cluster 8 | -0.0705 | 0.9986 | -0.3495 | 0.2086 | FALSE | neuroticism_adj_z | FALSE |
| Cluster 5 | Cluster 9 | 0.0188 | 1 | -0.5519 | 0.5895 | FALSE | neuroticism_adj_z | FALSE |
| Cluster 6 | Cluster 7 | -0.0751 | 0.9972 | -0.3463 | 0.1961 | FALSE | neuroticism_adj_z | FALSE |
| Cluster 6 | Cluster 8 | -0.1385 | 0.8972 | -0.4328 | 0.1559 | FALSE | neuroticism_adj_z | FALSE |
| Cluster 6 | Cluster 9 | -0.0492 | 1 | -0.6276 | 0.5292 | FALSE | neuroticism_adj_z | FALSE |
| Cluster 7 | Cluster 8 | -0.0634 | 0.9995 | -0.3454 | 0.2187 | FALSE | neuroticism_adj_z | FALSE |
| Cluster 7 | Cluster 9 | 0.0259 | 1 | -0.5463 | 0.5981 | FALSE | neuroticism_adj_z | FALSE |
| Cluster 8 | Cluster 9 | 0.0893 | 1 | -0.4942 | 0.6728 | FALSE | neuroticism_adj_z | FALSE |
| Cluster 1 | Cluster 10 | -0.2786 | 0.1975 | -0.612 | 0.0548 | FALSE | conscienciousness_adj_z | FALSE |
| Cluster 1 | Cluster 2 | 0.2171 | 0 | 0.172 | 0.2622 | TRUE | conscienciousness_adj_z | FALSE |
| Cluster 1 | Cluster 3 | 0.09 | 0 | 0.0362 | 0.1439 | TRUE | conscienciousness_adj_z | FALSE |
| Cluster 1 | Cluster 4 | -0.1208 | 0.0008 | -0.2103 | -0.0313 | TRUE | conscienciousness_adj_z | FALSE |
| Cluster 1 | Cluster 5 | -0.3008 | 0 | -0.4817 | -0.12 | TRUE | conscienciousness_adj_z | FALSE |
| Cluster 1 | Cluster 6 | -0.2401 | 0.0072 | -0.4435 | -0.0367 | TRUE | conscienciousness_adj_z | FALSE |
| Cluster 1 | Cluster 7 | -0.1356 | 0.3797 | -0.3209 | 0.0498 | FALSE | conscienciousness_adj_z | FALSE |
| Cluster 1 | Cluster 8 | -0.4027 | 0 | -0.6201 | -0.1853 | TRUE | conscienciousness_adj_z | FALSE |
| Cluster 1 | Cluster 9 | -0.6327 | 0.008 | -1.1727 | -0.0927 | TRUE | conscienciousness_adj_z | FALSE |
| Cluster 10 | Cluster 2 | 0.4957 | 0.0001 | 0.164 | 0.8273 | TRUE | conscienciousness_adj_z | FALSE |
| Cluster 10 | Cluster 3 | 0.3686 | 0.0167 | 0.0357 | 0.7016 | TRUE | conscienciousness_adj_z | FALSE |
| Cluster 10 | Cluster 4 | 0.1578 | 0.9055 | -0.1827 | 0.4984 | FALSE | conscienciousness_adj_z | FALSE |
| Cluster 10 | Cluster 5 | -0.0223 | 1 | -0.3973 | 0.3528 | FALSE | conscienciousness_adj_z | FALSE |
| Cluster 10 | Cluster 6 | 0.0385 | 1 | -0.348 | 0.4249 | FALSE | conscienciousness_adj_z | FALSE |
| Cluster 10 | Cluster 7 | 0.143 | 0.9727 | -0.2343 | 0.5203 | FALSE | conscienciousness_adj_z | FALSE |
| Cluster 10 | Cluster 8 | -0.1241 | 0.9925 | -0.5181 | 0.2699 | FALSE | conscienciousness_adj_z | FALSE |
| Cluster 10 | Cluster 9 | -0.3541 | 0.7528 | -0.9862 | 0.278 | FALSE | conscienciousness_adj_z | FALSE |
| Cluster 2 | Cluster 3 | -0.127 | 0 | -0.1687 | -0.0854 | TRUE | conscienciousness_adj_z | FALSE |
| Cluster 2 | Cluster 4 | -0.3379 | 0 | -0.4206 | -0.2551 | TRUE | conscienciousness_adj_z | FALSE |
| Cluster 2 | Cluster 5 | -0.5179 | 0 | -0.6955 | -0.3403 | TRUE | conscienciousness_adj_z | FALSE |
| Cluster 2 | Cluster 6 | -0.4572 | 0 | -0.6577 | -0.2567 | TRUE | conscienciousness_adj_z | FALSE |
| Cluster 2 | Cluster 7 | -0.3527 | 0 | -0.5349 | -0.1705 | TRUE | conscienciousness_adj_z | FALSE |
| Cluster 2 | Cluster 8 | -0.6198 | 0 | -0.8344 | -0.4051 | TRUE | conscienciousness_adj_z | FALSE |
| Cluster 2 | Cluster 9 | -0.8498 | 0 | -1.3887 | -0.3108 | TRUE | conscienciousness_adj_z | FALSE |
| Cluster 3 | Cluster 4 | -0.2108 | 0 | -0.2986 | -0.123 | TRUE | conscienciousness_adj_z | FALSE |
| Cluster 3 | Cluster 5 | -0.3909 | 0 | -0.5709 | -0.2109 | TRUE | conscienciousness_adj_z | FALSE |
| Cluster 3 | Cluster 6 | -0.3301 | 0 | -0.5328 | -0.1275 | TRUE | conscienciousness_adj_z | FALSE |
| Cluster 3 | Cluster 7 | -0.2256 | 0.0043 | -0.4102 | -0.0411 | TRUE | conscienciousness_adj_z | FALSE |
| Cluster 3 | Cluster 8 | -0.4927 | 0 | -0.7094 | -0.276 | TRUE | conscienciousness_adj_z | FALSE |
| Cluster 3 | Cluster 9 | -0.7227 | 0.0009 | -1.2625 | -0.183 | TRUE | conscienciousness_adj_z | FALSE |
| Cluster 4 | Cluster 5 | -0.1801 | 0.0944 | -0.3738 | 0.0136 | FALSE | conscienciousness_adj_z | FALSE |
| Cluster 4 | Cluster 6 | -0.1193 | 0.7622 | -0.3342 | 0.0955 | FALSE | conscienciousness_adj_z | FALSE |
| Cluster 4 | Cluster 7 | -0.0148 | 1 | -0.2127 | 0.1831 | FALSE | conscienciousness_adj_z | FALSE |
| Cluster 4 | Cluster 8 | -0.2819 | 0.0037 | -0.5101 | -0.0537 | TRUE | conscienciousness_adj_z | FALSE |
| Cluster 4 | Cluster 9 | -0.5119 | 0.0862 | -1.0564 | 0.0325 | FALSE | conscienciousness_adj_z | FALSE |
| Cluster 5 | Cluster 6 | 0.0607 | 0.9994 | -0.2055 | 0.3269 | FALSE | conscienciousness_adj_z | FALSE |
| Cluster 5 | Cluster 7 | 0.1653 | 0.5495 | -0.0874 | 0.418 | FALSE | conscienciousness_adj_z | FALSE |
| Cluster 5 | Cluster 8 | -0.1018 | 0.9778 | -0.3789 | 0.1752 | FALSE | conscienciousness_adj_z | FALSE |
| Cluster 5 | Cluster 9 | -0.3318 | 0.7011 | -0.8985 | 0.2348 | FALSE | conscienciousness_adj_z | FALSE |
| Cluster 6 | Cluster 7 | 0.1045 | 0.968 | -0.1648 | 0.3738 | FALSE | conscienciousness_adj_z | FALSE |
| Cluster 6 | Cluster 8 | -0.1626 | 0.7604 | -0.4548 | 0.1297 | FALSE | conscienciousness_adj_z | FALSE |
| Cluster 6 | Cluster 9 | -0.3926 | 0.4827 | -0.9668 | 0.1817 | FALSE | conscienciousness_adj_z | FALSE |
| Cluster 7 | Cluster 8 | -0.2671 | 0.0765 | -0.5471 | 0.0129 | FALSE | conscienciousness_adj_z | FALSE |
| Cluster 7 | Cluster 9 | -0.4971 | 0.1473 | -1.0653 | 0.071 | FALSE | conscienciousness_adj_z | FALSE |
| Cluster 8 | Cluster 9 | -0.23 | 0.963 | -0.8094 | 0.3494 | FALSE | conscienciousness_adj_z | FALSE |
| Cluster 1 | Cluster 10 | 0.1538 | 0.9113 | -0.1818 | 0.4895 | FALSE | agreeableness_adj_z | FALSE |
| Cluster 1 | Cluster 2 | 0.0953 | 0 | 0.0499 | 0.1407 | TRUE | agreeableness_adj_z | FALSE |
| Cluster 1 | Cluster 3 | 0.0682 | 0.0027 | 0.014 | 0.1224 | TRUE | agreeableness_adj_z | FALSE |
| Cluster 1 | Cluster 4 | 0.0951 | 0.0289 | 0.005 | 0.1852 | TRUE | agreeableness_adj_z | FALSE |
| Cluster 1 | Cluster 5 | 0.2988 | 0 | 0.1167 | 0.4808 | TRUE | agreeableness_adj_z | FALSE |
| Cluster 1 | Cluster 6 | -0.0467 | 0.9994 | -0.2514 | 0.158 | FALSE | agreeableness_adj_z | FALSE |
| Cluster 1 | Cluster 7 | 0.1184 | 0.5942 | -0.0682 | 0.305 | FALSE | agreeableness_adj_z | FALSE |
| Cluster 1 | Cluster 8 | 0.2252 | 0.0377 | 0.0064 | 0.444 | TRUE | agreeableness_adj_z | FALSE |
| Cluster 1 | Cluster 9 | -0.3987 | 0.3756 | -0.9423 | 0.1449 | FALSE | agreeableness_adj_z | FALSE |
| Cluster 10 | Cluster 2 | -0.0585 | 0.9999 | -0.3924 | 0.2754 | FALSE | agreeableness_adj_z | FALSE |
| Cluster 10 | Cluster 3 | -0.0856 | 0.9985 | -0.4208 | 0.2496 | FALSE | agreeableness_adj_z | FALSE |
| Cluster 10 | Cluster 4 | -0.0587 | 0.9999 | -0.4016 | 0.2841 | FALSE | agreeableness_adj_z | FALSE |
| Cluster 10 | Cluster 5 | 0.145 | 0.9703 | -0.2326 | 0.5225 | FALSE | agreeableness_adj_z | FALSE |
| Cluster 10 | Cluster 6 | -0.2005 | 0.8333 | -0.5895 | 0.1885 | FALSE | agreeableness_adj_z | FALSE |
| Cluster 10 | Cluster 7 | -0.0355 | 1 | -0.4152 | 0.3443 | FALSE | agreeableness_adj_z | FALSE |
| Cluster 10 | Cluster 8 | 0.0714 | 0.9999 | -0.3252 | 0.468 | FALSE | agreeableness_adj_z | FALSE |
| Cluster 10 | Cluster 9 | -0.5525 | 0.1552 | -1.1888 | 0.0838 | FALSE | agreeableness_adj_z | FALSE |
| Cluster 2 | Cluster 3 | -0.0271 | 0.5692 | -0.069 | 0.0149 | FALSE | agreeableness_adj_z | FALSE |
| Cluster 2 | Cluster 4 | -0.0002 | 1 | -0.0835 | 0.0831 | FALSE | agreeableness_adj_z | FALSE |
| Cluster 2 | Cluster 5 | 0.2035 | 0.0118 | 0.0247 | 0.3823 | TRUE | agreeableness_adj_z | FALSE |
| Cluster 2 | Cluster 6 | -0.142 | 0.4388 | -0.3438 | 0.0598 | FALSE | agreeableness_adj_z | FALSE |
| Cluster 2 | Cluster 7 | 0.0231 | 1 | -0.1603 | 0.2065 | FALSE | agreeableness_adj_z | FALSE |
| Cluster 2 | Cluster 8 | 0.1299 | 0.6681 | -0.0862 | 0.346 | FALSE | agreeableness_adj_z | FALSE |
| Cluster 2 | Cluster 9 | -0.494 | 0.1109 | -1.0365 | 0.0486 | FALSE | agreeableness_adj_z | FALSE |
| Cluster 3 | Cluster 4 | 0.0268 | 0.9943 | -0.0616 | 0.1152 | FALSE | agreeableness_adj_z | FALSE |
| Cluster 3 | Cluster 5 | 0.2305 | 0.0023 | 0.0493 | 0.4117 | TRUE | agreeableness_adj_z | FALSE |
| Cluster 3 | Cluster 6 | -0.1149 | 0.7464 | -0.3189 | 0.089 | FALSE | agreeableness_adj_z | FALSE |
| Cluster 3 | Cluster 7 | 0.0501 | 0.9977 | -0.1357 | 0.2359 | FALSE | agreeableness_adj_z | FALSE |
| Cluster 3 | Cluster 8 | 0.1569 | 0.4045 | -0.0612 | 0.3751 | FALSE | agreeableness_adj_z | FALSE |
| Cluster 3 | Cluster 9 | -0.4669 | 0.1659 | -1.0103 | 0.0764 | FALSE | agreeableness_adj_z | FALSE |
| Cluster 4 | Cluster 5 | 0.2037 | 0.0322 | 0.0087 | 0.3987 | TRUE | agreeableness_adj_z | FALSE |
| Cluster 4 | Cluster 6 | -0.1418 | 0.5463 | -0.3581 | 0.0745 | FALSE | agreeableness_adj_z | FALSE |
| Cluster 4 | Cluster 7 | 0.0233 | 1 | -0.176 | 0.2225 | FALSE | agreeableness_adj_z | FALSE |
| Cluster 4 | Cluster 8 | 0.1301 | 0.7405 | -0.0996 | 0.3598 | FALSE | agreeableness_adj_z | FALSE |
| Cluster 4 | Cluster 9 | -0.4938 | 0.12 | -1.0419 | 0.0543 | FALSE | agreeableness_adj_z | FALSE |
| Cluster 5 | Cluster 6 | -0.3455 | 0.0019 | -0.6134 | -0.0775 | TRUE | agreeableness_adj_z | FALSE |
| Cluster 5 | Cluster 7 | -0.1804 | 0.4266 | -0.4348 | 0.074 | FALSE | agreeableness_adj_z | FALSE |
| Cluster 5 | Cluster 8 | -0.0736 | 0.998 | -0.3525 | 0.2053 | FALSE | agreeableness_adj_z | FALSE |
| Cluster 5 | Cluster 9 | -0.6975 | 0.0043 | -1.2679 | -0.127 | TRUE | agreeableness_adj_z | FALSE |
| Cluster 6 | Cluster 7 | 0.165 | 0.651 | -0.106 | 0.4361 | FALSE | agreeableness_adj_z | FALSE |
| Cluster 6 | Cluster 8 | 0.2719 | 0.099 | -0.0223 | 0.5661 | FALSE | agreeableness_adj_z | FALSE |
| Cluster 6 | Cluster 9 | -0.352 | 0.6509 | -0.9301 | 0.2261 | FALSE | agreeableness_adj_z | FALSE |
| Cluster 7 | Cluster 8 | 0.1068 | 0.9727 | -0.1751 | 0.3887 | FALSE | agreeableness_adj_z | FALSE |
| Cluster 7 | Cluster 9 | -0.517 | 0.117 | -1.089 | 0.0549 | FALSE | agreeableness_adj_z | FALSE |
| Cluster 8 | Cluster 9 | -0.6239 | 0.0249 | -1.2071 | -0.0406 | TRUE | agreeableness_adj_z | FALSE |
| Cluster 1 | Cluster 10 | -0.0566 | 0.9999 | -0.3921 | 0.2789 | FALSE | perfectionism_adj_z | FALSE |
| Cluster 1 | Cluster 2 | 0.0776 | 0 | 0.0322 | 0.123 | TRUE | perfectionism_adj_z | FALSE |
| Cluster 1 | Cluster 3 | 0.0124 | 0.9994 | -0.0418 | 0.0665 | FALSE | perfectionism_adj_z | FALSE |
| Cluster 1 | Cluster 4 | -0.0147 | 1 | -0.1048 | 0.0754 | FALSE | perfectionism_adj_z | FALSE |
| Cluster 1 | Cluster 5 | -0.2199 | 0.0052 | -0.4019 | -0.0379 | TRUE | perfectionism_adj_z | FALSE |
| Cluster 1 | Cluster 6 | -0.041 | 0.9998 | -0.2457 | 0.1636 | FALSE | perfectionism_adj_z | FALSE |
| Cluster 1 | Cluster 7 | -0.0601 | 0.9912 | -0.2467 | 0.1264 | FALSE | perfectionism_adj_z | FALSE |
| Cluster 1 | Cluster 8 | -0.3033 | 0.0005 | -0.5221 | -0.0846 | TRUE | perfectionism_adj_z | FALSE |
| Cluster 1 | Cluster 9 | -0.0462 | 1 | -0.5897 | 0.4972 | FALSE | perfectionism_adj_z | FALSE |
| Cluster 10 | Cluster 2 | 0.1342 | 0.9598 | -0.1995 | 0.468 | FALSE | perfectionism_adj_z | FALSE |
| Cluster 10 | Cluster 3 | 0.069 | 0.9997 | -0.2661 | 0.404 | FALSE | perfectionism_adj_z | FALSE |
| Cluster 10 | Cluster 4 | 0.0419 | 1 | -0.3008 | 0.3846 | FALSE | perfectionism_adj_z | FALSE |
| Cluster 10 | Cluster 5 | -0.1632 | 0.9368 | -0.5407 | 0.2142 | FALSE | perfectionism_adj_z | FALSE |
| Cluster 10 | Cluster 6 | 0.0156 | 1 | -0.3733 | 0.4045 | FALSE | perfectionism_adj_z | FALSE |
| Cluster 10 | Cluster 7 | -0.0035 | 1 | -0.3831 | 0.3761 | FALSE | perfectionism_adj_z | FALSE |
| Cluster 10 | Cluster 8 | -0.2467 | 0.6211 | -0.6432 | 0.1497 | FALSE | perfectionism_adj_z | FALSE |
| Cluster 10 | Cluster 9 | 0.0104 | 1 | -0.6257 | 0.6465 | FALSE | perfectionism_adj_z | FALSE |
| Cluster 2 | Cluster 3 | -0.0653 | 0 | -0.1072 | -0.0234 | TRUE | perfectionism_adj_z | FALSE |
| Cluster 2 | Cluster 4 | -0.0923 | 0.0164 | -0.1756 | -0.0091 | TRUE | perfectionism_adj_z | FALSE |
| Cluster 2 | Cluster 5 | -0.2975 | 0 | -0.4762 | -0.1188 | TRUE | perfectionism_adj_z | FALSE |
| Cluster 2 | Cluster 6 | -0.1187 | 0.6958 | -0.3204 | 0.0831 | FALSE | perfectionism_adj_z | FALSE |
| Cluster 2 | Cluster 7 | -0.1378 | 0.3396 | -0.3211 | 0.0456 | FALSE | perfectionism_adj_z | FALSE |
| Cluster 2 | Cluster 8 | -0.381 | 0 | -0.597 | -0.1649 | TRUE | perfectionism_adj_z | FALSE |
| Cluster 2 | Cluster 9 | -0.1239 | 0.9994 | -0.6662 | 0.4185 | FALSE | perfectionism_adj_z | FALSE |
| Cluster 3 | Cluster 4 | -0.0271 | 0.9939 | -0.1154 | 0.0613 | FALSE | perfectionism_adj_z | FALSE |
| Cluster 3 | Cluster 5 | -0.2322 | 0.002 | -0.4134 | -0.0511 | TRUE | perfectionism_adj_z | FALSE |
| Cluster 3 | Cluster 6 | -0.0534 | 0.9982 | -0.2573 | 0.1505 | FALSE | perfectionism_adj_z | FALSE |
| Cluster 3 | Cluster 7 | -0.0725 | 0.9669 | -0.2582 | 0.1132 | FALSE | perfectionism_adj_z | FALSE |
| Cluster 3 | Cluster 8 | -0.3157 | 0.0002 | -0.5337 | -0.0976 | TRUE | perfectionism_adj_z | FALSE |
| Cluster 3 | Cluster 9 | -0.0586 | 1 | -0.6017 | 0.4846 | FALSE | perfectionism_adj_z | FALSE |
| Cluster 4 | Cluster 5 | -0.2052 | 0.0297 | -0.4001 | -0.0102 | TRUE | perfectionism_adj_z | FALSE |
| Cluster 4 | Cluster 6 | -0.0263 | 1 | -0.2426 | 0.1899 | FALSE | perfectionism_adj_z | FALSE |
| Cluster 4 | Cluster 7 | -0.0454 | 0.9994 | -0.2446 | 0.1537 | FALSE | perfectionism_adj_z | FALSE |
| Cluster 4 | Cluster 8 | -0.2886 | 0.0028 | -0.5182 | -0.059 | TRUE | perfectionism_adj_z | FALSE |
| Cluster 4 | Cluster 9 | -0.0315 | 1 | -0.5794 | 0.5164 | FALSE | perfectionism_adj_z | FALSE |
| Cluster 5 | Cluster 6 | 0.1788 | 0.5187 | -0.0891 | 0.4467 | FALSE | perfectionism_adj_z | FALSE |
| Cluster 5 | Cluster 7 | 0.1597 | 0.6081 | -0.0946 | 0.4141 | FALSE | perfectionism_adj_z | FALSE |
| Cluster 5 | Cluster 8 | -0.0835 | 0.9949 | -0.3623 | 0.1953 | FALSE | perfectionism_adj_z | FALSE |
| Cluster 5 | Cluster 9 | 0.1736 | 0.9942 | -0.3966 | 0.7439 | FALSE | perfectionism_adj_z | FALSE |
| Cluster 6 | Cluster 7 | -0.0191 | 1 | -0.2901 | 0.2519 | FALSE | perfectionism_adj_z | FALSE |
| Cluster 6 | Cluster 8 | -0.2623 | 0.129 | -0.5564 | 0.0318 | FALSE | perfectionism_adj_z | FALSE |
| Cluster 6 | Cluster 9 | -0.0052 | 1 | -0.5831 | 0.5727 | FALSE | perfectionism_adj_z | FALSE |
| Cluster 7 | Cluster 8 | -0.2432 | 0.1613 | -0.525 | 0.0386 | FALSE | perfectionism_adj_z | FALSE |
| Cluster 7 | Cluster 9 | 0.0139 | 1 | -0.5578 | 0.5856 | FALSE | perfectionism_adj_z | FALSE |
| Cluster 8 | Cluster 9 | 0.2571 | 0.9291 | -0.3259 | 0.8401 | FALSE | perfectionism_adj_z | FALSE |
| Cluster 1 | Cluster 10 | 0.2775 | 0.1941 | -0.0537 | 0.6088 | FALSE | reward_drive_adj_z | FALSE |
| Cluster 1 | Cluster 2 | -0.3222 | 0 | -0.367 | -0.2774 | TRUE | reward_drive_adj_z | FALSE |
| Cluster 1 | Cluster 3 | -0.161 | 0 | -0.2145 | -0.1075 | TRUE | reward_drive_adj_z | FALSE |
| Cluster 1 | Cluster 4 | 0.1256 | 0.0003 | 0.0367 | 0.2145 | TRUE | reward_drive_adj_z | FALSE |
| Cluster 1 | Cluster 5 | 0.4356 | 0 | 0.256 | 0.6153 | TRUE | reward_drive_adj_z | FALSE |
| Cluster 1 | Cluster 6 | 0.2633 | 0.0015 | 0.0612 | 0.4653 | TRUE | reward_drive_adj_z | FALSE |
| Cluster 1 | Cluster 7 | 0.3115 | 0 | 0.1274 | 0.4957 | TRUE | reward_drive_adj_z | FALSE |
| Cluster 1 | Cluster 8 | 0.1377 | 0.5864 | -0.0782 | 0.3537 | FALSE | reward_drive_adj_z | FALSE |
| Cluster 1 | Cluster 9 | 0.8226 | 0.0001 | 0.2862 | 1.3591 | TRUE | reward_drive_adj_z | FALSE |
| Cluster 10 | Cluster 2 | -0.5998 | 0 | -0.9292 | -0.2703 | TRUE | reward_drive_adj_z | FALSE |
| Cluster 10 | Cluster 3 | -0.4385 | 0.0011 | -0.7693 | -0.1078 | TRUE | reward_drive_adj_z | FALSE |
| Cluster 10 | Cluster 4 | -0.1519 | 0.921 | -0.4903 | 0.1864 | FALSE | reward_drive_adj_z | FALSE |
| Cluster 10 | Cluster 5 | 0.1581 | 0.9438 | -0.2145 | 0.5307 | FALSE | reward_drive_adj_z | FALSE |
| Cluster 10 | Cluster 6 | -0.0143 | 1 | -0.3981 | 0.3696 | FALSE | reward_drive_adj_z | FALSE |
| Cluster 10 | Cluster 7 | 0.034 | 1 | -0.3408 | 0.4088 | FALSE | reward_drive_adj_z | FALSE |
| Cluster 10 | Cluster 8 | -0.1398 | 0.9817 | -0.5312 | 0.2516 | FALSE | reward_drive_adj_z | FALSE |
| Cluster 10 | Cluster 9 | 0.5451 | 0.1554 | -0.0829 | 1.1731 | FALSE | reward_drive_adj_z | FALSE |
| Cluster 2 | Cluster 3 | 0.1612 | 0 | 0.1198 | 0.2026 | TRUE | reward_drive_adj_z | FALSE |
| Cluster 2 | Cluster 4 | 0.4478 | 0 | 0.3656 | 0.53 | TRUE | reward_drive_adj_z | FALSE |
| Cluster 2 | Cluster 5 | 0.7578 | 0 | 0.5814 | 0.9343 | TRUE | reward_drive_adj_z | FALSE |
| Cluster 2 | Cluster 6 | 0.5855 | 0 | 0.3863 | 0.7847 | TRUE | reward_drive_adj_z | FALSE |
| Cluster 2 | Cluster 7 | 0.6338 | 0 | 0.4528 | 0.8148 | TRUE | reward_drive_adj_z | FALSE |
| Cluster 2 | Cluster 8 | 0.4599 | 0 | 0.2467 | 0.6732 | TRUE | reward_drive_adj_z | FALSE |
| Cluster 2 | Cluster 9 | 1.1448 | 0 | 0.6094 | 1.6802 | TRUE | reward_drive_adj_z | FALSE |
| Cluster 3 | Cluster 4 | 0.2866 | 0 | 0.1994 | 0.3738 | TRUE | reward_drive_adj_z | FALSE |
| Cluster 3 | Cluster 5 | 0.5966 | 0 | 0.4178 | 0.7755 | TRUE | reward_drive_adj_z | FALSE |
| Cluster 3 | Cluster 6 | 0.4243 | 0 | 0.223 | 0.6256 | TRUE | reward_drive_adj_z | FALSE |
| Cluster 3 | Cluster 7 | 0.4726 | 0 | 0.2892 | 0.6559 | TRUE | reward_drive_adj_z | FALSE |
| Cluster 3 | Cluster 8 | 0.2987 | 0.0005 | 0.0835 | 0.514 | TRUE | reward_drive_adj_z | FALSE |
| Cluster 3 | Cluster 9 | 0.9836 | 0 | 0.4474 | 1.5198 | TRUE | reward_drive_adj_z | FALSE |
| Cluster 4 | Cluster 5 | 0.31 | 0 | 0.1176 | 0.5024 | TRUE | reward_drive_adj_z | FALSE |
| Cluster 4 | Cluster 6 | 0.1377 | 0.5699 | -0.0758 | 0.3511 | FALSE | reward_drive_adj_z | FALSE |
| Cluster 4 | Cluster 7 | 0.186 | 0.0821 | -0.0107 | 0.3826 | FALSE | reward_drive_adj_z | FALSE |
| Cluster 4 | Cluster 8 | 0.0121 | 1 | -0.2145 | 0.2388 | FALSE | reward_drive_adj_z | FALSE |
| Cluster 4 | Cluster 9 | 0.697 | 0.0019 | 0.1562 | 1.2379 | TRUE | reward_drive_adj_z | FALSE |
| Cluster 5 | Cluster 6 | -0.1723 | 0.5548 | -0.4368 | 0.0921 | FALSE | reward_drive_adj_z | FALSE |
| Cluster 5 | Cluster 7 | -0.1241 | 0.8657 | -0.3751 | 0.127 | FALSE | reward_drive_adj_z | FALSE |
| Cluster 5 | Cluster 8 | -0.2979 | 0.0218 | -0.5731 | -0.0227 | TRUE | reward_drive_adj_z | FALSE |
| Cluster 5 | Cluster 9 | 0.387 | 0.4742 | -0.176 | 0.95 | FALSE | reward_drive_adj_z | FALSE |
| Cluster 6 | Cluster 7 | 0.0483 | 0.9999 | -0.2193 | 0.3158 | FALSE | reward_drive_adj_z | FALSE |
| Cluster 6 | Cluster 8 | -0.1256 | 0.9369 | -0.4159 | 0.1648 | FALSE | reward_drive_adj_z | FALSE |
| Cluster 6 | Cluster 9 | 0.5593 | 0.0601 | -0.0111 | 1.1298 | FALSE | reward_drive_adj_z | FALSE |
| Cluster 7 | Cluster 8 | -0.1738 | 0.6153 | -0.452 | 0.1043 | FALSE | reward_drive_adj_z | FALSE |
| Cluster 7 | Cluster 9 | 0.5111 | 0.1156 | -0.0533 | 1.0755 | FALSE | reward_drive_adj_z | FALSE |
| Cluster 8 | Cluster 9 | 0.6849 | 0.0064 | 0.1093 | 1.2605 | TRUE | reward_drive_adj_z | FALSE |

**Supp Table 4. Personality and compulsivity post-hoc**

**Part IV - Post-hoc analysis of main results**

| group1 | group2 | meandiff | p | lower | upper | sig | variable | sig_corrected |
| --- | --- | --- | --- | --- | --- | --- | --- | --- |
| Cannabis and cocaine users | Cannabis users | -0.0586 | 0.9958 | -0.2596 | 0.1424 | FALSE | anxiety | FALSE |
| Cannabis and cocaine users | Cocaine users | -0.0876 | 0.9882 | -0.349 | 0.1737 | FALSE | anxiety | FALSE |
| Cannabis and cocaine users | Drug use history but no pandemic use | -0.0992 | 0.8105 | -0.2868 | 0.0883 | FALSE | anxiety | FALSE |
| Cannabis and cocaine users | Extreme polydrug users | 0.139 | 0.9991 | -0.4412 | 0.7191 | FALSE | anxiety | FALSE |
| Cannabis and cocaine users | MDMA/ecstasy and cannabis users | -0.4142 | 0.0212 | -0.7962 | -0.0323 | TRUE | anxiety | FALSE |
| Cannabis and cocaine users | Never used drugs | -0.0961 | 0.823 | -0.2802 | 0.0881 | FALSE | anxiety | FALSE |
| Cannabis and cocaine users | Psychedelics and cannabis users | 0.0712 | 0.9988 | -0.2173 | 0.3597 | FALSE | anxiety | FALSE |
| Cannabis and cocaine users | Unknown/Unwilling to disclose | -0.1323 | 0.4278 | -0.3189 | 0.0544 | FALSE | anxiety | FALSE |
| Cannabis and cocaine users | Users of other drugs | -0.0114 | 1 | -0.2881 | 0.2653 | FALSE | anxiety | FALSE |
| Cannabis users | Cocaine users | -0.029 | 1 | -0.2334 | 0.1754 | FALSE | anxiety | FALSE |
| Cannabis users | Drug use history but no pandemic use | -0.0406 | 0.9333 | -0.1337 | 0.0524 | FALSE | anxiety | FALSE |
| Cannabis users | Extreme polydrug users | 0.1976 | 0.9825 | -0.3593 | 0.7544 | FALSE | anxiety | FALSE |
| Cannabis users | MDMA/ecstasy and cannabis users | -0.3556 | 0.0375 | -0.7011 | -0.0102 | TRUE | anxiety | FALSE |
| Cannabis users | Never used drugs | -0.0375 | 0.9341 | -0.1234 | 0.0485 | FALSE | anxiety | FALSE |
| Cannabis users | Psychedelics and cannabis users | 0.1298 | 0.7816 | -0.1083 | 0.3679 | FALSE | anxiety | FALSE |
| Cannabis users | Unknown/Unwilling to disclose | -0.0736 | 0.2393 | -0.1648 | 0.0175 | FALSE | anxiety | FALSE |
| Cannabis users | Users of other drugs | 0.0472 | 0.9997 | -0.1765 | 0.271 | FALSE | anxiety | FALSE |
| Cocaine users | Drug use history but no pandemic use | -0.0116 | 1 | -0.2028 | 0.1797 | FALSE | anxiety | FALSE |
| Cocaine users | Extreme polydrug users | 0.2266 | 0.9672 | -0.3548 | 0.808 | FALSE | anxiety | FALSE |
| Cocaine users | MDMA/ecstasy and cannabis users | -0.3266 | 0.1764 | -0.7103 | 0.0571 | FALSE | anxiety | FALSE |
| Cocaine users | Never used drugs | -0.0084 | 1 | -0.1963 | 0.1795 | FALSE | anxiety | FALSE |
| Cocaine users | Psychedelics and cannabis users | 0.1588 | 0.78 | -0.1321 | 0.4497 | FALSE | anxiety | FALSE |
| Cocaine users | Unknown/Unwilling to disclose | -0.0446 | 0.9992 | -0.235 | 0.1457 | FALSE | anxiety | FALSE |
| Cocaine users | Users of other drugs | 0.0763 | 0.9974 | -0.203 | 0.3555 | FALSE | anxiety | FALSE |
| Drug use history but no pandemic use | Extreme polydrug users | 0.2382 | 0.9378 | -0.314 | 0.7903 | FALSE | anxiety | FALSE |
| Drug use history but no pandemic use | MDMA/ecstasy and cannabis users | -0.315 | 0.0922 | -0.6528 | 0.0228 | FALSE | anxiety | FALSE |
| Drug use history but no pandemic use | Never used drugs | 0.0032 | 1 | -0.0436 | 0.0499 | FALSE | anxiety | FALSE |
| Drug use history but no pandemic use | Psychedelics and cannabis users | 0.1704 | 0.3403 | -0.0565 | 0.3973 | FALSE | anxiety | FALSE |
| Drug use history but no pandemic use | Unknown/Unwilling to disclose | -0.033 | 0.6861 | -0.0888 | 0.0227 | FALSE | anxiety | FALSE |
| Drug use history but no pandemic use | Users of other drugs | 0.0878 | 0.9511 | -0.1239 | 0.2996 | FALSE | anxiety | FALSE |
| Extreme polydrug users | MDMA/ecstasy and cannabis users | -0.5532 | 0.1674 | -1.1978 | 0.0914 | FALSE | anxiety | FALSE |
| Extreme polydrug users | Never used drugs | -0.235 | 0.9419 | -0.786 | 0.316 | FALSE | anxiety | FALSE |
| Extreme polydrug users | Psychedelics and cannabis users | -0.0678 | 1 | -0.6618 | 0.5263 | FALSE | anxiety | FALSE |
| Extreme polydrug users | Unknown/Unwilling to disclose | -0.2712 | 0.8695 | -0.8231 | 0.2806 | FALSE | anxiety | FALSE |
| Extreme polydrug users | Users of other drugs | -0.1503 | 0.9985 | -0.7388 | 0.4381 | FALSE | anxiety | FALSE |
| MDMA/ecstasy and cannabis users | Never used drugs | 0.3182 | 0.0812 | -0.0178 | 0.6541 | FALSE | anxiety | FALSE |
| MDMA/ecstasy and cannabis users | Psychedelics and cannabis users | 0.4854 | 0.0053 | 0.0827 | 0.8881 | TRUE | anxiety | FALSE |
| MDMA/ecstasy and cannabis users | Unknown/Unwilling to disclose | 0.282 | 0.1968 | -0.0553 | 0.6193 | FALSE | anxiety | FALSE |
| MDMA/ecstasy and cannabis users | Users of other drugs | 0.4029 | 0.0406 | 0.0085 | 0.7972 | TRUE | anxiety | FALSE |
| Never used drugs | Psychedelics and cannabis users | 0.1673 | 0.3495 | -0.0569 | 0.3914 | FALSE | anxiety | FALSE |
| Never used drugs | Unknown/Unwilling to disclose | -0.0362 | 0.1867 | -0.0791 | 0.0067 | FALSE | anxiety | FALSE |
| Never used drugs | Users of other drugs | 0.0847 | 0.9575 | -0.1241 | 0.2934 | FALSE | anxiety | FALSE |
| Psychedelics and cannabis users | Unknown/Unwilling to disclose | -0.2035 | 0.1213 | -0.4296 | 0.0227 | FALSE | anxiety | FALSE |
| Psychedelics and cannabis users | Users of other drugs | -0.0826 | 0.9976 | -0.3874 | 0.2222 | FALSE | anxiety | FALSE |
| Unknown/Unwilling to disclose | Users of other drugs | 0.1209 | 0.7272 | -0.0901 | 0.3318 | FALSE | anxiety | FALSE |
| Cannabis and cocaine users | Cannabis users | -0.0099 | 1 | -0.2109 | 0.1911 | FALSE | unable to stop worrying | FALSE |
| Cannabis and cocaine users | Cocaine users | 0.0121 | 1 | -0.2493 | 0.2735 | FALSE | unable to stop worrying | FALSE |
| Cannabis and cocaine users | Drug use history but no pandemic use | -0.0304 | 1 | -0.218 | 0.1572 | FALSE | unable to stop worrying | FALSE |
| Cannabis and cocaine users | Extreme polydrug users | -0.1352 | 0.9993 | -0.7154 | 0.4449 | FALSE | unable to stop worrying | FALSE |
| Cannabis and cocaine users | MDMA/ecstasy and cannabis users | -0.2934 | 0.3073 | -0.6753 | 0.0885 | FALSE | unable to stop worrying | FALSE |
| Cannabis and cocaine users | Never used drugs | -0.0107 | 1 | -0.1949 | 0.1734 | FALSE | unable to stop worrying | FALSE |
| Cannabis and cocaine users | Psychedelics and cannabis users | 0.0939 | 0.9905 | -0.1945 | 0.3824 | FALSE | unable to stop worrying | FALSE |
| Cannabis and cocaine users | Unknown/Unwilling to disclose | -0.0427 | 0.9994 | -0.2293 | 0.144 | FALSE | unable to stop worrying | FALSE |
| Cannabis and cocaine users | Users of other drugs | 0.1151 | 0.9502 | -0.1616 | 0.3918 | FALSE | unable to stop worrying | FALSE |
| Cannabis users | Cocaine users | 0.022 | 1 | -0.1824 | 0.2264 | FALSE | unable to stop worrying | FALSE |
| Cannabis users | Drug use history but no pandemic use | -0.0205 | 0.9995 | -0.1135 | 0.0726 | FALSE | unable to stop worrying | FALSE |
| Cannabis users | Extreme polydrug users | -0.1253 | 0.9994 | -0.6821 | 0.4315 | FALSE | unable to stop worrying | FALSE |
| Cannabis users | MDMA/ecstasy and cannabis users | -0.2835 | 0.219 | -0.6289 | 0.0619 | FALSE | unable to stop worrying | FALSE |
| Cannabis users | Never used drugs | -0.0008 | 1 | -0.0868 | 0.0851 | FALSE | unable to stop worrying | FALSE |
| Cannabis users | Psychedelics and cannabis users | 0.1038 | 0.9336 | -0.1343 | 0.342 | FALSE | unable to stop worrying | FALSE |
| Cannabis users | Unknown/Unwilling to disclose | -0.0328 | 0.9809 | -0.1239 | 0.0584 | FALSE | unable to stop worrying | FALSE |
| Cannabis users | Users of other drugs | 0.125 | 0.7553 | -0.0987 | 0.3488 | FALSE | unable to stop worrying | FALSE |
| Cocaine users | Drug use history but no pandemic use | -0.0425 | 0.9995 | -0.2338 | 0.1488 | FALSE | unable to stop worrying | FALSE |
| Cocaine users | Extreme polydrug users | -0.1473 | 0.9986 | -0.7287 | 0.434 | FALSE | unable to stop worrying | FALSE |
| Cocaine users | MDMA/ecstasy and cannabis users | -0.3055 | 0.258 | -0.6892 | 0.0782 | FALSE | unable to stop worrying | FALSE |
| Cocaine users | Never used drugs | -0.0228 | 1 | -0.2108 | 0.1651 | FALSE | unable to stop worrying | FALSE |
| Cocaine users | Psychedelics and cannabis users | 0.0818 | 0.9968 | -0.2091 | 0.3727 | FALSE | unable to stop worrying | FALSE |
| Cocaine users | Unknown/Unwilling to disclose | -0.0548 | 0.9962 | -0.2451 | 0.1356 | FALSE | unable to stop worrying | FALSE |
| Cocaine users | Users of other drugs | 0.103 | 0.9772 | -0.1762 | 0.3823 | FALSE | unable to stop worrying | FALSE |
| Drug use history but no pandemic use | Extreme polydrug users | -0.1048 | 0.9999 | -0.657 | 0.4473 | FALSE | unable to stop worrying | FALSE |
| Drug use history but no pandemic use | MDMA/ecstasy and cannabis users | -0.263 | 0.2885 | -0.6008 | 0.0748 | FALSE | unable to stop worrying | FALSE |
| Drug use history but no pandemic use | Never used drugs | 0.0197 | 0.9469 | -0.0271 | 0.0664 | FALSE | unable to stop worrying | FALSE |
| Drug use history but no pandemic use | Psychedelics and cannabis users | 0.1243 | 0.7764 | -0.1026 | 0.3512 | FALSE | unable to stop worrying | FALSE |
| Drug use history but no pandemic use | Unknown/Unwilling to disclose | -0.0123 | 0.9995 | -0.068 | 0.0435 | FALSE | unable to stop worrying | FALSE |
| Drug use history but no pandemic use | Users of other drugs | 0.1455 | 0.4747 | -0.0662 | 0.3573 | FALSE | unable to stop worrying | FALSE |
| Extreme polydrug users | MDMA/ecstasy and cannabis users | -0.1582 | 0.9989 | -0.8028 | 0.4864 | FALSE | unable to stop worrying | FALSE |
| Extreme polydrug users | Never used drugs | 0.1245 | 0.9994 | -0.4265 | 0.6755 | FALSE | unable to stop worrying | FALSE |
| Extreme polydrug users | Psychedelics and cannabis users | 0.2292 | 0.9693 | -0.3649 | 0.8232 | FALSE | unable to stop worrying | FALSE |
| Extreme polydrug users | Unknown/Unwilling to disclose | 0.0925 | 1 | -0.4593 | 0.6444 | FALSE | unable to stop worrying | FALSE |
| Extreme polydrug users | Users of other drugs | 0.2504 | 0.9428 | -0.3381 | 0.8388 | FALSE | unable to stop worrying | FALSE |
| MDMA/ecstasy and cannabis users | Never used drugs | 0.2827 | 0.1892 | -0.0533 | 0.6186 | FALSE | unable to stop worrying | FALSE |
| MDMA/ecstasy and cannabis users | Psychedelics and cannabis users | 0.3873 | 0.0712 | -0.0153 | 0.79 | FALSE | unable to stop worrying | FALSE |
| MDMA/ecstasy and cannabis users | Unknown/Unwilling to disclose | 0.2507 | 0.3553 | -0.0866 | 0.588 | FALSE | unable to stop worrying | FALSE |
| MDMA/ecstasy and cannabis users | Users of other drugs | 0.4085 | 0.0352 | 0.0142 | 0.8029 | TRUE | unable to stop worrying | FALSE |
| Never used drugs | Psychedelics and cannabis users | 0.1047 | 0.9012 | -0.1194 | 0.3288 | FALSE | unable to stop worrying | FALSE |
| Never used drugs | Unknown/Unwilling to disclose | -0.0319 | 0.354 | -0.0748 | 0.011 | FALSE | unable to stop worrying | FALSE |
| Never used drugs | Users of other drugs | 0.1259 | 0.6638 | -0.0829 | 0.3346 | FALSE | unable to stop worrying | FALSE |
| Psychedelics and cannabis users | Unknown/Unwilling to disclose | -0.1366 | 0.6615 | -0.3628 | 0.0896 | FALSE | unable to stop worrying | FALSE |
| Psychedelics and cannabis users | Users of other drugs | 0.0212 | 1 | -0.2836 | 0.326 | FALSE | unable to stop worrying | FALSE |
| Unknown/Unwilling to disclose | Users of other drugs | 0.1578 | 0.346 | -0.0531 | 0.3687 | FALSE | unable to stop worrying | FALSE |
| Cannabis and cocaine users | Cannabis users | -0.0185 | 1 | -0.2194 | 0.1825 | FALSE | worrying about many things | FALSE |
| Cannabis and cocaine users | Cocaine users | 0.018 | 1 | -0.2434 | 0.2793 | FALSE | worrying about many things | FALSE |
| Cannabis and cocaine users | Drug use history but no pandemic use | -0.0183 | 1 | -0.2058 | 0.1693 | FALSE | worrying about many things | FALSE |
| Cannabis and cocaine users | Extreme polydrug users | 0.0743 | 1 | -0.5058 | 0.6544 | FALSE | worrying about many things | FALSE |
| Cannabis and cocaine users | MDMA/ecstasy and cannabis users | -0.1338 | 0.984 | -0.5157 | 0.2481 | FALSE | worrying about many things | FALSE |
| Cannabis and cocaine users | Never used drugs | 0.0091 | 1 | -0.175 | 0.1933 | FALSE | worrying about many things | FALSE |
| Cannabis and cocaine users | Psychedelics and cannabis users | 0.1599 | 0.7646 | -0.1286 | 0.4483 | FALSE | worrying about many things | FALSE |
| Cannabis and cocaine users | Unknown/Unwilling to disclose | -0.0166 | 1 | -0.2032 | 0.1701 | FALSE | worrying about many things | FALSE |
| Cannabis and cocaine users | Users of other drugs | 0.1289 | 0.9027 | -0.1478 | 0.4056 | FALSE | worrying about many things | FALSE |
| Cannabis users | Cocaine users | 0.0365 | 0.9999 | -0.168 | 0.2409 | FALSE | worrying about many things | FALSE |
| Cannabis users | Drug use history but no pandemic use | 0.0002 | 1 | -0.0928 | 0.0933 | FALSE | worrying about many things | FALSE |
| Cannabis users | Extreme polydrug users | 0.0928 | 1 | -0.464 | 0.6496 | FALSE | worrying about many things | FALSE |
| Cannabis users | MDMA/ecstasy and cannabis users | -0.1153 | 0.9886 | -0.4608 | 0.2301 | FALSE | worrying about many things | FALSE |
| Cannabis users | Never used drugs | 0.0276 | 0.9914 | -0.0584 | 0.1136 | FALSE | worrying about many things | FALSE |
| Cannabis users | Psychedelics and cannabis users | 0.1783 | 0.3444 | -0.0598 | 0.4164 | FALSE | worrying about many things | FALSE |
| Cannabis users | Unknown/Unwilling to disclose | 0.0019 | 1 | -0.0893 | 0.0931 | FALSE | worrying about many things | FALSE |
| Cannabis users | Users of other drugs | 0.1474 | 0.5389 | -0.0764 | 0.3711 | FALSE | worrying about many things | FALSE |
| Cocaine users | Drug use history but no pandemic use | -0.0362 | 0.9999 | -0.2275 | 0.155 | FALSE | worrying about many things | FALSE |
| Cocaine users | Extreme polydrug users | 0.0563 | 1 | -0.525 | 0.6377 | FALSE | worrying about many things | FALSE |
| Cocaine users | MDMA/ecstasy and cannabis users | -0.1518 | 0.9639 | -0.5355 | 0.2319 | FALSE | worrying about many things | FALSE |
| Cocaine users | Never used drugs | -0.0089 | 1 | -0.1968 | 0.1791 | FALSE | worrying about many things | FALSE |
| Cocaine users | Psychedelics and cannabis users | 0.1419 | 0.8748 | -0.149 | 0.4328 | FALSE | worrying about many things | FALSE |
| Cocaine users | Unknown/Unwilling to disclose | -0.0346 | 0.9999 | -0.2249 | 0.1558 | FALSE | worrying about many things | FALSE |
| Cocaine users | Users of other drugs | 0.1109 | 0.9629 | -0.1683 | 0.3901 | FALSE | worrying about many things | FALSE |
| Drug use history but no pandemic use | Extreme polydrug users | 0.0926 | 1 | -0.4596 | 0.6447 | FALSE | worrying about many things | FALSE |
| Drug use history but no pandemic use | MDMA/ecstasy and cannabis users | -0.1155 | 0.9865 | -0.4534 | 0.2223 | FALSE | worrying about many things | FALSE |
| Drug use history but no pandemic use | Never used drugs | 0.0274 | 0.7011 | -0.0194 | 0.0741 | FALSE | worrying about many things | FALSE |
| Drug use history but no pandemic use | Psychedelics and cannabis users | 0.1781 | 0.2774 | -0.0488 | 0.405 | FALSE | worrying about many things | FALSE |
| Drug use history but no pandemic use | Unknown/Unwilling to disclose | 0.0017 | 1 | -0.0541 | 0.0574 | FALSE | worrying about many things | FALSE |
| Drug use history but no pandemic use | Users of other drugs | 0.1471 | 0.4578 | -0.0646 | 0.3589 | FALSE | worrying about many things | FALSE |
| Extreme polydrug users | MDMA/ecstasy and cannabis users | -0.2081 | 0.9911 | -0.8527 | 0.4365 | FALSE | worrying about many things | FALSE |
| Extreme polydrug users | Never used drugs | -0.0652 | 1 | -0.6162 | 0.4858 | FALSE | worrying about many things | FALSE |
| Extreme polydrug users | Psychedelics and cannabis users | 0.0856 | 1 | -0.5085 | 0.6796 | FALSE | worrying about many things | FALSE |
| Extreme polydrug users | Unknown/Unwilling to disclose | -0.0909 | 1 | -0.6427 | 0.4609 | FALSE | worrying about many things | FALSE |
| Extreme polydrug users | Users of other drugs | 0.0546 | 1 | -0.5338 | 0.643 | FALSE | worrying about many things | FALSE |
| MDMA/ecstasy and cannabis users | Never used drugs | 0.1429 | 0.9428 | -0.193 | 0.4789 | FALSE | worrying about many things | FALSE |
| MDMA/ecstasy and cannabis users | Psychedelics and cannabis users | 0.2937 | 0.3841 | -0.109 | 0.6963 | FALSE | worrying about many things | FALSE |
| MDMA/ecstasy and cannabis users | Unknown/Unwilling to disclose | 0.1172 | 0.9849 | -0.2201 | 0.4545 | FALSE | worrying about many things | FALSE |
| MDMA/ecstasy and cannabis users | Users of other drugs | 0.2627 | 0.522 | -0.1317 | 0.657 | FALSE | worrying about many things | FALSE |
| Never used drugs | Psychedelics and cannabis users | 0.1507 | 0.5075 | -0.0734 | 0.3748 | FALSE | worrying about many things | FALSE |
| Never used drugs | Unknown/Unwilling to disclose | -0.0257 | 0.673 | -0.0686 | 0.0172 | FALSE | worrying about many things | FALSE |
| Never used drugs | Users of other drugs | 0.1198 | 0.7258 | -0.089 | 0.3285 | FALSE | worrying about many things | FALSE |
| Psychedelics and cannabis users | Unknown/Unwilling to disclose | -0.1764 | 0.2858 | -0.4026 | 0.0497 | FALSE | worrying about many things | FALSE |
| Psychedelics and cannabis users | Users of other drugs | -0.031 | 1 | -0.3357 | 0.2738 | FALSE | worrying about many things | FALSE |
| Unknown/Unwilling to disclose | Users of other drugs | 0.1455 | 0.4695 | -0.0655 | 0.3564 | FALSE | worrying about many things | FALSE |
| Cannabis and cocaine users | Cannabis users | 0.0037 | 1 | -0.1973 | 0.2046 | FALSE | unable to relax | FALSE |
| Cannabis and cocaine users | Cocaine users | 0.0081 | 1 | -0.2533 | 0.2695 | FALSE | unable to relax | FALSE |
| Cannabis and cocaine users | Drug use history but no pandemic use | 0.0083 | 1 | -0.1793 | 0.1959 | FALSE | unable to relax | FALSE |
| Cannabis and cocaine users | Extreme polydrug users | 0.0283 | 1 | -0.5519 | 0.6084 | FALSE | unable to relax | FALSE |
| Cannabis and cocaine users | MDMA/ecstasy and cannabis users | -0.2679 | 0.4433 | -0.6498 | 0.114 | FALSE | unable to relax | FALSE |
| Cannabis and cocaine users | Never used drugs | 0.0102 | 1 | -0.174 | 0.1944 | FALSE | unable to relax | FALSE |
| Cannabis and cocaine users | Psychedelics and cannabis users | 0.0851 | 0.9954 | -0.2034 | 0.3735 | FALSE | unable to relax | FALSE |
| Cannabis and cocaine users | Unknown/Unwilling to disclose | -0.0384 | 0.9997 | -0.225 | 0.1483 | FALSE | unable to relax | FALSE |
| Cannabis and cocaine users | Users of other drugs | 0.1142 | 0.9528 | -0.1626 | 0.3909 | FALSE | unable to relax | FALSE |
| Cannabis users | Cocaine users | 0.0044 | 1 | -0.2 | 0.2089 | FALSE | unable to relax | FALSE |
| Cannabis users | Drug use history but no pandemic use | 0.0046 | 1 | -0.0884 | 0.0977 | FALSE | unable to relax | FALSE |
| Cannabis users | Extreme polydrug users | 0.0246 | 1 | -0.5322 | 0.5814 | FALSE | unable to relax | FALSE |
| Cannabis users | MDMA/ecstasy and cannabis users | -0.2716 | 0.2753 | -0.617 | 0.0739 | FALSE | unable to relax | FALSE |
| Cannabis users | Never used drugs | 0.0065 | 1 | -0.0794 | 0.0925 | FALSE | unable to relax | FALSE |
| Cannabis users | Psychedelics and cannabis users | 0.0814 | 0.9865 | -0.1567 | 0.3195 | FALSE | unable to relax | FALSE |
| Cannabis users | Unknown/Unwilling to disclose | -0.042 | 0.9083 | -0.1332 | 0.0492 | FALSE | unable to relax | FALSE |
| Cannabis users | Users of other drugs | 0.1105 | 0.8661 | -0.1132 | 0.3342 | FALSE | unable to relax | FALSE |
| Cocaine users | Drug use history but no pandemic use | 0.0002 | 1 | -0.1911 | 0.1915 | FALSE | unable to relax | FALSE |
| Cocaine users | Extreme polydrug users | 0.0201 | 1 | -0.5612 | 0.6015 | FALSE | unable to relax | FALSE |
| Cocaine users | MDMA/ecstasy and cannabis users | -0.276 | 0.405 | -0.6597 | 0.1077 | FALSE | unable to relax | FALSE |
| Cocaine users | Never used drugs | 0.0021 | 1 | -0.1858 | 0.19 | FALSE | unable to relax | FALSE |
| Cocaine users | Psychedelics and cannabis users | 0.0769 | 0.998 | -0.214 | 0.3678 | FALSE | unable to relax | FALSE |
| Cocaine users | Unknown/Unwilling to disclose | -0.0465 | 0.9989 | -0.2368 | 0.1439 | FALSE | unable to relax | FALSE |
| Cocaine users | Users of other drugs | 0.106 | 0.9723 | -0.1732 | 0.3853 | FALSE | unable to relax | FALSE |
| Drug use history but no pandemic use | Extreme polydrug users | 0.02 | 1 | -0.5322 | 0.5721 | FALSE | unable to relax | FALSE |
| Drug use history but no pandemic use | MDMA/ecstasy and cannabis users | -0.2762 | 0.2238 | -0.614 | 0.0616 | FALSE | unable to relax | FALSE |
| Drug use history but no pandemic use | Never used drugs | 0.0019 | 1 | -0.0448 | 0.0487 | FALSE | unable to relax | FALSE |
| Drug use history but no pandemic use | Psychedelics and cannabis users | 0.0768 | 0.9875 | -0.1502 | 0.3037 | FALSE | unable to relax | FALSE |
| Drug use history but no pandemic use | Unknown/Unwilling to disclose | -0.0467 | 0.1952 | -0.1024 | 0.0091 | FALSE | unable to relax | FALSE |
| Drug use history but no pandemic use | Users of other drugs | 0.1059 | 0.8574 | -0.1059 | 0.3176 | FALSE | unable to relax | FALSE |
| Extreme polydrug users | MDMA/ecstasy and cannabis users | -0.2962 | 0.91 | -0.9408 | 0.3485 | FALSE | unable to relax | FALSE |
| Extreme polydrug users | Never used drugs | -0.018 | 1 | -0.5691 | 0.533 | FALSE | unable to relax | FALSE |
| Extreme polydrug users | Psychedelics and cannabis users | 0.0568 | 1 | -0.5373 | 0.6509 | FALSE | unable to relax | FALSE |
| Extreme polydrug users | Unknown/Unwilling to disclose | -0.0666 | 1 | -0.6185 | 0.4852 | FALSE | unable to relax | FALSE |
| Extreme polydrug users | Users of other drugs | 0.0859 | 1 | -0.5025 | 0.6743 | FALSE | unable to relax | FALSE |
| MDMA/ecstasy and cannabis users | Never used drugs | 0.2781 | 0.2085 | -0.0578 | 0.6141 | FALSE | unable to relax | FALSE |
| MDMA/ecstasy and cannabis users | Psychedelics and cannabis users | 0.3529 | 0.1457 | -0.0498 | 0.7557 | FALSE | unable to relax | FALSE |
| MDMA/ecstasy and cannabis users | Unknown/Unwilling to disclose | 0.2295 | 0.4898 | -0.1078 | 0.5668 | FALSE | unable to relax | FALSE |
| MDMA/ecstasy and cannabis users | Users of other drugs | 0.3821 | 0.0669 | -0.0123 | 0.7764 | FALSE | unable to relax | FALSE |
| Never used drugs | Psychedelics and cannabis users | 0.0748 | 0.9886 | -0.1493 | 0.299 | FALSE | unable to relax | FALSE |
| Never used drugs | Unknown/Unwilling to disclose | -0.0486 | 0.0127 | -0.0915 | -0.0057 | TRUE | unable to relax | FALSE |
| Never used drugs | Users of other drugs | 0.1039 | 0.8603 | -0.1048 | 0.3127 | FALSE | unable to relax | FALSE |
| Psychedelics and cannabis users | Unknown/Unwilling to disclose | -0.1234 | 0.7805 | -0.3496 | 0.1027 | FALSE | unable to relax | FALSE |
| Psychedelics and cannabis users | Users of other drugs | 0.0291 | 1 | -0.2757 | 0.3339 | FALSE | unable to relax | FALSE |
| Unknown/Unwilling to disclose | Users of other drugs | 0.1525 | 0.3971 | -0.0584 | 0.3635 | FALSE | unable to relax | FALSE |
| Cannabis and cocaine users | Cannabis users | -0.1905 | 0.0807 | -0.3915 | 0.0105 | FALSE | restlessness | FALSE |
| Cannabis and cocaine users | Cocaine users | -0.1766 | 0.5003 | -0.438 | 0.0847 | FALSE | restlessness | FALSE |
| Cannabis and cocaine users | Drug use history but no pandemic use | -0.2086 | 0.0158 | -0.3962 | -0.0211 | TRUE | restlessness | FALSE |
| Cannabis and cocaine users | Extreme polydrug users | 0.1269 | 0.9996 | -0.4533 | 0.7071 | FALSE | restlessness | FALSE |
| Cannabis and cocaine users | MDMA/ecstasy and cannabis users | -0.3693 | 0.0681 | -0.7512 | 0.0127 | FALSE | restlessness | FALSE |
| Cannabis and cocaine users | Never used drugs | -0.2016 | 0.0191 | -0.3858 | -0.0174 | TRUE | restlessness | FALSE |
| Cannabis and cocaine users | Psychedelics and cannabis users | -0.1887 | 0.5491 | -0.4772 | 0.0998 | FALSE | restlessness | FALSE |
| Cannabis and cocaine users | Unknown/Unwilling to disclose | -0.2377 | 0.0023 | -0.4244 | -0.051 | TRUE | restlessness | FALSE |
| Cannabis and cocaine users | Users of other drugs | -0.2101 | 0.3244 | -0.4869 | 0.0666 | FALSE | restlessness | FALSE |
| Cannabis users | Cocaine users | 0.0139 | 1 | -0.1906 | 0.2183 | FALSE | restlessness | FALSE |
| Cannabis users | Drug use history but no pandemic use | -0.0181 | 0.9998 | -0.1112 | 0.0749 | FALSE | restlessness | FALSE |
| Cannabis users | Extreme polydrug users | 0.3174 | 0.7334 | -0.2395 | 0.8743 | FALSE | restlessness | FALSE |
| Cannabis users | MDMA/ecstasy and cannabis users | -0.1788 | 0.8301 | -0.5242 | 0.1667 | FALSE | restlessness | FALSE |
| Cannabis users | Never used drugs | -0.0111 | 1 | -0.0971 | 0.0749 | FALSE | restlessness | FALSE |
| Cannabis users | Psychedelics and cannabis users | 0.0018 | 1 | -0.2364 | 0.2399 | FALSE | restlessness | FALSE |
| Cannabis users | Unknown/Unwilling to disclose | -0.0472 | 0.8301 | -0.1384 | 0.044 | FALSE | restlessness | FALSE |
| Cannabis users | Users of other drugs | -0.0196 | 1 | -0.2433 | 0.2041 | FALSE | restlessness | FALSE |
| Cocaine users | Drug use history but no pandemic use | -0.032 | 1 | -0.2233 | 0.1593 | FALSE | restlessness | FALSE |
| Cocaine users | Extreme polydrug users | 0.3035 | 0.8224 | -0.2779 | 0.8849 | FALSE | restlessness | FALSE |
| Cocaine users | MDMA/ecstasy and cannabis users | -0.1926 | 0.8544 | -0.5764 | 0.1912 | FALSE | restlessness | FALSE |
| Cocaine users | Never used drugs | -0.025 | 1 | -0.2129 | 0.163 | FALSE | restlessness | FALSE |
| Cocaine users | Psychedelics and cannabis users | -0.0121 | 1 | -0.303 | 0.2788 | FALSE | restlessness | FALSE |
| Cocaine users | Unknown/Unwilling to disclose | -0.061 | 0.9915 | -0.2514 | 0.1293 | FALSE | restlessness | FALSE |
| Cocaine users | Users of other drugs | -0.0335 | 1 | -0.3127 | 0.2458 | FALSE | restlessness | FALSE |
| Drug use history but no pandemic use | Extreme polydrug users | 0.3355 | 0.6537 | -0.2167 | 0.8877 | FALSE | restlessness | FALSE |
| Drug use history but no pandemic use | MDMA/ecstasy and cannabis users | -0.1606 | 0.891 | -0.4985 | 0.1772 | FALSE | restlessness | FALSE |
| Drug use history but no pandemic use | Never used drugs | 0.007 | 1 | -0.0397 | 0.0538 | FALSE | restlessness | FALSE |
| Drug use history but no pandemic use | Psychedelics and cannabis users | 0.0199 | 1 | -0.207 | 0.2468 | FALSE | restlessness | FALSE |
| Drug use history but no pandemic use | Unknown/Unwilling to disclose | -0.029 | 0.824 | -0.0848 | 0.0267 | FALSE | restlessness | FALSE |
| Drug use history but no pandemic use | Users of other drugs | -0.0015 | 1 | -0.2133 | 0.2103 | FALSE | restlessness | FALSE |
| Extreme polydrug users | MDMA/ecstasy and cannabis users | -0.4961 | 0.3049 | -1.1408 | 0.1485 | FALSE | restlessness | FALSE |
| Extreme polydrug users | Never used drugs | -0.3285 | 0.6786 | -0.8796 | 0.2225 | FALSE | restlessness | FALSE |
| Extreme polydrug users | Psychedelics and cannabis users | -0.3156 | 0.8066 | -0.9097 | 0.2785 | FALSE | restlessness | FALSE |
| Extreme polydrug users | Unknown/Unwilling to disclose | -0.3646 | 0.5345 | -0.9165 | 0.1873 | FALSE | restlessness | FALSE |
| Extreme polydrug users | Users of other drugs | -0.337 | 0.7279 | -0.9255 | 0.2515 | FALSE | restlessness | FALSE |
| MDMA/ecstasy and cannabis users | Never used drugs | 0.1676 | 0.8588 | -0.1683 | 0.5036 | FALSE | restlessness | FALSE |
| MDMA/ecstasy and cannabis users | Psychedelics and cannabis users | 0.1805 | 0.9219 | -0.2222 | 0.5833 | FALSE | restlessness | FALSE |
| MDMA/ecstasy and cannabis users | Unknown/Unwilling to disclose | 0.1316 | 0.967 | -0.2058 | 0.4689 | FALSE | restlessness | FALSE |
| MDMA/ecstasy and cannabis users | Users of other drugs | 0.1591 | 0.9589 | -0.2352 | 0.5535 | FALSE | restlessness | FALSE |
| Never used drugs | Psychedelics and cannabis users | 0.0129 | 1 | -0.2112 | 0.237 | FALSE | restlessness | FALSE |
| Never used drugs | Unknown/Unwilling to disclose | -0.0361 | 0.191 | -0.079 | 0.0069 | FALSE | restlessness | FALSE |
| Never used drugs | Users of other drugs | -0.0085 | 1 | -0.2173 | 0.2003 | FALSE | restlessness | FALSE |
| Psychedelics and cannabis users | Unknown/Unwilling to disclose | -0.0489 | 0.9996 | -0.2751 | 0.1772 | FALSE | restlessness | FALSE |
| Psychedelics and cannabis users | Users of other drugs | -0.0214 | 1 | -0.3262 | 0.2834 | FALSE | restlessness | FALSE |
| Unknown/Unwilling to disclose | Users of other drugs | 0.0276 | 1 | -0.1834 | 0.2385 | FALSE | restlessness | FALSE |
| Cannabis and cocaine users | Cannabis users | -0.107 | 0.8041 | -0.3079 | 0.0939 | FALSE | irritability | FALSE |
| Cannabis and cocaine users | Cocaine users | -0.0391 | 1 | -0.3004 | 0.2222 | FALSE | irritability | FALSE |
| Cannabis and cocaine users | Drug use history but no pandemic use | -0.0285 | 1 | -0.216 | 0.159 | FALSE | irritability | FALSE |
| Cannabis and cocaine users | Extreme polydrug users | 0.2532 | 0.9332 | -0.3268 | 0.8332 | FALSE | irritability | FALSE |
| Cannabis and cocaine users | MDMA/ecstasy and cannabis users | -0.0705 | 0.9999 | -0.4523 | 0.3113 | FALSE | irritability | FALSE |
| Cannabis and cocaine users | Never used drugs | -0.1012 | 0.7733 | -0.2853 | 0.0829 | FALSE | irritability | FALSE |
| Cannabis and cocaine users | Psychedelics and cannabis users | 0.1941 | 0.5063 | -0.0943 | 0.4825 | FALSE | irritability | FALSE |
| Cannabis and cocaine users | Unknown/Unwilling to disclose | -0.0977 | 0.8197 | -0.2843 | 0.0889 | FALSE | irritability | FALSE |
| Cannabis and cocaine users | Users of other drugs | 0.0187 | 1 | -0.2579 | 0.2954 | FALSE | irritability | FALSE |
| Cannabis users | Cocaine users | 0.068 | 0.9889 | -0.1364 | 0.2723 | FALSE | irritability | FALSE |
| Cannabis users | Drug use history but no pandemic use | 0.0785 | 0.1856 | -0.0145 | 0.1715 | FALSE | irritability | FALSE |
| Cannabis users | Extreme polydrug users | 0.3603 | 0.565 | -0.1964 | 0.9169 | FALSE | irritability | FALSE |
| Cannabis users | MDMA/ecstasy and cannabis users | 0.0365 | 1 | -0.3089 | 0.3818 | FALSE | irritability | FALSE |
| Cannabis users | Never used drugs | 0.0058 | 1 | -0.0801 | 0.0918 | FALSE | irritability | FALSE |
| Cannabis users | Psychedelics and cannabis users | 0.3011 | 0.0025 | 0.0631 | 0.5392 | TRUE | irritability | FALSE |
| Cannabis users | Unknown/Unwilling to disclose | 0.0093 | 1 | -0.0818 | 0.1005 | FALSE | irritability | FALSE |
| Cannabis users | Users of other drugs | 0.1257 | 0.749 | -0.0979 | 0.3494 | FALSE | irritability | FALSE |
| Cocaine users | Drug use history but no pandemic use | 0.0106 | 1 | -0.1806 | 0.2018 | FALSE | irritability | FALSE |
| Cocaine users | Extreme polydrug users | 0.2923 | 0.8529 | -0.2889 | 0.8735 | FALSE | irritability | FALSE |
| Cocaine users | MDMA/ecstasy and cannabis users | -0.0315 | 1 | -0.4151 | 0.3521 | FALSE | irritability | FALSE |
| Cocaine users | Never used drugs | -0.0621 | 0.9894 | -0.25 | 0.1258 | FALSE | irritability | FALSE |
| Cocaine users | Psychedelics and cannabis users | 0.2332 | 0.2487 | -0.0576 | 0.524 | FALSE | irritability | FALSE |
| Cocaine users | Unknown/Unwilling to disclose | -0.0586 | 0.9936 | -0.249 | 0.1317 | FALSE | irritability | FALSE |
| Cocaine users | Users of other drugs | 0.0578 | 0.9997 | -0.2214 | 0.3369 | FALSE | irritability | FALSE |
| Drug use history but no pandemic use | Extreme polydrug users | 0.2817 | 0.8413 | -0.2703 | 0.8337 | FALSE | irritability | FALSE |
| Drug use history but no pandemic use | MDMA/ecstasy and cannabis users | -0.0421 | 1 | -0.3798 | 0.2957 | FALSE | irritability | FALSE |
| Drug use history but no pandemic use | Never used drugs | -0.0727 | 0 | -0.1194 | -0.026 | TRUE | irritability | FALSE |
| Drug use history but no pandemic use | Psychedelics and cannabis users | 0.2226 | 0.0596 | -0.0042 | 0.4495 | FALSE | irritability | FALSE |
| Drug use history but no pandemic use | Unknown/Unwilling to disclose | -0.0692 | 0.0034 | -0.1249 | -0.0135 | TRUE | irritability | FALSE |
| Drug use history but no pandemic use | Users of other drugs | 0.0472 | 0.9995 | -0.1645 | 0.2589 | FALSE | irritability | FALSE |
| Extreme polydrug users | MDMA/ecstasy and cannabis users | -0.3238 | 0.8536 | -0.9682 | 0.3207 | FALSE | irritability | FALSE |
| Extreme polydrug users | Never used drugs | -0.3544 | 0.5735 | -0.9053 | 0.1964 | FALSE | irritability | FALSE |
| Extreme polydrug users | Psychedelics and cannabis users | -0.0591 | 1 | -0.653 | 0.5348 | FALSE | irritability | FALSE |
| Extreme polydrug users | Unknown/Unwilling to disclose | -0.3509 | 0.59 | -0.9026 | 0.2007 | FALSE | irritability | FALSE |
| Extreme polydrug users | Users of other drugs | -0.2345 | 0.962 | -0.8228 | 0.3537 | FALSE | irritability | FALSE |
| MDMA/ecstasy and cannabis users | Never used drugs | -0.0306 | 1 | -0.3665 | 0.3052 | FALSE | irritability | FALSE |
| MDMA/ecstasy and cannabis users | Psychedelics and cannabis users | 0.2647 | 0.5418 | -0.1379 | 0.6673 | FALSE | irritability | FALSE |
| MDMA/ecstasy and cannabis users | Unknown/Unwilling to disclose | -0.0272 | 1 | -0.3644 | 0.3101 | FALSE | irritability | FALSE |
| MDMA/ecstasy and cannabis users | Users of other drugs | 0.0893 | 0.9994 | -0.305 | 0.4835 | FALSE | irritability | FALSE |
| Never used drugs | Psychedelics and cannabis users | 0.2953 | 0.0013 | 0.0712 | 0.5194 | TRUE | irritability | FALSE |
| Never used drugs | Unknown/Unwilling to disclose | 0.0035 | 1 | -0.0394 | 0.0464 | FALSE | irritability | FALSE |
| Never used drugs | Users of other drugs | 0.1199 | 0.7242 | -0.0888 | 0.3286 | FALSE | irritability | FALSE |
| Psychedelics and cannabis users | Unknown/Unwilling to disclose | -0.2918 | 0.0018 | -0.5179 | -0.0657 | TRUE | irritability | FALSE |
| Psychedelics and cannabis users | Users of other drugs | -0.1754 | 0.7217 | -0.4801 | 0.1293 | FALSE | irritability | FALSE |
| Unknown/Unwilling to disclose | Users of other drugs | 0.1164 | 0.7686 | -0.0945 | 0.3273 | FALSE | irritability | FALSE |
| Cannabis and cocaine users | Cannabis users | 0.0141 | 1 | -0.1869 | 0.2151 | FALSE | negative premonition | FALSE |
| Cannabis and cocaine users | Cocaine users | 0.1357 | 0.8273 | -0.1257 | 0.3971 | FALSE | negative premonition | FALSE |
| Cannabis and cocaine users | Drug use history but no pandemic use | 0.0384 | 0.9997 | -0.1492 | 0.226 | FALSE | negative premonition | FALSE |
| Cannabis and cocaine users | Extreme polydrug users | 0.0805 | 1 | -0.4997 | 0.6607 | FALSE | negative premonition | FALSE |
| Cannabis and cocaine users | MDMA/ecstasy and cannabis users | -0.287 | 0.3393 | -0.669 | 0.0949 | FALSE | negative premonition | FALSE |
| Cannabis and cocaine users | Never used drugs | 0.0417 | 0.9994 | -0.1425 | 0.2258 | FALSE | negative premonition | FALSE |
| Cannabis and cocaine users | Psychedelics and cannabis users | 0.0483 | 1 | -0.2402 | 0.3368 | FALSE | negative premonition | FALSE |
| Cannabis and cocaine users | Unknown/Unwilling to disclose | 0.0351 | 0.9999 | -0.1516 | 0.2217 | FALSE | negative premonition | FALSE |
| Cannabis and cocaine users | Users of other drugs | 0.1514 | 0.7777 | -0.1253 | 0.4282 | FALSE | negative premonition | FALSE |
| Cannabis users | Cocaine users | 0.1216 | 0.6815 | -0.0828 | 0.326 | FALSE | negative premonition | FALSE |
| Cannabis users | Drug use history but no pandemic use | 0.0243 | 0.9982 | -0.0687 | 0.1174 | FALSE | negative premonition | FALSE |
| Cannabis users | Extreme polydrug users | 0.0665 | 1 | -0.4904 | 0.6233 | FALSE | negative premonition | FALSE |
| Cannabis users | MDMA/ecstasy and cannabis users | -0.3011 | 0.1512 | -0.6466 | 0.0444 | FALSE | negative premonition | FALSE |
| Cannabis users | Never used drugs | 0.0276 | 0.9915 | -0.0584 | 0.1136 | FALSE | negative premonition | FALSE |
| Cannabis users | Psychedelics and cannabis users | 0.0342 | 1 | -0.2039 | 0.2724 | FALSE | negative premonition | FALSE |
| Cannabis users | Unknown/Unwilling to disclose | 0.021 | 0.9993 | -0.0702 | 0.1122 | FALSE | negative premonition | FALSE |
| Cannabis users | Users of other drugs | 0.1374 | 0.6398 | -0.0864 | 0.3611 | FALSE | negative premonition | FALSE |
| Cocaine users | Drug use history but no pandemic use | -0.0973 | 0.8441 | -0.2886 | 0.094 | FALSE | negative premonition | FALSE |
| Cocaine users | Extreme polydrug users | -0.0551 | 1 | -0.6366 | 0.5263 | FALSE | negative premonition | FALSE |
| Cocaine users | MDMA/ecstasy and cannabis users | -0.4227 | 0.0177 | -0.8065 | -0.0389 | TRUE | negative premonition | FALSE |
| Cocaine users | Never used drugs | -0.094 | 0.8568 | -0.282 | 0.0939 | FALSE | negative premonition | FALSE |
| Cocaine users | Psychedelics and cannabis users | -0.0874 | 0.9947 | -0.3783 | 0.2036 | FALSE | negative premonition | FALSE |
| Cocaine users | Unknown/Unwilling to disclose | -0.1006 | 0.8113 | -0.291 | 0.0898 | FALSE | negative premonition | FALSE |
| Cocaine users | Users of other drugs | 0.0158 | 1 | -0.2635 | 0.295 | FALSE | negative premonition | FALSE |
| Drug use history but no pandemic use | Extreme polydrug users | 0.0421 | 1 | -0.51 | 0.5943 | FALSE | negative premonition | FALSE |
| Drug use history but no pandemic use | MDMA/ecstasy and cannabis users | -0.3254 | 0.0703 | -0.6633 | 0.0124 | FALSE | negative premonition | FALSE |
| Drug use history but no pandemic use | Never used drugs | 0.0033 | 1 | -0.0435 | 0.05 | FALSE | negative premonition | FALSE |
| Drug use history but no pandemic use | Psychedelics and cannabis users | 0.0099 | 1 | -0.217 | 0.2369 | FALSE | negative premonition | FALSE |
| Drug use history but no pandemic use | Unknown/Unwilling to disclose | -0.0033 | 1 | -0.0591 | 0.0524 | FALSE | negative premonition | FALSE |
| Drug use history but no pandemic use | Users of other drugs | 0.113 | 0.8022 | -0.0987 | 0.3248 | FALSE | negative premonition | FALSE |
| Extreme polydrug users | MDMA/ecstasy and cannabis users | -0.3676 | 0.733 | -1.0122 | 0.2771 | FALSE | negative premonition | FALSE |
| Extreme polydrug users | Never used drugs | -0.0389 | 1 | -0.5899 | 0.5122 | FALSE | negative premonition | FALSE |
| Extreme polydrug users | Psychedelics and cannabis users | -0.0322 | 1 | -0.6263 | 0.5619 | FALSE | negative premonition | FALSE |
| Extreme polydrug users | Unknown/Unwilling to disclose | -0.0455 | 1 | -0.5973 | 0.5064 | FALSE | negative premonition | FALSE |
| Extreme polydrug users | Users of other drugs | 0.0709 | 1 | -0.5176 | 0.6594 | FALSE | negative premonition | FALSE |
| MDMA/ecstasy and cannabis users | Never used drugs | 0.3287 | 0.0613 | -0.0073 | 0.6647 | FALSE | negative premonition | FALSE |
| MDMA/ecstasy and cannabis users | Psychedelics and cannabis users | 0.3353 | 0.2015 | -0.0674 | 0.7381 | FALSE | negative premonition | FALSE |
| MDMA/ecstasy and cannabis users | Unknown/Unwilling to disclose | 0.3221 | 0.0759 | -0.0153 | 0.6594 | FALSE | negative premonition | FALSE |
| MDMA/ecstasy and cannabis users | Users of other drugs | 0.4385 | 0.0158 | 0.0441 | 0.8328 | TRUE | negative premonition | FALSE |
| Never used drugs | Psychedelics and cannabis users | 0.0066 | 1 | -0.2175 | 0.2308 | FALSE | negative premonition | FALSE |
| Never used drugs | Unknown/Unwilling to disclose | -0.0066 | 1 | -0.0495 | 0.0363 | FALSE | negative premonition | FALSE |
| Never used drugs | Users of other drugs | 0.1098 | 0.8161 | -0.099 | 0.3185 | FALSE | negative premonition | FALSE |
| Psychedelics and cannabis users | Unknown/Unwilling to disclose | -0.0133 | 1 | -0.2394 | 0.2129 | FALSE | negative premonition | FALSE |
| Psychedelics and cannabis users | Users of other drugs | 0.1031 | 0.9875 | -0.2017 | 0.4079 | FALSE | negative premonition | FALSE |
| Unknown/Unwilling to disclose | Users of other drugs | 0.1164 | 0.7694 | -0.0946 | 0.3273 | FALSE | negative premonition | FALSE |
| Cannabis and cocaine users | Cannabis users | -0.0234 | 1 | -0.2242 | 0.1775 | FALSE | apathy | FALSE |
| Cannabis and cocaine users | Cocaine users | -0.0973 | 0.9756 | -0.3585 | 0.1639 | FALSE | apathy | FALSE |
| Cannabis and cocaine users | Drug use history but no pandemic use | -0.0969 | 0.8306 | -0.2844 | 0.0905 | FALSE | apathy | FALSE |
| Cannabis and cocaine users | Extreme polydrug users | 0.5487 | 0.0817 | -0.0311 | 1.1285 | FALSE | apathy | FALSE |
| Cannabis and cocaine users | MDMA/ecstasy and cannabis users | -0.2376 | 0.6205 | -0.6193 | 0.1441 | FALSE | apathy | FALSE |
| Cannabis and cocaine users | Never used drugs | -0.1381 | 0.3414 | -0.3222 | 0.0459 | FALSE | apathy | FALSE |
| Cannabis and cocaine users | Psychedelics and cannabis users | 0.1407 | 0.8742 | -0.1476 | 0.4291 | FALSE | apathy | FALSE |
| Cannabis and cocaine users | Unknown/Unwilling to disclose | -0.1729 | 0.0967 | -0.3594 | 0.0136 | FALSE | apathy | FALSE |
| Cannabis and cocaine users | Users of other drugs | 0.0818 | 0.9953 | -0.1947 | 0.3584 | FALSE | apathy | FALSE |
| Cannabis users | Cocaine users | -0.0739 | 0.98 | -0.2782 | 0.1304 | FALSE | apathy | FALSE |
| Cannabis users | Drug use history but no pandemic use | -0.0735 | 0.267 | -0.1665 | 0.0194 | FALSE | apathy | FALSE |
| Cannabis users | Extreme polydrug users | 0.5721 | 0.0381 | 0.0156 | 1.1286 | TRUE | apathy | FALSE |
| Cannabis users | MDMA/ecstasy and cannabis users | -0.2142 | 0.625 | -0.5595 | 0.131 | FALSE | apathy | FALSE |
| Cannabis users | Never used drugs | -0.1147 | 0.001 | -0.2006 | -0.0288 | TRUE | apathy | FALSE |
| Cannabis users | Psychedelics and cannabis users | 0.1641 | 0.4692 | -0.0738 | 0.4021 | FALSE | apathy | FALSE |
| Cannabis users | Unknown/Unwilling to disclose | -0.1495 | 0 | -0.2406 | -0.0584 | TRUE | apathy | FALSE |
| Cannabis users | Users of other drugs | 0.1052 | 0.8969 | -0.1184 | 0.3288 | FALSE | apathy | FALSE |
| Cocaine users | Drug use history but no pandemic use | 0.0004 | 1 | -0.1907 | 0.1915 | FALSE | apathy | FALSE |
| Cocaine users | Extreme polydrug users | 0.646 | 0.0158 | 0.065 | 1.2271 | TRUE | apathy | FALSE |
| Cocaine users | MDMA/ecstasy and cannabis users | -0.1403 | 0.9785 | -0.5238 | 0.2432 | FALSE | apathy | FALSE |
| Cocaine users | Never used drugs | -0.0408 | 0.9996 | -0.2286 | 0.147 | FALSE | apathy | FALSE |
| Cocaine users | Psychedelics and cannabis users | 0.2381 | 0.2218 | -0.0527 | 0.5288 | FALSE | apathy | FALSE |
| Cocaine users | Unknown/Unwilling to disclose | -0.0756 | 0.9629 | -0.2658 | 0.1147 | FALSE | apathy | FALSE |
| Cocaine users | Users of other drugs | 0.1792 | 0.5766 | -0.0999 | 0.4582 | FALSE | apathy | FALSE |
| Drug use history but no pandemic use | Extreme polydrug users | 0.6456 | 0.0082 | 0.0938 | 1.1974 | TRUE | apathy | FALSE |
| Drug use history but no pandemic use | MDMA/ecstasy and cannabis users | -0.1407 | 0.9497 | -0.4783 | 0.1969 | FALSE | apathy | FALSE |
| Drug use history but no pandemic use | Never used drugs | -0.0412 | 0.1402 | -0.0879 | 0.0055 | FALSE | apathy | FALSE |
| Drug use history but no pandemic use | Psychedelics and cannabis users | 0.2377 | 0.0311 | 0.0109 | 0.4645 | TRUE | apathy | FALSE |
| Drug use history but no pandemic use | Unknown/Unwilling to disclose | -0.076 | 0.0007 | -0.1317 | -0.0203 | TRUE | apathy | FALSE |
| Drug use history but no pandemic use | Users of other drugs | 0.1788 | 0.1848 | -0.0329 | 0.3904 | FALSE | apathy | FALSE |
| Extreme polydrug users | MDMA/ecstasy and cannabis users | -0.7863 | 0.0044 | -1.4306 | -0.1421 | TRUE | apathy | FALSE |
| Extreme polydrug users | Never used drugs | -0.6868 | 0.0032 | -1.2375 | -0.1361 | TRUE | apathy | FALSE |
| Extreme polydrug users | Psychedelics and cannabis users | -0.408 | 0.4748 | -1.0017 | 0.1857 | FALSE | apathy | FALSE |
| Extreme polydrug users | Unknown/Unwilling to disclose | -0.7216 | 0.0014 | -1.2731 | -0.1701 | TRUE | apathy | FALSE |
| Extreme polydrug users | Users of other drugs | -0.4669 | 0.2619 | -1.0549 | 0.1212 | FALSE | apathy | FALSE |
| MDMA/ecstasy and cannabis users | Never used drugs | 0.0995 | 0.9952 | -0.2362 | 0.4353 | FALSE | apathy | FALSE |
| MDMA/ecstasy and cannabis users | Psychedelics and cannabis users | 0.3784 | 0.0863 | -0.0241 | 0.7808 | FALSE | apathy | FALSE |
| MDMA/ecstasy and cannabis users | Unknown/Unwilling to disclose | 0.0647 | 0.9999 | -0.2724 | 0.4018 | FALSE | apathy | FALSE |
| MDMA/ecstasy and cannabis users | Users of other drugs | 0.3195 | 0.2346 | -0.0746 | 0.7136 | FALSE | apathy | FALSE |
| Never used drugs | Psychedelics and cannabis users | 0.2789 | 0.0033 | 0.0549 | 0.5028 | TRUE | apathy | FALSE |
| Never used drugs | Unknown/Unwilling to disclose | -0.0348 | 0.2336 | -0.0777 | 0.0081 | FALSE | apathy | FALSE |
| Never used drugs | Users of other drugs | 0.22 | 0.0292 | 0.0113 | 0.4286 | TRUE | apathy | FALSE |
| Psychedelics and cannabis users | Unknown/Unwilling to disclose | -0.3136 | 0.0005 | -0.5397 | -0.0876 | TRUE | apathy | FALSE |
| Psychedelics and cannabis users | Users of other drugs | -0.0589 | 0.9998 | -0.3635 | 0.2457 | FALSE | apathy | FALSE |
| Unknown/Unwilling to disclose | Users of other drugs | 0.2547 | 0.0052 | 0.0439 | 0.4656 | TRUE | apathy | FALSE |
| Cannabis and cocaine users | Cannabis users | -0.1361 | 0.4959 | -0.337 | 0.0647 | FALSE | depression | FALSE |
| Cannabis and cocaine users | Cocaine users | -0.0953 | 0.9789 | -0.3565 | 0.166 | FALSE | depression | FALSE |
| Cannabis and cocaine users | Drug use history but no pandemic use | -0.1902 | 0.0435 | -0.3777 | -0.0027 | TRUE | depression | FALSE |
| Cannabis and cocaine users | Extreme polydrug users | 0.1169 | 0.9998 | -0.463 | 0.6968 | FALSE | depression | FALSE |
| Cannabis and cocaine users | MDMA/ecstasy and cannabis users | -0.3745 | 0.0597 | -0.7563 | 0.0072 | FALSE | depression | FALSE |
| Cannabis and cocaine users | Never used drugs | -0.2231 | 0.0049 | -0.4072 | -0.039 | TRUE | depression | FALSE |
| Cannabis and cocaine users | Psychedelics and cannabis users | -0.0364 | 1 | -0.3248 | 0.252 | FALSE | depression | FALSE |
| Cannabis and cocaine users | Unknown/Unwilling to disclose | -0.2517 | 0.0008 | -0.4383 | -0.0651 | TRUE | depression | FALSE |
| Cannabis and cocaine users | Users of other drugs | -0.0097 | 1 | -0.2863 | 0.2669 | FALSE | depression | FALSE |
| Cannabis users | Cocaine users | 0.0409 | 0.9998 | -0.1635 | 0.2452 | FALSE | depression | FALSE |
| Cannabis users | Drug use history but no pandemic use | -0.0541 | 0.7104 | -0.1471 | 0.039 | FALSE | depression | FALSE |
| Cannabis users | Extreme polydrug users | 0.2531 | 0.9152 | -0.3035 | 0.8097 | FALSE | depression | FALSE |
| Cannabis users | MDMA/ecstasy and cannabis users | -0.2384 | 0.4677 | -0.5837 | 0.1069 | FALSE | depression | FALSE |
| Cannabis users | Never used drugs | -0.087 | 0.0445 | -0.1729 | -0.001 | TRUE | depression | FALSE |
| Cannabis users | Psychedelics and cannabis users | 0.0997 | 0.9479 | -0.1383 | 0.3378 | FALSE | depression | FALSE |
| Cannabis users | Unknown/Unwilling to disclose | -0.1155 | 0.0024 | -0.2067 | -0.0244 | TRUE | depression | FALSE |
| Cannabis users | Users of other drugs | 0.1264 | 0.7426 | -0.0972 | 0.3501 | FALSE | depression | FALSE |
| Cocaine users | Drug use history but no pandemic use | -0.0949 | 0.8624 | -0.2861 | 0.0963 | FALSE | depression | FALSE |
| Cocaine users | Extreme polydrug users | 0.2122 | 0.9787 | -0.3689 | 0.7933 | FALSE | depression | FALSE |
| Cocaine users | MDMA/ecstasy and cannabis users | -0.2792 | 0.3867 | -0.6628 | 0.1043 | FALSE | depression | FALSE |
| Cocaine users | Never used drugs | -0.1278 | 0.4896 | -0.3157 | 0.06 | FALSE | depression | FALSE |
| Cocaine users | Psychedelics and cannabis users | 0.0589 | 0.9998 | -0.2319 | 0.3497 | FALSE | depression | FALSE |
| Cocaine users | Unknown/Unwilling to disclose | -0.1564 | 0.2171 | -0.3467 | 0.0339 | FALSE | depression | FALSE |
| Cocaine users | Users of other drugs | 0.0856 | 0.9939 | -0.1935 | 0.3647 | FALSE | depression | FALSE |
| Drug use history but no pandemic use | Extreme polydrug users | 0.3071 | 0.7601 | -0.2448 | 0.859 | FALSE | depression | FALSE |
| Drug use history but no pandemic use | MDMA/ecstasy and cannabis users | -0.1843 | 0.7802 | -0.522 | 0.1533 | FALSE | depression | FALSE |
| Drug use history but no pandemic use | Never used drugs | -0.0329 | 0.4365 | -0.0797 | 0.0138 | FALSE | depression | FALSE |
| Drug use history but no pandemic use | Psychedelics and cannabis users | 0.1538 | 0.4952 | -0.073 | 0.3806 | FALSE | depression | FALSE |
| Drug use history but no pandemic use | Unknown/Unwilling to disclose | -0.0615 | 0.0173 | -0.1172 | -0.0058 | TRUE | depression | FALSE |
| Drug use history but no pandemic use | Users of other drugs | 0.1805 | 0.1744 | -0.0312 | 0.3922 | FALSE | depression | FALSE |
| Extreme polydrug users | MDMA/ecstasy and cannabis users | -0.4914 | 0.3178 | -1.1358 | 0.1529 | FALSE | depression | FALSE |
| Extreme polydrug users | Never used drugs | -0.34 | 0.6321 | -0.8908 | 0.2107 | FALSE | depression | FALSE |
| Extreme polydrug users | Psychedelics and cannabis users | -0.1533 | 0.9984 | -0.7471 | 0.4405 | FALSE | depression | FALSE |
| Extreme polydrug users | Unknown/Unwilling to disclose | -0.3686 | 0.5172 | -0.9202 | 0.183 | FALSE | depression | FALSE |
| Extreme polydrug users | Users of other drugs | -0.1266 | 0.9996 | -0.7148 | 0.4616 | FALSE | depression | FALSE |
| MDMA/ecstasy and cannabis users | Never used drugs | 0.1514 | 0.9192 | -0.1844 | 0.4872 | FALSE | depression | FALSE |
| MDMA/ecstasy and cannabis users | Psychedelics and cannabis users | 0.3381 | 0.1912 | -0.0644 | 0.7407 | FALSE | depression | FALSE |
| MDMA/ecstasy and cannabis users | Unknown/Unwilling to disclose | 0.1228 | 0.9791 | -0.2143 | 0.46 | FALSE | depression | FALSE |
| MDMA/ecstasy and cannabis users | Users of other drugs | 0.3648 | 0.0979 | -0.0294 | 0.759 | FALSE | depression | FALSE |
| Never used drugs | Psychedelics and cannabis users | 0.1867 | 0.2003 | -0.0373 | 0.4107 | FALSE | depression | FALSE |
| Never used drugs | Unknown/Unwilling to disclose | -0.0286 | 0.5223 | -0.0715 | 0.0143 | FALSE | depression | FALSE |
| Never used drugs | Users of other drugs | 0.2134 | 0.0401 | 0.0047 | 0.4221 | TRUE | depression | FALSE |
| Psychedelics and cannabis users | Unknown/Unwilling to disclose | -0.2153 | 0.0775 | -0.4414 | 0.0108 | FALSE | depression | FALSE |
| Psychedelics and cannabis users | Users of other drugs | 0.0267 | 1 | -0.278 | 0.3313 | FALSE | depression | FALSE |
| Unknown/Unwilling to disclose | Users of other drugs | 0.242 | 0.0106 | 0.0311 | 0.4528 | TRUE | depression | FALSE |
| Cannabis and cocaine users | Cannabis users | 0.0179 | 1 | -0.183 | 0.2188 | FALSE | tiredness | FALSE |
| Cannabis and cocaine users | Cocaine users | 0.0767 | 0.9956 | -0.1846 | 0.338 | FALSE | tiredness | FALSE |
| Cannabis and cocaine users | Drug use history but no pandemic use | 0.0638 | 0.987 | -0.1237 | 0.2513 | FALSE | tiredness | FALSE |
| Cannabis and cocaine users | Extreme polydrug users | 0.4425 | 0.3172 | -0.1374 | 1.0225 | FALSE | tiredness | FALSE |
| Cannabis and cocaine users | MDMA/ecstasy and cannabis users | -0.2289 | 0.6715 | -0.6107 | 0.1529 | FALSE | tiredness | FALSE |
| Cannabis and cocaine users | Never used drugs | -0.0035 | 1 | -0.1876 | 0.1807 | FALSE | tiredness | FALSE |
| Cannabis and cocaine users | Psychedelics and cannabis users | 0.0129 | 1 | -0.2755 | 0.3013 | FALSE | tiredness | FALSE |
| Cannabis and cocaine users | Unknown/Unwilling to disclose | -0.0343 | 0.9999 | -0.2209 | 0.1523 | FALSE | tiredness | FALSE |
| Cannabis and cocaine users | Users of other drugs | 0.1491 | 0.7931 | -0.1276 | 0.4257 | FALSE | tiredness | FALSE |
| Cannabis users | Cocaine users | 0.0588 | 0.9962 | -0.1456 | 0.2632 | FALSE | tiredness | FALSE |
| Cannabis users | Drug use history but no pandemic use | 0.0459 | 0.8665 | -0.0471 | 0.1389 | FALSE | tiredness | FALSE |
| Cannabis users | Extreme polydrug users | 0.4247 | 0.3174 | -0.132 | 0.9813 | FALSE | tiredness | FALSE |
| Cannabis users | MDMA/ecstasy and cannabis users | -0.2467 | 0.4151 | -0.5921 | 0.0986 | FALSE | tiredness | FALSE |
| Cannabis users | Never used drugs | -0.0213 | 0.9988 | -0.1073 | 0.0646 | FALSE | tiredness | FALSE |
| Cannabis users | Psychedelics and cannabis users | -0.005 | 1 | -0.243 | 0.2331 | FALSE | tiredness | FALSE |
| Cannabis users | Unknown/Unwilling to disclose | -0.0522 | 0.7279 | -0.1434 | 0.039 | FALSE | tiredness | FALSE |
| Cannabis users | Users of other drugs | 0.1312 | 0.6991 | -0.0925 | 0.3548 | FALSE | tiredness | FALSE |
| Cocaine users | Drug use history but no pandemic use | -0.0129 | 1 | -0.2041 | 0.1783 | FALSE | tiredness | FALSE |
| Cocaine users | Extreme polydrug users | 0.3659 | 0.605 | -0.2153 | 0.9471 | FALSE | tiredness | FALSE |
| Cocaine users | MDMA/ecstasy and cannabis users | -0.3056 | 0.2575 | -0.6892 | 0.0781 | FALSE | tiredness | FALSE |
| Cocaine users | Never used drugs | -0.0801 | 0.9419 | -0.268 | 0.1077 | FALSE | tiredness | FALSE |
| Cocaine users | Psychedelics and cannabis users | -0.0638 | 0.9996 | -0.3546 | 0.227 | FALSE | tiredness | FALSE |
| Cocaine users | Unknown/Unwilling to disclose | -0.111 | 0.7059 | -0.3013 | 0.0793 | FALSE | tiredness | FALSE |
| Cocaine users | Users of other drugs | 0.0724 | 0.9983 | -0.2068 | 0.3515 | FALSE | tiredness | FALSE |
| Drug use history but no pandemic use | Extreme polydrug users | 0.3787 | 0.477 | -0.1732 | 0.9307 | FALSE | tiredness | FALSE |
| Drug use history but no pandemic use | MDMA/ecstasy and cannabis users | -0.2927 | 0.1571 | -0.6304 | 0.0451 | FALSE | tiredness | FALSE |
| Drug use history but no pandemic use | Never used drugs | -0.0673 | 0.0002 | -0.114 | -0.0205 | TRUE | tiredness | FALSE |
| Drug use history but no pandemic use | Psychedelics and cannabis users | -0.0509 | 0.9995 | -0.2778 | 0.1759 | FALSE | tiredness | FALSE |
| Drug use history but no pandemic use | Unknown/Unwilling to disclose | -0.0981 | 0 | -0.1538 | -0.0424 | TRUE | tiredness | FALSE |
| Drug use history but no pandemic use | Users of other drugs | 0.0853 | 0.9594 | -0.1264 | 0.297 | FALSE | tiredness | FALSE |
| Extreme polydrug users | MDMA/ecstasy and cannabis users | -0.6714 | 0.0331 | -1.3158 | -0.027 | TRUE | tiredness | FALSE |
| Extreme polydrug users | Never used drugs | -0.446 | 0.2361 | -0.9968 | 0.1048 | FALSE | tiredness | FALSE |
| Extreme polydrug users | Psychedelics and cannabis users | -0.4297 | 0.3961 | -1.0235 | 0.1642 | FALSE | tiredness | FALSE |
| Extreme polydrug users | Unknown/Unwilling to disclose | -0.4769 | 0.1597 | -1.0285 | 0.0748 | FALSE | tiredness | FALSE |
| Extreme polydrug users | Users of other drugs | -0.2935 | 0.8589 | -0.8817 | 0.2948 | FALSE | tiredness | FALSE |
| MDMA/ecstasy and cannabis users | Never used drugs | 0.2254 | 0.5106 | -0.1104 | 0.5613 | FALSE | tiredness | FALSE |
| MDMA/ecstasy and cannabis users | Psychedelics and cannabis users | 0.2418 | 0.6692 | -0.1608 | 0.6443 | FALSE | tiredness | FALSE |
| MDMA/ecstasy and cannabis users | Unknown/Unwilling to disclose | 0.1945 | 0.7192 | -0.1427 | 0.5318 | FALSE | tiredness | FALSE |
| MDMA/ecstasy and cannabis users | Users of other drugs | 0.3779 | 0.0733 | -0.0163 | 0.7722 | FALSE | tiredness | FALSE |
| Never used drugs | Psychedelics and cannabis users | 0.0163 | 1 | -0.2077 | 0.2404 | FALSE | tiredness | FALSE |
| Never used drugs | Unknown/Unwilling to disclose | -0.0309 | 0.4047 | -0.0738 | 0.012 | FALSE | tiredness | FALSE |
| Never used drugs | Users of other drugs | 0.1525 | 0.3808 | -0.0562 | 0.3612 | FALSE | tiredness | FALSE |
| Psychedelics and cannabis users | Unknown/Unwilling to disclose | -0.0472 | 0.9997 | -0.2733 | 0.1789 | FALSE | tiredness | FALSE |
| Psychedelics and cannabis users | Users of other drugs | 0.1362 | 0.9233 | -0.1685 | 0.4409 | FALSE | tiredness | FALSE |
| Unknown/Unwilling to disclose | Users of other drugs | 0.1834 | 0.1536 | -0.0275 | 0.3943 | FALSE | tiredness | FALSE |
| Cannabis and cocaine users | Cannabis users | -0.0877 | 0.9331 | -0.2886 | 0.1132 | FALSE | concentration problems | FALSE |
| Cannabis and cocaine users | Cocaine users | 0.0015 | 1 | -0.2598 | 0.2628 | FALSE | concentration problems | FALSE |
| Cannabis and cocaine users | Drug use history but no pandemic use | -0.0494 | 0.9981 | -0.2369 | 0.1381 | FALSE | concentration problems | FALSE |
| Cannabis and cocaine users | Extreme polydrug users | 0.4974 | 0.1682 | -0.0826 | 1.0774 | FALSE | concentration problems | FALSE |
| Cannabis and cocaine users | MDMA/ecstasy and cannabis users | -0.054 | 1 | -0.4358 | 0.3278 | FALSE | concentration problems | FALSE |
| Cannabis and cocaine users | Never used drugs | -0.1201 | 0.5535 | -0.3042 | 0.064 | FALSE | concentration problems | FALSE |
| Cannabis and cocaine users | Psychedelics and cannabis users | 0.0564 | 0.9998 | -0.232 | 0.3448 | FALSE | concentration problems | FALSE |
| Cannabis and cocaine users | Unknown/Unwilling to disclose | -0.125 | 0.514 | -0.3116 | 0.0616 | FALSE | concentration problems | FALSE |
| Cannabis and cocaine users | Users of other drugs | 0.0373 | 1 | -0.2393 | 0.314 | FALSE | concentration problems | FALSE |
| Cannabis users | Cocaine users | 0.0892 | 0.9331 | -0.1151 | 0.2936 | FALSE | concentration problems | FALSE |
| Cannabis users | Drug use history but no pandemic use | 0.0384 | 0.9529 | -0.0547 | 0.1314 | FALSE | concentration problems | FALSE |
| Cannabis users | Extreme polydrug users | 0.5851 | 0.0302 | 0.0284 | 1.1418 | TRUE | concentration problems | FALSE |
| Cannabis users | MDMA/ecstasy and cannabis users | 0.0338 | 1 | -0.3116 | 0.3791 | FALSE | concentration problems | FALSE |
| Cannabis users | Never used drugs | -0.0324 | 0.9739 | -0.1183 | 0.0536 | FALSE | concentration problems | FALSE |
| Cannabis users | Psychedelics and cannabis users | 0.1442 | 0.6582 | -0.0939 | 0.3822 | FALSE | concentration problems | FALSE |
| Cannabis users | Unknown/Unwilling to disclose | -0.0372 | 0.9556 | -0.1284 | 0.0539 | FALSE | concentration problems | FALSE |
| Cannabis users | Users of other drugs | 0.125 | 0.755 | -0.0986 | 0.3487 | FALSE | concentration problems | FALSE |
| Cocaine users | Drug use history but no pandemic use | -0.0509 | 0.9979 | -0.2421 | 0.1403 | FALSE | concentration problems | FALSE |
| Cocaine users | Extreme polydrug users | 0.4959 | 0.1738 | -0.0853 | 1.077 | FALSE | concentration problems | FALSE |
| Cocaine users | MDMA/ecstasy and cannabis users | -0.0555 | 1 | -0.4391 | 0.3281 | FALSE | concentration problems | FALSE |
| Cocaine users | Never used drugs | -0.1216 | 0.5649 | -0.3095 | 0.0663 | FALSE | concentration problems | FALSE |
| Cocaine users | Psychedelics and cannabis users | 0.0549 | 0.9999 | -0.2359 | 0.3457 | FALSE | concentration problems | FALSE |
| Cocaine users | Unknown/Unwilling to disclose | -0.1265 | 0.5256 | -0.3168 | 0.0638 | FALSE | concentration problems | FALSE |
| Cocaine users | Users of other drugs | 0.0358 | 1 | -0.2433 | 0.315 | FALSE | concentration problems | FALSE |
| Drug use history but no pandemic use | Extreme polydrug users | 0.5467 | 0.0547 | -0.0052 | 1.0987 | FALSE | concentration problems | FALSE |
| Drug use history but no pandemic use | MDMA/ecstasy and cannabis users | -0.0046 | 1 | -0.3423 | 0.3331 | FALSE | concentration problems | FALSE |
| Drug use history but no pandemic use | Never used drugs | -0.0707 | 0.0001 | -0.1174 | -0.024 | TRUE | concentration problems | FALSE |
| Drug use history but no pandemic use | Psychedelics and cannabis users | 0.1058 | 0.9021 | -0.1211 | 0.3326 | FALSE | concentration problems | FALSE |
| Drug use history but no pandemic use | Unknown/Unwilling to disclose | -0.0756 | 0.0007 | -0.1313 | -0.0199 | TRUE | concentration problems | FALSE |
| Drug use history but no pandemic use | Users of other drugs | 0.0867 | 0.9549 | -0.125 | 0.2984 | FALSE | concentration problems | FALSE |
| Extreme polydrug users | MDMA/ecstasy and cannabis users | -0.5513 | 0.1707 | -1.1958 | 0.0931 | FALSE | concentration problems | FALSE |
| Extreme polydrug users | Never used drugs | -0.6174 | 0.0143 | -1.1683 | -0.0666 | TRUE | concentration problems | FALSE |
| Extreme polydrug users | Psychedelics and cannabis users | -0.4409 | 0.3571 | -1.0348 | 0.1529 | FALSE | concentration problems | FALSE |
| Extreme polydrug users | Unknown/Unwilling to disclose | -0.6223 | 0.0132 | -1.174 | -0.0707 | TRUE | concentration problems | FALSE |
| Extreme polydrug users | Users of other drugs | -0.46 | 0.2824 | -1.0483 | 0.1282 | FALSE | concentration problems | FALSE |
| MDMA/ecstasy and cannabis users | Never used drugs | -0.0661 | 0.9998 | -0.402 | 0.2697 | FALSE | concentration problems | FALSE |
| MDMA/ecstasy and cannabis users | Psychedelics and cannabis users | 0.1104 | 0.9974 | -0.2922 | 0.513 | FALSE | concentration problems | FALSE |
| MDMA/ecstasy and cannabis users | Unknown/Unwilling to disclose | -0.071 | 0.9997 | -0.4082 | 0.2662 | FALSE | concentration problems | FALSE |
| MDMA/ecstasy and cannabis users | Users of other drugs | 0.0913 | 0.9993 | -0.303 | 0.4855 | FALSE | concentration problems | FALSE |
| Never used drugs | Psychedelics and cannabis users | 0.1765 | 0.2723 | -0.0475 | 0.4006 | FALSE | concentration problems | FALSE |
| Never used drugs | Unknown/Unwilling to disclose | -0.0049 | 1 | -0.0478 | 0.038 | FALSE | concentration problems | FALSE |
| Never used drugs | Users of other drugs | 0.1574 | 0.334 | -0.0513 | 0.3661 | FALSE | concentration problems | FALSE |
| Psychedelics and cannabis users | Unknown/Unwilling to disclose | -0.1814 | 0.248 | -0.4075 | 0.0447 | FALSE | concentration problems | FALSE |
| Psychedelics and cannabis users | Users of other drugs | -0.0191 | 1 | -0.3238 | 0.2856 | FALSE | concentration problems | FALSE |
| Unknown/Unwilling to disclose | Users of other drugs | 0.1623 | 0.3051 | -0.0486 | 0.3732 | FALSE | concentration problems | FALSE |
| Cannabis and cocaine users | Cannabis users | 0.0612 | 0.9942 | -0.1398 | 0.2621 | FALSE | insomnia | FALSE |
| Cannabis and cocaine users | Cocaine users | 0.0334 | 1 | -0.228 | 0.2947 | FALSE | insomnia | FALSE |
| Cannabis and cocaine users | Drug use history but no pandemic use | 0.0642 | 0.9864 | -0.1234 | 0.2518 | FALSE | insomnia | FALSE |
| Cannabis and cocaine users | Extreme polydrug users | 0.3783 | 0.5537 | -0.2018 | 0.9585 | FALSE | insomnia | FALSE |
| Cannabis and cocaine users | MDMA/ecstasy and cannabis users | -0.1746 | 0.9126 | -0.5565 | 0.2073 | FALSE | insomnia | FALSE |
| Cannabis and cocaine users | Never used drugs | 0.0144 | 1 | -0.1698 | 0.1985 | FALSE | insomnia | FALSE |
| Cannabis and cocaine users | Psychedelics and cannabis users | -0.0175 | 1 | -0.3059 | 0.271 | FALSE | insomnia | FALSE |
| Cannabis and cocaine users | Unknown/Unwilling to disclose | -0.0247 | 1 | -0.2114 | 0.1619 | FALSE | insomnia | FALSE |
| Cannabis and cocaine users | Users of other drugs | 0.141 | 0.8425 | -0.1357 | 0.4177 | FALSE | insomnia | FALSE |
| Cannabis users | Cocaine users | -0.0278 | 1 | -0.2322 | 0.1766 | FALSE | insomnia | FALSE |
| Cannabis users | Drug use history but no pandemic use | 0.003 | 1 | -0.09 | 0.0961 | FALSE | insomnia | FALSE |
| Cannabis users | Extreme polydrug users | 0.3172 | 0.7341 | -0.2396 | 0.874 | FALSE | insomnia | FALSE |
| Cannabis users | MDMA/ecstasy and cannabis users | -0.2357 | 0.4853 | -0.5812 | 0.1097 | FALSE | insomnia | FALSE |
| Cannabis users | Never used drugs | -0.0468 | 0.7828 | -0.1328 | 0.0392 | FALSE | insomnia | FALSE |
| Cannabis users | Psychedelics and cannabis users | -0.0786 | 0.9895 | -0.3167 | 0.1595 | FALSE | insomnia | FALSE |
| Cannabis users | Unknown/Unwilling to disclose | -0.0859 | 0.085 | -0.1771 | 0.0053 | FALSE | insomnia | FALSE |
| Cannabis users | Users of other drugs | 0.0798 | 0.9818 | -0.1439 | 0.3036 | FALSE | insomnia | FALSE |
| Cocaine users | Drug use history but no pandemic use | 0.0309 | 1 | -0.1604 | 0.2221 | FALSE | insomnia | FALSE |
| Cocaine users | Extreme polydrug users | 0.345 | 0.6845 | -0.2364 | 0.9263 | FALSE | insomnia | FALSE |
| Cocaine users | MDMA/ecstasy and cannabis users | -0.2079 | 0.7876 | -0.5916 | 0.1758 | FALSE | insomnia | FALSE |
| Cocaine users | Never used drugs | -0.019 | 1 | -0.2069 | 0.1689 | FALSE | insomnia | FALSE |
| Cocaine users | Psychedelics and cannabis users | -0.0508 | 0.9999 | -0.3417 | 0.2401 | FALSE | insomnia | FALSE |
| Cocaine users | Unknown/Unwilling to disclose | -0.0581 | 0.9941 | -0.2484 | 0.1323 | FALSE | insomnia | FALSE |
| Cocaine users | Users of other drugs | 0.1077 | 0.9694 | -0.1716 | 0.3869 | FALSE | insomnia | FALSE |
| Drug use history but no pandemic use | Extreme polydrug users | 0.3141 | 0.7354 | -0.238 | 0.8663 | FALSE | insomnia | FALSE |
| Drug use history but no pandemic use | MDMA/ecstasy and cannabis users | -0.2388 | 0.4316 | -0.5766 | 0.099 | FALSE | insomnia | FALSE |
| Drug use history but no pandemic use | Never used drugs | -0.0498 | 0.0258 | -0.0966 | -0.0031 | TRUE | insomnia | FALSE |
| Drug use history but no pandemic use | Psychedelics and cannabis users | -0.0817 | 0.9807 | -0.3086 | 0.1452 | FALSE | insomnia | FALSE |
| Drug use history but no pandemic use | Unknown/Unwilling to disclose | -0.0889 | 0 | -0.1447 | -0.0332 | TRUE | insomnia | FALSE |
| Drug use history but no pandemic use | Users of other drugs | 0.0768 | 0.9797 | -0.1349 | 0.2886 | FALSE | insomnia | FALSE |
| Extreme polydrug users | MDMA/ecstasy and cannabis users | -0.5529 | 0.1679 | -1.1975 | 0.0917 | FALSE | insomnia | FALSE |
| Extreme polydrug users | Never used drugs | -0.364 | 0.5345 | -0.915 | 0.187 | FALSE | insomnia | FALSE |
| Extreme polydrug users | Psychedelics and cannabis users | -0.3958 | 0.5216 | -0.9898 | 0.1982 | FALSE | insomnia | FALSE |
| Extreme polydrug users | Unknown/Unwilling to disclose | -0.4031 | 0.3817 | -0.9549 | 0.1488 | FALSE | insomnia | FALSE |
| Extreme polydrug users | Users of other drugs | -0.2373 | 0.959 | -0.8257 | 0.3511 | FALSE | insomnia | FALSE |
| MDMA/ecstasy and cannabis users | Never used drugs | 0.1889 | 0.7485 | -0.147 | 0.5249 | FALSE | insomnia | FALSE |
| MDMA/ecstasy and cannabis users | Psychedelics and cannabis users | 0.1571 | 0.9669 | -0.2456 | 0.5598 | FALSE | insomnia | FALSE |
| MDMA/ecstasy and cannabis users | Unknown/Unwilling to disclose | 0.1499 | 0.9259 | -0.1874 | 0.4872 | FALSE | insomnia | FALSE |
| MDMA/ecstasy and cannabis users | Users of other drugs | 0.3156 | 0.2512 | -0.0788 | 0.7099 | FALSE | insomnia | FALSE |
| Never used drugs | Psychedelics and cannabis users | -0.0318 | 1 | -0.2559 | 0.1923 | FALSE | insomnia | FALSE |
| Never used drugs | Unknown/Unwilling to disclose | -0.0391 | 0.1111 | -0.082 | 0.0038 | FALSE | insomnia | FALSE |
| Never used drugs | Users of other drugs | 0.1267 | 0.6555 | -0.0821 | 0.3354 | FALSE | insomnia | FALSE |
| Psychedelics and cannabis users | Unknown/Unwilling to disclose | -0.0072 | 1 | -0.2334 | 0.2189 | FALSE | insomnia | FALSE |
| Psychedelics and cannabis users | Users of other drugs | 0.1585 | 0.8257 | -0.1463 | 0.4632 | FALSE | insomnia | FALSE |
| Unknown/Unwilling to disclose | Users of other drugs | 0.1657 | 0.276 | -0.0452 | 0.3767 | FALSE | insomnia | FALSE |
| Cannabis and cocaine users | Cannabis users | -0.0627 | 0.993 | -0.2637 | 0.1382 | FALSE | MH composite | FALSE |
| Cannabis and cocaine users | Cocaine users | -0.0235 | 1 | -0.2848 | 0.2379 | FALSE | MH composite | FALSE |
| Cannabis and cocaine users | Drug use history but no pandemic use | -0.0692 | 0.9772 | -0.2567 | 0.1184 | FALSE | MH composite | FALSE |
| Cannabis and cocaine users | Extreme polydrug users | 0.2499 | 0.9384 | -0.3302 | 0.83 | FALSE | MH composite | FALSE |
| Cannabis and cocaine users | MDMA/ecstasy and cannabis users | -0.3512 | 0.1027 | -0.7331 | 0.0307 | FALSE | MH composite | FALSE |
| Cannabis and cocaine users | Never used drugs | -0.0939 | 0.8418 | -0.2781 | 0.0902 | FALSE | MH composite | FALSE |
| Cannabis and cocaine users | Psychedelics and cannabis users | 0.0876 | 0.9943 | -0.2008 | 0.3761 | FALSE | MH composite | FALSE |
| Cannabis and cocaine users | Unknown/Unwilling to disclose | -0.1332 | 0.4173 | -0.3198 | 0.0535 | FALSE | MH composite | FALSE |
| Cannabis and cocaine users | Users of other drugs | 0.093 | 0.9881 | -0.1837 | 0.3697 | FALSE | MH composite | FALSE |
| Cannabis users | Cocaine users | 0.0392 | 0.9999 | -0.1652 | 0.2436 | FALSE | MH composite | FALSE |
| Cannabis users | Drug use history but no pandemic use | -0.0065 | 1 | -0.0995 | 0.0866 | FALSE | MH composite | FALSE |
| Cannabis users | Extreme polydrug users | 0.3126 | 0.7503 | -0.2442 | 0.8694 | FALSE | MH composite | FALSE |
| Cannabis users | MDMA/ecstasy and cannabis users | -0.2885 | 0.1979 | -0.6339 | 0.0569 | FALSE | MH composite | FALSE |
| Cannabis users | Never used drugs | -0.0312 | 0.9795 | -0.1172 | 0.0547 | FALSE | MH composite | FALSE |
| Cannabis users | Psychedelics and cannabis users | 0.1504 | 0.6004 | -0.0877 | 0.3884 | FALSE | MH composite | FALSE |
| Cannabis users | Unknown/Unwilling to disclose | -0.0704 | 0.2993 | -0.1616 | 0.0207 | FALSE | MH composite | FALSE |
| Cannabis users | Users of other drugs | 0.1557 | 0.4553 | -0.068 | 0.3794 | FALSE | MH composite | FALSE |
| Cocaine users | Drug use history but no pandemic use | -0.0457 | 0.9991 | -0.237 | 0.1455 | FALSE | MH composite | FALSE |
| Cocaine users | Extreme polydrug users | 0.2734 | 0.8974 | -0.3079 | 0.8547 | FALSE | MH composite | FALSE |
| Cocaine users | MDMA/ecstasy and cannabis users | -0.3277 | 0.1725 | -0.7114 | 0.056 | FALSE | MH composite | FALSE |
| Cocaine users | Never used drugs | -0.0705 | 0.9746 | -0.2584 | 0.1174 | FALSE | MH composite | FALSE |
| Cocaine users | Psychedelics and cannabis users | 0.1111 | 0.9712 | -0.1797 | 0.402 | FALSE | MH composite | FALSE |
| Cocaine users | Unknown/Unwilling to disclose | -0.1097 | 0.7206 | -0.3 | 0.0807 | FALSE | MH composite | FALSE |
| Cocaine users | Users of other drugs | 0.1164 | 0.9495 | -0.1628 | 0.3956 | FALSE | MH composite | FALSE |
| Drug use history but no pandemic use | Extreme polydrug users | 0.3191 | 0.717 | -0.233 | 0.8712 | FALSE | MH composite | FALSE |
| Drug use history but no pandemic use | MDMA/ecstasy and cannabis users | -0.282 | 0.1984 | -0.6198 | 0.0558 | FALSE | MH composite | FALSE |
| Drug use history but no pandemic use | Never used drugs | -0.0247 | 0.8099 | -0.0715 | 0.022 | FALSE | MH composite | FALSE |
| Drug use history but no pandemic use | Psychedelics and cannabis users | 0.1568 | 0.4657 | -0.0701 | 0.3837 | FALSE | MH composite | FALSE |
| Drug use history but no pandemic use | Unknown/Unwilling to disclose | -0.064 | 0.0106 | -0.1197 | -0.0082 | TRUE | MH composite | FALSE |
| Drug use history but no pandemic use | Users of other drugs | 0.1622 | 0.3118 | -0.0496 | 0.3739 | FALSE | MH composite | FALSE |
| Extreme polydrug users | MDMA/ecstasy and cannabis users | -0.6011 | 0.0921 | -1.2456 | 0.0434 | FALSE | MH composite | FALSE |
| Extreme polydrug users | Never used drugs | -0.3438 | 0.6171 | -0.8947 | 0.2071 | FALSE | MH composite | FALSE |
| Extreme polydrug users | Psychedelics and cannabis users | -0.1622 | 0.9974 | -0.7562 | 0.4317 | FALSE | MH composite | FALSE |
| Extreme polydrug users | Unknown/Unwilling to disclose | -0.383 | 0.4591 | -0.9348 | 0.1687 | FALSE | MH composite | FALSE |
| Extreme polydrug users | Users of other drugs | -0.1569 | 0.9979 | -0.7453 | 0.4314 | FALSE | MH composite | FALSE |
| MDMA/ecstasy and cannabis users | Never used drugs | 0.2573 | 0.3117 | -0.0786 | 0.5932 | FALSE | MH composite | FALSE |
| MDMA/ecstasy and cannabis users | Psychedelics and cannabis users | 0.4388 | 0.0201 | 0.0362 | 0.8415 | TRUE | MH composite | FALSE |
| MDMA/ecstasy and cannabis users | Unknown/Unwilling to disclose | 0.218 | 0.5665 | -0.1192 | 0.5553 | FALSE | MH composite | FALSE |
| MDMA/ecstasy and cannabis users | Users of other drugs | 0.4442 | 0.0135 | 0.0499 | 0.8385 | TRUE | MH composite | FALSE |
| Never used drugs | Psychedelics and cannabis users | 0.1816 | 0.2351 | -0.0425 | 0.4057 | FALSE | MH composite | FALSE |
| Never used drugs | Unknown/Unwilling to disclose | -0.0392 | 0.1077 | -0.0821 | 0.0037 | FALSE | MH composite | FALSE |
| Never used drugs | Users of other drugs | 0.1869 | 0.1254 | -0.0218 | 0.3956 | FALSE | MH composite | FALSE |
| Psychedelics and cannabis users | Unknown/Unwilling to disclose | -0.2208 | 0.0624 | -0.4469 | 0.0053 | FALSE | MH composite | FALSE |
| Psychedelics and cannabis users | Users of other drugs | 0.0053 | 1 | -0.2994 | 0.3101 | FALSE | MH composite | FALSE |
| Unknown/Unwilling to disclose | Users of other drugs | 0.2261 | 0.0243 | 0.0152 | 0.437 | TRUE | MH composite | FALSE |

**Supp Table 5. Mood post-hoc.**

| group1 | group2 | meandiff | p | lower | upper | sig | variable | sig_corrected |
| --- | --- | --- | --- | --- | --- | --- | --- | --- |
| Cannabis and cocaine users | Cannabis users | -0.0086 | 1 | -0.2119 | 0.1947 | FALSE | more relaxed lifestyle | FALSE |
| Cannabis and cocaine users | Cocaine users | 0.0363 | 1 | -0.2281 | 0.3007 | FALSE | more relaxed lifestyle | FALSE |
| Cannabis and cocaine users | Drug use history but no pandemic use | -0.0638 | 0.988 | -0.2536 | 0.1259 | FALSE | more relaxed lifestyle | FALSE |
| Cannabis and cocaine users | Extreme polydrug users | 0.1086 | 0.9999 | -0.4783 | 0.6955 | FALSE | more relaxed lifestyle | FALSE |
| Cannabis and cocaine users | MDMA/ecstasy and cannabis users | -0.0067 | 1 | -0.393 | 0.3797 | FALSE | more relaxed lifestyle | FALSE |
| Cannabis and cocaine users | Never used drugs | -0.1187 | 0.5874 | -0.305 | 0.0676 | FALSE | more relaxed lifestyle | FALSE |
| Cannabis and cocaine users | Psychedelics and cannabis users | 0.0715 | 0.9989 | -0.2203 | 0.3634 | FALSE | more relaxed lifestyle | FALSE |
| Cannabis and cocaine users | Unknown/Unwilling to disclose | -0.1382 | 0.3791 | -0.327 | 0.0507 | FALSE | more relaxed lifestyle | FALSE |
| Cannabis and cocaine users | Users of other drugs | 0.1213 | 0.936 | -0.1586 | 0.4013 | FALSE | more relaxed lifestyle | FALSE |
| Cannabis users | Cocaine users | 0.0449 | 0.9996 | -0.1619 | 0.2518 | FALSE | more relaxed lifestyle | FALSE |
| Cannabis users | Drug use history but no pandemic use | -0.0552 | 0.6996 | -0.1494 | 0.039 | FALSE | more relaxed lifestyle | FALSE |
| Cannabis users | Extreme polydrug users | 0.1172 | 0.9997 | -0.4461 | 0.6805 | FALSE | more relaxed lifestyle | FALSE |
| Cannabis users | MDMA/ecstasy and cannabis users | 0.0019 | 1 | -0.3475 | 0.3514 | FALSE | more relaxed lifestyle | FALSE |
| Cannabis users | Never used drugs | -0.1101 | 0.0025 | -0.1972 | -0.0231 | TRUE | more relaxed lifestyle | FALSE |
| Cannabis users | Psychedelics and cannabis users | 0.0802 | 0.9889 | -0.1607 | 0.3211 | FALSE | more relaxed lifestyle | FALSE |
| Cannabis users | Unknown/Unwilling to disclose | -0.1295 | 0.0004 | -0.2218 | -0.0372 | TRUE | more relaxed lifestyle | FALSE |
| Cannabis users | Users of other drugs | 0.13 | 0.7249 | -0.0964 | 0.3563 | FALSE | more relaxed lifestyle | FALSE |
| Cocaine users | Drug use history but no pandemic use | -0.1002 | 0.8295 | -0.2937 | 0.0933 | FALSE | more relaxed lifestyle | FALSE |
| Cocaine users | Extreme polydrug users | 0.0723 | 1 | -0.5159 | 0.6604 | FALSE | more relaxed lifestyle | FALSE |
| Cocaine users | MDMA/ecstasy and cannabis users | -0.043 | 1 | -0.4312 | 0.3452 | FALSE | more relaxed lifestyle | FALSE |
| Cocaine users | Never used drugs | -0.1551 | 0.2267 | -0.3452 | 0.035 | FALSE | more relaxed lifestyle | FALSE |
| Cocaine users | Psychedelics and cannabis users | 0.0352 | 1 | -0.2591 | 0.3295 | FALSE | more relaxed lifestyle | FALSE |
| Cocaine users | Unknown/Unwilling to disclose | -0.1745 | 0.1151 | -0.3671 | 0.0181 | FALSE | more relaxed lifestyle | FALSE |
| Cocaine users | Users of other drugs | 0.085 | 0.9947 | -0.1975 | 0.3675 | FALSE | more relaxed lifestyle | FALSE |
| Drug use history but no pandemic use | Extreme polydrug users | 0.1724 | 0.9936 | -0.3861 | 0.731 | FALSE | more relaxed lifestyle | FALSE |
| Drug use history but no pandemic use | MDMA/ecstasy and cannabis users | 0.0572 | 1 | -0.2846 | 0.3989 | FALSE | more relaxed lifestyle | FALSE |
| Drug use history but no pandemic use | Never used drugs | -0.0549 | 0.0092 | -0.1022 | -0.0076 | TRUE | more relaxed lifestyle | FALSE |
| Drug use history but no pandemic use | Psychedelics and cannabis users | 0.1354 | 0.6923 | -0.0942 | 0.365 | FALSE | more relaxed lifestyle | FALSE |
| Drug use history but no pandemic use | Unknown/Unwilling to disclose | -0.0743 | 0.0013 | -0.1307 | -0.0179 | TRUE | more relaxed lifestyle | FALSE |
| Drug use history but no pandemic use | Users of other drugs | 0.1852 | 0.1597 | -0.029 | 0.3994 | FALSE | more relaxed lifestyle | FALSE |
| Extreme polydrug users | MDMA/ecstasy and cannabis users | -0.1153 | 0.9999 | -0.7674 | 0.5368 | FALSE | more relaxed lifestyle | FALSE |
| Extreme polydrug users | Never used drugs | -0.2273 | 0.9561 | -0.7847 | 0.3301 | FALSE | more relaxed lifestyle | FALSE |
| Extreme polydrug users | Psychedelics and cannabis users | -0.037 | 1 | -0.638 | 0.5639 | FALSE | more relaxed lifestyle | FALSE |
| Extreme polydrug users | Unknown/Unwilling to disclose | -0.2467 | 0.9281 | -0.805 | 0.3115 | FALSE | more relaxed lifestyle | FALSE |
| Extreme polydrug users | Users of other drugs | 0.0127 | 1 | -0.5825 | 0.608 | FALSE | more relaxed lifestyle | FALSE |
| MDMA/ecstasy and cannabis users | Never used drugs | -0.1121 | 0.9896 | -0.4519 | 0.2278 | FALSE | more relaxed lifestyle | FALSE |
| MDMA/ecstasy and cannabis users | Psychedelics and cannabis users | 0.0782 | 0.9999 | -0.3292 | 0.4856 | FALSE | more relaxed lifestyle | FALSE |
| MDMA/ecstasy and cannabis users | Unknown/Unwilling to disclose | -0.1315 | 0.9695 | -0.4727 | 0.2098 | FALSE | more relaxed lifestyle | FALSE |
| MDMA/ecstasy and cannabis users | Users of other drugs | 0.128 | 0.9914 | -0.2709 | 0.5269 | FALSE | more relaxed lifestyle | FALSE |
| Never used drugs | Psychedelics and cannabis users | 0.1903 | 0.1922 | -0.0364 | 0.417 | FALSE | more relaxed lifestyle | FALSE |
| Never used drugs | Unknown/Unwilling to disclose | -0.0194 | 0.9233 | -0.0629 | 0.024 | FALSE | more relaxed lifestyle | FALSE |
| Never used drugs | Users of other drugs | 0.2401 | 0.012 | 0.0289 | 0.4513 | TRUE | more relaxed lifestyle | FALSE |
| Psychedelics and cannabis users | Unknown/Unwilling to disclose | -0.2097 | 0.1055 | -0.4385 | 0.0191 | FALSE | more relaxed lifestyle | FALSE |
| Psychedelics and cannabis users | Users of other drugs | 0.0498 | 1 | -0.2585 | 0.3581 | FALSE | more relaxed lifestyle | FALSE |
| Unknown/Unwilling to disclose | Users of other drugs | 0.2595 | 0.0047 | 0.0461 | 0.4729 | TRUE | more relaxed lifestyle | FALSE |
| Cannabis and cocaine users | Cannabis users | 0.0577 | 0.9973 | -0.1521 | 0.2675 | FALSE | disrupted lifestyle | FALSE |
| Cannabis and cocaine users | Cocaine users | 0.1398 | 0.8383 | -0.1331 | 0.4126 | FALSE | disrupted lifestyle | FALSE |
| Cannabis and cocaine users | Drug use history but no pandemic use | 0.125 | 0.5849 | -0.0708 | 0.3208 | FALSE | disrupted lifestyle | FALSE |
| Cannabis and cocaine users | Extreme polydrug users | 0.3662 | 0.6599 | -0.2393 | 0.9718 | FALSE | disrupted lifestyle | FALSE |
| Cannabis and cocaine users | MDMA/ecstasy and cannabis users | 0.1038 | 0.9982 | -0.2949 | 0.5024 | FALSE | disrupted lifestyle | FALSE |
| Cannabis and cocaine users | Never used drugs | 0.1008 | 0.8185 | -0.0914 | 0.293 | FALSE | disrupted lifestyle | FALSE |
| Cannabis and cocaine users | Psychedelics and cannabis users | 0.1169 | 0.968 | -0.1842 | 0.418 | FALSE | disrupted lifestyle | FALSE |
| Cannabis and cocaine users | Unknown/Unwilling to disclose | 0.1124 | 0.7196 | -0.0825 | 0.3072 | FALSE | disrupted lifestyle | FALSE |
| Cannabis and cocaine users | Users of other drugs | 0.3134 | 0.0212 | 0.0246 | 0.6022 | TRUE | disrupted lifestyle | FALSE |
| Cannabis users | Cocaine users | 0.0821 | 0.9699 | -0.1313 | 0.2954 | FALSE | disrupted lifestyle | FALSE |
| Cannabis users | Drug use history but no pandemic use | 0.0673 | 0.4632 | -0.0299 | 0.1645 | FALSE | disrupted lifestyle | FALSE |
| Cannabis users | Extreme polydrug users | 0.3085 | 0.8073 | -0.2727 | 0.8898 | FALSE | disrupted lifestyle | FALSE |
| Cannabis users | MDMA/ecstasy and cannabis users | 0.0461 | 1 | -0.3145 | 0.4066 | FALSE | disrupted lifestyle | FALSE |
| Cannabis users | Never used drugs | 0.0431 | 0.8851 | -0.0467 | 0.1329 | FALSE | disrupted lifestyle | FALSE |
| Cannabis users | Psychedelics and cannabis users | 0.0592 | 0.9991 | -0.1894 | 0.3078 | FALSE | disrupted lifestyle | FALSE |
| Cannabis users | Unknown/Unwilling to disclose | 0.0547 | 0.7253 | -0.0406 | 0.1499 | FALSE | disrupted lifestyle | FALSE |
| Cannabis users | Users of other drugs | 0.2557 | 0.019 | 0.0222 | 0.4892 | TRUE | disrupted lifestyle | FALSE |
| Cocaine users | Drug use history but no pandemic use | -0.0148 | 1 | -0.2144 | 0.1849 | FALSE | disrupted lifestyle | FALSE |
| Cocaine users | Extreme polydrug users | 0.2265 | 0.9753 | -0.3803 | 0.8333 | FALSE | disrupted lifestyle | FALSE |
| Cocaine users | MDMA/ecstasy and cannabis users | -0.036 | 1 | -0.4365 | 0.3645 | FALSE | disrupted lifestyle | FALSE |
| Cocaine users | Never used drugs | -0.039 | 0.9998 | -0.2351 | 0.1572 | FALSE | disrupted lifestyle | FALSE |
| Cocaine users | Psychedelics and cannabis users | -0.0229 | 1 | -0.3265 | 0.2808 | FALSE | disrupted lifestyle | FALSE |
| Cocaine users | Unknown/Unwilling to disclose | -0.0274 | 1 | -0.2261 | 0.1713 | FALSE | disrupted lifestyle | FALSE |
| Cocaine users | Users of other drugs | 0.1736 | 0.6794 | -0.1178 | 0.4651 | FALSE | disrupted lifestyle | FALSE |
| Drug use history but no pandemic use | Extreme polydrug users | 0.2413 | 0.9483 | -0.3351 | 0.8176 | FALSE | disrupted lifestyle | FALSE |
| Drug use history but no pandemic use | MDMA/ecstasy and cannabis users | -0.0212 | 1 | -0.3738 | 0.3314 | FALSE | disrupted lifestyle | FALSE |
| Drug use history but no pandemic use | Never used drugs | -0.0242 | 0.864 | -0.073 | 0.0246 | FALSE | disrupted lifestyle | FALSE |
| Drug use history but no pandemic use | Psychedelics and cannabis users | -0.0081 | 1 | -0.245 | 0.2288 | FALSE | disrupted lifestyle | FALSE |
| Drug use history but no pandemic use | Unknown/Unwilling to disclose | -0.0126 | 0.9996 | -0.0709 | 0.0456 | FALSE | disrupted lifestyle | FALSE |
| Drug use history but no pandemic use | Users of other drugs | 0.1884 | 0.1748 | -0.0326 | 0.4094 | FALSE | disrupted lifestyle | FALSE |
| Extreme polydrug users | MDMA/ecstasy and cannabis users | -0.2625 | 0.967 | -0.9353 | 0.4104 | FALSE | disrupted lifestyle | FALSE |
| Extreme polydrug users | Never used drugs | -0.2654 | 0.9076 | -0.8406 | 0.3097 | FALSE | disrupted lifestyle | FALSE |
| Extreme polydrug users | Psychedelics and cannabis users | -0.2494 | 0.9598 | -0.8694 | 0.3707 | FALSE | disrupted lifestyle | FALSE |
| Extreme polydrug users | Unknown/Unwilling to disclose | -0.2539 | 0.9293 | -0.8299 | 0.3221 | FALSE | disrupted lifestyle | FALSE |
| Extreme polydrug users | Users of other drugs | -0.0528 | 1 | -0.667 | 0.5613 | FALSE | disrupted lifestyle | FALSE |
| MDMA/ecstasy and cannabis users | Never used drugs | -0.003 | 1 | -0.3536 | 0.3477 | FALSE | disrupted lifestyle | FALSE |
| MDMA/ecstasy and cannabis users | Psychedelics and cannabis users | 0.0131 | 1 | -0.4072 | 0.4334 | FALSE | disrupted lifestyle | FALSE |
| MDMA/ecstasy and cannabis users | Unknown/Unwilling to disclose | 0.0086 | 1 | -0.3435 | 0.3607 | FALSE | disrupted lifestyle | FALSE |
| MDMA/ecstasy and cannabis users | Users of other drugs | 0.2096 | 0.8431 | -0.202 | 0.6212 | FALSE | disrupted lifestyle | FALSE |
| Never used drugs | Psychedelics and cannabis users | 0.0161 | 1 | -0.2178 | 0.25 | FALSE | disrupted lifestyle | FALSE |
| Never used drugs | Unknown/Unwilling to disclose | 0.0116 | 0.9984 | -0.0333 | 0.0564 | FALSE | disrupted lifestyle | FALSE |
| Never used drugs | Users of other drugs | 0.2126 | 0.0628 | -0.0053 | 0.4305 | FALSE | disrupted lifestyle | FALSE |
| Psychedelics and cannabis users | Unknown/Unwilling to disclose | -0.0045 | 1 | -0.2406 | 0.2315 | FALSE | disrupted lifestyle | FALSE |
| Psychedelics and cannabis users | Users of other drugs | 0.1965 | 0.6313 | -0.1216 | 0.5146 | FALSE | disrupted lifestyle | FALSE |
| Unknown/Unwilling to disclose | Users of other drugs | 0.201 | 0.1087 | -0.0192 | 0.4212 | FALSE | disrupted lifestyle | FALSE |
| Cannabis and cocaine users | Cannabis users | -0.0122 | 1 | -0.2166 | 0.1923 | FALSE | elevated health concerns | FALSE |
| Cannabis and cocaine users | Cocaine users | 0.0472 | 0.9999 | -0.2186 | 0.313 | FALSE | elevated health concerns | FALSE |
| Cannabis and cocaine users | Drug use history but no pandemic use | 0.0082 | 1 | -0.1826 | 0.1989 | FALSE | elevated health concerns | FALSE |
| Cannabis and cocaine users | Extreme polydrug users | -0.2862 | 0.8785 | -0.8762 | 0.3039 | FALSE | elevated health concerns | FALSE |
| Cannabis and cocaine users | MDMA/ecstasy and cannabis users | 0.0656 | 0.9999 | -0.3228 | 0.454 | FALSE | elevated health concerns | FALSE |
| Cannabis and cocaine users | Never used drugs | 0.0729 | 0.9673 | -0.1144 | 0.2603 | FALSE | elevated health concerns | FALSE |
| Cannabis and cocaine users | Psychedelics and cannabis users | 0.011 | 1 | -0.2824 | 0.3044 | FALSE | elevated health concerns | FALSE |
| Cannabis and cocaine users | Unknown/Unwilling to disclose | 0.0423 | 0.9995 | -0.1475 | 0.2322 | FALSE | elevated health concerns | FALSE |
| Cannabis and cocaine users | Users of ‚ other drugs | -0.1764 | 0.6108 | -0.4579 | 0.105 | FALSE | elevated health concerns | FALSE |
| Cannabis users | Cocaine users | 0.0594 | 0.9964 | -0.1486 | 0.2673 | FALSE | elevated health concerns | FALSE |
| Cannabis users | Drug use history but no pandemic use | 0.0203 | 0.9996 | -0.0744 | 0.115 | FALSE | elevated health concerns | FALSE |
| Cannabis users | Extreme polydrug users | -0.274 | 0.88 | -0.8404 | 0.2923 | FALSE | elevated health concerns | FALSE |
| Cannabis users | MDMA/ecstasy and cannabis users | 0.0778 | 0.9995 | -0.2736 | 0.4291 | FALSE | elevated health concerns | FALSE |
| Cannabis users | Never used drugs | 0.0851 | 0.0646 | -0.0024 | 0.1726 | FALSE | elevated health concerns | FALSE |
| Cannabis users | Psychedelics and cannabis users | 0.0231 | 1 | -0.2191 | 0.2653 | FALSE | elevated health concerns | FALSE |
| Cannabis users | Unknown/Unwilling to disclose | 0.0545 | 0.6977 | -0.0383 | 0.1473 | FALSE | elevated health concerns | FALSE |
| Cannabis users | Users of other drugs | -0.1643 | 0.3994 | -0.3918 | 0.0633 | FALSE | elevated health concerns | FALSE |
| Cocaine users | Drug use history but no pandemic use | -0.0391 | 0.9998 | -0.2336 | 0.1555 | FALSE | elevated health concerns | FALSE |
| Cocaine users | Extreme polydrug users | -0.3334 | 0.7456 | -0.9247 | 0.2579 | FALSE | elevated health concerns | FALSE |
| Cocaine users | MDMA/ecstasy and cannabis users | 0.0184 | 1 | -0.3719 | 0.4087 | FALSE | elevated health concerns | FALSE |
| Cocaine users | Never used drugs | 0.0257 | 1 | -0.1654 | 0.2169 | FALSE | elevated health concerns | FALSE |
| Cocaine users | Psychedelics and cannabis users | -0.0363 | 1 | -0.3321 | 0.2596 | FALSE | elevated health concerns | FALSE |
| Cocaine users | Unknown/Unwilling to disclose | -0.0049 | 1 | -0.1985 | 0.1887 | FALSE | elevated health concerns | FALSE |
| Cocaine users | Users of other drugs | -0.2237 | 0.2728 | -0.5077 | 0.0604 | FALSE | elevated health concerns | FALSE |
| Drug use history but no pandemic use | Extreme polydrug users | -0.2943 | 0.8189 | -0.8559 | 0.2672 | FALSE | elevated health concerns | FALSE |
| Drug use history but no pandemic use | MDMA/ecstasy and cannabis users | 0.0574 | 1 | -0.2861 | 0.401 | FALSE | elevated health concerns | FALSE |
| Drug use history but no pandemic use | Never used drugs | 0.0648 | 0.0007 | 0.0172 | 0.1124 | TRUE | elevated health concerns | FALSE |
| Drug use history but no pandemic use | Psychedelics and cannabis users | 0.0028 | 1 | -0.228 | 0.2336 | FALSE | elevated health concerns | FALSE |
| Drug use history but no pandemic use | Unknown/Unwilling to disclose | 0.0342 | 0.6651 | -0.0226 | 0.0909 | FALSE | elevated health concerns | FALSE |
| Drug use history but no pandemic use | Users of other drugs | -0.1846 | 0.1688 | -0.4 | 0.0308 | FALSE | elevated health concerns | FALSE |
| Extreme polydrug users | MDMA/ecstasy and cannabis users | 0.3518 | 0.7972 | -0.3038 | 1.0074 | FALSE | elevated health concerns | FALSE |
| Extreme polydrug users | Never used drugs | 0.3591 | 0.5794 | -0.2013 | 0.9195 | FALSE | elevated health concerns | FALSE |
| Extreme polydrug users | Psychedelics and cannabis users | 0.2971 | 0.8691 | -0.3071 | 0.9013 | FALSE | elevated health concerns | FALSE |
| Extreme polydrug users | Unknown/Unwilling to disclose | 0.3285 | 0.7017 | -0.2327 | 0.8898 | FALSE | elevated health concerns | FALSE |
| Extreme polydrug users | Users of other drugs | 0.1097 | 0.9999 | -0.4887 | 0.7082 | FALSE | elevated health concerns | FALSE |
| MDMA/ecstasy and cannabis users | Never used drugs | 0.0073 | 1 | -0.3343 | 0.349 | FALSE | elevated health concerns | FALSE |
| MDMA/ecstasy and cannabis users | Psychedelics and cannabis users | -0.0546 | 1 | -0.4642 | 0.3549 | FALSE | elevated health concerns | FALSE |
| MDMA/ecstasy and cannabis users | Unknown/Unwilling to disclose | -0.0233 | 1 | -0.3663 | 0.3198 | FALSE | elevated health concerns | FALSE |
| MDMA/ecstasy and cannabis users | Users of other drugs | -0.242 | 0.6628 | -0.6431 | 0.159 | FALSE | elevated health concerns | FALSE |
| Never used drugs | Psychedelics and cannabis users | -0.062 | 0.9975 | -0.2899 | 0.166 | FALSE | elevated health concerns | FALSE |
| Never used drugs | Unknown/Unwilling to disclose | -0.0306 | 0.4452 | -0.0743 | 0.0131 | FALSE | elevated health concerns | FALSE |
| Never used drugs | Users of other drugs | -0.2494 | 0.0077 | -0.4617 | -0.0371 | TRUE | elevated health concerns | FALSE |
| Psychedelics and cannabis users | Unknown/Unwilling to disclose | 0.0314 | 1 | -0.1986 | 0.2614 | FALSE | elevated health concerns | FALSE |
| Psychedelics and cannabis users | Users of other drugs | -0.1874 | 0.6604 | -0.4974 | 0.1226 | FALSE | elevated health concerns | FALSE |
| Unknown/Unwilling to disclose | Users of other drugs | -0.2188 | 0.0413 | -0.4333 | -0.0042 | TRUE | elevated health concerns | FALSE |
| Cannabis and cocaine users | Cannabis users | 0.0166 | 1 | -0.1853 | 0.2184 | FALSE | optimism | FALSE |
| Cannabis and cocaine users | Cocaine users | 0.0046 | 1 | -0.2579 | 0.2671 | FALSE | optimism | FALSE |
| Cannabis and cocaine users | Drug use history but no pandemic use | -0.0376 | 0.9998 | -0.226 | 0.1508 | FALSE | optimism | FALSE |
| Cannabis and cocaine users | Extreme polydrug users | -0.3535 | 0.6558 | -0.9362 | 0.2292 | FALSE | optimism | FALSE |
| Cannabis and cocaine users | MDMA/ecstasy and cannabis users | 0.1615 | 0.9464 | -0.2221 | 0.545 | FALSE | optimism | FALSE |
| Cannabis and cocaine users | Never used drugs | -0.0206 | 1 | -0.2055 | 0.1644 | FALSE | optimism | FALSE |
| Cannabis and cocaine users | Psychedelics and cannabis users | 0.0687 | 0.9992 | -0.221 | 0.3584 | FALSE | optimism | FALSE |
| Cannabis and cocaine users | Unknown/Unwilling to disclose | -0.0425 | 0.9994 | -0.23 | 0.145 | FALSE | optimism | FALSE |
| Cannabis and cocaine users | Users of other drugs | -0.1149 | 0.9523 | -0.3928 | 0.1631 | FALSE | optimism | FALSE |
| Cannabis users | Cocaine users | -0.012 | 1 | -0.2173 | 0.1934 | FALSE | optimism | FALSE |
| Cannabis users | Drug use history but no pandemic use | -0.0542 | 0.7148 | -0.1477 | 0.0394 | FALSE | optimism | FALSE |
| Cannabis users | Extreme polydrug users | -0.3701 | 0.532 | -0.9293 | 0.1892 | FALSE | optimism | FALSE |
| Cannabis users | MDMA/ecstasy and cannabis users | 0.1449 | 0.949 | -0.2021 | 0.4919 | FALSE | optimism | FALSE |
| Cannabis users | Never used drugs | -0.0371 | 0.9392 | -0.1236 | 0.0493 | FALSE | optimism | FALSE |
| Cannabis users | Psychedelics and cannabis users | 0.0521 | 0.9996 | -0.1871 | 0.2913 | FALSE | optimism | FALSE |
| Cannabis users | Unknown/Unwilling to disclose | -0.0591 | 0.5703 | -0.1507 | 0.0325 | FALSE | optimism | FALSE |
| Cannabis users | Users of other drugs | -0.1314 | 0.7026 | -0.3561 | 0.0933 | FALSE | optimism | FALSE |
| Cocaine users | Drug use history but no pandemic use | -0.0422 | 0.9995 | -0.2343 | 0.1499 | FALSE | optimism | FALSE |
| Cocaine users | Extreme polydrug users | -0.3581 | 0.6411 | -0.942 | 0.2258 | FALSE | optimism | FALSE |
| Cocaine users | MDMA/ecstasy and cannabis users | 0.1569 | 0.9566 | -0.2286 | 0.5423 | FALSE | optimism | FALSE |
| Cocaine users | Never used drugs | -0.0252 | 1 | -0.2139 | 0.1636 | FALSE | optimism | FALSE |
| Cocaine users | Psychedelics and cannabis users | 0.0641 | 0.9996 | -0.2281 | 0.3562 | FALSE | optimism | FALSE |
| Cocaine users | Unknown/Unwilling to disclose | -0.0471 | 0.9989 | -0.2383 | 0.1441 | FALSE | optimism | FALSE |
| Cocaine users | Users of other drugs | -0.1195 | 0.9424 | -0.3999 | 0.161 | FALSE | optimism | FALSE |
| Drug use history but no pandemic use | Extreme polydrug users | -0.3159 | 0.7339 | -0.8705 | 0.2386 | FALSE | optimism | FALSE |
| Drug use history but no pandemic use | MDMA/ecstasy and cannabis users | 0.1991 | 0.6989 | -0.1402 | 0.5383 | FALSE | optimism | FALSE |
| Drug use history but no pandemic use | Never used drugs | 0.017 | 0.9799 | -0.03 | 0.064 | FALSE | optimism | FALSE |
| Drug use history but no pandemic use | Psychedelics and cannabis users | 0.1063 | 0.9021 | -0.1216 | 0.3342 | FALSE | optimism | FALSE |
| Drug use history but no pandemic use | Unknown/Unwilling to disclose | -0.0049 | 1 | -0.061 | 0.0511 | FALSE | optimism | FALSE |
| Drug use history but no pandemic use | Users of other drugs | -0.0773 | 0.9794 | -0.29 | 0.1354 | FALSE | optimism | FALSE |
| Extreme polydrug users | MDMA/ecstasy and cannabis users | 0.515 | 0.2593 | -0.1324 | 1.1624 | FALSE | optimism | FALSE |
| Extreme polydrug users | Never used drugs | 0.3329 | 0.6668 | -0.2205 | 0.8863 | FALSE | optimism | FALSE |
| Extreme polydrug users | Psychedelics and cannabis users | 0.4222 | 0.4299 | -0.1744 | 1.0188 | FALSE | optimism | FALSE |
| Extreme polydrug users | Unknown/Unwilling to disclose | 0.311 | 0.7511 | -0.2433 | 0.8652 | FALSE | optimism | FALSE |
| Extreme polydrug users | Users of other drugs | 0.2386 | 0.9587 | -0.3523 | 0.8296 | FALSE | optimism | FALSE |
| MDMA/ecstasy and cannabis users | Never used drugs | -0.182 | 0.7919 | -0.5194 | 0.1554 | FALSE | optimism | FALSE |
| MDMA/ecstasy and cannabis users | Psychedelics and cannabis users | -0.0928 | 0.9994 | -0.4972 | 0.3117 | FALSE | optimism | FALSE |
| MDMA/ecstasy and cannabis users | Unknown/Unwilling to disclose | -0.204 | 0.6657 | -0.5428 | 0.1348 | FALSE | optimism | FALSE |
| MDMA/ecstasy and cannabis users | Users of other drugs | -0.2763 | 0.4516 | -0.6724 | 0.1197 | FALSE | optimism | FALSE |
| Never used drugs | Psychedelics and cannabis users | 0.0893 | 0.9633 | -0.1358 | 0.3144 | FALSE | optimism | FALSE |
| Never used drugs | Unknown/Unwilling to disclose | -0.022 | 0.8435 | -0.0651 | 0.0212 | FALSE | optimism | FALSE |
| Never used drugs | Users of other drugs | -0.0943 | 0.9204 | -0.304 | 0.1154 | FALSE | optimism | FALSE |
| Psychedelics and cannabis users | Unknown/Unwilling to disclose | -0.1112 | 0.8721 | -0.3384 | 0.1159 | FALSE | optimism | FALSE |
| Psychedelics and cannabis users | Users of other drugs | -0.1836 | 0.671 | -0.4897 | 0.1225 | FALSE | optimism | FALSE |
| Unknown/Unwilling to disclose | Users of other drugs | -0.0723 | 0.9867 | -0.2842 | 0.1395 | FALSE | optimism | FALSE |
| Cannabis and cocaine users | Cannabis users | 0.0372 | 0.9999 | -0.1662 | 0.2405 | FALSE | increased conflict at home | FALSE |
| Cannabis and cocaine users | Cocaine users | 0.0143 | 1 | -0.2502 | 0.2788 | FALSE | increased conflict at home | FALSE |
| Cannabis and cocaine users | Drug use history but no pandemic use | -0.0016 | 1 | -0.1914 | 0.1882 | FALSE | increased conflict at home | FALSE |
| Cannabis and cocaine users | Extreme polydrug users | -0.0515 | 1 | -0.6386 | 0.5355 | FALSE | increased conflict at home | FALSE |
| Cannabis and cocaine users | MDMA/ecstasy and cannabis users | -0.0182 | 1 | -0.4046 | 0.3682 | FALSE | increased conflict at home | FALSE |
| Cannabis and cocaine users | Never used drugs | 0.0416 | 0.9995 | -0.1447 | 0.228 | FALSE | increased conflict at home | FALSE |
| Cannabis and cocaine users | Psychedelics and cannabis users | 0.0379 | 1 | -0.254 | 0.3298 | FALSE | increased conflict at home | FALSE |
| Cannabis and cocaine users | Unknown/Unwilling to disclose | -0.0062 | 1 | -0.1951 | 0.1826 | FALSE | increased conflict at home | FALSE |
| Cannabis and cocaine users | Users of other drugs | -0.0558 | 0.9998 | -0.3358 | 0.2242 | FALSE | increased conflict at home | FALSE |
| Cannabis users | Cocaine users | -0.0228 | 1 | -0.2297 | 0.184 | FALSE | increased conflict at home | FALSE |
| Cannabis users | Drug use history but no pandemic use | -0.0387 | 0.9537 | -0.133 | 0.0555 | FALSE | increased conflict at home | FALSE |
| Cannabis users | Extreme polydrug users | -0.0887 | 1 | -0.6521 | 0.4748 | FALSE | increased conflict at home | FALSE |
| Cannabis users | MDMA/ecstasy and cannabis users | -0.0554 | 1 | -0.4049 | 0.2942 | FALSE | increased conflict at home | FALSE |
| Cannabis users | Never used drugs | 0.0045 | 1 | -0.0826 | 0.0915 | FALSE | increased conflict at home | FALSE |
| Cannabis users | Psychedelics and cannabis users | 0.0007 | 1 | -0.2402 | 0.2417 | FALSE | increased conflict at home | FALSE |
| Cannabis users | Unknown/Unwilling to disclose | -0.0434 | 0.8977 | -0.1357 | 0.0489 | FALSE | increased conflict at home | FALSE |
| Cannabis users | Users of other drugs | -0.093 | 0.954 | -0.3194 | 0.1334 | FALSE | increased conflict at home | FALSE |
| Cocaine users | Drug use history but no pandemic use | -0.0159 | 1 | -0.2094 | 0.1776 | FALSE | increased conflict at home | FALSE |
| Cocaine users | Extreme polydrug users | -0.0658 | 1 | -0.6541 | 0.5224 | FALSE | increased conflict at home | FALSE |
| Cocaine users | MDMA/ecstasy and cannabis users | -0.0325 | 1 | -0.4208 | 0.3558 | FALSE | increased conflict at home | FALSE |
| Cocaine users | Never used drugs | 0.0273 | 1 | -0.1629 | 0.2175 | FALSE | increased conflict at home | FALSE |
| Cocaine users | Psychedelics and cannabis users | 0.0236 | 1 | -0.2708 | 0.3179 | FALSE | increased conflict at home | FALSE |
| Cocaine users | Unknown/Unwilling to disclose | -0.0205 | 1 | -0.2132 | 0.1721 | FALSE | increased conflict at home | FALSE |
| Cocaine users | Users of other drugs | -0.0701 | 0.9988 | -0.3527 | 0.2124 | FALSE | increased conflict at home | FALSE |
| Drug use history but no pandemic use | Extreme polydrug users | -0.0499 | 1 | -0.6086 | 0.5088 | FALSE | increased conflict at home | FALSE |
| Drug use history but no pandemic use | MDMA/ecstasy and cannabis users | -0.0166 | 1 | -0.3585 | 0.3252 | FALSE | increased conflict at home | FALSE |
| Drug use history but no pandemic use | Never used drugs | 0.0432 | 0.1091 | -0.0041 | 0.0905 | FALSE | increased conflict at home | FALSE |
| Drug use history but no pandemic use | Psychedelics and cannabis users | 0.0395 | 0.9999 | -0.1902 | 0.2691 | FALSE | increased conflict at home | FALSE |
| Drug use history but no pandemic use | Unknown/Unwilling to disclose | -0.0046 | 1 | -0.0611 | 0.0518 | FALSE | increased conflict at home | FALSE |
| Drug use history but no pandemic use | Users of other drugs | -0.0543 | 0.9986 | -0.2685 | 0.16 | FALSE | increased conflict at home | FALSE |
| Extreme polydrug users | MDMA/ecstasy and cannabis users | 0.0333 | 1 | -0.619 | 0.6856 | FALSE | increased conflict at home | FALSE |
| Extreme polydrug users | Never used drugs | 0.0931 | 1 | -0.4644 | 0.6507 | FALSE | increased conflict at home | FALSE |
| Extreme polydrug users | Psychedelics and cannabis users | 0.0894 | 1 | -0.5117 | 0.6905 | FALSE | increased conflict at home | FALSE |
| Extreme polydrug users | Unknown/Unwilling to disclose | 0.0453 | 1 | -0.5131 | 0.6037 | FALSE | increased conflict at home | FALSE |
| Extreme polydrug users | Users of other drugs | -0.0043 | 1 | -0.5997 | 0.5911 | FALSE | increased conflict at home | FALSE |
| MDMA/ecstasy and cannabis users | Never used drugs | 0.0598 | 0.9999 | -0.2801 | 0.3998 | FALSE | increased conflict at home | FALSE |
| MDMA/ecstasy and cannabis users | Psychedelics and cannabis users | 0.0561 | 1 | -0.3514 | 0.4636 | FALSE | increased conflict at home | FALSE |
| MDMA/ecstasy and cannabis users | Unknown/Unwilling to disclose | 0.012 | 1 | -0.3294 | 0.3533 | FALSE | increased conflict at home | FALSE |
| MDMA/ecstasy and cannabis users | Users of other drugs | -0.0376 | 1 | -0.4367 | 0.3614 | FALSE | increased conflict at home | FALSE |
| Never used drugs | Psychedelics and cannabis users | -0.0037 | 1 | -0.2305 | 0.223 | FALSE | increased conflict at home | FALSE |
| Never used drugs | Unknown/Unwilling to disclose | -0.0479 | 0.0178 | -0.0913 | -0.0044 | TRUE | increased conflict at home | FALSE |
| Never used drugs | Users of other drugs | -0.0975 | 0.9078 | -0.3087 | 0.1138 | FALSE | increased conflict at home | FALSE |
| Psychedelics and cannabis users | Unknown/Unwilling to disclose | -0.0441 | 0.9998 | -0.273 | 0.1847 | FALSE | increased conflict at home | FALSE |
| Psychedelics and cannabis users | Users of other drugs | -0.0937 | 0.9943 | -0.4021 | 0.2147 | FALSE | increased conflict at home | FALSE |
| Unknown/Unwilling to disclose | Users of other drugs | -0.0496 | 0.9993 | -0.2631 | 0.1639 | FALSE | increased conflict at home | FALSE |
| Cannabis and cocaine users | Cannabis users | 0.1523 | 0.3929 | -0.0578 | 0.3624 | FALSE | improved environment | FALSE |
| Cannabis and cocaine users | Cocaine users | 0.2621 | 0.0728 | -0.0111 | 0.5352 | FALSE | improved environment | FALSE |
| Cannabis and cocaine users | Drug use history but no pandemic use | 0.2069 | 0.0289 | 0.0108 | 0.4029 | TRUE | improved environment | FALSE |
| Cannabis and cocaine users | Extreme polydrug users | -0.0019 | 1 | -0.6082 | 0.6045 | FALSE | improved environment | FALSE |
| Cannabis and cocaine users | MDMA/ecstasy and cannabis users | 0.0472 | 1 | -0.3519 | 0.4464 | FALSE | improved environment | FALSE |
| Cannabis and cocaine users | Never used drugs | 0.3134 | 0 | 0.121 | 0.5059 | TRUE | improved environment | FALSE |
| Cannabis and cocaine users | Psychedelics and cannabis users | 0.1311 | 0.9348 | -0.1704 | 0.4326 | FALSE | improved environment | FALSE |
| Cannabis and cocaine users | Unknown/Unwilling to disclose | 0.2175 | 0.0153 | 0.0224 | 0.4126 | TRUE | improved environment | FALSE |
| Cannabis and cocaine users | Users of other drugs | 0.1638 | 0.7403 | -0.1254 | 0.4531 | FALSE | improved environment | FALSE |
| Cannabis users | Cocaine users | 0.1098 | 0.8359 | -0.1039 | 0.3235 | FALSE | improved environment | FALSE |
| Cannabis users | Drug use history but no pandemic use | 0.0546 | 0.752 | -0.0428 | 0.1519 | FALSE | improved environment | FALSE |
| Cannabis users | Extreme polydrug users | -0.1542 | 0.998 | -0.7362 | 0.4278 | FALSE | improved environment | FALSE |
| Cannabis users | MDMA/ecstasy and cannabis users | -0.1051 | 0.9959 | -0.4661 | 0.256 | FALSE | improved environment | FALSE |
| Cannabis users | Never used drugs | 0.1611 | 0 | 0.0712 | 0.2511 | TRUE | improved environment | FALSE |
| Cannabis users | Psychedelics and cannabis users | -0.0212 | 1 | -0.2701 | 0.2277 | FALSE | improved environment | FALSE |
| Cannabis users | Unknown/Unwilling to disclose | 0.0652 | 0.4825 | -0.0302 | 0.1606 | FALSE | improved environment | FALSE |
| Cannabis users | Users of other drugs | 0.0115 | 1 | -0.2223 | 0.2454 | FALSE | improved environment | FALSE |
| Cocaine users | Drug use history but no pandemic use | -0.0552 | 0.9972 | -0.2551 | 0.1447 | FALSE | improved environment | FALSE |
| Cocaine users | Extreme polydrug users | -0.264 | 0.9351 | -0.8716 | 0.3436 | FALSE | improved environment | FALSE |
| Cocaine users | MDMA/ecstasy and cannabis users | -0.2149 | 0.7987 | -0.6159 | 0.1862 | FALSE | improved environment | FALSE |
| Cocaine users | Never used drugs | 0.0514 | 0.9982 | -0.1451 | 0.2478 | FALSE | improved environment | FALSE |
| Cocaine users | Psychedelics and cannabis users | -0.131 | 0.9383 | -0.435 | 0.1731 | FALSE | improved environment | FALSE |
| Cocaine users | Unknown/Unwilling to disclose | -0.0446 | 0.9995 | -0.2435 | 0.1544 | FALSE | improved environment | FALSE |
| Cocaine users | Users of other drugs | -0.0982 | 0.9879 | -0.3901 | 0.1936 | FALSE | improved environment | FALSE |
| Drug use history but no pandemic use | Extreme polydrug users | -0.2087 | 0.9801 | -0.7858 | 0.3683 | FALSE | improved environment | FALSE |
| Drug use history but no pandemic use | MDMA/ecstasy and cannabis users | -0.1596 | 0.9179 | -0.5127 | 0.1934 | FALSE | improved environment | FALSE |
| Drug use history but no pandemic use | Never used drugs | 0.1066 | 0 | 0.0577 | 0.1555 | TRUE | improved environment | FALSE |
| Drug use history but no pandemic use | Psychedelics and cannabis users | -0.0758 | 0.9917 | -0.3129 | 0.1614 | FALSE | improved environment | FALSE |
| Drug use history but no pandemic use | Unknown/Unwilling to disclose | 0.0106 | 0.9999 | -0.0477 | 0.069 | FALSE | improved environment | FALSE |
| Drug use history but no pandemic use | Users of other drugs | -0.043 | 0.9998 | -0.2643 | 0.1783 | FALSE | improved environment | FALSE |
| Extreme polydrug users | MDMA/ecstasy and cannabis users | 0.0491 | 1 | -0.6246 | 0.7228 | FALSE | improved environment | FALSE |
| Extreme polydrug users | Never used drugs | 0.3153 | 0.777 | -0.2605 | 0.8912 | FALSE | improved environment | FALSE |
| Extreme polydrug users | Psychedelics and cannabis users | 0.133 | 0.9996 | -0.4879 | 0.7539 | FALSE | improved environment | FALSE |
| Extreme polydrug users | Unknown/Unwilling to disclose | 0.2194 | 0.972 | -0.3573 | 0.7961 | FALSE | improved environment | FALSE |
| Extreme polydrug users | Users of other drugs | 0.1657 | 0.9977 | -0.4493 | 0.7807 | FALSE | improved environment | FALSE |
| MDMA/ecstasy and cannabis users | Never used drugs | 0.2662 | 0.3263 | -0.0849 | 0.6173 | FALSE | improved environment | FALSE |
| MDMA/ecstasy and cannabis users | Psychedelics and cannabis users | 0.0839 | 0.9998 | -0.337 | 0.5047 | FALSE | improved environment | FALSE |
| MDMA/ecstasy and cannabis users | Unknown/Unwilling to disclose | 0.1703 | 0.8812 | -0.1823 | 0.5228 | FALSE | improved environment | FALSE |
| MDMA/ecstasy and cannabis users | Users of other drugs | 0.1166 | 0.9966 | -0.2955 | 0.5288 | FALSE | improved environment | FALSE |
| Never used drugs | Psychedelics and cannabis users | -0.1823 | 0.2888 | -0.4166 | 0.0519 | FALSE | improved environment | FALSE |
| Never used drugs | Unknown/Unwilling to disclose | -0.0959 | 0 | -0.1408 | -0.051 | TRUE | improved environment | FALSE |
| Never used drugs | Users of other drugs | -0.1496 | 0.4782 | -0.3678 | 0.0686 | FALSE | improved environment | FALSE |
| Psychedelics and cannabis users | Unknown/Unwilling to disclose | 0.0864 | 0.9786 | -0.15 | 0.3228 | FALSE | improved environment | FALSE |
| Psychedelics and cannabis users | Users of other drugs | 0.0327 | 1 | -0.2858 | 0.3513 | FALSE | improved environment | FALSE |
| Unknown/Unwilling to disclose | Users of other drugs | -0.0537 | 0.999 | -0.2741 | 0.1668 | FALSE | improved environment | FALSE |
| Cannabis and cocaine users | Cannabis users | 0.0781 | 0.9765 | -0.1327 | 0.2889 | FALSE | spending more time with loved ones | FALSE |
| Cannabis and cocaine users | Cocaine users | 0.1142 | 0.9497 | -0.1599 | 0.3883 | FALSE | spending more time with loved ones | FALSE |
| Cannabis and cocaine users | Drug use history but no pandemic use | 0.0887 | 0.919 | -0.108 | 0.2855 | FALSE | spending more time with loved ones | FALSE |
| Cannabis and cocaine users | Extreme polydrug users | -0.2668 | 0.9314 | -0.8753 | 0.3416 | FALSE | spending more time with loved ones | FALSE |
| Cannabis and cocaine users | MDMA/ecstasy and cannabis users | 0.1863 | 0.9034 | -0.2142 | 0.5868 | FALSE | spending more time with loved ones | FALSE |
| Cannabis and cocaine users | Never used drugs | 0.158 | 0.2231 | -0.0351 | 0.3511 | FALSE | spending more time with loved ones | FALSE |
| Cannabis and cocaine users | Psychedelics and cannabis users | -0.0212 | 1 | -0.3238 | 0.2813 | FALSE | spending more time with loved ones | FALSE |
| Cannabis and cocaine users | Unknown/Unwilling to disclose | 0.0986 | 0.852 | -0.0972 | 0.2943 | FALSE | spending more time with loved ones | FALSE |
| Cannabis and cocaine users | Users of other drugs | 0.1405 | 0.8797 | -0.1497 | 0.4307 | FALSE | spending more time with loved ones | FALSE |
| Cannabis users | Cocaine users | 0.0361 | 1 | -0.1783 | 0.2505 | FALSE | spending more time with loved ones | FALSE |
| Cannabis users | Drug use history but no pandemic use | 0.0106 | 1 | -0.087 | 0.1083 | FALSE | spending more time with loved ones | FALSE |
| Cannabis users | Extreme polydrug users | -0.3449 | 0.6904 | -0.9289 | 0.2391 | FALSE | spending more time with loved ones | FALSE |
| Cannabis users | MDMA/ecstasy and cannabis users | 0.1082 | 0.995 | -0.2541 | 0.4705 | FALSE | spending more time with loved ones | FALSE |
| Cannabis users | Never used drugs | 0.0799 | 0.1359 | -0.0103 | 0.1701 | FALSE | spending more time with loved ones | FALSE |
| Cannabis users | Psychedelics and cannabis users | -0.0994 | 0.9625 | -0.3491 | 0.1504 | FALSE | spending more time with loved ones | FALSE |
| Cannabis users | Unknown/Unwilling to disclose | 0.0205 | 0.9996 | -0.0752 | 0.1161 | FALSE | spending more time with loved ones | FALSE |
| Cannabis users | Users of other drugs | 0.0624 | 0.9979 | -0.1723 | 0.297 | FALSE | spending more time with loved ones | FALSE |
| Cocaine users | Drug use history but no pandemic use | -0.0255 | 1 | -0.2261 | 0.1751 | FALSE | spending more time with loved ones | FALSE |
| Cocaine users | Extreme polydrug users | -0.3811 | 0.6151 | -0.9908 | 0.2287 | FALSE | spending more time with loved ones | FALSE |
| Cocaine users | MDMA/ecstasy and cannabis users | 0.0721 | 0.9999 | -0.3303 | 0.4745 | FALSE | spending more time with loved ones | FALSE |
| Cocaine users | Never used drugs | 0.0438 | 0.9995 | -0.1533 | 0.2409 | FALSE | spending more time with loved ones | FALSE |
| Cocaine users | Psychedelics and cannabis users | -0.1355 | 0.9261 | -0.4405 | 0.1696 | FALSE | spending more time with loved ones | FALSE |
| Cocaine users | Unknown/Unwilling to disclose | -0.0157 | 1 | -0.2153 | 0.184 | FALSE | spending more time with loved ones | FALSE |
| Cocaine users | Users of other drugs | 0.0263 | 1 | -0.2666 | 0.3191 | FALSE | spending more time with loved ones | FALSE |
| Drug use history but no pandemic use | Extreme polydrug users | -0.3556 | 0.6395 | -0.9346 | 0.2235 | FALSE | spending more time with loved ones | FALSE |
| Drug use history but no pandemic use | MDMA/ecstasy and cannabis users | 0.0976 | 0.9973 | -0.2567 | 0.4519 | FALSE | spending more time with loved ones | FALSE |
| Drug use history but no pandemic use | Never used drugs | 0.0692 | 0.0003 | 0.0202 | 0.1183 | TRUE | spending more time with loved ones | FALSE |
| Drug use history but no pandemic use | Psychedelics and cannabis users | -0.11 | 0.9069 | -0.348 | 0.128 | FALSE | spending more time with loved ones | FALSE |
| Drug use history but no pandemic use | Unknown/Unwilling to disclose | 0.0098 | 1 | -0.0487 | 0.0683 | FALSE | spending more time with loved ones | FALSE |
| Drug use history but no pandemic use | Users of other drugs | 0.0517 | 0.9993 | -0.1704 | 0.2738 | FALSE | spending more time with loved ones | FALSE |
| Extreme polydrug users | MDMA/ecstasy and cannabis users | 0.4532 | 0.5126 | -0.2229 | 1.1292 | FALSE | spending more time with loved ones | FALSE |
| Extreme polydrug users | Never used drugs | 0.4248 | 0.3719 | -0.153 | 1.0027 | FALSE | spending more time with loved ones | FALSE |
| Extreme polydrug users | Psychedelics and cannabis users | 0.2456 | 0.9647 | -0.3774 | 0.8686 | FALSE | spending more time with loved ones | FALSE |
| Extreme polydrug users | Unknown/Unwilling to disclose | 0.3654 | 0.6008 | -0.2133 | 0.9441 | FALSE | spending more time with loved ones | FALSE |
| Extreme polydrug users | Users of other drugs | 0.4073 | 0.5359 | -0.2098 | 1.0244 | FALSE | spending more time with loved ones | FALSE |
| MDMA/ecstasy and cannabis users | Never used drugs | -0.0283 | 1 | -0.3806 | 0.324 | FALSE | spending more time with loved ones | FALSE |
| MDMA/ecstasy and cannabis users | Psychedelics and cannabis users | -0.2076 | 0.8696 | -0.6299 | 0.2148 | FALSE | spending more time with loved ones | FALSE |
| MDMA/ecstasy and cannabis users | Unknown/Unwilling to disclose | -0.0878 | 0.9988 | -0.4415 | 0.266 | FALSE | spending more time with loved ones | FALSE |
| MDMA/ecstasy and cannabis users | Users of other drugs | -0.0458 | 1 | -0.4594 | 0.3677 | FALSE | spending more time with loved ones | FALSE |
| Never used drugs | Psychedelics and cannabis users | -0.1792 | 0.318 | -0.4143 | 0.0558 | FALSE | spending more time with loved ones | FALSE |
| Never used drugs | Unknown/Unwilling to disclose | -0.0594 | 0.0012 | -0.1045 | -0.0144 | TRUE | spending more time with loved ones | FALSE |
| Never used drugs | Users of other drugs | -0.0175 | 1 | -0.2365 | 0.2014 | FALSE | spending more time with loved ones | FALSE |
| Psychedelics and cannabis users | Unknown/Unwilling to disclose | 0.1198 | 0.8496 | -0.1174 | 0.357 | FALSE | spending more time with loved ones | FALSE |
| Psychedelics and cannabis users | Users of other drugs | 0.1617 | 0.8482 | -0.1579 | 0.4814 | FALSE | spending more time with loved ones | FALSE |
| Unknown/Unwilling to disclose | Users of other drugs | 0.0419 | 0.9999 | -0.1793 | 0.2632 | FALSE | spending more time with loved ones | FALSE |

**Supp Table 6. Resilience post-hoc.**

Part V – Regressing out the effects of confounds

- 1. From mental health varibles

OLS Regression Results

==============================================================================

Dep. Variable: anxiety_score R-squared: 0.425

Model: OLS Adj. R-squared: 0.424

Method: Least Squares F-statistic: 438.2

Date: Tue, 11 Apr 2023 Prob (F-statistic): 0.00

Time: 20:01:52 Log-Likelihood: -51767.

No. Observations: 37383 AIC: 1.037e+05

Df Residuals: 37319 BIC: 1.042e+05

Df Model: 63

Covariance Type: nonrobust

==============================================================================================================================

coef std err t P>|t| [0.025 0.975]

------------------------------------------------------------------------------------------------------------------------------

Intercept 3.0992 0.085 36.403 0.000 2.932 3.266

C(timepoint)[T.june2021] -0.0648 0.010 -6.374 0.000 -0.085 -0.045

C(Age_decade)[T.20] -0.0870 0.038 -2.309 0.021 -0.161 -0.013

C(Age_decade)[T.30] -0.1485 0.041 -3.640 0.000 -0.228 -0.069

C(Age_decade)[T.40] -0.2445 0.040 -6.056 0.000 -0.324 -0.165

C(Age_decade)[T.50] -0.4032 0.040 -9.997 0.000 -0.482 -0.324

C(Age_decade)[T.60] -0.5024 0.042 -11.841 0.000 -0.586 -0.419

C(Age_decade)[T.70] -0.5134 0.047 -11.000 0.000 -0.605 -0.422

C(Age_decade)[T.80] -0.5656 0.072 -7.807 0.000 -0.708 -0.424

C(Residence)[T.United Kingdom] 0.0159 0.018 0.885 0.376 -0.019 0.051

C(Sex)[T.Male] -0.1246 0.011 -10.974 0.000 -0.147 -0.102

C(Sex)[T.Other] 0.0606 0.071 0.858 0.391 -0.078 0.199

C(Education)[T.PhD] 0.0607 0.024 2.547 0.011 0.014 0.107

C(Education)[T.School] -0.0510 0.011 -4.448 0.000 -0.073 -0.029

C(Education)[T.preGCSE] -0.0542 0.041 -1.310 0.190 -0.135 0.027

C(Ethnicity)[T.Asian or Asian British] -0.1531 0.059 -2.591 0.010 -0.269 -0.037

C(Ethnicity)[T.Black, Black British, Caribbean or African] -0.0836 0.095 -0.879 0.380 -0.270 0.103

C(Ethnicity)[T.Mixed or multiple ethnic groups] -0.0664 0.061 -1.081 0.280 -0.187 0.054

C(Ethnicity)[T.Other ethnic group] -0.0328 0.097 -0.339 0.735 -0.223 0.157

C(Ethnicity)[T.White] -0.0541 0.053 -1.030 0.303 -0.157 0.049

C(Occupation)[T.Homemaker] -0.3994 0.052 -7.623 0.000 -0.502 -0.297

C(Occupation)[T.Retired] -0.4755 0.047 -10.202 0.000 -0.567 -0.384

C(Occupation)[T.Student] -0.3120 0.051 -6.127 0.000 -0.412 -0.212

C(Occupation)[T.Unemployed/Looking for work] -0.3490 0.052 -6.687 0.000 -0.451 -0.247

C(Occupation)[T.Worker] -0.3767 0.044 -8.535 0.000 -0.463 -0.290

C(Exercise)[T.Daily] -0.0559 0.019 -3.005 0.003 -0.092 -0.019

C(Exercise)[T.Hourly] 0.1380 0.104 1.330 0.183 -0.065 0.341

C(Exercise)[T.More Often] -0.0215 0.085 -0.254 0.799 -0.188 0.145

C(Exercise)[T.Never] -0.0463 0.037 -1.262 0.207 -0.118 0.026

C(Exercise)[T.Once or twice a week] -0.0523 0.020 -2.667 0.008 -0.091 -0.014

C(Exercise)[T.Several times a week] -0.0672 0.019 -3.602 0.000 -0.104 -0.031

C(Meditation)[T.Daily] 0.0229 0.026 0.884 0.376 -0.028 0.074

C(Meditation)[T.Hourly] 0.2906 0.223 1.302 0.193 -0.147 0.728

C(Meditation)[T.More Often] -0.4111 0.224 -1.838 0.066 -0.850 0.027

C(Meditation)[T.Never] -0.1093 0.012 -9.104 0.000 -0.133 -0.086

C(Meditation)[T.Once or twice a week] -0.0065 0.019 -0.351 0.726 -0.043 0.030

C(Meditation)[T.Several times a week] 0.0327 0.026 1.254 0.210 -0.018 0.084

C(Reading)[T.Daily] -0.0536 0.014 -3.724 0.000 -0.082 -0.025

C(Reading)[T.Hourly] -0.0013 0.070 -0.018 0.986 -0.138 0.136

C(Reading)[T.More Often] -0.1531 0.115 -1.329 0.184 -0.379 0.073

C(Reading)[T.Never] 0.0469 0.022 2.153 0.031 0.004 0.090

C(Reading)[T.Once or twice a week] -0.0330 0.015 -2.177 0.029 -0.063 -0.003

C(Reading)[T.Several times a week] -0.0478 0.017 -2.877 0.004 -0.080 -0.015

C(OnlineGambling)[T.Daily] 0.1451 0.057 2.545 0.011 0.033 0.257

C(OnlineGambling)[T.Hourly] -0.0195 0.212 -0.092 0.927 -0.436 0.397

C(OnlineGambling)[T.More Often] -0.0189 0.344 -0.055 0.956 -0.693 0.655

C(OnlineGambling)[T.Never] -0.0057 0.016 -0.368 0.713 -0.036 0.025

C(OnlineGambling)[T.Once or twice a week] 0.0178 0.026 0.680 0.496 -0.034 0.069

C(OnlineGambling)[T.Several times a week] 0.0882 0.048 1.848 0.065 -0.005 0.182

C(HoursOnline)[T.2-4h] -0.1661 0.021 -7.740 0.000 -0.208 -0.124

C(HoursOnline)[T.4-6h] -0.1168 0.022 -5.416 0.000 -0.159 -0.075

C(HoursOnline)[T.6-8h] -0.0358 0.024 -1.502 0.133 -0.082 0.011

C(HoursOnline)[T.8-10h] -0.0477 0.024 -1.970 0.049 -0.095 -0.000

C(HoursOnline)[T.less than 2] -0.2122 0.028 -7.531 0.000 -0.267 -0.157

C(HoursOnline)[T.more than 12h] 0.0663 0.036 1.821 0.069 -0.005 0.138

cigarettes_z 0.0019 0.005 0.370 0.711 -0.008 0.012

alcohol_z 0.0271 0.005 5.106 0.000 0.017 0.037

openness 0.0972 0.006 16.672 0.000 0.086 0.109

extraversion -0.0794 0.005 -14.533 0.000 -0.090 -0.069

neuroticism 0.7368 0.006 114.269 0.000 0.724 0.749

conscienciousness -0.1191 0.007 -17.414 0.000 -0.133 -0.106

agreeableness 0.0132 0.007 2.016 0.044 0.000 0.026

perfectionism 0.1530 0.010 14.937 0.000 0.133 0.173

reward_drive 0.0780 0.007 10.868 0.000 0.064 0.092

==============================================================================

Omnibus: 2291.073 Durbin-Watson: 2.016

Prob(Omnibus): 0.000 Jarque-Bera (JB): 3125.117

Skew: 0.557 Prob(JB): 0.00

Kurtosis: 3.874 Cond. No. 162.

==============================================================================

Notes:

[1] Standard Errors assume that the covariance matrix of the errors is correctly specified.

OLS Regression Results

=========================================================================================

Dep. Variable: unable_to_stop_worrying_score R-squared: 0.436

Model: OLS Adj. R-squared: 0.435

Method: Least Squares F-statistic: 457.1

Date: Tue, 11 Apr 2023 Prob (F-statistic): 0.00

Time: 20:01:55 Log-Likelihood: -52426.

No. Observations: 37388 AIC: 1.050e+05

Df Residuals: 37324 BIC: 1.055e+05

Df Model: 63

Covariance Type: nonrobust

==============================================================================================================================

coef std err t P>|t| [0.025 0.975]

------------------------------------------------------------------------------------------------------------------------------

Intercept 2.5181 0.087 29.081 0.000 2.348 2.688

C(timepoint)[T.june2021] -0.0486 0.010 -4.697 0.000 -0.069 -0.028

C(Age_decade)[T.20] -0.0676 0.038 -1.762 0.078 -0.143 0.008

C(Age_decade)[T.30] -0.1374 0.042 -3.311 0.001 -0.219 -0.056

C(Age_decade)[T.40] -0.2115 0.041 -5.149 0.000 -0.292 -0.131

C(Age_decade)[T.50] -0.3153 0.041 -7.683 0.000 -0.396 -0.235

C(Age_decade)[T.60] -0.4058 0.043 -9.400 0.000 -0.490 -0.321

C(Age_decade)[T.70] -0.3970 0.047 -8.359 0.000 -0.490 -0.304

C(Age_decade)[T.80] -0.3988 0.074 -5.410 0.000 -0.543 -0.254

C(Residence)[T.United Kingdom] 0.0028 0.018 0.151 0.880 -0.033 0.039

C(Sex)[T.Male] -0.1590 0.012 -13.769 0.000 -0.182 -0.136

C(Sex)[T.Other] 0.0351 0.072 0.489 0.625 -0.106 0.176

C(Education)[T.PhD] 0.0251 0.024 1.035 0.301 -0.022 0.073

C(Education)[T.School] 0.0189 0.012 1.622 0.105 -0.004 0.042

C(Education)[T.preGCSE] 0.0270 0.042 0.641 0.521 -0.056 0.110

C(Ethnicity)[T.Asian or Asian British] 0.0038 0.060 0.064 0.949 -0.114 0.122

C(Ethnicity)[T.Black, Black British, Caribbean or African] -0.0123 0.097 -0.127 0.899 -0.202 0.177

C(Ethnicity)[T.Mixed or multiple ethnic groups] -0.0718 0.062 -1.149 0.250 -0.194 0.051

C(Ethnicity)[T.Other ethnic group] 0.1151 0.099 1.167 0.243 -0.078 0.308

C(Ethnicity)[T.White] -0.0377 0.053 -0.706 0.480 -0.142 0.067

C(Occupation)[T.Homemaker] -0.3614 0.053 -6.782 0.000 -0.466 -0.257

C(Occupation)[T.Retired] -0.4071 0.047 -8.591 0.000 -0.500 -0.314

C(Occupation)[T.Student] -0.2736 0.052 -5.284 0.000 -0.375 -0.172

C(Occupation)[T.Unemployed/Looking for work] -0.3379 0.053 -6.366 0.000 -0.442 -0.234

C(Occupation)[T.Worker] -0.3453 0.045 -7.696 0.000 -0.433 -0.257

C(Exercise)[T.Daily] -0.0441 0.019 -2.329 0.020 -0.081 -0.007

C(Exercise)[T.Hourly] 0.1481 0.106 1.402 0.161 -0.059 0.355

C(Exercise)[T.More Often] 0.0411 0.086 0.477 0.634 -0.128 0.210

C(Exercise)[T.Never] -0.0260 0.037 -0.695 0.487 -0.099 0.047

C(Exercise)[T.Once or twice a week] -0.0553 0.020 -2.771 0.006 -0.094 -0.016

C(Exercise)[T.Several times a week] -0.0532 0.019 -2.804 0.005 -0.090 -0.016

C(Meditation)[T.Daily] 0.0077 0.026 0.292 0.771 -0.044 0.059

C(Meditation)[T.Hourly] 0.2956 0.227 1.301 0.193 -0.150 0.741

C(Meditation)[T.More Often] -0.1075 0.228 -0.472 0.637 -0.554 0.339

C(Meditation)[T.Never] -0.0969 0.012 -7.932 0.000 -0.121 -0.073

C(Meditation)[T.Once or twice a week] -0.0055 0.019 -0.290 0.772 -0.043 0.032

C(Meditation)[T.Several times a week] 0.0189 0.027 0.714 0.475 -0.033 0.071

C(Reading)[T.Daily] -0.0458 0.015 -3.131 0.002 -0.075 -0.017

C(Reading)[T.Hourly] 0.0149 0.071 0.210 0.834 -0.124 0.154

C(Reading)[T.More Often] 0.0368 0.117 0.314 0.754 -0.193 0.267

C(Reading)[T.Never] 0.0584 0.022 2.635 0.008 0.015 0.102

C(Reading)[T.Once or twice a week] -0.0086 0.015 -0.555 0.579 -0.039 0.022

C(Reading)[T.Several times a week] -0.0602 0.017 -3.567 0.000 -0.093 -0.027

C(OnlineGambling)[T.Daily] 0.1052 0.058 1.812 0.070 -0.009 0.219

C(OnlineGambling)[T.Hourly] 0.2171 0.216 1.005 0.315 -0.206 0.641

C(OnlineGambling)[T.More Often] 0.1107 0.350 0.316 0.752 -0.575 0.797

C(OnlineGambling)[T.Never] -0.0317 0.016 -2.007 0.045 -0.063 -0.001

C(OnlineGambling)[T.Once or twice a week] 0.0316 0.027 1.184 0.236 -0.021 0.084

C(OnlineGambling)[T.Several times a week] 0.0896 0.049 1.845 0.065 -0.006 0.185

C(HoursOnline)[T.2-4h] -0.1283 0.022 -5.875 0.000 -0.171 -0.086

C(HoursOnline)[T.4-6h] -0.0752 0.022 -3.427 0.001 -0.118 -0.032

C(HoursOnline)[T.6-8h] 0.0009 0.024 0.037 0.970 -0.047 0.048

C(HoursOnline)[T.8-10h] -0.0268 0.025 -1.089 0.276 -0.075 0.021

C(HoursOnline)[T.less than 2] -0.1644 0.029 -5.732 0.000 -0.221 -0.108

C(HoursOnline)[T.more than 12h] 0.0776 0.037 2.097 0.036 0.005 0.150

cigarettes_z 0.0066 0.005 1.253 0.210 -0.004 0.017

alcohol_z 0.0149 0.005 2.756 0.006 0.004 0.025

openness 0.0783 0.006 13.195 0.000 0.067 0.090

extraversion -0.0573 0.006 -10.316 0.000 -0.068 -0.046

neuroticism 0.7687 0.007 117.163 0.000 0.756 0.782

conscienciousness -0.1354 0.007 -19.446 0.000 -0.149 -0.122

agreeableness -0.0136 0.007 -2.052 0.040 -0.027 -0.001

perfectionism 0.2255 0.010 21.638 0.000 0.205 0.246

reward_drive 0.0958 0.007 13.125 0.000 0.082 0.110

==============================================================================

Omnibus: 3460.783 Durbin-Watson: 2.013

Prob(Omnibus): 0.000 Jarque-Bera (JB): 5444.686

Skew: 0.696 Prob(JB): 0.00

Kurtosis: 4.247 Cond. No. 162.

==============================================================================

Notes:

[1] Standard Errors assume that the covariance matrix of the errors is correctly specified.

OLS Regression Results

============================================================================================

Dep. Variable: worrying_about_many_things_score R-squared: 0.444

Model: OLS Adj. R-squared: 0.443

Method: Least Squares F-statistic: 473.9

Date: Tue, 11 Apr 2023 Prob (F-statistic): 0.00

Time: 20:01:58 Log-Likelihood: -51701.

No. Observations: 37389 AIC: 1.035e+05

Df Residuals: 37325 BIC: 1.041e+05

Df Model: 63

Covariance Type: nonrobust

==============================================================================================================================

coef std err t P>|t| [0.025 0.975]

------------------------------------------------------------------------------------------------------------------------------

Intercept 2.6470 0.085 31.170 0.000 2.481 2.813

C(timepoint)[T.june2021] -0.0502 0.010 -4.953 0.000 -0.070 -0.030

C(Age_decade)[T.20] -0.0587 0.038 -1.561 0.119 -0.132 0.015

C(Age_decade)[T.30] -0.1232 0.041 -3.027 0.002 -0.203 -0.043

C(Age_decade)[T.40] -0.2083 0.040 -5.170 0.000 -0.287 -0.129

C(Age_decade)[T.50] -0.3346 0.040 -8.315 0.000 -0.413 -0.256

C(Age_decade)[T.60] -0.4133 0.042 -9.762 0.000 -0.496 -0.330

C(Age_decade)[T.70] -0.4259 0.047 -9.144 0.000 -0.517 -0.335

C(Age_decade)[T.80] -0.4385 0.072 -6.065 0.000 -0.580 -0.297

C(Residence)[T.United Kingdom] 0.0118 0.018 0.656 0.512 -0.023 0.047

C(Sex)[T.Male] -0.1368 0.011 -12.081 0.000 -0.159 -0.115

C(Sex)[T.Other] -0.0077 0.070 -0.110 0.913 -0.146 0.130

C(Education)[T.PhD] 0.0118 0.024 0.498 0.618 -0.035 0.058

C(Education)[T.School] 0.0122 0.011 1.065 0.287 -0.010 0.035

C(Education)[T.preGCSE] 0.0114 0.041 0.276 0.782 -0.070 0.092

C(Ethnicity)[T.Asian or Asian British] 0.0097 0.059 0.165 0.869 -0.106 0.125

C(Ethnicity)[T.Black, Black British, Caribbean or African] 0.0855 0.095 0.901 0.368 -0.101 0.272

C(Ethnicity)[T.Mixed or multiple ethnic groups] 0.0091 0.061 0.148 0.883 -0.111 0.129

C(Ethnicity)[T.Other ethnic group] 0.1630 0.097 1.686 0.092 -0.026 0.352

C(Ethnicity)[T.White] 0.0002 0.052 0.003 0.997 -0.102 0.103

C(Occupation)[T.Homemaker] -0.3298 0.052 -6.311 0.000 -0.432 -0.227

C(Occupation)[T.Retired] -0.4122 0.046 -8.869 0.000 -0.503 -0.321

C(Occupation)[T.Student] -0.2824 0.051 -5.561 0.000 -0.382 -0.183

C(Occupation)[T.Unemployed/Looking for work] -0.3391 0.052 -6.514 0.000 -0.441 -0.237

C(Occupation)[T.Worker] -0.3332 0.044 -7.573 0.000 -0.419 -0.247

C(Exercise)[T.Daily] -0.0533 0.019 -2.871 0.004 -0.090 -0.017

C(Exercise)[T.Hourly] 0.0621 0.104 0.599 0.549 -0.141 0.265

C(Exercise)[T.More Often] 0.1096 0.085 1.296 0.195 -0.056 0.275

C(Exercise)[T.Never] -0.0074 0.037 -0.202 0.840 -0.079 0.064

C(Exercise)[T.Once or twice a week] -0.0580 0.020 -2.963 0.003 -0.096 -0.020

C(Exercise)[T.Several times a week] -0.0628 0.019 -3.372 0.001 -0.099 -0.026

C(Meditation)[T.Daily] 0.0024 0.026 0.091 0.927 -0.048 0.053

C(Meditation)[T.Hourly] 0.4473 0.223 2.008 0.045 0.011 0.884

C(Meditation)[T.More Often] -0.1719 0.223 -0.770 0.441 -0.610 0.266

C(Meditation)[T.Never] -0.0941 0.012 -7.854 0.000 -0.118 -0.071

C(Meditation)[T.Once or twice a week] 0.0005 0.019 0.029 0.977 -0.036 0.037

C(Meditation)[T.Several times a week] 0.0051 0.026 0.197 0.844 -0.046 0.056

C(Reading)[T.Daily] -0.0563 0.014 -3.919 0.000 -0.084 -0.028

C(Reading)[T.Hourly] -0.0065 0.070 -0.093 0.926 -0.143 0.130

C(Reading)[T.More Often] -0.1533 0.115 -1.333 0.182 -0.379 0.072

C(Reading)[T.Never] 0.0342 0.022 1.574 0.116 -0.008 0.077

C(Reading)[T.Once or twice a week] -0.0142 0.015 -0.941 0.347 -0.044 0.015

C(Reading)[T.Several times a week] -0.0604 0.017 -3.649 0.000 -0.093 -0.028

C(OnlineGambling)[T.Daily] 0.0759 0.057 1.333 0.183 -0.036 0.187

C(OnlineGambling)[T.Hourly] 0.0610 0.212 0.288 0.773 -0.354 0.476

C(OnlineGambling)[T.More Often] -0.2727 0.343 -0.794 0.427 -0.946 0.400

C(OnlineGambling)[T.Never] -0.0593 0.015 -3.831 0.000 -0.090 -0.029

C(OnlineGambling)[T.Once or twice a week] 0.0121 0.026 0.464 0.643 -0.039 0.063

C(OnlineGambling)[T.Several times a week] 0.0960 0.048 2.016 0.044 0.003 0.189

C(HoursOnline)[T.2-4h] -0.1415 0.021 -6.606 0.000 -0.184 -0.100

C(HoursOnline)[T.4-6h] -0.0818 0.022 -3.799 0.000 -0.124 -0.040

C(HoursOnline)[T.6-8h] -0.0278 0.024 -1.169 0.242 -0.074 0.019

C(HoursOnline)[T.8-10h] -0.0449 0.024 -1.856 0.063 -0.092 0.003

C(HoursOnline)[T.less than 2] -0.1992 0.028 -7.085 0.000 -0.254 -0.144

C(HoursOnline)[T.more than 12h] 0.0843 0.036 2.322 0.020 0.013 0.155

cigarettes_z 0.0064 0.005 1.231 0.218 -0.004 0.017

alcohol_z 0.0130 0.005 2.464 0.014 0.003 0.023

openness 0.0825 0.006 14.183 0.000 0.071 0.094

extraversion -0.0550 0.005 -10.098 0.000 -0.066 -0.044

neuroticism 0.7597 0.006 118.061 0.000 0.747 0.772

conscienciousness -0.1300 0.007 -19.041 0.000 -0.143 -0.117

agreeableness 0.0093 0.007 1.424 0.154 -0.003 0.022

perfectionism 0.2380 0.010 23.283 0.000 0.218 0.258

reward_drive 0.0969 0.007 13.524 0.000 0.083 0.111

==============================================================================

Omnibus: 2736.172 Durbin-Watson: 2.018

Prob(Omnibus): 0.000 Jarque-Bera (JB): 4097.186

Skew: 0.598 Prob(JB): 0.00

Kurtosis: 4.095 Cond. No. 162.

==============================================================================

Notes:

[1] Standard Errors assume that the covariance matrix of the errors is correctly specified.

OLS Regression Results

=================================================================================

Dep. Variable: unable_to_relax_score R-squared: 0.354

Model: OLS Adj. R-squared: 0.353

Method: Least Squares F-statistic: 325.3

Date: Tue, 11 Apr 2023 Prob (F-statistic): 0.00

Time: 20:02:03 Log-Likelihood: -55030.

No. Observations: 37384 AIC: 1.102e+05

Df Residuals: 37320 BIC: 1.107e+05

Df Model: 63

Covariance Type: nonrobust

==============================================================================================================================

coef std err t P>|t| [0.025 0.975]

------------------------------------------------------------------------------------------------------------------------------

Intercept 2.8992 0.093 31.221 0.000 2.717 3.081

C(timepoint)[T.june2021] -0.0474 0.011 -4.279 0.000 -0.069 -0.026

C(Age_decade)[T.20] 0.0588 0.041 1.429 0.153 -0.022 0.139

C(Age_decade)[T.30] 0.0685 0.045 1.538 0.124 -0.019 0.156

C(Age_decade)[T.40] -0.0361 0.044 -0.820 0.412 -0.122 0.050

C(Age_decade)[T.50] -0.1543 0.044 -3.505 0.000 -0.241 -0.068

C(Age_decade)[T.60] -0.2504 0.046 -5.408 0.000 -0.341 -0.160

C(Age_decade)[T.70] -0.2830 0.051 -5.556 0.000 -0.383 -0.183

C(Age_decade)[T.80] -0.3245 0.079 -4.104 0.000 -0.479 -0.170

C(Residence)[T.United Kingdom] 0.0123 0.020 0.628 0.530 -0.026 0.051

C(Sex)[T.Male] -0.0970 0.012 -7.836 0.000 -0.121 -0.073

C(Sex)[T.Other] 0.2627 0.077 3.412 0.001 0.112 0.414

C(Education)[T.PhD] 0.0667 0.026 2.566 0.010 0.016 0.118

C(Education)[T.School] -0.0315 0.013 -2.516 0.012 -0.056 -0.007

C(Education)[T.preGCSE] -0.0480 0.045 -1.062 0.288 -0.137 0.041

C(Ethnicity)[T.Asian or Asian British] -0.1382 0.064 -2.146 0.032 -0.264 -0.012

C(Ethnicity)[T.Black, Black British, Caribbean or African] -0.1420 0.104 -1.368 0.171 -0.345 0.061

C(Ethnicity)[T.Mixed or multiple ethnic groups] -0.0486 0.067 -0.725 0.469 -0.180 0.083

C(Ethnicity)[T.Other ethnic group] -0.0107 0.106 -0.102 0.919 -0.218 0.196

C(Ethnicity)[T.White] -0.0142 0.057 -0.249 0.804 -0.126 0.098

C(Occupation)[T.Homemaker] -0.4816 0.057 -8.429 0.000 -0.594 -0.370

C(Occupation)[T.Retired] -0.6064 0.051 -11.932 0.000 -0.706 -0.507

C(Occupation)[T.Student] -0.4439 0.056 -7.995 0.000 -0.553 -0.335

C(Occupation)[T.Unemployed/Looking for work] -0.5157 0.057 -9.061 0.000 -0.627 -0.404

C(Occupation)[T.Worker] -0.4992 0.048 -10.376 0.000 -0.593 -0.405

C(Exercise)[T.Daily] -0.1064 0.020 -5.240 0.000 -0.146 -0.067

C(Exercise)[T.Hourly] 0.0958 0.113 0.846 0.397 -0.126 0.318

C(Exercise)[T.More Often] -0.1433 0.092 -1.550 0.121 -0.325 0.038

C(Exercise)[T.Never] -0.0464 0.040 -1.158 0.247 -0.125 0.032

C(Exercise)[T.Once or twice a week] -0.1171 0.021 -5.474 0.000 -0.159 -0.075

C(Exercise)[T.Several times a week] -0.1087 0.020 -5.342 0.000 -0.149 -0.069

C(Meditation)[T.Daily] -0.1086 0.028 -3.845 0.000 -0.164 -0.053

C(Meditation)[T.Hourly] -0.0487 0.244 -0.200 0.841 -0.526 0.429

C(Meditation)[T.More Often] -0.8349 0.244 -3.420 0.001 -1.313 -0.356

C(Meditation)[T.Never] -0.1270 0.013 -9.692 0.000 -0.153 -0.101

C(Meditation)[T.Once or twice a week] 0.0155 0.020 0.760 0.447 -0.024 0.055

C(Meditation)[T.Several times a week] 0.0007 0.028 0.024 0.981 -0.055 0.056

C(Reading)[T.Daily] -0.0698 0.016 -4.447 0.000 -0.101 -0.039

C(Reading)[T.Hourly] -0.1345 0.076 -1.765 0.078 -0.284 0.015

C(Reading)[T.More Often] 0.0083 0.126 0.066 0.947 -0.238 0.255

C(Reading)[T.Never] 0.0274 0.024 1.154 0.249 -0.019 0.074

C(Reading)[T.Once or twice a week] -0.0229 0.017 -1.383 0.167 -0.055 0.010

C(Reading)[T.Several times a week] -0.0515 0.018 -2.845 0.004 -0.087 -0.016

C(OnlineGambling)[T.Daily] 0.0669 0.062 1.075 0.283 -0.055 0.189

C(OnlineGambling)[T.Hourly] -0.1828 0.232 -0.789 0.430 -0.637 0.271

C(OnlineGambling)[T.More Often] -0.1661 0.375 -0.442 0.658 -0.902 0.570

C(OnlineGambling)[T.Never] -0.0487 0.017 -2.874 0.004 -0.082 -0.015

C(OnlineGambling)[T.Once or twice a week] -0.0062 0.029 -0.217 0.828 -0.062 0.050

C(OnlineGambling)[T.Several times a week] 0.0484 0.052 0.930 0.352 -0.054 0.150

C(HoursOnline)[T.2-4h] -0.2143 0.023 -9.148 0.000 -0.260 -0.168

C(HoursOnline)[T.4-6h] -0.1685 0.024 -7.160 0.000 -0.215 -0.122

C(HoursOnline)[T.6-8h] -0.0862 0.026 -3.315 0.001 -0.137 -0.035

C(HoursOnline)[T.8-10h] -0.0632 0.026 -2.391 0.017 -0.115 -0.011

C(HoursOnline)[T.less than 2] -0.2832 0.031 -9.209 0.000 -0.343 -0.223

C(HoursOnline)[T.more than 12h] 0.0956 0.040 2.409 0.016 0.018 0.173

cigarettes_z 0.0205 0.006 3.625 0.000 0.009 0.032

alcohol_z 0.0052 0.006 0.894 0.372 -0.006 0.017

openness 0.0775 0.006 12.181 0.000 0.065 0.090

extraversion -0.0317 0.006 -5.318 0.000 -0.043 -0.020

neuroticism 0.6403 0.007 91.007 0.000 0.627 0.654

conscienciousness -0.1041 0.007 -13.942 0.000 -0.119 -0.089

agreeableness -0.0476 0.007 -6.690 0.000 -0.062 -0.034

perfectionism 0.2259 0.011 20.214 0.000 0.204 0.248

reward_drive 0.1253 0.008 15.999 0.000 0.110 0.141

==============================================================================

Omnibus: 2469.333 Durbin-Watson: 2.001

Prob(Omnibus): 0.000 Jarque-Bera (JB): 3269.320

Skew: 0.602 Prob(JB): 0.00

Kurtosis: 3.806 Cond. No. 162.

==============================================================================

Notes:

[1] Standard Errors assume that the covariance matrix of the errors is correctly specified.

OLS Regression Results

==============================================================================

Dep. Variable: restlessness_score R-squared: 0.210

Model: OLS Adj. R-squared: 0.209

Method: Least Squares F-statistic: 157.9

Date: Tue, 11 Apr 2023 Prob (F-statistic): 0.00

Time: 20:02:07 Log-Likelihood: -56878.

No. Observations: 37389 AIC: 1.139e+05

Df Residuals: 37325 BIC: 1.144e+05

Df Model: 63

Covariance Type: nonrobust

==============================================================================================================================

coef std err t P>|t| [0.025 0.975]

------------------------------------------------------------------------------------------------------------------------------

Intercept 2.0757 0.098 21.282 0.000 1.884 2.267

C(timepoint)[T.june2021] -0.0326 0.012 -2.801 0.005 -0.055 -0.010

C(Age_decade)[T.20] -0.0606 0.043 -1.404 0.160 -0.145 0.024

C(Age_decade)[T.30] -0.1608 0.047 -3.439 0.001 -0.252 -0.069

C(Age_decade)[T.40] -0.2226 0.046 -4.812 0.000 -0.313 -0.132

C(Age_decade)[T.50] -0.3162 0.046 -6.840 0.000 -0.407 -0.226

C(Age_decade)[T.60] -0.3621 0.049 -7.448 0.000 -0.457 -0.267

C(Age_decade)[T.70] -0.4053 0.053 -7.577 0.000 -0.510 -0.300

C(Age_decade)[T.80] -0.4432 0.083 -5.337 0.000 -0.606 -0.280

C(Residence)[T.United Kingdom] 0.0473 0.021 2.291 0.022 0.007 0.088

C(Sex)[T.Male] 0.0090 0.013 0.695 0.487 -0.016 0.035

C(Sex)[T.Other] 0.1433 0.081 1.772 0.076 -0.015 0.302

C(Education)[T.PhD] -0.0668 0.027 -2.451 0.014 -0.120 -0.013

C(Education)[T.School] 0.0739 0.013 5.626 0.000 0.048 0.100

C(Education)[T.preGCSE] 0.0892 0.047 1.879 0.060 -0.004 0.182

C(Ethnicity)[T.Asian or Asian British] -0.0920 0.068 -1.360 0.174 -0.225 0.041

C(Ethnicity)[T.Black, Black British, Caribbean or African] -0.3264 0.109 -2.994 0.003 -0.540 -0.113

C(Ethnicity)[T.Mixed or multiple ethnic groups] -0.0678 0.070 -0.963 0.335 -0.206 0.070

C(Ethnicity)[T.Other ethnic group] 0.0166 0.111 0.149 0.881 -0.201 0.234

C(Ethnicity)[T.White] 0.0077 0.060 0.128 0.898 -0.110 0.126

C(Occupation)[T.Homemaker] -0.4735 0.060 -7.889 0.000 -0.591 -0.356

C(Occupation)[T.Retired] -0.5640 0.053 -10.565 0.000 -0.669 -0.459

C(Occupation)[T.Student] -0.4050 0.058 -6.943 0.000 -0.519 -0.291

C(Occupation)[T.Unemployed/Looking for work] -0.4761 0.060 -7.964 0.000 -0.593 -0.359

C(Occupation)[T.Worker] -0.4738 0.051 -9.376 0.000 -0.573 -0.375

C(Exercise)[T.Daily] 0.0748 0.021 3.504 0.000 0.033 0.117

C(Exercise)[T.Hourly] 0.4604 0.119 3.870 0.000 0.227 0.694

C(Exercise)[T.More Often] 0.2888 0.097 2.973 0.003 0.098 0.479

C(Exercise)[T.Never] -0.0131 0.042 -0.310 0.756 -0.096 0.069

C(Exercise)[T.Once or twice a week] -0.0507 0.022 -2.256 0.024 -0.095 -0.007

C(Exercise)[T.Several times a week] -0.0027 0.021 -0.127 0.899 -0.045 0.039

C(Meditation)[T.Daily] -0.0778 0.030 -2.622 0.009 -0.136 -0.020

C(Meditation)[T.Hourly] -0.0968 0.256 -0.378 0.705 -0.598 0.405

C(Meditation)[T.More Often] -0.2913 0.256 -1.136 0.256 -0.794 0.211

C(Meditation)[T.Never] -0.1256 0.014 -9.124 0.000 -0.153 -0.099

C(Meditation)[T.Once or twice a week] -0.0040 0.021 -0.188 0.851 -0.046 0.038

C(Meditation)[T.Several times a week] -0.0211 0.030 -0.705 0.481 -0.080 0.038

C(Reading)[T.Daily] -0.0128 0.016 -0.779 0.436 -0.045 0.019

C(Reading)[T.Hourly] 0.0165 0.080 0.206 0.837 -0.140 0.173

C(Reading)[T.More Often] 0.2504 0.132 1.896 0.058 -0.008 0.509

C(Reading)[T.Never] 0.0822 0.025 3.294 0.001 0.033 0.131

C(Reading)[T.Once or twice a week] 0.0114 0.017 0.659 0.510 -0.023 0.045

C(Reading)[T.Several times a week] -0.0306 0.019 -1.607 0.108 -0.068 0.007

C(OnlineGambling)[T.Daily] 0.1068 0.065 1.633 0.103 -0.021 0.235

C(OnlineGambling)[T.Hourly] -0.2697 0.243 -1.108 0.268 -0.747 0.207

C(OnlineGambling)[T.More Often] 0.1582 0.394 0.401 0.688 -0.615 0.931

C(OnlineGambling)[T.Never] -0.0205 0.018 -1.153 0.249 -0.055 0.014

C(OnlineGambling)[T.Once or twice a week] -0.0086 0.030 -0.288 0.774 -0.068 0.050

C(OnlineGambling)[T.Several times a week] 0.0781 0.055 1.429 0.153 -0.029 0.185

C(HoursOnline)[T.2-4h] -0.1058 0.025 -4.302 0.000 -0.154 -0.058

C(HoursOnline)[T.4-6h] -0.0703 0.025 -2.846 0.004 -0.119 -0.022

C(HoursOnline)[T.6-8h] -0.0432 0.027 -1.583 0.113 -0.097 0.010

C(HoursOnline)[T.8-10h] -0.0155 0.028 -0.559 0.576 -0.070 0.039

C(HoursOnline)[T.less than 2] -0.1487 0.032 -4.604 0.000 -0.212 -0.085

C(HoursOnline)[T.more than 12h] 0.1795 0.042 4.306 0.000 0.098 0.261

cigarettes_z 0.0266 0.006 4.476 0.000 0.015 0.038

alcohol_z 0.0002 0.006 0.030 0.976 -0.012 0.012

openness 0.0942 0.007 14.105 0.000 0.081 0.107

extraversion 0.0217 0.006 3.458 0.001 0.009 0.034

neuroticism 0.3721 0.007 50.348 0.000 0.358 0.387

conscienciousness -0.1505 0.008 -19.199 0.000 -0.166 -0.135

agreeableness -0.0560 0.007 -7.483 0.000 -0.071 -0.041

perfectionism 0.2946 0.012 25.100 0.000 0.272 0.318

reward_drive 0.1361 0.008 16.547 0.000 0.120 0.152

==============================================================================

Omnibus: 5705.923 Durbin-Watson: 1.998

Prob(Omnibus): 0.000 Jarque-Bera (JB): 9699.205

Skew: 1.015 Prob(JB): 0.00

Kurtosis: 4.452 Cond. No. 162.

==============================================================================

Notes:

[1] Standard Errors assume that the covariance matrix of the errors is correctly specified.

OLS Regression Results

==============================================================================

Dep. Variable: irritability_score R-squared: 0.306

Model: OLS Adj. R-squared: 0.305

Method: Least Squares F-statistic: 261.7

Date: Tue, 11 Apr 2023 Prob (F-statistic): 0.00

Time: 20:02:11 Log-Likelihood: -52072.

No. Observations: 37389 AIC: 1.043e+05

Df Residuals: 37325 BIC: 1.048e+05

Df Model: 63

Covariance Type: nonrobust

==============================================================================================================================

coef std err t P>|t| [0.025 0.975]

------------------------------------------------------------------------------------------------------------------------------

Intercept 2.7977 0.086 32.619 0.000 2.630 2.966

C(timepoint)[T.june2021] -0.0435 0.010 -4.250 0.000 -0.064 -0.023

C(Age_decade)[T.20] -0.1413 0.038 -3.719 0.000 -0.216 -0.067

C(Age_decade)[T.30] -0.0645 0.041 -1.570 0.117 -0.145 0.016

C(Age_decade)[T.40] -0.1610 0.041 -3.957 0.000 -0.241 -0.081

C(Age_decade)[T.50] -0.3934 0.041 -9.678 0.000 -0.473 -0.314

C(Age_decade)[T.60] -0.5050 0.043 -11.810 0.000 -0.589 -0.421

C(Age_decade)[T.70] -0.5205 0.047 -11.066 0.000 -0.613 -0.428

C(Age_decade)[T.80] -0.5574 0.073 -7.633 0.000 -0.701 -0.414

C(Residence)[T.United Kingdom] 0.0107 0.018 0.590 0.555 -0.025 0.046

C(Sex)[T.Male] -0.0331 0.011 -2.893 0.004 -0.056 -0.011

C(Sex)[T.Other] 0.1476 0.071 2.076 0.038 0.008 0.287

C(Education)[T.PhD] -0.0327 0.024 -1.365 0.172 -0.080 0.014

C(Education)[T.School] -0.0079 0.012 -0.681 0.496 -0.031 0.015

C(Education)[T.preGCSE] 0.0764 0.042 1.830 0.067 -0.005 0.158

C(Ethnicity)[T.Asian or Asian British] -0.0612 0.059 -1.029 0.304 -0.178 0.055

C(Ethnicity)[T.Black, Black British, Caribbean or African] -0.2080 0.096 -2.169 0.030 -0.396 -0.020

C(Ethnicity)[T.Mixed or multiple ethnic groups] -0.0681 0.062 -1.100 0.271 -0.189 0.053

C(Ethnicity)[T.Other ethnic group] -0.0725 0.098 -0.742 0.458 -0.264 0.119

C(Ethnicity)[T.White] -0.0843 0.053 -1.593 0.111 -0.188 0.019

C(Occupation)[T.Homemaker] -0.0731 0.053 -1.385 0.166 -0.177 0.030

C(Occupation)[T.Retired] -0.3034 0.047 -6.464 0.000 -0.395 -0.211

C(Occupation)[T.Student] -0.3172 0.051 -6.184 0.000 -0.418 -0.217

C(Occupation)[T.Unemployed/Looking for work] -0.2166 0.053 -4.119 0.000 -0.320 -0.114

C(Occupation)[T.Worker] -0.2505 0.044 -5.636 0.000 -0.338 -0.163

C(Exercise)[T.Daily] -0.1026 0.019 -5.469 0.000 -0.139 -0.066

C(Exercise)[T.Hourly] 0.2067 0.105 1.976 0.048 0.002 0.412

C(Exercise)[T.More Often] -0.0404 0.085 -0.473 0.636 -0.208 0.127

C(Exercise)[T.Never] 0.0499 0.037 1.348 0.178 -0.023 0.122

C(Exercise)[T.Once or twice a week] -0.1008 0.020 -5.102 0.000 -0.140 -0.062

C(Exercise)[T.Several times a week] -0.1008 0.019 -5.364 0.000 -0.138 -0.064

C(Meditation)[T.Daily] -0.1012 0.026 -3.878 0.000 -0.152 -0.050

C(Meditation)[T.Hourly] -0.0745 0.225 -0.331 0.741 -0.515 0.367

C(Meditation)[T.More Often] -0.3998 0.225 -1.773 0.076 -0.842 0.042

C(Meditation)[T.Never] -0.0626 0.012 -5.175 0.000 -0.086 -0.039

C(Meditation)[T.Once or twice a week] -0.0484 0.019 -2.578 0.010 -0.085 -0.012

C(Meditation)[T.Several times a week] -0.0081 0.026 -0.310 0.757 -0.060 0.043

C(Reading)[T.Daily] -0.0087 0.015 -0.598 0.550 -0.037 0.020

C(Reading)[T.Hourly] -0.0125 0.070 -0.177 0.860 -0.150 0.126

C(Reading)[T.More Often] 0.2409 0.116 2.074 0.038 0.013 0.468

C(Reading)[T.Never] 0.0587 0.022 2.672 0.008 0.016 0.102

C(Reading)[T.Once or twice a week] -0.0066 0.015 -0.434 0.665 -0.037 0.023

C(Reading)[T.Several times a week] -0.0377 0.017 -2.253 0.024 -0.070 -0.005

C(OnlineGambling)[T.Daily] -0.0002 0.057 -0.003 0.998 -0.113 0.113

C(OnlineGambling)[T.Hourly] 0.2225 0.214 1.040 0.298 -0.197 0.642

C(OnlineGambling)[T.More Often] 0.5114 0.347 1.475 0.140 -0.168 1.191

C(OnlineGambling)[T.Never] -0.0526 0.016 -3.365 0.001 -0.083 -0.022

C(OnlineGambling)[T.Once or twice a week] -0.0042 0.026 -0.159 0.874 -0.056 0.048

C(OnlineGambling)[T.Several times a week] 0.1190 0.048 2.476 0.013 0.025 0.213

C(HoursOnline)[T.2-4h] -0.1246 0.022 -5.760 0.000 -0.167 -0.082

C(HoursOnline)[T.4-6h] -0.0592 0.022 -2.726 0.006 -0.102 -0.017

C(HoursOnline)[T.6-8h] -0.0341 0.024 -1.421 0.155 -0.081 0.013

C(HoursOnline)[T.8-10h] -0.0428 0.024 -1.753 0.080 -0.091 0.005

C(HoursOnline)[T.less than 2] -0.1913 0.028 -6.736 0.000 -0.247 -0.136

C(HoursOnline)[T.more than 12h] -0.0166 0.037 -0.453 0.650 -0.088 0.055

cigarettes_z -0.0208 0.005 -3.970 0.000 -0.031 -0.011

alcohol_z 0.0344 0.005 6.438 0.000 0.024 0.045

openness 0.0299 0.006 5.084 0.000 0.018 0.041

extraversion 0.0128 0.006 2.318 0.020 0.002 0.024

neuroticism 0.4678 0.006 71.987 0.000 0.455 0.481

conscienciousness -0.1253 0.007 -18.169 0.000 -0.139 -0.112

agreeableness -0.2067 0.007 -31.419 0.000 -0.220 -0.194

perfectionism 0.2592 0.010 25.110 0.000 0.239 0.279

reward_drive 0.0682 0.007 9.424 0.000 0.054 0.082

==============================================================================

Omnibus: 2503.635 Durbin-Watson: 1.999

Prob(Omnibus): 0.000 Jarque-Bera (JB): 3448.771

Skew: 0.591 Prob(JB): 0.00

Kurtosis: 3.904 Cond. No. 162.

==============================================================================

Notes:

[1] Standard Errors assume that the covariance matrix of the errors is correctly specified.

OLS Regression Results

======================================================================================

Dep. Variable: negative_premonition_score R-squared: 0.317

Model: OLS Adj. R-squared: 0.316

Method: Least Squares F-statistic: 274.9

Date: Tue, 11 Apr 2023 Prob (F-statistic): 0.00

Time: 20:02:14 Log-Likelihood: -54918.

No. Observations: 37389 AIC: 1.100e+05

Df Residuals: 37325 BIC: 1.105e+05

Df Model: 63

Covariance Type: nonrobust

==============================================================================================================================

coef std err t P>|t| [0.025 0.975]

------------------------------------------------------------------------------------------------------------------------------

Intercept 2.0975 0.093 22.662 0.000 1.916 2.279

C(timepoint)[T.june2021] -0.0794 0.011 -7.180 0.000 -0.101 -0.058

C(Age_decade)[T.20] 0.0192 0.041 0.468 0.640 -0.061 0.100

C(Age_decade)[T.30] 0.0145 0.044 0.326 0.744 -0.072 0.101

C(Age_decade)[T.40] -0.0457 0.044 -1.041 0.298 -0.132 0.040

C(Age_decade)[T.50] -0.1446 0.044 -3.298 0.001 -0.231 -0.059

C(Age_decade)[T.60] -0.2086 0.046 -4.520 0.000 -0.299 -0.118

C(Age_decade)[T.70] -0.2476 0.051 -4.879 0.000 -0.347 -0.148

C(Age_decade)[T.80] -0.2938 0.079 -3.729 0.000 -0.448 -0.139

C(Residence)[T.United Kingdom] 0.0078 0.020 0.396 0.692 -0.031 0.046

C(Sex)[T.Male] -0.1478 0.012 -11.973 0.000 -0.172 -0.124

C(Sex)[T.Other] 0.1450 0.077 1.889 0.059 -0.005 0.295

C(Education)[T.PhD] 0.0276 0.026 1.065 0.287 -0.023 0.078

C(Education)[T.School] 0.0500 0.012 4.010 0.000 0.026 0.074

C(Education)[T.preGCSE] 0.0937 0.045 2.080 0.038 0.005 0.182

C(Ethnicity)[T.Asian or Asian British] 0.0625 0.064 0.974 0.330 -0.063 0.188

C(Ethnicity)[T.Black, Black British, Caribbean or African] 0.0737 0.103 0.713 0.476 -0.129 0.277

C(Ethnicity)[T.Mixed or multiple ethnic groups] -0.0706 0.067 -1.058 0.290 -0.202 0.060

C(Ethnicity)[T.Other ethnic group] 0.1492 0.105 1.416 0.157 -0.057 0.356

C(Ethnicity)[T.White] -0.0129 0.057 -0.226 0.821 -0.125 0.099

C(Occupation)[T.Homemaker] -0.2711 0.057 -4.760 0.000 -0.383 -0.159

C(Occupation)[T.Retired] -0.4008 0.051 -7.913 0.000 -0.500 -0.302

C(Occupation)[T.Student] -0.3522 0.055 -6.364 0.000 -0.461 -0.244

C(Occupation)[T.Unemployed/Looking for work] -0.3678 0.057 -6.482 0.000 -0.479 -0.257

C(Occupation)[T.Worker] -0.3880 0.048 -8.090 0.000 -0.482 -0.294

C(Exercise)[T.Daily] -0.0475 0.020 -2.345 0.019 -0.087 -0.008

C(Exercise)[T.Hourly] 0.0787 0.113 0.697 0.486 -0.143 0.300

C(Exercise)[T.More Often] -0.0167 0.092 -0.182 0.856 -0.197 0.164

C(Exercise)[T.Never] 0.0253 0.040 0.633 0.527 -0.053 0.104

C(Exercise)[T.Once or twice a week] -0.0600 0.021 -2.812 0.005 -0.102 -0.018

C(Exercise)[T.Several times a week] -0.0834 0.020 -4.110 0.000 -0.123 -0.044

C(Meditation)[T.Daily] -0.0477 0.028 -1.692 0.091 -0.103 0.008

C(Meditation)[T.Hourly] 0.4015 0.243 1.654 0.098 -0.074 0.877

C(Meditation)[T.More Often] -0.1032 0.243 -0.424 0.672 -0.580 0.374

C(Meditation)[T.Never] -0.0990 0.013 -7.577 0.000 -0.125 -0.073

C(Meditation)[T.Once or twice a week] 0.0010 0.020 0.048 0.962 -0.039 0.041

C(Meditation)[T.Several times a week] 0.0392 0.028 1.381 0.167 -0.016 0.095

C(Reading)[T.Daily] -0.0228 0.016 -1.459 0.145 -0.054 0.008

C(Reading)[T.Hourly] 0.0363 0.076 0.478 0.633 -0.113 0.185

C(Reading)[T.More Often] 0.0729 0.125 0.582 0.560 -0.173 0.319

C(Reading)[T.Never] 0.0740 0.024 3.125 0.002 0.028 0.120

C(Reading)[T.Once or twice a week] -0.0132 0.016 -0.801 0.423 -0.045 0.019

C(Reading)[T.Several times a week] -0.0667 0.018 -3.696 0.000 -0.102 -0.031

C(OnlineGambling)[T.Daily] 0.1384 0.062 2.230 0.026 0.017 0.260

C(OnlineGambling)[T.Hourly] 0.4057 0.231 1.757 0.079 -0.047 0.858

C(OnlineGambling)[T.More Often] -0.0762 0.374 -0.204 0.839 -0.809 0.657

C(OnlineGambling)[T.Never] -0.0569 0.017 -3.371 0.001 -0.090 -0.024

C(OnlineGambling)[T.Once or twice a week] 0.0696 0.028 2.441 0.015 0.014 0.125

C(OnlineGambling)[T.Several times a week] 0.1549 0.052 2.986 0.003 0.053 0.257

C(HoursOnline)[T.2-4h] -0.1270 0.023 -5.437 0.000 -0.173 -0.081

C(HoursOnline)[T.4-6h] -0.0659 0.023 -2.810 0.005 -0.112 -0.020

C(HoursOnline)[T.6-8h] 0.0082 0.026 0.317 0.751 -0.043 0.059

C(HoursOnline)[T.8-10h] -0.0133 0.026 -0.506 0.613 -0.065 0.038

C(HoursOnline)[T.less than 2] -0.1786 0.031 -5.826 0.000 -0.239 -0.118

C(HoursOnline)[T.more than 12h] 0.1025 0.040 2.591 0.010 0.025 0.180

cigarettes_z 0.0158 0.006 2.790 0.005 0.005 0.027

alcohol_z 0.0114 0.006 1.976 0.048 8.94e-05 0.023

openness 0.1142 0.006 18.015 0.000 0.102 0.127

extraversion -0.0390 0.006 -6.572 0.000 -0.051 -0.027

neuroticism 0.6006 0.007 85.636 0.000 0.587 0.614

conscienciousness -0.1540 0.007 -20.700 0.000 -0.169 -0.139

agreeableness -0.0236 0.007 -3.320 0.001 -0.037 -0.010

perfectionism 0.2104 0.011 18.884 0.000 0.189 0.232

reward_drive 0.1085 0.008 13.904 0.000 0.093 0.124

==============================================================================

Omnibus: 4891.474 Durbin-Watson: 2.013

Prob(Omnibus): 0.000 Jarque-Bera (JB): 8455.144

Skew: 0.879 Prob(JB): 0.00

Kurtosis: 4.529 Cond. No. 162.

==============================================================================

Notes:

[1] Standard Errors assume that the covariance matrix of the errors is correctly specified.

OLS Regression Results

==============================================================================

Dep. Variable: apathy_score R-squared: 0.273

Model: OLS Adj. R-squared: 0.272

Method: Least Squares F-statistic: 222.3

Date: Tue, 11 Apr 2023 Prob (F-statistic): 0.00

Time: 20:02:19 Log-Likelihood: -54700.

No. Observations: 37380 AIC: 1.095e+05

Df Residuals: 37316 BIC: 1.101e+05

Df Model: 63

Covariance Type: nonrobust

==============================================================================================================================

coef std err t P>|t| [0.025 0.975]

------------------------------------------------------------------------------------------------------------------------------

Intercept 2.7714 0.092 30.071 0.000 2.591 2.952

C(timepoint)[T.june2021] -0.0493 0.011 -4.487 0.000 -0.071 -0.028

C(Age_decade)[T.20] -0.1686 0.041 -4.129 0.000 -0.249 -0.089

C(Age_decade)[T.30] -0.2080 0.044 -4.707 0.000 -0.295 -0.121

C(Age_decade)[T.40] -0.2735 0.044 -6.256 0.000 -0.359 -0.188

C(Age_decade)[T.50] -0.3554 0.044 -8.136 0.000 -0.441 -0.270

C(Age_decade)[T.60] -0.4253 0.046 -9.258 0.000 -0.515 -0.335

C(Age_decade)[T.70] -0.4780 0.051 -9.459 0.000 -0.577 -0.379

C(Age_decade)[T.80] -0.5291 0.078 -6.750 0.000 -0.683 -0.375

C(Residence)[T.United Kingdom] 0.0055 0.019 0.280 0.780 -0.033 0.044

C(Sex)[T.Male] 0.0111 0.012 0.907 0.364 -0.013 0.035

C(Sex)[T.Other] 0.1382 0.076 1.811 0.070 -0.011 0.288

C(Education)[T.PhD] -0.0206 0.026 -0.802 0.423 -0.071 0.030

C(Education)[T.School] 0.0293 0.012 2.366 0.018 0.005 0.054

C(Education)[T.preGCSE] 0.0733 0.045 1.637 0.102 -0.014 0.161

C(Ethnicity)[T.Asian or Asian British] 0.0095 0.064 0.149 0.882 -0.116 0.135

C(Ethnicity)[T.Black, Black British, Caribbean or African] -0.0913 0.103 -0.887 0.375 -0.293 0.110

C(Ethnicity)[T.Mixed or multiple ethnic groups] 0.0036 0.067 0.054 0.957 -0.127 0.134

C(Ethnicity)[T.Other ethnic group] 0.0704 0.105 0.671 0.502 -0.135 0.276

C(Ethnicity)[T.White] 0.0162 0.057 0.284 0.776 -0.095 0.128

C(Occupation)[T.Homemaker] -0.5181 0.057 -9.146 0.000 -0.629 -0.407

C(Occupation)[T.Retired] -0.6172 0.050 -12.251 0.000 -0.716 -0.518

C(Occupation)[T.Student] -0.5280 0.055 -9.590 0.000 -0.636 -0.420

C(Occupation)[T.Unemployed/Looking for work] -0.3983 0.056 -7.058 0.000 -0.509 -0.288

C(Occupation)[T.Worker] -0.5819 0.048 -12.202 0.000 -0.675 -0.488

C(Exercise)[T.Daily] -0.3059 0.020 -15.191 0.000 -0.345 -0.266

C(Exercise)[T.Hourly] -0.1303 0.112 -1.161 0.246 -0.350 0.090

C(Exercise)[T.More Often] -0.1403 0.092 -1.530 0.126 -0.320 0.039

C(Exercise)[T.Never] 0.1345 0.040 3.386 0.001 0.057 0.212

C(Exercise)[T.Once or twice a week] -0.1983 0.021 -9.347 0.000 -0.240 -0.157

C(Exercise)[T.Several times a week] -0.2815 0.020 -13.954 0.000 -0.321 -0.242

C(Meditation)[T.Daily] -0.0544 0.028 -1.942 0.052 -0.109 0.001

C(Meditation)[T.Hourly] -0.0013 0.241 -0.005 0.996 -0.475 0.472

C(Meditation)[T.More Often] -0.2018 0.242 -0.834 0.404 -0.676 0.273

C(Meditation)[T.Never] -0.0884 0.013 -6.808 0.000 -0.114 -0.063

C(Meditation)[T.Once or twice a week] -0.0085 0.020 -0.422 0.673 -0.048 0.031

C(Meditation)[T.Several times a week] 0.0339 0.028 1.201 0.230 -0.021 0.089

C(Reading)[T.Daily] -0.1487 0.016 -9.554 0.000 -0.179 -0.118

C(Reading)[T.Hourly] -0.1819 0.076 -2.407 0.016 -0.330 -0.034

C(Reading)[T.More Often] -0.3163 0.125 -2.538 0.011 -0.561 -0.072

C(Reading)[T.Never] 0.1021 0.024 4.336 0.000 0.056 0.148

C(Reading)[T.Once or twice a week] -0.0888 0.016 -5.418 0.000 -0.121 -0.057

C(Reading)[T.Several times a week] -0.1186 0.018 -6.603 0.000 -0.154 -0.083

C(OnlineGambling)[T.Daily] 0.0182 0.062 0.295 0.768 -0.103 0.139

C(OnlineGambling)[T.Hourly] 0.5888 0.230 2.564 0.010 0.139 1.039

C(OnlineGambling)[T.More Often] 0.7067 0.372 1.899 0.058 -0.023 1.436

C(OnlineGambling)[T.Never] -0.0501 0.017 -2.982 0.003 -0.083 -0.017

C(OnlineGambling)[T.Once or twice a week] -0.0114 0.028 -0.401 0.688 -0.067 0.044

C(OnlineGambling)[T.Several times a week] 0.0384 0.052 0.744 0.457 -0.063 0.140

C(HoursOnline)[T.2-4h] -0.2431 0.023 -10.468 0.000 -0.289 -0.198

C(HoursOnline)[T.4-6h] -0.1673 0.023 -7.170 0.000 -0.213 -0.122

C(HoursOnline)[T.6-8h] -0.0970 0.026 -3.765 0.000 -0.148 -0.047

C(HoursOnline)[T.8-10h] -0.0575 0.026 -2.192 0.028 -0.109 -0.006

C(HoursOnline)[T.less than 2] -0.3001 0.030 -9.845 0.000 -0.360 -0.240

C(HoursOnline)[T.more than 12h] 0.0785 0.039 1.994 0.046 0.001 0.156

cigarettes_z 0.0526 0.006 9.360 0.000 0.042 0.064

alcohol_z 0.0252 0.006 4.400 0.000 0.014 0.036

openness 0.0157 0.006 2.498 0.012 0.003 0.028

extraversion -0.1111 0.006 -18.795 0.000 -0.123 -0.099

neuroticism 0.4004 0.007 57.401 0.000 0.387 0.414

conscienciousness -0.2285 0.007 -30.879 0.000 -0.243 -0.214

agreeableness -0.1132 0.007 -16.038 0.000 -0.127 -0.099

perfectionism 0.1927 0.011 17.394 0.000 0.171 0.214

reward_drive 0.1309 0.008 16.856 0.000 0.116 0.146

==============================================================================

Omnibus: 5746.233 Durbin-Watson: 2.007

Prob(Omnibus): 0.000 Jarque-Bera (JB): 10867.506

Skew: 0.969 Prob(JB): 0.00

Kurtosis: 4.795 Cond. No. 162.

==============================================================================

Notes:

[1] Standard Errors assume that the covariance matrix of the errors is correctly specified.

OLS Regression Results

==============================================================================

Dep. Variable: depression_score R-squared: 0.323

Model: OLS Adj. R-squared: 0.322

Method: Least Squares F-statistic: 283.0

Date: Tue, 11 Apr 2023 Prob (F-statistic): 0.00

Time: 20:02:23 Log-Likelihood: -53182.

No. Observations: 37389 AIC: 1.065e+05

Df Residuals: 37325 BIC: 1.070e+05

Df Model: 63

Covariance Type: nonrobust

==============================================================================================================================

coef std err t P>|t| [0.025 0.975]

------------------------------------------------------------------------------------------------------------------------------

Intercept 2.8288 0.088 32.016 0.000 2.656 3.002

C(timepoint)[T.june2021] -0.0868 0.011 -8.225 0.000 -0.107 -0.066

C(Age_decade)[T.20] -0.1638 0.039 -4.185 0.000 -0.240 -0.087

C(Age_decade)[T.30] -0.2065 0.042 -4.876 0.000 -0.290 -0.124

C(Age_decade)[T.40] -0.2632 0.042 -6.281 0.000 -0.345 -0.181

C(Age_decade)[T.50] -0.3384 0.042 -8.081 0.000 -0.420 -0.256

C(Age_decade)[T.60] -0.3779 0.044 -8.579 0.000 -0.464 -0.292

C(Age_decade)[T.70] -0.3671 0.048 -7.576 0.000 -0.462 -0.272

C(Age_decade)[T.80] -0.3325 0.075 -4.420 0.000 -0.480 -0.185

C(Residence)[T.United Kingdom] 0.0221 0.019 1.180 0.238 -0.015 0.059

C(Sex)[T.Male] -0.0113 0.012 -0.960 0.337 -0.034 0.012

C(Sex)[T.Other] 0.1284 0.073 1.752 0.080 -0.015 0.272

C(Education)[T.PhD] -0.0379 0.025 -1.533 0.125 -0.086 0.011

C(Education)[T.School] 0.0189 0.012 1.593 0.111 -0.004 0.042

C(Education)[T.preGCSE] 0.0311 0.043 0.722 0.470 -0.053 0.115

C(Ethnicity)[T.Asian or Asian British] -0.0234 0.061 -0.382 0.702 -0.144 0.097

C(Ethnicity)[T.Black, Black British, Caribbean or African] -0.0481 0.099 -0.487 0.626 -0.242 0.145

C(Ethnicity)[T.Mixed or multiple ethnic groups] -0.0064 0.064 -0.101 0.920 -0.131 0.119

C(Ethnicity)[T.Other ethnic group] 0.0355 0.101 0.353 0.724 -0.162 0.233

C(Ethnicity)[T.White] 0.0066 0.054 0.121 0.904 -0.100 0.113

C(Occupation)[T.Homemaker] -0.5890 0.054 -10.833 0.000 -0.696 -0.482

C(Occupation)[T.Retired] -0.6777 0.048 -14.014 0.000 -0.772 -0.583

C(Occupation)[T.Student] -0.5953 0.053 -11.267 0.000 -0.699 -0.492

C(Occupation)[T.Unemployed/Looking for work] -0.4341 0.054 -8.016 0.000 -0.540 -0.328

C(Occupation)[T.Worker] -0.6366 0.046 -13.905 0.000 -0.726 -0.547

C(Exercise)[T.Daily] -0.2115 0.019 -10.947 0.000 -0.249 -0.174

C(Exercise)[T.Hourly] -0.1233 0.108 -1.144 0.253 -0.334 0.088

C(Exercise)[T.More Often] -0.1043 0.088 -1.186 0.236 -0.277 0.068

C(Exercise)[T.Never] 0.1213 0.038 3.181 0.001 0.047 0.196

C(Exercise)[T.Once or twice a week] -0.1662 0.020 -8.162 0.000 -0.206 -0.126

C(Exercise)[T.Several times a week] -0.2043 0.019 -10.554 0.000 -0.242 -0.166

C(Meditation)[T.Daily] -0.0298 0.027 -1.108 0.268 -0.082 0.023

C(Meditation)[T.Hourly] 0.7751 0.232 3.344 0.001 0.321 1.229

C(Meditation)[T.More Often] -0.0358 0.232 -0.154 0.877 -0.491 0.419

C(Meditation)[T.Never] -0.0839 0.012 -6.730 0.000 -0.108 -0.059

C(Meditation)[T.Once or twice a week] 0.0005 0.019 0.028 0.977 -0.037 0.038

C(Meditation)[T.Several times a week] 0.0272 0.027 1.006 0.315 -0.026 0.080

C(Reading)[T.Daily] -0.0806 0.015 -5.397 0.000 -0.110 -0.051

C(Reading)[T.Hourly] 0.0204 0.073 0.281 0.779 -0.122 0.163

C(Reading)[T.More Often] -0.2579 0.120 -2.156 0.031 -0.492 -0.023

C(Reading)[T.Never] 0.0814 0.023 3.599 0.000 0.037 0.126

C(Reading)[T.Once or twice a week] -0.0546 0.016 -3.471 0.001 -0.085 -0.024

C(Reading)[T.Several times a week] -0.0700 0.017 -4.061 0.000 -0.104 -0.036

C(OnlineGambling)[T.Daily] 0.1237 0.059 2.088 0.037 0.008 0.240

C(OnlineGambling)[T.Hourly] -0.1614 0.220 -0.732 0.464 -0.593 0.271

C(OnlineGambling)[T.More Often] 0.0671 0.357 0.188 0.851 -0.633 0.767

C(OnlineGambling)[T.Never] -0.0264 0.016 -1.640 0.101 -0.058 0.005

C(OnlineGambling)[T.Once or twice a week] 0.0301 0.027 1.105 0.269 -0.023 0.083

C(OnlineGambling)[T.Several times a week] 0.1352 0.050 2.730 0.006 0.038 0.232

C(HoursOnline)[T.2-4h] -0.2283 0.022 -10.245 0.000 -0.272 -0.185

C(HoursOnline)[T.4-6h] -0.1471 0.022 -6.569 0.000 -0.191 -0.103

C(HoursOnline)[T.6-8h] -0.0849 0.025 -3.431 0.001 -0.133 -0.036

C(HoursOnline)[T.8-10h] -0.0463 0.025 -1.841 0.066 -0.096 0.003

C(HoursOnline)[T.less than 2] -0.2464 0.029 -8.421 0.000 -0.304 -0.189

C(HoursOnline)[T.more than 12h] 0.0459 0.038 1.214 0.225 -0.028 0.120

cigarettes_z 0.0332 0.005 6.169 0.000 0.023 0.044

alcohol_z 0.0363 0.006 6.586 0.000 0.025 0.047

openness 0.0730 0.006 12.061 0.000 0.061 0.085

extraversion -0.0966 0.006 -17.033 0.000 -0.108 -0.085

neuroticism 0.5685 0.007 84.916 0.000 0.555 0.582

conscienciousness -0.1857 0.007 -26.151 0.000 -0.200 -0.172

agreeableness -0.0607 0.007 -8.953 0.000 -0.074 -0.047

perfectionism 0.1692 0.011 15.909 0.000 0.148 0.190

reward_drive 0.0836 0.007 11.223 0.000 0.069 0.098

==============================================================================

Omnibus: 4841.811 Durbin-Watson: 2.010

Prob(Omnibus): 0.000 Jarque-Bera (JB): 8732.939

Skew: 0.854 Prob(JB): 0.00

Kurtosis: 4.640 Cond. No. 162.

==============================================================================

Notes:

[1] Standard Errors assume that the covariance matrix of the errors is correctly specified.

OLS Regression Results

==============================================================================

Dep. Variable: tiredness_score R-squared: 0.276

Model: OLS Adj. R-squared: 0.275

Method: Least Squares F-statistic: 226.0

Date: Tue, 11 Apr 2023 Prob (F-statistic): 0.00

Time: 20:02:26 Log-Likelihood: -57582.

No. Observations: 37389 AIC: 1.153e+05

Df Residuals: 37325 BIC: 1.158e+05

Df Model: 63

Covariance Type: nonrobust

==============================================================================================================================

coef std err t P>|t| [0.025 0.975]

------------------------------------------------------------------------------------------------------------------------------

Intercept 3.9651 0.099 39.896 0.000 3.770 4.160

C(timepoint)[T.june2021] 0.0872 0.012 7.349 0.000 0.064 0.110

C(Age_decade)[T.20] -0.1332 0.044 -3.027 0.002 -0.220 -0.047

C(Age_decade)[T.30] -0.0989 0.048 -2.075 0.038 -0.192 -0.005

C(Age_decade)[T.40] -0.2080 0.047 -4.412 0.000 -0.300 -0.116

C(Age_decade)[T.50] -0.3596 0.047 -7.635 0.000 -0.452 -0.267

C(Age_decade)[T.60] -0.4370 0.050 -8.821 0.000 -0.534 -0.340

C(Age_decade)[T.70] -0.3679 0.055 -6.750 0.000 -0.475 -0.261

C(Age_decade)[T.80] -0.0929 0.085 -1.097 0.272 -0.259 0.073

C(Residence)[T.United Kingdom] 0.0196 0.021 0.933 0.351 -0.022 0.061

C(Sex)[T.Male] -0.1612 0.013 -12.162 0.000 -0.187 -0.135

C(Sex)[T.Other] 0.1630 0.082 1.978 0.048 0.001 0.324

C(Education)[T.PhD] -0.0005 0.028 -0.019 0.985 -0.055 0.054

C(Education)[T.School] 0.0374 0.013 2.797 0.005 0.011 0.064

C(Education)[T.preGCSE] 0.0396 0.048 0.818 0.413 -0.055 0.134

C(Ethnicity)[T.Asian or Asian British] -0.2064 0.069 -2.995 0.003 -0.342 -0.071

C(Ethnicity)[T.Black, Black British, Caribbean or African] -0.3362 0.111 -3.026 0.002 -0.554 -0.118

C(Ethnicity)[T.Mixed or multiple ethnic groups] -0.0887 0.072 -1.237 0.216 -0.229 0.052

C(Ethnicity)[T.Other ethnic group] 0.0269 0.113 0.237 0.812 -0.195 0.249

C(Ethnicity)[T.White] -0.0333 0.061 -0.544 0.587 -0.153 0.087

C(Occupation)[T.Homemaker] -0.5680 0.061 -9.287 0.000 -0.688 -0.448

C(Occupation)[T.Retired] -0.8282 0.054 -15.225 0.000 -0.935 -0.722

C(Occupation)[T.Student] -0.7202 0.059 -12.119 0.000 -0.837 -0.604

C(Occupation)[T.Unemployed/Looking for work] -0.6955 0.061 -11.416 0.000 -0.815 -0.576

C(Occupation)[T.Worker] -0.7666 0.051 -14.887 0.000 -0.868 -0.666

C(Exercise)[T.Daily] -0.5985 0.022 -27.532 0.000 -0.641 -0.556

C(Exercise)[T.Hourly] -0.2875 0.121 -2.372 0.018 -0.525 -0.050

C(Exercise)[T.More Often] -0.6889 0.099 -6.960 0.000 -0.883 -0.495

C(Exercise)[T.Never] 0.1693 0.043 3.948 0.000 0.085 0.253

C(Exercise)[T.Once or twice a week] -0.3321 0.023 -14.503 0.000 -0.377 -0.287

C(Exercise)[T.Several times a week] -0.5112 0.022 -23.471 0.000 -0.554 -0.469

C(Meditation)[T.Daily] 0.0105 0.030 0.347 0.729 -0.049 0.070

C(Meditation)[T.Hourly] 0.3288 0.261 1.261 0.207 -0.182 0.840

C(Meditation)[T.More Often] -0.3295 0.261 -1.261 0.207 -0.842 0.183

C(Meditation)[T.Never] -0.0616 0.014 -4.395 0.000 -0.089 -0.034

C(Meditation)[T.Once or twice a week] 0.0140 0.022 0.643 0.521 -0.029 0.057

C(Meditation)[T.Several times a week] 0.0707 0.030 2.320 0.020 0.011 0.130

C(Reading)[T.Daily] -0.1123 0.017 -6.681 0.000 -0.145 -0.079

C(Reading)[T.Hourly] 0.1079 0.082 1.323 0.186 -0.052 0.268

C(Reading)[T.More Often] 0.2104 0.135 1.564 0.118 -0.053 0.474

C(Reading)[T.Never] 0.0758 0.025 2.981 0.003 0.026 0.126

C(Reading)[T.Once or twice a week] -0.0785 0.018 -4.436 0.000 -0.113 -0.044

C(Reading)[T.Several times a week] -0.0934 0.019 -4.820 0.000 -0.131 -0.055

C(OnlineGambling)[T.Daily] 0.0079 0.067 0.119 0.905 -0.123 0.139

C(OnlineGambling)[T.Hourly] -0.0347 0.248 -0.140 0.889 -0.521 0.451

C(OnlineGambling)[T.More Often] -0.6294 0.402 -1.567 0.117 -1.417 0.158

C(OnlineGambling)[T.Never] -0.0391 0.018 -2.157 0.031 -0.075 -0.004

C(OnlineGambling)[T.Once or twice a week] 0.0307 0.031 1.004 0.315 -0.029 0.091

C(OnlineGambling)[T.Several times a week] 0.1564 0.056 2.806 0.005 0.047 0.266

C(HoursOnline)[T.2-4h] -0.2826 0.025 -11.271 0.000 -0.332 -0.233

C(HoursOnline)[T.4-6h] -0.1565 0.025 -6.215 0.000 -0.206 -0.107

C(HoursOnline)[T.6-8h] -0.1033 0.028 -3.710 0.000 -0.158 -0.049

C(HoursOnline)[T.8-10h] -0.0652 0.028 -2.304 0.021 -0.121 -0.010

C(HoursOnline)[T.less than 2] -0.3239 0.033 -9.842 0.000 -0.388 -0.259

C(HoursOnline)[T.more than 12h] -0.0050 0.042 -0.118 0.906 -0.088 0.078

cigarettes_z 0.0355 0.006 5.851 0.000 0.024 0.047

alcohol_z -0.0051 0.006 -0.816 0.414 -0.017 0.007

openness 0.0648 0.007 9.516 0.000 0.051 0.078

extraversion -0.0860 0.006 -13.479 0.000 -0.098 -0.073

neuroticism 0.4362 0.008 57.927 0.000 0.421 0.451

conscienciousness -0.1954 0.008 -24.461 0.000 -0.211 -0.180

agreeableness -0.0212 0.008 -2.786 0.005 -0.036 -0.006

perfectionism 0.2178 0.012 18.212 0.000 0.194 0.241

reward_drive 0.0823 0.008 9.818 0.000 0.066 0.099

==============================================================================

Omnibus: 1827.382 Durbin-Watson: 2.017

Prob(Omnibus): 0.000 Jarque-Bera (JB): 2160.877

Skew: 0.538 Prob(JB): 0.00

Kurtosis: 3.479 Cond. No. 162.

==============================================================================

Notes:

[1] Standard Errors assume that the covariance matrix of the errors is correctly specified.

OLS Regression Results

========================================================================================

Dep. Variable: concentration_problems_score R-squared: 0.261

Model: OLS Adj. R-squared: 0.260

Method: Least Squares F-statistic: 209.4

Date: Tue, 11 Apr 2023 Prob (F-statistic): 0.00

Time: 20:02:30 Log-Likelihood: -56247.

No. Observations: 37389 AIC: 1.126e+05

Df Residuals: 37325 BIC: 1.132e+05

Df Model: 63

Covariance Type: nonrobust

==============================================================================================================================

coef std err t P>|t| [0.025 0.975]

------------------------------------------------------------------------------------------------------------------------------

Intercept 2.6722 0.096 27.864 0.000 2.484 2.860

C(timepoint)[T.june2021] 0.0079 0.011 0.689 0.491 -0.015 0.030

C(Age_decade)[T.20] 0.0030 0.042 0.070 0.944 -0.080 0.086

C(Age_decade)[T.30] -0.0331 0.046 -0.719 0.472 -0.123 0.057

C(Age_decade)[T.40] -0.1096 0.045 -2.410 0.016 -0.199 -0.020

C(Age_decade)[T.50] -0.2104 0.045 -4.629 0.000 -0.299 -0.121

C(Age_decade)[T.60] -0.2852 0.048 -5.964 0.000 -0.379 -0.191

C(Age_decade)[T.70] -0.3162 0.053 -6.011 0.000 -0.419 -0.213

C(Age_decade)[T.80] -0.3472 0.082 -4.253 0.000 -0.507 -0.187

C(Residence)[T.United Kingdom] 0.0500 0.020 2.465 0.014 0.010 0.090

C(Sex)[T.Male] -0.1008 0.013 -7.882 0.000 -0.126 -0.076

C(Sex)[T.Other] 0.2405 0.080 3.025 0.002 0.085 0.396

C(Education)[T.PhD] -0.0362 0.027 -1.351 0.177 -0.089 0.016

C(Education)[T.School] -0.0027 0.013 -0.210 0.833 -0.028 0.023

C(Education)[T.preGCSE] 0.0687 0.047 1.473 0.141 -0.023 0.160

C(Ethnicity)[T.Asian or Asian British] 0.0298 0.067 0.448 0.654 -0.101 0.160

C(Ethnicity)[T.Black, Black British, Caribbean or African] -0.0170 0.107 -0.158 0.874 -0.227 0.193

C(Ethnicity)[T.Mixed or multiple ethnic groups] 0.1322 0.069 1.910 0.056 -0.003 0.268

C(Ethnicity)[T.Other ethnic group] 0.0981 0.109 0.899 0.369 -0.116 0.312

C(Ethnicity)[T.White] 0.1238 0.059 2.095 0.036 0.008 0.240

C(Occupation)[T.Homemaker] -0.6027 0.059 -10.213 0.000 -0.718 -0.487

C(Occupation)[T.Retired] -0.6500 0.052 -12.384 0.000 -0.753 -0.547

C(Occupation)[T.Student] -0.4934 0.057 -8.603 0.000 -0.606 -0.381

C(Occupation)[T.Unemployed/Looking for work] -0.5001 0.059 -8.507 0.000 -0.615 -0.385

C(Occupation)[T.Worker] -0.6309 0.050 -12.697 0.000 -0.728 -0.534

C(Exercise)[T.Daily] -0.1470 0.021 -7.009 0.000 -0.188 -0.106

C(Exercise)[T.Hourly] 0.0968 0.117 0.828 0.408 -0.132 0.326

C(Exercise)[T.More Often] -0.1168 0.096 -1.223 0.221 -0.304 0.070

C(Exercise)[T.Never] 0.1467 0.041 3.545 0.000 0.066 0.228

C(Exercise)[T.Once or twice a week] -0.1281 0.022 -5.796 0.000 -0.171 -0.085

C(Exercise)[T.Several times a week] -0.1609 0.021 -7.654 0.000 -0.202 -0.120

C(Meditation)[T.Daily] -0.1355 0.029 -4.644 0.000 -0.193 -0.078

C(Meditation)[T.Hourly] -0.2495 0.252 -0.992 0.321 -0.743 0.244

C(Meditation)[T.More Often] -0.4090 0.252 -1.622 0.105 -0.903 0.085

C(Meditation)[T.Never] -0.1069 0.014 -7.900 0.000 -0.133 -0.080

C(Meditation)[T.Once or twice a week] 0.0012 0.021 0.058 0.954 -0.040 0.042

C(Meditation)[T.Several times a week] 0.0392 0.029 1.333 0.183 -0.018 0.097

C(Reading)[T.Daily] -0.2131 0.016 -13.141 0.000 -0.245 -0.181

C(Reading)[T.Hourly] -0.1519 0.079 -1.929 0.054 -0.306 0.002

C(Reading)[T.More Often] -0.3732 0.130 -2.874 0.004 -0.628 -0.119

C(Reading)[T.Never] 0.0819 0.025 3.338 0.001 0.034 0.130

C(Reading)[T.Once or twice a week] -0.0812 0.017 -4.757 0.000 -0.115 -0.048

C(Reading)[T.Several times a week] -0.1133 0.019 -6.057 0.000 -0.150 -0.077

C(OnlineGambling)[T.Daily] -0.0026 0.064 -0.040 0.968 -0.129 0.123

C(OnlineGambling)[T.Hourly] 0.3444 0.239 1.439 0.150 -0.125 0.813

C(OnlineGambling)[T.More Often] -0.0809 0.388 -0.209 0.835 -0.841 0.679

C(OnlineGambling)[T.Never] -0.0440 0.017 -2.516 0.012 -0.078 -0.010

C(OnlineGambling)[T.Once or twice a week] 0.0194 0.030 0.656 0.512 -0.039 0.077

C(OnlineGambling)[T.Several times a week] 0.1324 0.054 2.462 0.014 0.027 0.238

C(HoursOnline)[T.2-4h] -0.2793 0.024 -11.544 0.000 -0.327 -0.232

C(HoursOnline)[T.4-6h] -0.1653 0.024 -6.802 0.000 -0.213 -0.118

C(HoursOnline)[T.6-8h] -0.1268 0.027 -4.721 0.000 -0.179 -0.074

C(HoursOnline)[T.8-10h] -0.0727 0.027 -2.661 0.008 -0.126 -0.019

C(HoursOnline)[T.less than 2] -0.3323 0.032 -10.464 0.000 -0.395 -0.270

C(HoursOnline)[T.more than 12h] 0.0685 0.041 1.672 0.095 -0.012 0.149

cigarettes_z 0.0157 0.006 2.682 0.007 0.004 0.027

alcohol_z 0.0164 0.006 2.747 0.006 0.005 0.028

openness 0.0766 0.007 11.664 0.000 0.064 0.089

extraversion -0.0666 0.006 -10.826 0.000 -0.079 -0.055

neuroticism 0.3870 0.007 53.252 0.000 0.373 0.401

conscienciousness -0.2806 0.008 -36.393 0.000 -0.296 -0.265

agreeableness -0.0427 0.007 -5.806 0.000 -0.057 -0.028

perfectionism 0.2682 0.012 23.238 0.000 0.246 0.291

reward_drive 0.1270 0.008 15.703 0.000 0.111 0.143

==============================================================================

Omnibus: 3797.938 Durbin-Watson: 1.983

Prob(Omnibus): 0.000 Jarque-Bera (JB): 5594.418

Skew: 0.780 Prob(JB): 0.00

Kurtosis: 4.077 Cond. No. 162.

==============================================================================

Notes:

[1] Standard Errors assume that the covariance matrix of the errors is correctly specified.

OLS Regression Results

==============================================================================

Dep. Variable: insomnia_score R-squared: 0.147

Model: OLS Adj. R-squared: 0.145

Method: Least Squares F-statistic: 102.0

Date: Tue, 11 Apr 2023 Prob (F-statistic): 0.00

Time: 20:02:33 Log-Likelihood: -59787.

No. Observations: 37388 AIC: 1.197e+05

Df Residuals: 37324 BIC: 1.202e+05

Df Model: 63

Covariance Type: nonrobust

==============================================================================================================================

coef std err t P>|t| [0.025 0.975]

------------------------------------------------------------------------------------------------------------------------------

Intercept 2.9368 0.105 27.855 0.000 2.730 3.143

C(timepoint)[T.june2021] -0.0895 0.013 -7.109 0.000 -0.114 -0.065

C(Age_decade)[T.20] -0.0430 0.047 -0.920 0.357 -0.135 0.049

C(Age_decade)[T.30] 0.0146 0.051 0.288 0.773 -0.084 0.114

C(Age_decade)[T.40] 0.0876 0.050 1.752 0.080 -0.010 0.186

C(Age_decade)[T.50] 0.1506 0.050 3.015 0.003 0.053 0.249

C(Age_decade)[T.60] 0.1004 0.053 1.911 0.056 -0.003 0.203

C(Age_decade)[T.70] -0.0117 0.058 -0.203 0.839 -0.125 0.102

C(Age_decade)[T.80] -0.0428 0.090 -0.477 0.633 -0.219 0.133

C(Residence)[T.United Kingdom] 0.0284 0.022 1.272 0.203 -0.015 0.072

C(Sex)[T.Male] -0.2174 0.014 -15.457 0.000 -0.245 -0.190

C(Sex)[T.Other] 0.0443 0.087 0.507 0.612 -0.127 0.216

C(Education)[T.PhD] 0.0395 0.029 1.338 0.181 -0.018 0.097

C(Education)[T.School] 0.0680 0.014 4.786 0.000 0.040 0.096

C(Education)[T.preGCSE] 0.0863 0.051 1.681 0.093 -0.014 0.187

C(Ethnicity)[T.Asian or Asian British] -0.1143 0.073 -1.563 0.118 -0.258 0.029

C(Ethnicity)[T.Black, Black British, Caribbean or African] -0.0404 0.118 -0.343 0.732 -0.271 0.191

C(Ethnicity)[T.Mixed or multiple ethnic groups] -0.0555 0.076 -0.729 0.466 -0.205 0.094

C(Ethnicity)[T.Other ethnic group] -0.1606 0.120 -1.338 0.181 -0.396 0.075

C(Ethnicity)[T.White] -0.0322 0.065 -0.495 0.620 -0.160 0.095

C(Occupation)[T.Homemaker] -0.5055 0.065 -7.791 0.000 -0.633 -0.378

C(Occupation)[T.Retired] -0.5998 0.058 -10.394 0.000 -0.713 -0.487

C(Occupation)[T.Student] -0.5346 0.063 -8.479 0.000 -0.658 -0.411

C(Occupation)[T.Unemployed/Looking for work] -0.5255 0.065 -8.131 0.000 -0.652 -0.399

C(Occupation)[T.Worker] -0.6214 0.055 -11.376 0.000 -0.729 -0.514

C(Exercise)[T.Daily] -0.1935 0.023 -8.390 0.000 -0.239 -0.148

C(Exercise)[T.Hourly] -0.2891 0.129 -2.248 0.025 -0.541 -0.037

C(Exercise)[T.More Often] -0.1825 0.105 -1.738 0.082 -0.388 0.023

C(Exercise)[T.Never] 0.0931 0.046 2.046 0.041 0.004 0.182

C(Exercise)[T.Once or twice a week] -0.1303 0.024 -5.363 0.000 -0.178 -0.083

C(Exercise)[T.Several times a week] -0.1706 0.023 -7.381 0.000 -0.216 -0.125

C(Meditation)[T.Daily] -0.0799 0.032 -2.490 0.013 -0.143 -0.017

C(Meditation)[T.Hourly] -0.4777 0.277 -1.727 0.084 -1.020 0.064

C(Meditation)[T.More Often] -0.6820 0.277 -2.461 0.014 -1.225 -0.139

C(Meditation)[T.Never] -0.0570 0.015 -3.831 0.000 -0.086 -0.028

C(Meditation)[T.Once or twice a week] 0.0156 0.023 0.677 0.498 -0.030 0.061

C(Meditation)[T.Several times a week] 0.0073 0.032 0.225 0.822 -0.056 0.071

C(Reading)[T.Daily] -0.0163 0.018 -0.912 0.362 -0.051 0.019

C(Reading)[T.Hourly] 0.0355 0.087 0.410 0.682 -0.134 0.205

C(Reading)[T.More Often] 0.2625 0.143 1.839 0.066 -0.017 0.542

C(Reading)[T.Never] 0.0633 0.027 2.348 0.019 0.010 0.116

C(Reading)[T.Once or twice a week] -0.0335 0.019 -1.784 0.074 -0.070 0.003

C(Reading)[T.Several times a week] -0.0151 0.021 -0.734 0.463 -0.055 0.025

C(OnlineGambling)[T.Daily] 0.1156 0.071 1.636 0.102 -0.023 0.254

C(OnlineGambling)[T.Hourly] 0.4643 0.263 1.765 0.078 -0.051 0.980

C(OnlineGambling)[T.More Often] 0.3487 0.426 0.818 0.413 -0.487 1.184

C(OnlineGambling)[T.Never] -0.0781 0.019 -4.062 0.000 -0.116 -0.040

C(OnlineGambling)[T.Once or twice a week] 0.0026 0.032 0.081 0.935 -0.061 0.066

C(OnlineGambling)[T.Several times a week] 0.1251 0.059 2.116 0.034 0.009 0.241

C(HoursOnline)[T.2-4h] -0.2428 0.027 -9.131 0.000 -0.295 -0.191

C(HoursOnline)[T.4-6h] -0.2066 0.027 -7.734 0.000 -0.259 -0.154

C(HoursOnline)[T.6-8h] -0.1229 0.030 -4.163 0.000 -0.181 -0.065

C(HoursOnline)[T.8-10h] -0.1397 0.030 -4.652 0.000 -0.198 -0.081

C(HoursOnline)[T.less than 2] -0.3082 0.035 -8.828 0.000 -0.377 -0.240

C(HoursOnline)[T.more than 12h] 0.0909 0.045 2.017 0.044 0.003 0.179

cigarettes_z 0.0282 0.006 4.388 0.000 0.016 0.041

alcohol_z 0.0287 0.007 4.368 0.000 0.016 0.042

openness 0.0627 0.007 8.680 0.000 0.049 0.077

extraversion -0.0433 0.007 -6.399 0.000 -0.057 -0.030

neuroticism 0.3724 0.008 46.610 0.000 0.357 0.388

conscienciousness -0.1078 0.008 -12.724 0.000 -0.124 -0.091

agreeableness -0.0415 0.008 -5.135 0.000 -0.057 -0.026

perfectionism 0.1990 0.013 15.685 0.000 0.174 0.224

reward_drive 0.0442 0.009 4.968 0.000 0.027 0.062

==============================================================================

Omnibus: 1517.952 Durbin-Watson: 2.001

Prob(Omnibus): 0.000 Jarque-Bera (JB): 1708.527

Skew: 0.513 Prob(JB): 0.00

Kurtosis: 3.208 Cond. No. 162.

==============================================================================

Notes:

[1] Standard Errors assume that the covariance matrix of the errors is correctly specified.

OLS Regression Results

==============================================================================

Dep. Variable: MH_composite R-squared: 0.491

Model: OLS Adj. R-squared: 0.491

Method: Least Squares F-statistic: 572.0

Date: Tue, 11 Apr 2023 Prob (F-statistic): 0.00

Time: 20:02:36 Log-Likelihood: -39505.

No. Observations: 37370 AIC: 7.914e+04

Df Residuals: 37306 BIC: 7.968e+04

Df Model: 63

Covariance Type: nonrobust

==============================================================================================================================

coef std err t P>|t| [0.025 0.975]

------------------------------------------------------------------------------------------------------------------------------

Intercept 1.0708 0.061 17.432 0.000 0.950 1.191

C(timepoint)[T.june2021] -0.0422 0.007 -5.762 0.000 -0.057 -0.028

C(Age_decade)[T.20] -0.0669 0.027 -2.460 0.014 -0.120 -0.014

C(Age_decade)[T.30] -0.0928 0.029 -3.152 0.002 -0.150 -0.035

C(Age_decade)[T.40] -0.1650 0.029 -5.666 0.000 -0.222 -0.108

C(Age_decade)[T.50] -0.2724 0.029 -9.361 0.000 -0.329 -0.215

C(Age_decade)[T.60] -0.3454 0.031 -11.285 0.000 -0.405 -0.285

C(Age_decade)[T.70] -0.3606 0.034 -10.710 0.000 -0.427 -0.295

C(Age_decade)[T.80] -0.3660 0.052 -7.007 0.000 -0.468 -0.264

C(Residence)[T.United Kingdom] 0.0166 0.013 1.279 0.201 -0.009 0.042

C(Sex)[T.Male] -0.0910 0.008 -11.127 0.000 -0.107 -0.075

C(Sex)[T.Other] 0.1180 0.051 2.322 0.020 0.018 0.218

C(Education)[T.PhD] 0.0078 0.017 0.453 0.650 -0.026 0.041

C(Education)[T.School] 0.0099 0.008 1.203 0.229 -0.006 0.026

C(Education)[T.preGCSE] 0.0279 0.030 0.933 0.351 -0.031 0.086

C(Ethnicity)[T.Asian or Asian British] -0.0448 0.043 -1.051 0.293 -0.128 0.039

C(Ethnicity)[T.Black, Black British, Caribbean or African] -0.0718 0.069 -1.047 0.295 -0.206 0.063

C(Ethnicity)[T.Mixed or multiple ethnic groups] -0.0270 0.044 -0.609 0.543 -0.114 0.060

C(Ethnicity)[T.Other ethnic group] 0.0467 0.070 0.669 0.504 -0.090 0.184

C(Ethnicity)[T.White] -0.0067 0.038 -0.177 0.859 -0.081 0.068

C(Occupation)[T.Homemaker] -0.4140 0.038 -10.965 0.000 -0.488 -0.340

C(Occupation)[T.Retired] -0.5158 0.034 -15.356 0.000 -0.582 -0.450

C(Occupation)[T.Student] -0.4088 0.037 -11.137 0.000 -0.481 -0.337

C(Occupation)[T.Unemployed/Looking for work] -0.4024 0.038 -10.698 0.000 -0.476 -0.329

C(Occupation)[T.Worker] -0.4594 0.032 -14.447 0.000 -0.522 -0.397

C(Exercise)[T.Daily] -0.1326 0.013 -9.884 0.000 -0.159 -0.106

C(Exercise)[T.Hourly] 0.0426 0.075 0.570 0.569 -0.104 0.189

C(Exercise)[T.More Often] -0.0698 0.061 -1.143 0.253 -0.189 0.050

C(Exercise)[T.Never] 0.0377 0.026 1.423 0.155 -0.014 0.090

C(Exercise)[T.Once or twice a week] -0.1106 0.014 -7.825 0.000 -0.138 -0.083

C(Exercise)[T.Several times a week] -0.1345 0.013 -10.005 0.000 -0.161 -0.108

C(Meditation)[T.Daily] -0.0419 0.019 -2.244 0.025 -0.078 -0.005

C(Meditation)[T.Hourly] 0.1919 0.161 1.193 0.233 -0.123 0.507

C(Meditation)[T.More Often] -0.3026 0.161 -1.877 0.061 -0.619 0.013

C(Meditation)[T.Never] -0.0916 0.009 -10.584 0.000 -0.109 -0.075

C(Meditation)[T.Once or twice a week] -0.0022 0.013 -0.167 0.867 -0.029 0.024

C(Meditation)[T.Several times a week] 0.0199 0.019 1.057 0.291 -0.017 0.057

C(Reading)[T.Daily] -0.0696 0.010 -6.707 0.000 -0.090 -0.049

C(Reading)[T.Hourly] -0.0274 0.050 -0.544 0.586 -0.126 0.071

C(Reading)[T.More Often] -0.0527 0.083 -0.635 0.526 -0.215 0.110

C(Reading)[T.Never] 0.0586 0.016 3.737 0.000 0.028 0.089

C(Reading)[T.Once or twice a week] -0.0328 0.011 -3.007 0.003 -0.054 -0.011

C(Reading)[T.Several times a week] -0.0626 0.012 -5.228 0.000 -0.086 -0.039

C(OnlineGambling)[T.Daily] 0.0738 0.041 1.792 0.073 -0.007 0.154

C(OnlineGambling)[T.Hourly] 0.0970 0.153 0.634 0.526 -0.203 0.397

C(OnlineGambling)[T.More Often] 0.0207 0.248 0.083 0.934 -0.465 0.507

C(OnlineGambling)[T.Never] -0.0395 0.011 -3.530 0.000 -0.061 -0.018

C(OnlineGambling)[T.Once or twice a week] 0.0160 0.019 0.847 0.397 -0.021 0.053

C(OnlineGambling)[T.Several times a week] 0.0993 0.034 2.886 0.004 0.032 0.167

C(HoursOnline)[T.2-4h] -0.1789 0.015 -11.564 0.000 -0.209 -0.149

C(HoursOnline)[T.4-6h] -0.1158 0.016 -7.452 0.000 -0.146 -0.085

C(HoursOnline)[T.6-8h] -0.0558 0.017 -3.249 0.001 -0.089 -0.022

C(HoursOnline)[T.8-10h] -0.0468 0.017 -2.679 0.007 -0.081 -0.013

C(HoursOnline)[T.less than 2] -0.2258 0.020 -11.121 0.000 -0.266 -0.186

C(HoursOnline)[T.more than 12h] 0.0692 0.026 2.638 0.008 0.018 0.121

cigarettes_z 0.0166 0.004 4.442 0.000 0.009 0.024

alcohol_z 0.0167 0.004 4.366 0.000 0.009 0.024

openness 0.0702 0.004 16.722 0.000 0.062 0.078

extraversion -0.0539 0.004 -13.698 0.000 -0.062 -0.046

neuroticism 0.5554 0.005 119.506 0.000 0.546 0.564

conscienciousness -0.1504 0.005 -30.507 0.000 -0.160 -0.141

agreeableness -0.0428 0.005 -9.106 0.000 -0.052 -0.034

perfectionism 0.2078 0.007 28.150 0.000 0.193 0.222

reward_drive 0.0953 0.005 18.424 0.000 0.085 0.105

==============================================================================

Omnibus: 4492.907 Durbin-Watson: 2.010

Prob(Omnibus): 0.000 Jarque-Bera (JB): 8659.374

Skew: 0.779 Prob(JB): 0.00

Kurtosis: 4.771 Cond. No. 162.

==============================================================================

Notes:

[1] Standard Errors assume that the covariance matrix of the errors is correctly specified.

2.From resilience variables

OLS Regression Results

==================================================================================

Dep. Variable: more_relaxed_lifestyle R-squared: 0.211

Model: OLS Adj. R-squared: 0.211

Method: Least Squares F-statistic: 451.2

Date: Tue, 11 Apr 2023 Prob (F-statistic): 0.00

Time: 20:04:26 Log-Likelihood: -86726.

No. Observations: 108031 AIC: 1.736e+05

Df Residuals: 107966 BIC: 1.742e+05

Df Model: 64

Covariance Type: nonrobust

==============================================================================================================================

coef std err t P>|t| [0.025 0.975]

------------------------------------------------------------------------------------------------------------------------------

Intercept 0.4030 0.034 11.733 0.000 0.336 0.470

C(timepoint)[T.june2021] -0.1493 0.006 -26.386 0.000 -0.160 -0.138

C(timepoint)[T.may2020] -0.0567 0.004 -13.056 0.000 -0.065 -0.048

C(Age_decade)[T.20.0] -0.0599 0.014 -4.420 0.000 -0.086 -0.033

C(Age_decade)[T.30.0] -0.1413 0.015 -9.567 0.000 -0.170 -0.112

C(Age_decade)[T.40.0] -0.1981 0.015 -13.590 0.000 -0.227 -0.170

C(Age_decade)[T.50.0] -0.2345 0.014 -16.237 0.000 -0.263 -0.206

C(Age_decade)[T.60.0] -0.2545 0.015 -17.054 0.000 -0.284 -0.225

C(Age_decade)[T.70.0] -0.2686 0.016 -16.841 0.000 -0.300 -0.237

C(Age_decade)[T.80.0] -0.1608 0.021 -7.494 0.000 -0.203 -0.119

C(Residence)[T.United Kingdom] 0.0152 0.006 2.421 0.015 0.003 0.028

C(Sex)[T.Male] -0.1145 0.004 -29.843 0.000 -0.122 -0.107

C(Sex)[T.Other] 0.0187 0.026 0.719 0.472 -0.032 0.070

C(Education)[T.PhD] -0.0238 0.008 -2.891 0.004 -0.040 -0.008

C(Education)[T.School] -0.0046 0.004 -1.234 0.217 -0.012 0.003

C(Education)[T.preGCSE] -0.0313 0.014 -2.193 0.028 -0.059 -0.003

C(Ethnicity)[T.Asian or Asian British] 0.1300 0.027 4.757 0.000 0.076 0.184

C(Ethnicity)[T.Black, Black British, Caribbean or African] 0.2666 0.040 6.671 0.000 0.188 0.345

C(Ethnicity)[T.Mixed or multiple ethnic groups] 0.0351 0.028 1.248 0.212 -0.020 0.090

C(Ethnicity)[T.Other ethnic group] -0.0585 0.037 -1.561 0.119 -0.132 0.015

C(Ethnicity)[T.White] 0.0917 0.025 3.632 0.000 0.042 0.141

C(Occupation)[T.Homemaker] -0.0948 0.018 -5.336 0.000 -0.130 -0.060

C(Occupation)[T.Retired] 0.0137 0.016 0.886 0.376 -0.017 0.044

C(Occupation)[T.Student] 0.0976 0.017 5.632 0.000 0.064 0.132

C(Occupation)[T.Unemployed/Looking for work] 0.1677 0.018 9.493 0.000 0.133 0.202

C(Occupation)[T.Worker] -0.0183 0.015 -1.234 0.217 -0.047 0.011

C(Exercise)[T.Daily] -0.1049 0.007 -15.836 0.000 -0.118 -0.092

C(Exercise)[T.Hourly] -0.1947 0.028 -6.921 0.000 -0.250 -0.140

C(Exercise)[T.More Often] -0.1944 0.025 -7.910 0.000 -0.243 -0.146

C(Exercise)[T.Never] -0.0958 0.015 -6.602 0.000 -0.124 -0.067

C(Exercise)[T.Once or twice a week] 0.0189 0.007 2.633 0.008 0.005 0.033

C(Exercise)[T.Several times a week] -0.0322 0.007 -4.775 0.000 -0.045 -0.019

C(Meditation)[T.Daily] -0.1033 0.008 -12.816 0.000 -0.119 -0.087

C(Meditation)[T.Hourly] -0.3220 0.090 -3.567 0.000 -0.499 -0.145

C(Meditation)[T.More Often] -0.2080 0.060 -3.451 0.001 -0.326 -0.090

C(Meditation)[T.Never] -0.0723 0.004 -18.325 0.000 -0.080 -0.065

C(Meditation)[T.Once or twice a week] -0.0211 0.006 -3.557 0.000 -0.033 -0.009

C(Meditation)[T.Several times a week] -0.0761 0.008 -9.188 0.000 -0.092 -0.060

C(Reading)[T.Daily] -0.0413 0.005 -8.599 0.000 -0.051 -0.032

C(Reading)[T.Hourly] 0.0305 0.020 1.542 0.123 -0.008 0.069

C(Reading)[T.More Often] -0.1229 0.032 -3.891 0.000 -0.185 -0.061

C(Reading)[T.Never] 0.0513 0.008 6.187 0.000 0.035 0.068

C(Reading)[T.Once or twice a week] -0.0067 0.005 -1.275 0.202 -0.017 0.004

C(Reading)[T.Several times a week] -0.0131 0.006 -2.371 0.018 -0.024 -0.002

C(OnlineGambling)[T.Daily] -0.0730 0.022 -3.303 0.001 -0.116 -0.030

C(OnlineGambling)[T.Hourly] 0.0463 0.089 0.519 0.604 -0.128 0.221

C(OnlineGambling)[T.More Often] -0.1504 0.087 -1.733 0.083 -0.320 0.020

C(OnlineGambling)[T.Never] -0.0792 0.005 -14.733 0.000 -0.090 -0.069

C(OnlineGambling)[T.Once or twice a week] -0.0357 0.009 -3.777 0.000 -0.054 -0.017

C(OnlineGambling)[T.Several times a week] 0.0002 0.019 0.009 0.993 -0.036 0.037

C(HoursOnline)[T.2-4h] -0.0436 0.008 -5.402 0.000 -0.059 -0.028

C(HoursOnline)[T.4-6h] 0.0011 0.008 0.132 0.895 -0.015 0.017

C(HoursOnline)[T.6-8h] 0.0086 0.009 0.968 0.333 -0.009 0.026

C(HoursOnline)[T.8-10h] 0.0084 0.009 0.914 0.361 -0.010 0.026

C(HoursOnline)[T.less than 2] -0.1157 0.010 -11.925 0.000 -0.135 -0.097

C(HoursOnline)[T.more than 12h] 0.0091 0.014 0.638 0.523 -0.019 0.037

cigarettes_z 0.0135 0.002 7.816 0.000 0.010 0.017

alcohol_z 0.0209 0.002 11.862 0.000 0.017 0.024

extraversion 0.0032 0.002 1.839 0.066 -0.000 0.007

openness 0.0352 0.002 18.867 0.000 0.032 0.039

neuroticism 0.1597 0.002 75.902 0.000 0.156 0.164

conscienciousness -0.1016 0.002 -45.285 0.000 -0.106 -0.097

agreeableness 0.0295 0.002 13.857 0.000 0.025 0.034

perfectionism 0.0863 0.003 25.637 0.000 0.080 0.093

reward_drive 0.0600 0.002 25.838 0.000 0.055 0.065

==============================================================================

Omnibus: 953.771 Durbin-Watson: 0.931

Prob(Omnibus): 0.000 Jarque-Bera (JB): 728.642

Skew: -0.113 Prob(JB): 5.99e-159

Kurtosis: 2.668 Cond. No. 135.

==============================================================================

Notes:

[1] Standard Errors assume that the covariance matrix of the errors is correctly specified.

OLS Regression Results

===============================================================================

Dep. Variable: disrupted_lifestyle R-squared: 0.110

Model: OLS Adj. R-squared: 0.109

Method: Least Squares F-statistic: 207.5

Date: Tue, 11 Apr 2023 Prob (F-statistic): 0.00

Time: 20:04:35 Log-Likelihood: -91414.

No. Observations: 108031 AIC: 1.830e+05

Df Residuals: 107966 BIC: 1.836e+05

Df Model: 64

Covariance Type: nonrobust

==============================================================================================================================

coef std err t P>|t| [0.025 0.975]

------------------------------------------------------------------------------------------------------------------------------

Intercept 0.3801 0.036 10.599 0.000 0.310 0.450

C(timepoint)[T.june2021] -0.0833 0.006 -14.092 0.000 -0.095 -0.072

C(timepoint)[T.may2020] 0.0568 0.005 12.516 0.000 0.048 0.066

C(Age_decade)[T.20.0] -0.0752 0.014 -5.315 0.000 -0.103 -0.047

C(Age_decade)[T.30.0] -0.1065 0.015 -6.904 0.000 -0.137 -0.076

C(Age_decade)[T.40.0] -0.1385 0.015 -9.098 0.000 -0.168 -0.109

C(Age_decade)[T.50.0] -0.1536 0.015 -10.187 0.000 -0.183 -0.124

C(Age_decade)[T.60.0] -0.1422 0.016 -9.121 0.000 -0.173 -0.112

C(Age_decade)[T.70.0] -0.1252 0.017 -7.513 0.000 -0.158 -0.093

C(Age_decade)[T.80.0] 0.0277 0.022 1.237 0.216 -0.016 0.072

C(Residence)[T.United Kingdom] 0.0189 0.007 2.886 0.004 0.006 0.032

C(Sex)[T.Male] -0.0513 0.004 -12.815 0.000 -0.059 -0.043

C(Sex)[T.Other] 0.1161 0.027 4.275 0.000 0.063 0.169

C(Education)[T.PhD] -0.0493 0.009 -5.736 0.000 -0.066 -0.032

C(Education)[T.School] -0.0220 0.004 -5.637 0.000 -0.030 -0.014

C(Education)[T.preGCSE] -0.0024 0.015 -0.160 0.873 -0.032 0.027

C(Ethnicity)[T.Asian or Asian British] -0.0086 0.029 -0.301 0.763 -0.065 0.047

C(Ethnicity)[T.Black, Black British, Caribbean or African] -0.0859 0.042 -2.058 0.040 -0.168 -0.004

C(Ethnicity)[T.Mixed or multiple ethnic groups] -0.0778 0.029 -2.644 0.008 -0.135 -0.020

C(Ethnicity)[T.Other ethnic group] -0.0247 0.039 -0.632 0.527 -0.101 0.052

C(Ethnicity)[T.White] -0.0298 0.026 -1.129 0.259 -0.081 0.022

C(Occupation)[T.Homemaker] -0.0311 0.019 -1.678 0.093 -0.067 0.005

C(Occupation)[T.Retired] -0.0657 0.016 -4.052 0.000 -0.097 -0.034

C(Occupation)[T.Student] 0.0042 0.018 0.231 0.818 -0.031 0.040

C(Occupation)[T.Unemployed/Looking for work] 0.0236 0.018 1.281 0.200 -0.013 0.060

C(Occupation)[T.Worker] -0.0148 0.015 -0.954 0.340 -0.045 0.016

C(Exercise)[T.Daily] -0.0028 0.007 -0.403 0.687 -0.016 0.011

C(Exercise)[T.Hourly] -0.0057 0.029 -0.193 0.847 -0.063 0.052

C(Exercise)[T.More Often] -0.1333 0.026 -5.191 0.000 -0.184 -0.083

C(Exercise)[T.Never] -0.0752 0.015 -4.963 0.000 -0.105 -0.046

C(Exercise)[T.Once or twice a week] -0.0210 0.008 -2.801 0.005 -0.036 -0.006

C(Exercise)[T.Several times a week] 0.0041 0.007 0.580 0.562 -0.010 0.018

C(Meditation)[T.Daily] 0.0110 0.008 1.305 0.192 -0.006 0.027

C(Meditation)[T.Hourly] -0.1158 0.094 -1.228 0.219 -0.301 0.069

C(Meditation)[T.More Often] -0.1236 0.063 -1.964 0.050 -0.247 -0.000

C(Meditation)[T.Never] -0.0905 0.004 -21.947 0.000 -0.099 -0.082

C(Meditation)[T.Once or twice a week] 0.0126 0.006 2.036 0.042 0.000 0.025

C(Meditation)[T.Several times a week] -0.0092 0.009 -1.065 0.287 -0.026 0.008

C(Reading)[T.Daily] -0.0555 0.005 -11.057 0.000 -0.065 -0.046

C(Reading)[T.Hourly] 0.0375 0.021 1.821 0.069 -0.003 0.078

C(Reading)[T.More Often] 0.0666 0.033 2.018 0.044 0.002 0.131

C(Reading)[T.Never] -0.0206 0.009 -2.379 0.017 -0.038 -0.004

C(Reading)[T.Once or twice a week] -0.0281 0.005 -5.121 0.000 -0.039 -0.017

C(Reading)[T.Several times a week] -0.0493 0.006 -8.536 0.000 -0.061 -0.038

C(OnlineGambling)[T.Daily] -0.0176 0.023 -0.761 0.447 -0.063 0.028

C(OnlineGambling)[T.Hourly] -0.3397 0.093 -3.649 0.000 -0.522 -0.157

C(OnlineGambling)[T.More Often] -0.3920 0.091 -4.328 0.000 -0.570 -0.214

C(OnlineGambling)[T.Never] -0.0830 0.006 -14.789 0.000 -0.094 -0.072

C(OnlineGambling)[T.Once or twice a week] -0.0200 0.010 -2.024 0.043 -0.039 -0.001

C(OnlineGambling)[T.Several times a week] -0.0069 0.019 -0.356 0.722 -0.045 0.031

C(HoursOnline)[T.2-4h] -0.0446 0.008 -5.288 0.000 -0.061 -0.028

C(HoursOnline)[T.4-6h] -0.0106 0.008 -1.246 0.213 -0.027 0.006

C(HoursOnline)[T.6-8h] -0.0325 0.009 -3.519 0.000 -0.051 -0.014

C(HoursOnline)[T.8-10h] -0.0187 0.010 -1.948 0.051 -0.038 0.000

C(HoursOnline)[T.less than 2] -0.1056 0.010 -10.420 0.000 -0.125 -0.086

C(HoursOnline)[T.more than 12h] -0.0057 0.015 -0.386 0.699 -0.035 0.023

cigarettes_z 5.813e-05 0.002 0.032 0.974 -0.003 0.004

alcohol_z 0.0027 0.002 1.486 0.137 -0.001 0.006

extraversion 0.0124 0.002 6.735 0.000 0.009 0.016

openness 0.0517 0.002 26.486 0.000 0.048 0.055

neuroticism 0.0710 0.002 32.316 0.000 0.067 0.075

conscienciousness -0.0728 0.002 -31.053 0.000 -0.077 -0.068

agreeableness 0.0121 0.002 5.460 0.000 0.008 0.017

perfectionism 0.0923 0.004 26.251 0.000 0.085 0.099

reward_drive 0.0869 0.002 35.833 0.000 0.082 0.092

==============================================================================

Omnibus: 300.872 Durbin-Watson: 0.980

Prob(Omnibus): 0.000 Jarque-Bera (JB): 240.777

Skew: 0.032 Prob(JB): 5.20e-53

Kurtosis: 2.778 Cond. No. 135.

==============================================================================

Notes:

[1] Standard Errors assume that the covariance matrix of the errors is correctly specified.

OLS Regression Results

====================================================================================

Dep. Variable: elevated_health_concerns R-squared: 0.093

Model: OLS Adj. R-squared: 0.092

Method: Least Squares F-statistic: 172.2

Date: Tue, 11 Apr 2023 Prob (F-statistic): 0.00

Time: 20:04:43 Log-Likelihood: -1.2840e+05

No. Observations: 108031 AIC: 2.569e+05

Df Residuals: 107966 BIC: 2.576e+05

Df Model: 64

Covariance Type: nonrobust

==============================================================================================================================

coef std err t P>|t| [0.025 0.975]

------------------------------------------------------------------------------------------------------------------------------

Intercept -0.4277 0.051 -8.468 0.000 -0.527 -0.329

C(timepoint)[T.june2021] 0.0704 0.008 8.461 0.000 0.054 0.087

C(timepoint)[T.may2020] 0.2475 0.006 38.722 0.000 0.235 0.260

C(Age_decade)[T.20.0] -0.0791 0.020 -3.969 0.000 -0.118 -0.040

C(Age_decade)[T.30.0] 0.0498 0.022 2.294 0.022 0.007 0.092

C(Age_decade)[T.40.0] 0.0695 0.021 3.243 0.001 0.028 0.112

C(Age_decade)[T.50.0] 0.1205 0.021 5.674 0.000 0.079 0.162

C(Age_decade)[T.60.0] 0.0577 0.022 2.627 0.009 0.015 0.101

C(Age_decade)[T.70.0] 0.0194 0.023 0.828 0.408 -0.027 0.065

C(Age_decade)[T.80.0] 0.0168 0.032 0.534 0.594 -0.045 0.079

C(Residence)[T.United Kingdom] -0.0183 0.009 -1.981 0.048 -0.036 -0.000

C(Sex)[T.Male] -0.0809 0.006 -14.343 0.000 -0.092 -0.070

C(Sex)[T.Other] -0.0683 0.038 -1.785 0.074 -0.143 0.007

C(Education)[T.PhD] -0.0917 0.012 -7.576 0.000 -0.115 -0.068

C(Education)[T.School] -0.0063 0.005 -1.147 0.251 -0.017 0.004

C(Education)[T.preGCSE] -0.0063 0.021 -0.301 0.763 -0.047 0.035

C(Ethnicity)[T.Asian or Asian British] -0.0240 0.040 -0.598 0.550 -0.103 0.055

C(Ethnicity)[T.Black, Black British, Caribbean or African] -0.0379 0.059 -0.645 0.519 -0.153 0.077

C(Ethnicity)[T.Mixed or multiple ethnic groups] 0.0726 0.041 1.753 0.080 -0.009 0.154

C(Ethnicity)[T.Other ethnic group] 0.0824 0.055 1.496 0.135 -0.026 0.190

C(Ethnicity)[T.White] -0.0537 0.037 -1.447 0.148 -0.127 0.019

C(Occupation)[T.Homemaker] 0.3523 0.026 13.486 0.000 0.301 0.404

C(Occupation)[T.Retired] 0.2362 0.023 10.352 0.000 0.191 0.281

C(Occupation)[T.Student] 0.1292 0.025 5.067 0.000 0.079 0.179

C(Occupation)[T.Unemployed/Looking for work] 0.2193 0.026 8.439 0.000 0.168 0.270

C(Occupation)[T.Worker] 0.3431 0.022 15.723 0.000 0.300 0.386

C(Exercise)[T.Daily] 0.2459 0.010 25.243 0.000 0.227 0.265

C(Exercise)[T.Hourly] 0.0613 0.041 1.480 0.139 -0.020 0.142

C(Exercise)[T.More Often] 0.2283 0.036 6.314 0.000 0.157 0.299

C(Exercise)[T.Never] 0.1074 0.021 5.033 0.000 0.066 0.149

C(Exercise)[T.Once or twice a week] 0.0918 0.011 8.677 0.000 0.071 0.113

C(Exercise)[T.Several times a week] 0.1644 0.010 16.595 0.000 0.145 0.184

C(Meditation)[T.Daily] 0.1706 0.012 14.391 0.000 0.147 0.194

C(Meditation)[T.Hourly] 0.1777 0.133 1.339 0.181 -0.082 0.438

C(Meditation)[T.More Often] 0.3825 0.089 4.316 0.000 0.209 0.556

C(Meditation)[T.Never] -0.0352 0.006 -6.058 0.000 -0.047 -0.024

C(Meditation)[T.Once or twice a week] 0.0348 0.009 3.985 0.000 0.018 0.052

C(Meditation)[T.Several times a week] 0.1473 0.012 12.095 0.000 0.123 0.171

C(Reading)[T.Daily] -0.0349 0.007 -4.942 0.000 -0.049 -0.021

C(Reading)[T.Hourly] 0.0603 0.029 2.076 0.038 0.003 0.117

C(Reading)[T.More Often] 0.0949 0.046 2.043 0.041 0.004 0.186

C(Reading)[T.Never] -0.0709 0.012 -5.811 0.000 -0.095 -0.047

C(Reading)[T.Once or twice a week] 0.0051 0.008 0.658 0.510 -0.010 0.020

C(Reading)[T.Several times a week] -0.0725 0.008 -8.922 0.000 -0.088 -0.057

C(OnlineGambling)[T.Daily] 0.1237 0.033 3.805 0.000 0.060 0.187

C(OnlineGambling)[T.Hourly] 0.0782 0.131 0.597 0.551 -0.179 0.335

C(OnlineGambling)[T.More Often] -0.5281 0.128 -4.140 0.000 -0.778 -0.278

C(OnlineGambling)[T.Never] -0.0442 0.008 -5.593 0.000 -0.060 -0.029

C(OnlineGambling)[T.Once or twice a week] -0.0071 0.014 -0.509 0.611 -0.034 0.020

C(OnlineGambling)[T.Several times a week] -0.0434 0.027 -1.581 0.114 -0.097 0.010

C(HoursOnline)[T.2-4h] -0.0320 0.012 -2.690 0.007 -0.055 -0.009

C(HoursOnline)[T.4-6h] -0.0276 0.012 -2.307 0.021 -0.051 -0.004

C(HoursOnline)[T.6-8h] -0.0449 0.013 -3.453 0.001 -0.070 -0.019

C(HoursOnline)[T.8-10h] -0.0048 0.014 -0.354 0.723 -0.031 0.022

C(HoursOnline)[T.less than 2] -0.0335 0.014 -2.348 0.019 -0.061 -0.006

C(HoursOnline)[T.more than 12h] -0.0305 0.021 -1.455 0.146 -0.072 0.011

cigarettes_z -0.0177 0.003 -7.002 0.000 -0.023 -0.013

alcohol_z -0.0194 0.003 -7.501 0.000 -0.024 -0.014

extraversion 0.0180 0.003 6.965 0.000 0.013 0.023

openness 0.0444 0.003 16.161 0.000 0.039 0.050

neuroticism -0.1473 0.003 -47.590 0.000 -0.153 -0.141

conscienciousness 0.0345 0.003 10.454 0.000 0.028 0.041

agreeableness 0.0510 0.003 16.280 0.000 0.045 0.057

perfectionism -0.0099 0.005 -1.994 0.046 -0.020 -0.000

reward_drive 0.0592 0.003 17.344 0.000 0.053 0.066

==============================================================================

Omnibus: 659.187 Durbin-Watson: 0.940

Prob(Omnibus): 0.000 Jarque-Bera (JB): 709.047

Skew: -0.163 Prob(JB): 1.08e-154

Kurtosis: 3.227 Cond. No. 135.

==============================================================================

Notes:

[1] Standard Errors assume that the covariance matrix of the errors is correctly specified.

OLS Regression Results

==============================================================================

Dep. Variable: optimism R-squared: 0.101

Model: OLS Adj. R-squared: 0.100

Method: Least Squares F-statistic: 188.6

Date: Tue, 11 Apr 2023 Prob (F-statistic): 0.00

Time: 20:04:50 Log-Likelihood: -1.4488e+05

No. Observations: 108031 AIC: 2.899e+05

Df Residuals: 107966 BIC: 2.905e+05

Df Model: 64

Covariance Type: nonrobust

==============================================================================================================================

coef std err t P>|t| [0.025 0.975]

------------------------------------------------------------------------------------------------------------------------------

Intercept -0.2921 0.059 -4.965 0.000 -0.407 -0.177

C(timepoint)[T.june2021] -0.0241 0.010 -2.491 0.013 -0.043 -0.005

C(timepoint)[T.may2020] 0.3038 0.007 40.809 0.000 0.289 0.318

C(Age_decade)[T.20.0] -0.1825 0.023 -7.864 0.000 -0.228 -0.137

C(Age_decade)[T.30.0] -0.2784 0.025 -11.000 0.000 -0.328 -0.229

C(Age_decade)[T.40.0] -0.3420 0.025 -13.698 0.000 -0.391 -0.293

C(Age_decade)[T.50.0] -0.2228 0.025 -9.004 0.000 -0.271 -0.174

C(Age_decade)[T.60.0] -0.2331 0.026 -9.117 0.000 -0.283 -0.183

C(Age_decade)[T.70.0] -0.2883 0.027 -10.550 0.000 -0.342 -0.235

C(Age_decade)[T.80.0] -0.2902 0.037 -7.898 0.000 -0.362 -0.218

C(Residence)[T.United Kingdom] -0.0349 0.011 -3.246 0.001 -0.056 -0.014

C(Sex)[T.Male] -0.0938 0.007 -14.269 0.000 -0.107 -0.081

C(Sex)[T.Other] -0.1122 0.045 -2.517 0.012 -0.200 -0.025

C(Education)[T.PhD] -0.1398 0.014 -9.915 0.000 -0.167 -0.112

C(Education)[T.School] 0.0065 0.006 1.020 0.308 -0.006 0.019

C(Education)[T.preGCSE] -0.0102 0.024 -0.418 0.676 -0.058 0.038

C(Ethnicity)[T.Asian or Asian British] -0.0223 0.047 -0.476 0.634 -0.114 0.069

C(Ethnicity)[T.Black, Black British, Caribbean or African] -0.2346 0.068 -3.427 0.001 -0.369 -0.100

C(Ethnicity)[T.Mixed or multiple ethnic groups] -0.0531 0.048 -1.101 0.271 -0.148 0.041

C(Ethnicity)[T.Other ethnic group] 0.1290 0.064 2.010 0.044 0.003 0.255

C(Ethnicity)[T.White] -0.1355 0.043 -3.133 0.002 -0.220 -0.051

C(Occupation)[T.Homemaker] 0.1806 0.030 5.934 0.000 0.121 0.240

C(Occupation)[T.Retired] 0.3261 0.027 12.268 0.000 0.274 0.378

C(Occupation)[T.Student] 0.1125 0.030 3.787 0.000 0.054 0.171

C(Occupation)[T.Unemployed/Looking for work] 0.3368 0.030 11.124 0.000 0.277 0.396

C(Occupation)[T.Worker] 0.2670 0.025 10.504 0.000 0.217 0.317

C(Exercise)[T.Daily] 0.2758 0.011 24.297 0.000 0.254 0.298

C(Exercise)[T.Hourly] 0.1117 0.048 2.318 0.020 0.017 0.206

C(Exercise)[T.More Often] 0.2731 0.042 6.486 0.000 0.191 0.356

C(Exercise)[T.Never] 0.1294 0.025 5.204 0.000 0.081 0.178

C(Exercise)[T.Once or twice a week] 0.1517 0.012 12.313 0.000 0.128 0.176

C(Exercise)[T.Several times a week] 0.2220 0.012 19.243 0.000 0.199 0.245

C(Meditation)[T.Daily] 0.1288 0.014 9.327 0.000 0.102 0.156

C(Meditation)[T.Hourly] 0.1046 0.155 0.676 0.499 -0.198 0.408

C(Meditation)[T.More Often] -0.6608 0.103 -6.400 0.000 -0.863 -0.458

C(Meditation)[T.Never] -0.0208 0.007 -3.083 0.002 -0.034 -0.008

C(Meditation)[T.Once or twice a week] 0.0083 0.010 0.819 0.413 -0.012 0.028

C(Meditation)[T.Several times a week] 0.0707 0.014 4.981 0.000 0.043 0.098

C(Reading)[T.Daily] 0.2046 0.008 24.862 0.000 0.188 0.221

C(Reading)[T.Hourly] 0.2782 0.034 8.222 0.000 0.212 0.345

C(Reading)[T.More Often] 0.3188 0.054 5.889 0.000 0.213 0.425

C(Reading)[T.Never] -0.2822 0.014 -19.849 0.000 -0.310 -0.254

C(Reading)[T.Once or twice a week] 0.1321 0.009 14.683 0.000 0.114 0.150

C(Reading)[T.Several times a week] 0.1358 0.009 14.351 0.000 0.117 0.154

C(OnlineGambling)[T.Daily] 0.1485 0.038 3.920 0.000 0.074 0.223

C(OnlineGambling)[T.Hourly] 0.1057 0.153 0.692 0.489 -0.194 0.405

C(OnlineGambling)[T.More Often] -0.3285 0.149 -2.211 0.027 -0.620 -0.037

C(OnlineGambling)[T.Never] -0.0037 0.009 -0.405 0.685 -0.022 0.014

C(OnlineGambling)[T.Once or twice a week] 0.0266 0.016 1.641 0.101 -0.005 0.058

C(OnlineGambling)[T.Several times a week] -0.0500 0.032 -1.564 0.118 -0.113 0.013

C(HoursOnline)[T.2-4h] 0.0891 0.014 6.441 0.000 0.062 0.116

C(HoursOnline)[T.4-6h] 0.0718 0.014 5.148 0.000 0.044 0.099

C(HoursOnline)[T.6-8h] 0.0219 0.015 1.447 0.148 -0.008 0.052

C(HoursOnline)[T.8-10h] 0.0133 0.016 0.841 0.401 -0.018 0.044

C(HoursOnline)[T.less than 2] 0.0667 0.017 4.016 0.000 0.034 0.099

C(HoursOnline)[T.more than 12h] 0.0248 0.024 1.016 0.309 -0.023 0.073

cigarettes_z -0.0196 0.003 -6.644 0.000 -0.025 -0.014

alcohol_z -0.0071 0.003 -2.362 0.018 -0.013 -0.001

extraversion 0.0004 0.003 0.116 0.908 -0.006 0.006

openness 0.0646 0.003 20.187 0.000 0.058 0.071

neuroticism -0.1132 0.004 -31.402 0.000 -0.120 -0.106

conscienciousness 0.0050 0.004 1.295 0.195 -0.003 0.013

agreeableness 0.0498 0.004 13.641 0.000 0.043 0.057

perfectionism -0.0147 0.006 -2.554 0.011 -0.026 -0.003

reward_drive 0.0720 0.004 18.093 0.000 0.064 0.080

==============================================================================

Omnibus: 787.815 Durbin-Watson: 0.937

Prob(Omnibus): 0.000 Jarque-Bera (JB): 873.185

Skew: -0.171 Prob(JB): 2.46e-190

Kurtosis: 3.278 Cond. No. 135.

==============================================================================

Notes:

[1] Standard Errors assume that the covariance matrix of the errors is correctly specified.

OLS Regression Results

======================================================================================

Dep. Variable: increased_conflict_at_home R-squared: 0.122

Model: OLS Adj. R-squared: 0.122

Method: Least Squares F-statistic: 235.0

Date: Tue, 11 Apr 2023 Prob (F-statistic): 0.00

Time: 20:04:58 Log-Likelihood: -1.6876e+05

No. Observations: 108031 AIC: 3.377e+05

Df Residuals: 107966 BIC: 3.383e+05

Df Model: 64

Covariance Type: nonrobust

==============================================================================================================================

coef std err t P>|t| [0.025 0.975]

------------------------------------------------------------------------------------------------------------------------------

Intercept -0.5312 0.073 -7.238 0.000 -0.675 -0.387

C(timepoint)[T.june2021] 0.0571 0.012 4.723 0.000 0.033 0.081

C(timepoint)[T.may2020] 0.4437 0.009 47.777 0.000 0.425 0.462

C(Age_decade)[T.20.0] -0.1739 0.029 -6.009 0.000 -0.231 -0.117

C(Age_decade)[T.30.0] -0.0247 0.032 -0.783 0.434 -0.087 0.037

C(Age_decade)[T.40.0] 0.0310 0.031 0.995 0.320 -0.030 0.092

C(Age_decade)[T.50.0] 0.0922 0.031 2.988 0.003 0.032 0.153

C(Age_decade)[T.60.0] -0.0438 0.032 -1.373 0.170 -0.106 0.019

C(Age_decade)[T.70.0] -0.1794 0.034 -5.262 0.000 -0.246 -0.113

C(Age_decade)[T.80.0] -0.4779 0.046 -10.427 0.000 -0.568 -0.388

C(Residence)[T.United Kingdom] 0.0497 0.013 3.704 0.000 0.023 0.076

C(Sex)[T.Male] -0.3427 0.008 -41.811 0.000 -0.359 -0.327

C(Sex)[T.Other] -0.2521 0.056 -4.535 0.000 -0.361 -0.143

C(Education)[T.PhD] -0.1272 0.018 -7.233 0.000 -0.162 -0.093

C(Education)[T.School] 0.0164 0.008 2.049 0.040 0.001 0.032

C(Education)[T.preGCSE] 0.0341 0.030 1.119 0.263 -0.026 0.094

C(Ethnicity)[T.Asian or Asian British] -0.2229 0.058 -3.818 0.000 -0.337 -0.108

C(Ethnicity)[T.Black, Black British, Caribbean or African] -0.1989 0.085 -2.329 0.020 -0.366 -0.032

C(Ethnicity)[T.Mixed or multiple ethnic groups] -0.2690 0.060 -4.468 0.000 -0.387 -0.151

C(Ethnicity)[T.Other ethnic group] -0.0702 0.080 -0.877 0.380 -0.227 0.087

C(Ethnicity)[T.White] -0.1677 0.054 -3.108 0.002 -0.273 -0.062

C(Occupation)[T.Homemaker] 0.3317 0.038 8.738 0.000 0.257 0.406

C(Occupation)[T.Retired] 0.3154 0.033 9.514 0.000 0.250 0.380

C(Occupation)[T.Student] 0.1271 0.037 3.432 0.001 0.055 0.200

C(Occupation)[T.Unemployed/Looking for work] 0.2609 0.038 6.909 0.000 0.187 0.335

C(Occupation)[T.Worker] 0.3771 0.032 11.891 0.000 0.315 0.439

C(Exercise)[T.Daily] 0.3303 0.014 23.331 0.000 0.303 0.358

C(Exercise)[T.Hourly] 0.2763 0.060 4.595 0.000 0.158 0.394

C(Exercise)[T.More Often] 0.5964 0.053 11.354 0.000 0.493 0.699

C(Exercise)[T.Never] 0.1032 0.031 3.328 0.001 0.042 0.164

C(Exercise)[T.Once or twice a week] 0.1943 0.015 12.643 0.000 0.164 0.224

C(Exercise)[T.Several times a week] 0.2701 0.014 18.770 0.000 0.242 0.298

C(Meditation)[T.Daily] 0.2476 0.017 14.377 0.000 0.214 0.281

C(Meditation)[T.Hourly] -0.0473 0.193 -0.245 0.806 -0.425 0.331

C(Meditation)[T.More Often] -0.2833 0.129 -2.200 0.028 -0.536 -0.031

C(Meditation)[T.Never] -0.1272 0.008 -15.079 0.000 -0.144 -0.111

C(Meditation)[T.Once or twice a week] 0.1322 0.013 10.420 0.000 0.107 0.157

C(Meditation)[T.Several times a week] 0.1732 0.018 9.784 0.000 0.138 0.208

C(Reading)[T.Daily] -0.0098 0.010 -0.958 0.338 -0.030 0.010

C(Reading)[T.Hourly] 0.1881 0.042 4.458 0.000 0.105 0.271

C(Reading)[T.More Often] 0.1183 0.068 1.752 0.080 -0.014 0.251

C(Reading)[T.Never] -0.0920 0.018 -5.189 0.000 -0.127 -0.057

C(Reading)[T.Once or twice a week] 0.0215 0.011 1.916 0.055 -0.000 0.043

C(Reading)[T.Several times a week] -0.0098 0.012 -0.829 0.407 -0.033 0.013

C(OnlineGambling)[T.Daily] 0.1424 0.047 3.015 0.003 0.050 0.235

C(OnlineGambling)[T.Hourly] -0.4681 0.190 -2.458 0.014 -0.841 -0.095

C(OnlineGambling)[T.More Often] 0.0395 0.185 0.213 0.831 -0.324 0.403

C(OnlineGambling)[T.Never] -0.0282 0.011 -2.452 0.014 -0.051 -0.006

C(OnlineGambling)[T.Once or twice a week] 0.0331 0.020 1.637 0.102 -0.007 0.073

C(OnlineGambling)[T.Several times a week] 0.0583 0.040 1.462 0.144 -0.020 0.137

C(HoursOnline)[T.2-4h] 0.1492 0.017 8.645 0.000 0.115 0.183

C(HoursOnline)[T.4-6h] 0.1504 0.017 8.648 0.000 0.116 0.184

C(HoursOnline)[T.6-8h] 0.0685 0.019 3.624 0.000 0.031 0.106

C(HoursOnline)[T.8-10h] 0.0940 0.020 4.781 0.000 0.055 0.133

C(HoursOnline)[T.less than 2] 0.0948 0.021 4.571 0.000 0.054 0.135

C(HoursOnline)[T.more than 12h] 0.0177 0.030 0.581 0.561 -0.042 0.077

cigarettes_z -0.0140 0.004 -3.790 0.000 -0.021 -0.007

alcohol_z -0.0067 0.004 -1.791 0.073 -0.014 0.001

extraversion 0.0467 0.004 12.402 0.000 0.039 0.054

openness 0.1024 0.004 25.656 0.000 0.095 0.110

neuroticism -0.0620 0.004 -13.778 0.000 -0.071 -0.053

conscienciousness 0.0175 0.005 3.646 0.000 0.008 0.027

agreeableness 0.1642 0.005 36.084 0.000 0.155 0.173

perfectionism 0.0676 0.007 9.398 0.000 0.054 0.082

reward_drive 0.0862 0.005 17.379 0.000 0.076 0.096

==============================================================================

Omnibus: 6859.437 Durbin-Watson: 0.934

Prob(Omnibus): 0.000 Jarque-Bera (JB): 8968.910

Skew: -0.591 Prob(JB): 0.00

Kurtosis: 3.772 Cond. No. 135.

==============================================================================

Notes:

[1] Standard Errors assume that the covariance matrix of the errors is correctly specified.

OLS Regression Results

================================================================================

Dep. Variable: improved_environment R-squared: 0.076

Model: OLS Adj. R-squared: 0.075

Method: Least Squares F-statistic: 138.3

Date: Tue, 11 Apr 2023 Prob (F-statistic): 0.00

Time: 20:05:06 Log-Likelihood: -1.4373e+05

No. Observations: 108031 AIC: 2.876e+05

Df Residuals: 107966 BIC: 2.882e+05

Df Model: 64

Covariance Type: nonrobust

==============================================================================================================================

coef std err t P>|t| [0.025 0.975]

------------------------------------------------------------------------------------------------------------------------------

Intercept -0.1710 0.058 -2.937 0.003 -0.285 -0.057

C(timepoint)[T.june2021] 0.0087 0.010 0.906 0.365 -0.010 0.027

C(timepoint)[T.may2020] 0.2522 0.007 34.236 0.000 0.238 0.267

C(Age_decade)[T.20.0] -0.1082 0.023 -4.714 0.000 -0.153 -0.063

C(Age_decade)[T.30.0] -0.0780 0.025 -3.113 0.002 -0.127 -0.029

C(Age_decade)[T.40.0] -0.0856 0.025 -3.464 0.001 -0.134 -0.037

C(Age_decade)[T.50.0] -0.0284 0.024 -1.162 0.245 -0.076 0.020

C(Age_decade)[T.60.0] -0.0701 0.025 -2.770 0.006 -0.120 -0.020

C(Age_decade)[T.70.0] -0.0174 0.027 -0.642 0.521 -0.070 0.036

C(Age_decade)[T.80.0] 0.0291 0.036 0.801 0.423 -0.042 0.100

C(Residence)[T.United Kingdom] -0.0080 0.011 -0.751 0.453 -0.029 0.013

C(Sex)[T.Male] -0.1950 0.007 -29.985 0.000 -0.208 -0.182

C(Sex)[T.Other] 0.0573 0.044 1.299 0.194 -0.029 0.144

C(Education)[T.PhD] -0.1289 0.014 -9.237 0.000 -0.156 -0.102

C(Education)[T.School] 0.0606 0.006 9.570 0.000 0.048 0.073

C(Education)[T.preGCSE] 0.1856 0.024 7.681 0.000 0.138 0.233

C(Ethnicity)[T.Asian or Asian British] 0.0575 0.046 1.242 0.214 -0.033 0.148

C(Ethnicity)[T.Black, Black British, Caribbean or African] 0.0321 0.068 0.474 0.636 -0.101 0.165

C(Ethnicity)[T.Mixed or multiple ethnic groups] -0.1226 0.048 -2.568 0.010 -0.216 -0.029

C(Ethnicity)[T.Other ethnic group] 0.2058 0.063 3.241 0.001 0.081 0.330

C(Ethnicity)[T.White] 0.0434 0.043 1.015 0.310 -0.040 0.127

C(Occupation)[T.Homemaker] 0.1108 0.030 3.680 0.000 0.052 0.170

C(Occupation)[T.Retired] 0.2045 0.026 7.778 0.000 0.153 0.256

C(Occupation)[T.Student] 0.2495 0.029 8.490 0.000 0.192 0.307

C(Occupation)[T.Unemployed/Looking for work] 0.2281 0.030 7.618 0.000 0.169 0.287

C(Occupation)[T.Worker] 0.2248 0.025 8.937 0.000 0.175 0.274

C(Exercise)[T.Daily] 0.0412 0.011 3.670 0.000 0.019 0.063

C(Exercise)[T.Hourly] -0.0936 0.048 -1.963 0.050 -0.187 -0.000

C(Exercise)[T.More Often] 0.0053 0.042 0.128 0.898 -0.076 0.087

C(Exercise)[T.Never] 0.0584 0.025 2.375 0.018 0.010 0.107

C(Exercise)[T.Once or twice a week] 0.0279 0.012 2.286 0.022 0.004 0.052

C(Exercise)[T.Several times a week] 0.0129 0.011 1.126 0.260 -0.010 0.035

C(Meditation)[T.Daily] 0.1327 0.014 9.715 0.000 0.106 0.159

C(Meditation)[T.Hourly] 0.0801 0.153 0.524 0.601 -0.220 0.380

C(Meditation)[T.More Often] -0.1373 0.102 -1.344 0.179 -0.337 0.063

C(Meditation)[T.Never] -0.0768 0.007 -11.472 0.000 -0.090 -0.064

C(Meditation)[T.Once or twice a week] 0.0404 0.010 4.014 0.000 0.021 0.060

C(Meditation)[T.Several times a week] 0.0559 0.014 3.984 0.000 0.028 0.083

C(Reading)[T.Daily] -0.0160 0.008 -1.967 0.049 -0.032 -5.82e-05

C(Reading)[T.Hourly] 0.1558 0.033 4.654 0.000 0.090 0.221

C(Reading)[T.More Often] 0.2820 0.054 5.266 0.000 0.177 0.387

C(Reading)[T.Never] -0.0464 0.014 -3.300 0.001 -0.074 -0.019

C(Reading)[T.Once or twice a week] 0.0217 0.009 2.434 0.015 0.004 0.039

C(Reading)[T.Several times a week] -0.0332 0.009 -3.546 0.000 -0.052 -0.015

C(OnlineGambling)[T.Daily] -0.1024 0.037 -2.733 0.006 -0.176 -0.029

C(OnlineGambling)[T.Hourly] 0.1545 0.151 1.023 0.306 -0.142 0.451

C(OnlineGambling)[T.More Often] -0.9749 0.147 -6.631 0.000 -1.263 -0.687

C(OnlineGambling)[T.Never] -0.0698 0.009 -7.668 0.000 -0.088 -0.052

C(OnlineGambling)[T.Once or twice a week] -0.0468 0.016 -2.918 0.004 -0.078 -0.015

C(OnlineGambling)[T.Several times a week] -0.0824 0.032 -2.605 0.009 -0.144 -0.020

C(HoursOnline)[T.2-4h] 0.0165 0.014 1.205 0.228 -0.010 0.043

C(HoursOnline)[T.4-6h] 0.0356 0.014 2.582 0.010 0.009 0.063

C(HoursOnline)[T.6-8h] 0.0250 0.015 1.664 0.096 -0.004 0.054

C(HoursOnline)[T.8-10h] -0.0006 0.016 -0.039 0.969 -0.031 0.030

C(HoursOnline)[T.less than 2] 0.0759 0.016 4.616 0.000 0.044 0.108

C(HoursOnline)[T.more than 12h] 0.0930 0.024 3.847 0.000 0.046 0.140

cigarettes_z -0.0084 0.003 -2.890 0.004 -0.014 -0.003

alcohol_z -0.0313 0.003 -10.516 0.000 -0.037 -0.025

extraversion 0.0199 0.003 6.652 0.000 0.014 0.026

openness 0.0386 0.003 12.206 0.000 0.032 0.045

neuroticism -0.1441 0.004 -40.399 0.000 -0.151 -0.137

conscienciousness 0.0242 0.004 6.356 0.000 0.017 0.032

agreeableness 0.1196 0.004 33.143 0.000 0.113 0.127

perfectionism 0.0575 0.006 10.077 0.000 0.046 0.069

reward_drive 0.0374 0.004 9.517 0.000 0.030 0.045

==============================================================================

Omnibus: 1748.065 Durbin-Watson: 0.970

Prob(Omnibus): 0.000 Jarque-Bera (JB): 1728.108

Skew: -0.286 Prob(JB): 0.00

Kurtosis: 2.761 Cond. No. 135.

==============================================================================

Notes:

[1] Standard Errors assume that the covariance matrix of the errors is correctly specified.

OLS Regression Results

==============================================================================================

Dep. Variable: spending_more_time_with_loved_ones R-squared: 0.075

Model: OLS Adj. R-squared: 0.074

Method: Least Squares F-statistic: 136.2

Date: Tue, 11 Apr 2023 Prob (F-statistic): 0.00

Time: 20:05:15 Log-Likelihood: -2.0068e+05

No. Observations: 108031 AIC: 4.015e+05

Df Residuals: 107966 BIC: 4.021e+05

Df Model: 64

Covariance Type: nonrobust

==============================================================================================================================

coef std err t P>|t| [0.025 0.975]

------------------------------------------------------------------------------------------------------------------------------

Intercept 0.2506 0.099 2.541 0.011 0.057 0.444

C(timepoint)[T.june2021] -0.1343 0.016 -8.263 0.000 -0.166 -0.102

C(timepoint)[T.may2020] 0.2002 0.012 16.042 0.000 0.176 0.225

C(Age_decade)[T.20.0] -0.2344 0.039 -6.028 0.000 -0.311 -0.158

C(Age_decade)[T.30.0] -0.1967 0.042 -4.637 0.000 -0.280 -0.114

C(Age_decade)[T.40.0] -0.2644 0.042 -6.317 0.000 -0.346 -0.182

C(Age_decade)[T.50.0] -0.2958 0.041 -7.134 0.000 -0.377 -0.215

C(Age_decade)[T.60.0] -0.3759 0.043 -8.770 0.000 -0.460 -0.292

C(Age_decade)[T.70.0] -0.1820 0.046 -3.972 0.000 -0.272 -0.092

C(Age_decade)[T.80.0] -0.5504 0.062 -8.936 0.000 -0.671 -0.430

C(Residence)[T.United Kingdom] -0.0897 0.018 -4.974 0.000 -0.125 -0.054

C(Sex)[T.Male] -0.3307 0.011 -30.023 0.000 -0.352 -0.309

C(Sex)[T.Other] -0.5575 0.075 -7.463 0.000 -0.704 -0.411

C(Education)[T.PhD] -0.0879 0.024 -3.717 0.000 -0.134 -0.042

C(Education)[T.School] -0.0423 0.011 -3.939 0.000 -0.063 -0.021

C(Education)[T.preGCSE] -0.1164 0.041 -2.842 0.004 -0.197 -0.036

C(Ethnicity)[T.Asian or Asian British] 0.2922 0.078 3.725 0.000 0.138 0.446

C(Ethnicity)[T.Black, Black British, Caribbean or African] 0.4713 0.115 4.107 0.000 0.246 0.696

C(Ethnicity)[T.Mixed or multiple ethnic groups] 0.0907 0.081 1.122 0.262 -0.068 0.249

C(Ethnicity)[T.Other ethnic group] 0.4024 0.108 3.741 0.000 0.192 0.613

C(Ethnicity)[T.White] 0.0998 0.073 1.377 0.168 -0.042 0.242

C(Occupation)[T.Homemaker] 0.1871 0.051 3.668 0.000 0.087 0.287

C(Occupation)[T.Retired] 0.4508 0.045 10.119 0.000 0.363 0.538

C(Occupation)[T.Student] 0.3822 0.050 7.678 0.000 0.285 0.480

C(Occupation)[T.Unemployed/Looking for work] 0.2017 0.051 3.975 0.000 0.102 0.301

C(Occupation)[T.Worker] 0.3737 0.043 8.771 0.000 0.290 0.457

C(Exercise)[T.Daily] 0.1578 0.019 8.295 0.000 0.121 0.195

C(Exercise)[T.Hourly] 0.3748 0.081 4.638 0.000 0.216 0.533

C(Exercise)[T.More Often] 0.5013 0.071 7.103 0.000 0.363 0.640

C(Exercise)[T.Never] 0.1479 0.042 3.549 0.000 0.066 0.230

C(Exercise)[T.Once or twice a week] 0.1210 0.021 5.857 0.000 0.080 0.161

C(Exercise)[T.Several times a week] 0.1757 0.019 9.085 0.000 0.138 0.214

C(Meditation)[T.Daily] 0.2055 0.023 8.879 0.000 0.160 0.251

C(Meditation)[T.Hourly] 0.4218 0.259 1.628 0.104 -0.086 0.930

C(Meditation)[T.More Often] -1.5661 0.173 -9.050 0.000 -1.905 -1.227

C(Meditation)[T.Never] -0.0221 0.011 -1.952 0.051 -0.044 8.72e-05

C(Meditation)[T.Once or twice a week] 0.0435 0.017 2.550 0.011 0.010 0.077

C(Meditation)[T.Several times a week] 0.0185 0.024 0.777 0.437 -0.028 0.065

C(Reading)[T.Daily] -0.0902 0.014 -6.543 0.000 -0.117 -0.063

C(Reading)[T.Hourly] 0.2828 0.057 4.986 0.000 0.172 0.394

C(Reading)[T.More Often] 0.2252 0.091 2.482 0.013 0.047 0.403

C(Reading)[T.Never] -0.0607 0.024 -2.549 0.011 -0.107 -0.014

C(Reading)[T.Once or twice a week] 0.0177 0.015 1.176 0.240 -0.012 0.047

C(Reading)[T.Several times a week] -0.1143 0.016 -7.207 0.000 -0.145 -0.083

C(OnlineGambling)[T.Daily] -0.1464 0.063 -2.307 0.021 -0.271 -0.022

C(OnlineGambling)[T.Hourly] 0.2969 0.256 1.160 0.246 -0.205 0.798

C(OnlineGambling)[T.More Often] -0.5608 0.249 -2.252 0.024 -1.049 -0.073

C(OnlineGambling)[T.Never] -0.0892 0.015 -5.780 0.000 -0.119 -0.059

C(OnlineGambling)[T.Once or twice a week] 0.1032 0.027 3.801 0.000 0.050 0.156

C(OnlineGambling)[T.Several times a week] -0.2451 0.054 -4.573 0.000 -0.350 -0.140

C(HoursOnline)[T.2-4h] -0.3742 0.023 -16.132 0.000 -0.420 -0.329

C(HoursOnline)[T.4-6h] -0.2539 0.023 -10.863 0.000 -0.300 -0.208

C(HoursOnline)[T.6-8h] -0.1788 0.025 -7.038 0.000 -0.229 -0.129

C(HoursOnline)[T.8-10h] -0.1872 0.026 -7.083 0.000 -0.239 -0.135

C(HoursOnline)[T.less than 2] -0.6000 0.028 -21.540 0.000 -0.655 -0.545

C(HoursOnline)[T.more than 12h] -0.1289 0.041 -3.149 0.002 -0.209 -0.049

cigarettes_z -0.0588 0.005 -11.877 0.000 -0.068 -0.049

alcohol_z 0.0258 0.005 5.107 0.000 0.016 0.036

extraversion 0.1206 0.005 23.841 0.000 0.111 0.131

openness 0.0437 0.005 8.148 0.000 0.033 0.054

neuroticism -0.1546 0.006 -25.588 0.000 -0.166 -0.143

conscienciousness 0.0930 0.006 14.434 0.000 0.080 0.106

agreeableness 0.2773 0.006 45.346 0.000 0.265 0.289

perfectionism 0.0908 0.010 9.394 0.000 0.072 0.110

reward_drive 0.0808 0.007 12.116 0.000 0.068 0.094

==============================================================================

Omnibus: 7309.506 Durbin-Watson: 0.968

Prob(Omnibus): 0.000 Jarque-Bera (JB): 9281.928

Skew: -0.636 Prob(JB): 0.00

Kurtosis: 3.666 Cond. No. 135.

==============================================================================

Notes:

[1] Standard Errors assume that the covariance matrix of the errors is correctly specified.

Part VI – Modelling the contribution of various clusters to mental health variables and resilience

- 1. Modelling the contribution to mental health

OLS Regression Results

===============================================================================

Dep. Variable: anxiety_score_adj_z R-squared: 0.001

Model: OLS Adj. R-squared: 0.001

Method: Least Squares F-statistic: 3.771

Date: Tue, 11 Apr 2023 Prob (F-statistic): 9.19e-05

Time: 20:02:45 Log-Likelihood: -53027.

No. Observations: 37383 AIC: 1.061e+05

Df Residuals: 37373 BIC: 1.062e+05

Df Model: 9

Covariance Type: nonrobust

==============================================================================

coef std err t P>|t| [0.025 0.975]

------------------------------------------------------------------------------

Intercept 0.0005 0.007 0.067 0.947 -0.013 0.014

Cluster_1 0.0084 0.015 0.568 0.570 -0.021 0.037

Cluster_3 -0.0301 0.014 -2.217 0.027 -0.057 -0.003

Cluster_4 0.0455 0.027 1.674 0.094 -0.008 0.099

Cluster_5 0.1249 0.058 2.145 0.032 0.011 0.239

Cluster_6 0.0978 0.066 1.482 0.138 -0.032 0.227

Cluster_7 0.0302 0.059 0.508 0.611 -0.086 0.147

Cluster_8 0.1796 0.071 2.535 0.011 0.041 0.318

Cluster_9 0.2610 0.174 1.498 0.134 -0.080 0.602

Cluster_10 -0.3020 0.106 -2.844 0.004 -0.510 -0.094

==============================================================================

Omnibus: 2277.321 Durbin-Watson: 2.017

Prob(Omnibus): 0.000 Jarque-Bera (JB): 3104.291

Skew: 0.555 Prob(JB): 0.00

Kurtosis: 3.872 Cond. No. 34.9

==============================================================================

Notes:

[1] Standard Errors assume that the covariance matrix of the errors is correctly specified.

OLS Regression Results

===============================================================================================

Dep. Variable: unable_to_stop_worrying_score_adj_z R-squared: 0.001

Model: OLS Adj. R-squared: 0.000

Method: Least Squares F-statistic: 2.141

Date: Tue, 11 Apr 2023 Prob (F-statistic): 0.0230

Time: 20:02:45 Log-Likelihood: -53042.

No. Observations: 37388 AIC: 1.061e+05

Df Residuals: 37378 BIC: 1.062e+05

Df Model: 9

Covariance Type: nonrobust

==============================================================================

coef std err t P>|t| [0.025 0.975]

------------------------------------------------------------------------------

Intercept 0.0039 0.007 0.573 0.567 -0.009 0.017

Cluster_1 -0.0068 0.015 -0.460 0.646 -0.036 0.022

Cluster_3 -0.0244 0.014 -1.799 0.072 -0.051 0.002

Cluster_4 0.0103 0.027 0.379 0.705 -0.043 0.064

Cluster_5 0.0429 0.058 0.737 0.461 -0.071 0.157

Cluster_6 0.1408 0.066 2.133 0.033 0.011 0.270

Cluster_7 0.0479 0.059 0.807 0.420 -0.068 0.164

Cluster_8 0.1170 0.071 1.651 0.099 -0.022 0.256

Cluster_9 -0.0926 0.174 -0.531 0.595 -0.434 0.249

Cluster_10 -0.2651 0.106 -2.496 0.013 -0.473 -0.057

==============================================================================

Omnibus: 3452.309 Durbin-Watson: 2.014

Prob(Omnibus): 0.000 Jarque-Bera (JB): 5432.677

Skew: 0.695 Prob(JB): 0.00

Kurtosis: 4.248 Cond. No. 34.9

==============================================================================

Notes:

[1] Standard Errors assume that the covariance matrix of the errors is correctly specified.

OLS Regression Results

==================================================================================================

Dep. Variable: worrying_about_many_things_score_adj_z R-squared: 0.000

Model: OLS Adj. R-squared: 0.000

Method: Least Squares F-statistic: 1.678

Date: Tue, 11 Apr 2023 Prob (F-statistic): 0.0883

Time: 20:02:45 Log-Likelihood: -53045.

No. Observations: 37389 AIC: 1.061e+05

Df Residuals: 37379 BIC: 1.062e+05

Df Model: 9

Covariance Type: nonrobust

==============================================================================

coef std err t P>|t| [0.025 0.975]

------------------------------------------------------------------------------

Intercept 0.0046 0.007 0.678 0.498 -0.009 0.018

Cluster_1 -0.0146 0.015 -0.985 0.325 -0.044 0.014

Cluster_3 -0.0184 0.014 -1.358 0.174 -0.045 0.008

Cluster_4 -0.0181 0.027 -0.667 0.505 -0.071 0.035

Cluster_5 0.0226 0.058 0.388 0.698 -0.092 0.137

Cluster_6 0.1345 0.066 2.038 0.042 0.005 0.264

Cluster_7 0.0340 0.059 0.571 0.568 -0.082 0.150

Cluster_8 0.1627 0.071 2.296 0.022 0.024 0.302

Cluster_9 0.0956 0.174 0.549 0.583 -0.246 0.437

Cluster_10 -0.1265 0.106 -1.191 0.234 -0.335 0.082

==============================================================================

Omnibus: 2731.535 Durbin-Watson: 2.019

Prob(Omnibus): 0.000 Jarque-Bera (JB): 4089.455

Skew: 0.597 Prob(JB): 0.00

Kurtosis: 4.095 Cond. No. 34.9

==============================================================================

Notes:

[1] Standard Errors assume that the covariance matrix of the errors is correctly specified.

OLS Regression Results

=======================================================================================

Dep. Variable: unable_to_relax_score_adj_z R-squared: 0.001

Model: OLS Adj. R-squared: 0.000

Method: Least Squares F-statistic: 2.563

Date: Tue, 11 Apr 2023 Prob (F-statistic): 0.00606

Time: 20:02:45 Log-Likelihood: -53034.

No. Observations: 37384 AIC: 1.061e+05

Df Residuals: 37374 BIC: 1.062e+05

Df Model: 9

Covariance Type: nonrobust

==============================================================================

coef std err t P>|t| [0.025 0.975]

------------------------------------------------------------------------------

Intercept 0.0053 0.007 0.787 0.431 -0.008 0.019

Cluster_1 0.0106 0.015 0.716 0.474 -0.018 0.040

Cluster_3 -0.0413 0.014 -3.042 0.002 -0.068 -0.015

Cluster_4 0.0022 0.027 0.082 0.934 -0.051 0.055

Cluster_5 0.0194 0.058 0.333 0.739 -0.095 0.133

Cluster_6 0.1168 0.066 1.769 0.077 -0.013 0.246

Cluster_7 0.0205 0.059 0.346 0.730 -0.096 0.137

Cluster_8 0.0890 0.071 1.256 0.209 -0.050 0.228

Cluster_9 0.0424 0.174 0.244 0.808 -0.299 0.384

Cluster_10 -0.2616 0.106 -2.463 0.014 -0.470 -0.053

==============================================================================

Omnibus: 2458.859 Durbin-Watson: 2.002

Prob(Omnibus): 0.000 Jarque-Bera (JB): 3250.971

Skew: 0.601 Prob(JB): 0.00

Kurtosis: 3.802 Cond. No. 34.9

==============================================================================

Notes:

[1] Standard Errors assume that the covariance matrix of the errors is correctly specified.

OLS Regression Results

====================================================================================

Dep. Variable: restlessness_score_adj_z R-squared: 0.001

Model: OLS Adj. R-squared: 0.001

Method: Least Squares F-statistic: 3.423

Date: Tue, 11 Apr 2023 Prob (F-statistic): 0.000321

Time: 20:02:45 Log-Likelihood: -53037.

No. Observations: 37389 AIC: 1.061e+05

Df Residuals: 37379 BIC: 1.062e+05

Df Model: 9

Covariance Type: nonrobust

==============================================================================

coef std err t P>|t| [0.025 0.975]

------------------------------------------------------------------------------

Intercept 0.0009 0.007 0.132 0.895 -0.012 0.014

Cluster_1 0.0077 0.015 0.522 0.601 -0.021 0.037

Cluster_3 -0.0280 0.014 -2.061 0.039 -0.055 -0.001

Cluster_4 0.0218 0.027 0.802 0.423 -0.031 0.075

Cluster_5 0.2376 0.058 4.082 0.000 0.124 0.352

Cluster_6 0.0086 0.066 0.130 0.896 -0.121 0.138

Cluster_7 0.0535 0.059 0.901 0.368 -0.063 0.170

Cluster_8 0.0283 0.071 0.399 0.690 -0.111 0.167

Cluster_9 0.3611 0.174 2.074 0.038 0.020 0.703

Cluster_10 -0.1479 0.106 -1.393 0.164 -0.356 0.060

==============================================================================

Omnibus: 5672.095 Durbin-Watson: 1.999

Prob(Omnibus): 0.000 Jarque-Bera (JB): 9620.314

Skew: 1.010 Prob(JB): 0.00

Kurtosis: 4.446 Cond. No. 34.9

==============================================================================

Notes:

[1] Standard Errors assume that the covariance matrix of the errors is correctly specified.

OLS Regression Results

====================================================================================

Dep. Variable: irritability_score_adj_z R-squared: 0.002

Model: OLS Adj. R-squared: 0.002

Method: Least Squares F-statistic: 7.255

Date: Tue, 11 Apr 2023 Prob (F-statistic): 1.29e-10

Time: 20:02:45 Log-Likelihood: -53020.

No. Observations: 37389 AIC: 1.061e+05

Df Residuals: 37379 BIC: 1.061e+05

Df Model: 9

Covariance Type: nonrobust

==============================================================================

coef std err t P>|t| [0.025 0.975]

------------------------------------------------------------------------------

Intercept -0.0205 0.007 -3.023 0.003 -0.034 -0.007

Cluster_1 0.0863 0.015 5.843 0.000 0.057 0.115

Cluster_3 0.0109 0.014 0.805 0.421 -0.016 0.037

Cluster_4 0.0049 0.027 0.181 0.857 -0.048 0.058

Cluster_5 0.1393 0.058 2.394 0.017 0.025 0.253

Cluster_6 0.1385 0.066 2.099 0.036 0.009 0.268

Cluster_7 0.0924 0.059 1.557 0.119 -0.024 0.209

Cluster_8 0.3055 0.071 4.314 0.000 0.167 0.444

Cluster_9 0.3945 0.174 2.266 0.023 0.053 0.736

Cluster_10 0.0484 0.106 0.456 0.649 -0.160 0.256

==============================================================================

Omnibus: 2473.005 Durbin-Watson: 2.000

Prob(Omnibus): 0.000 Jarque-Bera (JB): 3402.394

Skew: 0.586 Prob(JB): 0.00

Kurtosis: 3.901 Cond. No. 34.9

==============================================================================

Notes:

[1] Standard Errors assume that the covariance matrix of the errors is correctly specified.

OLS Regression Results

============================================================================================

Dep. Variable: negative_premonition_score_adj_z R-squared: 0.000

Model: OLS Adj. R-squared: 0.000

Method: Least Squares F-statistic: 1.932

Date: Tue, 11 Apr 2023 Prob (F-statistic): 0.0430

Time: 20:02:45 Log-Likelihood: -53044.

No. Observations: 37389 AIC: 1.061e+05

Df Residuals: 37379 BIC: 1.062e+05

Df Model: 9

Covariance Type: nonrobust

==============================================================================

coef std err t P>|t| [0.025 0.975]

------------------------------------------------------------------------------

Intercept -0.0022 0.007 -0.323 0.746 -0.015 0.011

Cluster_1 0.0107 0.015 0.722 0.470 -0.018 0.040

Cluster_3 0.0008 0.014 0.060 0.952 -0.026 0.027

Cluster_4 -0.0174 0.027 -0.642 0.521 -0.071 0.036

Cluster_5 -0.0086 0.058 -0.147 0.883 -0.123 0.106

Cluster_6 0.1249 0.066 1.892 0.058 -0.004 0.254

Cluster_7 0.1187 0.059 1.999 0.046 0.002 0.235

Cluster_8 0.0206 0.071 0.291 0.771 -0.118 0.160

Cluster_9 0.0698 0.174 0.401 0.689 -0.272 0.411

Cluster_10 -0.3095 0.106 -2.915 0.004 -0.518 -0.101

==============================================================================

Omnibus: 4881.943 Durbin-Watson: 2.014

Prob(Omnibus): 0.000 Jarque-Bera (JB): 8436.810

Skew: 0.878 Prob(JB): 0.00

Kurtosis: 4.528 Cond. No. 34.9

==============================================================================

Notes:

[1] Standard Errors assume that the covariance matrix of the errors is correctly specified.

OLS Regression Results

==============================================================================

Dep. Variable: apathy_score_adj_z R-squared: 0.003

Model: OLS Adj. R-squared: 0.002

Method: Least Squares F-statistic: 11.09

Date: Tue, 11 Apr 2023 Prob (F-statistic): 1.81e-17

Time: 20:02:45 Log-Likelihood: -52990.

No. Observations: 37380 AIC: 1.060e+05

Df Residuals: 37370 BIC: 1.061e+05

Df Model: 9

Covariance Type: nonrobust

==============================================================================

coef std err t P>|t| [0.025 0.975]

------------------------------------------------------------------------------

Intercept -0.0149 0.007 -2.199 0.028 -0.028 -0.002

Cluster_1 0.0587 0.015 3.978 0.000 0.030 0.088

Cluster_3 -0.0242 0.014 -1.789 0.074 -0.051 0.002

Cluster_4 0.1276 0.027 4.698 0.000 0.074 0.181

Cluster_5 0.1816 0.058 3.123 0.002 0.068 0.296

Cluster_6 0.2385 0.066 3.617 0.000 0.109 0.368

Cluster_7 0.0726 0.059 1.223 0.221 -0.044 0.189

Cluster_8 0.2986 0.071 4.219 0.000 0.160 0.437

Cluster_9 0.7213 0.174 4.145 0.000 0.380 1.062

Cluster_10 -0.0744 0.106 -0.701 0.483 -0.282 0.134

==============================================================================

Omnibus: 5674.621 Durbin-Watson: 2.007

Prob(Omnibus): 0.000 Jarque-Bera (JB): 10712.687

Skew: 0.959 Prob(JB): 0.00

Kurtosis: 4.789 Cond. No. 34.9

==============================================================================

Notes:

[1] Standard Errors assume that the covariance matrix of the errors is correctly specified.

OLS Regression Results

==================================================================================

Dep. Variable: depression_score_adj_z R-squared: 0.002

Model: OLS Adj. R-squared: 0.002

Method: Least Squares F-statistic: 8.623

Date: Tue, 11 Apr 2023 Prob (F-statistic): 4.99e-13

Time: 20:02:46 Log-Likelihood: -53014.

No. Observations: 37389 AIC: 1.060e+05

Df Residuals: 37379 BIC: 1.061e+05

Df Model: 9

Covariance Type: nonrobust

==============================================================================

coef std err t P>|t| [0.025 0.975]

------------------------------------------------------------------------------

Intercept -0.0132 0.007 -1.943 0.052 -0.026 0.000

Cluster_1 0.0478 0.015 3.240 0.001 0.019 0.077

Cluster_3 -0.0199 0.014 -1.468 0.142 -0.046 0.007

Cluster_4 0.0978 0.027 3.602 0.000 0.045 0.151

Cluster_5 0.2609 0.058 4.484 0.000 0.147 0.375

Cluster_6 0.2303 0.066 3.492 0.000 0.101 0.360

Cluster_7 0.1558 0.059 2.625 0.009 0.039 0.272

Cluster_8 0.2023 0.071 2.857 0.004 0.064 0.341

Cluster_9 0.3750 0.174 2.155 0.031 0.034 0.716

Cluster_10 -0.1303 0.106 -1.228 0.220 -0.338 0.078

==============================================================================

Omnibus: 4822.922 Durbin-Watson: 2.010

Prob(Omnibus): 0.000 Jarque-Bera (JB): 8720.259

Skew: 0.850 Prob(JB): 0.00

Kurtosis: 4.645 Cond. No. 34.9

==============================================================================

Notes:

[1] Standard Errors assume that the covariance matrix of the errors is correctly specified.

OLS Regression Results

=================================================================================

Dep. Variable: tiredness_score_adj_z R-squared: 0.002

Model: OLS Adj. R-squared: 0.001

Method: Least Squares F-statistic: 6.598

Date: Tue, 11 Apr 2023 Prob (F-statistic): 1.79e-09

Time: 20:02:46 Log-Likelihood: -53023.

No. Observations: 37389 AIC: 1.061e+05

Df Residuals: 37379 BIC: 1.062e+05

Df Model: 9

Covariance Type: nonrobust

==============================================================================

coef std err t P>|t| [0.025 0.975]

------------------------------------------------------------------------------

Intercept -0.0111 0.007 -1.642 0.101 -0.024 0.002

Cluster_1 0.0796 0.015 5.391 0.000 0.051 0.109

Cluster_3 -0.0239 0.014 -1.764 0.078 -0.050 0.003

Cluster_4 0.0304 0.027 1.119 0.263 -0.023 0.084

Cluster_5 0.0373 0.058 0.641 0.522 -0.077 0.151

Cluster_6 0.1679 0.066 2.545 0.011 0.039 0.297

Cluster_7 0.1056 0.059 1.779 0.075 -0.011 0.222

Cluster_8 0.0294 0.071 0.415 0.678 -0.109 0.168

Cluster_9 0.4775 0.174 2.743 0.006 0.136 0.819

Cluster_10 -0.2077 0.106 -1.957 0.050 -0.416 0.000

==============================================================================

Omnibus: 1834.097 Durbin-Watson: 2.018

Prob(Omnibus): 0.000 Jarque-Bera (JB): 2172.661

Skew: 0.538 Prob(JB): 0.00

Kurtosis: 3.485 Cond. No. 34.9

==============================================================================

Notes:

[1] Standard Errors assume that the covariance matrix of the errors is correctly specified.

OLS Regression Results

==============================================================================================

Dep. Variable: concentration_problems_score_adj_z R-squared: 0.002

Model: OLS Adj. R-squared: 0.002

Method: Least Squares F-statistic: 8.356

Date: Tue, 11 Apr 2023 Prob (F-statistic): 1.49e-12

Time: 20:02:46 Log-Likelihood: -53015.

No. Observations: 37389 AIC: 1.061e+05

Df Residuals: 37379 BIC: 1.061e+05

Df Model: 9

Covariance Type: nonrobust

==============================================================================

coef std err t P>|t| [0.025 0.975]

------------------------------------------------------------------------------

Intercept -0.0218 0.007 -3.224 0.001 -0.035 -0.009

Cluster_1 0.0880 0.015 5.957 0.000 0.059 0.117

Cluster_3 0.0051 0.014 0.378 0.706 -0.021 0.032

Cluster_4 0.0454 0.027 1.672 0.094 -0.008 0.099

Cluster_5 0.1651 0.058 2.838 0.005 0.051 0.279

Cluster_6 0.1773 0.066 2.689 0.007 0.048 0.307

Cluster_7 0.1551 0.059 2.613 0.009 0.039 0.271

Cluster_8 0.1947 0.071 2.750 0.006 0.056 0.333

Cluster_9 0.6557 0.174 3.767 0.000 0.315 0.997

Cluster_10 0.0893 0.106 0.841 0.400 -0.119 0.297

==============================================================================

Omnibus: 3767.637 Durbin-Watson: 1.984

Prob(Omnibus): 0.000 Jarque-Bera (JB): 5550.504

Skew: 0.774 Prob(JB): 0.00

Kurtosis: 4.079 Cond. No. 34.9

==============================================================================

Notes:

[1] Standard Errors assume that the covariance matrix of the errors is correctly specified.

OLS Regression Results

================================================================================

Dep. Variable: insomnia_score_adj_z R-squared: 0.001

Model: OLS Adj. R-squared: 0.001

Method: Least Squares F-statistic: 4.824

Date: Tue, 11 Apr 2023 Prob (F-statistic): 1.82e-06

Time: 20:02:46 Log-Likelihood: -53030.

No. Observations: 37388 AIC: 1.061e+05

Df Residuals: 37378 BIC: 1.062e+05

Df Model: 9

Covariance Type: nonrobust

==============================================================================

coef std err t P>|t| [0.025 0.975]

------------------------------------------------------------------------------

Intercept -0.0050 0.007 -0.741 0.459 -0.018 0.008

Cluster_1 0.0570 0.015 3.857 0.000 0.028 0.086

Cluster_3 -0.0350 0.014 -2.582 0.010 -0.062 -0.008

Cluster_4 0.0523 0.027 1.924 0.054 -0.001 0.105

Cluster_5 0.0064 0.058 0.109 0.913 -0.108 0.120

Cluster_6 0.1369 0.066 2.076 0.038 0.008 0.266

Cluster_7 0.0358 0.059 0.603 0.547 -0.081 0.152

Cluster_8 -0.0261 0.071 -0.369 0.712 -0.165 0.113

Cluster_9 0.3869 0.174 2.222 0.026 0.046 0.728

Cluster_10 -0.1795 0.106 -1.691 0.091 -0.388 0.029

==============================================================================

Omnibus: 1506.992 Durbin-Watson: 2.001

Prob(Omnibus): 0.000 Jarque-Bera (JB): 1694.721

Skew: 0.511 Prob(JB): 0.00

Kurtosis: 3.206 Cond. No. 34.9

==============================================================================

Notes:

[1] Standard Errors assume that the covariance matrix of the errors is correctly specified.

OLS Regression Results

==============================================================================

Dep. Variable: MH_composite_adj_z R-squared: 0.001

Model: OLS Adj. R-squared: 0.001

Method: Least Squares F-statistic: 6.076

Date: Tue, 11 Apr 2023 Prob (F-statistic): 1.42e-08

Time: 20:02:46 Log-Likelihood: -52998.

No. Observations: 37370 AIC: 1.060e+05

Df Residuals: 37360 BIC: 1.061e+05

Df Model: 9

Covariance Type: nonrobust

==============================================================================

coef std err t P>|t| [0.025 0.975]

------------------------------------------------------------------------------

Intercept -0.0071 0.007 -1.045 0.296 -0.020 0.006

Cluster_1 0.0431 0.015 2.919 0.004 0.014 0.072

Cluster_3 -0.0282 0.014 -2.082 0.037 -0.055 -0.002

Cluster_4 0.0453 0.027 1.667 0.096 -0.008 0.099

Cluster_5 0.1417 0.058 2.435 0.015 0.028 0.256

Cluster_6 0.2078 0.066 3.150 0.002 0.079 0.337

Cluster_7 0.1068 0.059 1.799 0.072 -0.010 0.223

Cluster_8 0.2001 0.071 2.826 0.005 0.061 0.339

Cluster_9 0.3867 0.174 2.221 0.026 0.045 0.728

Cluster_10 -0.2302 0.106 -2.168 0.030 -0.438 -0.022

==============================================================================

Omnibus: 4472.912 Durbin-Watson: 2.011

Prob(Omnibus): 0.000 Jarque-Bera (JB): 8628.012

Skew: 0.775 Prob(JB): 0.00

Kurtosis: 4.771 Cond. No. 34.8

==============================================================================

Notes:

[1] Standard Errors assume that the covariance matrix of the errors is correctly specified.

2. Modelling the contribution of different clusters to resilience variables

OLS Regression Results

====================================================================================================

Dep. Variable: spending_more_time_with_loved_ones_adj_z R-squared: 0.001

Model: OLS Adj. R-squared: 0.001

Method: Least Squares F-statistic: 5.369

Date: Tue, 11 Apr 2023 Prob (F-statistic): 2.25e-07

Time: 17:53:18 Log-Likelihood: -54689.

No. Observations: 37303 AIC: 1.094e+05

Df Residuals: 37293 BIC: 1.095e+05

Df Model: 9

Covariance Type: nonrobust

==============================================================================

coef std err t P>|t| [0.025 0.975]

------------------------------------------------------------------------------

Intercept 0.0283 0.007 3.981 0.000 0.014 0.042

Cluster_1 -0.0692 0.016 -4.465 0.000 -0.100 -0.039

Cluster_3 -0.0594 0.014 -4.174 0.000 -0.087 -0.032

Cluster_4 -0.0799 0.029 -2.801 0.005 -0.136 -0.024

Cluster_5 -0.1580 0.061 -2.588 0.010 -0.278 -0.038

Cluster_6 -0.0175 0.069 -0.253 0.800 -0.153 0.118

Cluster_7 -0.0438 0.062 -0.703 0.482 -0.166 0.078

Cluster_8 -0.1792 0.074 -2.413 0.016 -0.325 -0.034

Cluster_9 -0.4248 0.183 -2.326 0.020 -0.783 -0.067

Cluster_10 0.0283 0.111 0.254 0.799 -0.190 0.247

==============================================================================

Omnibus: 2724.274 Durbin-Watson: 1.986

Prob(Omnibus): 0.000 Jarque-Bera (JB): 3495.294

Skew: -0.668 Prob(JB): 0.00

Kurtosis: 3.681 Cond. No. 34.8

==============================================================================

Notes:

[1] Standard Errors assume that the covariance matrix of the errors is correctly specified. OLS Regression Results

========================================================================================

Dep. Variable: more_relaxed_lifestyle_adj_z R-squared: 0.002

Model: OLS Adj. R-squared: 0.001

Method: Least Squares F-statistic: 7.037

Date: Tue, 11 Apr 2023 Prob (F-statistic): 3.11e-10

Time: 20:05:25 Log-Likelihood: -53344.

No. Observations: 37303 AIC: 1.067e+05

Df Residuals: 37293 BIC: 1.068e+05

Df Model: 9

Covariance Type: nonrobust

==============================================================================

coef std err t P>|t| [0.025 0.975]

------------------------------------------------------------------------------

Intercept -0.0141 0.007 -2.060 0.039 -0.028 -0.001

Cluster_1 0.0549 0.015 3.669 0.000 0.026 0.084

Cluster_3 -0.0194 0.014 -1.414 0.157 -0.046 0.008

Cluster_4 0.1101 0.028 4.003 0.000 0.056 0.164

Cluster_5 0.1187 0.059 2.016 0.044 0.003 0.234

Cluster_6 0.2401 0.067 3.597 0.000 0.109 0.371

Cluster_7 0.1551 0.060 2.581 0.010 0.037 0.273

Cluster_8 0.1903 0.072 2.655 0.008 0.050 0.331

Cluster_9 0.2273 0.176 1.290 0.197 -0.118 0.573

Cluster_10 0.1121 0.107 1.043 0.297 -0.098 0.323

==============================================================================

Omnibus: 341.277 Durbin-Watson: 1.996

Prob(Omnibus): 0.000 Jarque-Bera (JB): 258.928

Skew: -0.114 Prob(JB): 5.95e-57

Kurtosis: 2.662 Cond. No. 34.8

==============================================================================

Notes:

[1] Standard Errors assume that the covariance matrix of the errors is correctly specified.

OLS Regression Results

=====================================================================================

Dep. Variable: disrupted_lifestyle_adj_z R-squared: 0.001

Model: OLS Adj. R-squared: 0.000

Method: Least Squares F-statistic: 2.271

Date: Tue, 11 Apr 2023 Prob (F-statistic): 0.0154

Time: 20:05:25 Log-Likelihood: -54511.

No. Observations: 37303 AIC: 1.090e+05

Df Residuals: 37293 BIC: 1.091e+05

Df Model: 9

Covariance Type: nonrobust

==============================================================================

coef std err t P>|t| [0.025 0.975]

------------------------------------------------------------------------------

Intercept -0.0055 0.007 -0.772 0.440 -0.019 0.008

Cluster_1 0.0242 0.015 1.567 0.117 -0.006 0.054

Cluster_3 0.0116 0.014 0.816 0.415 -0.016 0.039

Cluster_4 -0.0431 0.028 -1.519 0.129 -0.099 0.013

Cluster_5 -0.1008 0.061 -1.659 0.097 -0.220 0.018

Cluster_6 0.2126 0.069 3.087 0.002 0.078 0.348

Cluster_7 0.0390 0.062 0.628 0.530 -0.083 0.160

Cluster_8 0.0161 0.074 0.218 0.828 -0.129 0.161

Cluster_9 0.2654 0.182 1.460 0.144 -0.091 0.622

Cluster_10 0.0030 0.111 0.027 0.979 -0.214 0.220

==============================================================================

Omnibus: 247.404 Durbin-Watson: 2.005

Prob(Omnibus): 0.000 Jarque-Bera (JB): 197.198

Skew: 0.102 Prob(JB): 1.51e-43

Kurtosis: 2.708 Cond. No. 34.8

==============================================================================

Notes:

[1] Standard Errors assume that the covariance matrix of the errors is correctly specified.

OLS Regression Results

==========================================================================================

Dep. Variable: elevated_health_concerns_adj_z R-squared: 0.001

Model: OLS Adj. R-squared: 0.001

Method: Least Squares F-statistic: 4.755

Date: Tue, 11 Apr 2023 Prob (F-statistic): 2.37e-06

Time: 20:05:25 Log-Likelihood: -53545.

No. Observations: 37303 AIC: 1.071e+05

Df Residuals: 37293 BIC: 1.072e+05

Df Model: 9

Covariance Type: nonrobust

==============================================================================

coef std err t P>|t| [0.025 0.975]

------------------------------------------------------------------------------

Intercept 0.0223 0.007 3.227 0.001 0.009 0.036

Cluster_1 -0.0648 0.015 -4.308 0.000 -0.094 -0.035

Cluster_3 -0.0306 0.014 -2.217 0.027 -0.058 -0.004

Cluster_4 -0.0851 0.028 -3.077 0.002 -0.139 -0.031

Cluster_5 -0.0729 0.059 -1.232 0.218 -0.189 0.043

Cluster_6 -0.2494 0.067 -3.716 0.000 -0.381 -0.118

Cluster_7 -0.0257 0.060 -0.426 0.670 -0.144 0.093

Cluster_8 -0.0620 0.072 -0.860 0.390 -0.203 0.079

Cluster_9 -0.3591 0.177 -2.028 0.043 -0.706 -0.012

Cluster_10 -0.0073 0.108 -0.068 0.946 -0.219 0.204

==============================================================================

Omnibus: 259.028 Durbin-Watson: 1.987

Prob(Omnibus): 0.000 Jarque-Bera (JB): 270.399

Skew: -0.188 Prob(JB): 1.92e-59

Kurtosis: 3.181 Cond. No. 34.8

==============================================================================

Notes:

[1] Standard Errors assume that the covariance matrix of the errors is correctly specified.

OLS Regression Results

==============================================================================

Dep. Variable: optimism_adj_z R-squared: 0.000

Model: OLS Adj. R-squared: 0.000

Method: Least Squares F-statistic: 1.848

Date: Tue, 11 Apr 2023 Prob (F-statistic): 0.0548

Time: 20:05:25 Log-Likelihood: -53075.

No. Observations: 37303 AIC: 1.062e+05

Df Residuals: 37293 BIC: 1.063e+05

Df Model: 9

Covariance Type: nonrobust

==============================================================================

coef std err t P>|t| [0.025 0.975]

------------------------------------------------------------------------------

Intercept 0.0051 0.007 0.743 0.457 -0.008 0.018

Cluster_1 -0.0170 0.015 -1.146 0.252 -0.046 0.012

Cluster_3 -0.0220 0.014 -1.610 0.107 -0.049 0.005

Cluster_4 0.0371 0.027 1.360 0.174 -0.016 0.091

Cluster_5 0.0206 0.058 0.352 0.725 -0.094 0.135

Cluster_6 -0.0943 0.066 -1.423 0.155 -0.224 0.036

Cluster_7 0.0252 0.060 0.422 0.673 -0.092 0.142

Cluster_8 0.0893 0.071 1.255 0.210 -0.050 0.229

Cluster_9 -0.3329 0.175 -1.903 0.057 -0.676 0.010

Cluster_10 0.1820 0.107 1.707 0.088 -0.027 0.391

==============================================================================

Omnibus: 436.836 Durbin-Watson: 1.998

Prob(Omnibus): 0.000 Jarque-Bera (JB): 458.545

Skew: -0.253 Prob(JB): 2.68e-100

Kurtosis: 3.196 Cond. No. 34.8

==============================================================================

Notes:

[1] Standard Errors assume that the covariance matrix of the errors is correctly specified.

OLS Regression Results

============================================================================================

Dep. Variable: increased_conflict_at_home_adj_z R-squared: 0.001

Model: OLS Adj. R-squared: 0.000

Method: Least Squares F-statistic: 2.112

Date: Tue, 11 Apr 2023 Prob (F-statistic): 0.0252

Time: 20:05:25 Log-Likelihood: -53354.

No. Observations: 37303 AIC: 1.067e+05

Df Residuals: 37293 BIC: 1.068e+05

Df Model: 9

Covariance Type: nonrobust

==============================================================================

coef std err t P>|t| [0.025 0.975]

------------------------------------------------------------------------------

Intercept 0.0175 0.007 2.555 0.011 0.004 0.031

Cluster_1 -0.0432 0.015 -2.887 0.004 -0.073 -0.014

Cluster_3 -0.0479 0.014 -3.483 0.000 -0.075 -0.021

Cluster_4 -0.0045 0.028 -0.162 0.871 -0.058 0.049

Cluster_5 -0.0416 0.059 -0.707 0.480 -0.157 0.074

Cluster_6 -0.0975 0.067 -1.460 0.144 -0.228 0.033

Cluster_7 -0.0273 0.060 -0.454 0.650 -0.145 0.090

Cluster_8 -0.0037 0.072 -0.052 0.958 -0.144 0.137

Cluster_9 -0.0931 0.176 -0.529 0.597 -0.439 0.252

Cluster_10 -0.0598 0.107 -0.557 0.578 -0.270 0.151

==============================================================================

Omnibus: 2836.820 Durbin-Watson: 2.010

Prob(Omnibus): 0.000 Jarque-Bera (JB): 4161.338

Skew: -0.623 Prob(JB): 0.00

Kurtosis: 4.060 Cond. No. 34.8

==============================================================================

Notes:

[1] Standard Errors assume that the covariance matrix of the errors is correctly specified.

OLS Regression Results

======================================================================================

Dep. Variable: improved_environment_adj_z R-squared: 0.003

Model: OLS Adj. R-squared: 0.003

Method: Least Squares F-statistic: 14.11

Date: Tue, 11 Apr 2023 Prob (F-statistic): 5.52e-23

Time: 20:05:25 Log-Likelihood: -54560.

No. Observations: 37303 AIC: 1.091e+05

Df Residuals: 37293 BIC: 1.092e+05

Df Model: 9

Covariance Type: nonrobust

==============================================================================

coef std err t P>|t| [0.025 0.975]

------------------------------------------------------------------------------

Intercept 0.0471 0.007 6.639 0.000 0.033 0.061

Cluster_1 -0.1066 0.015 -6.896 0.000 -0.137 -0.076

Cluster_3 -0.0959 0.014 -6.761 0.000 -0.124 -0.068

Cluster_4 -0.1611 0.028 -5.670 0.000 -0.217 -0.105

Cluster_5 -0.3134 0.061 -5.152 0.000 -0.433 -0.194

Cluster_6 -0.1496 0.069 -2.169 0.030 -0.285 -0.014

Cluster_7 -0.0514 0.062 -0.827 0.408 -0.173 0.070

Cluster_8 -0.1823 0.074 -2.463 0.014 -0.327 -0.037

Cluster_9 -0.3153 0.182 -1.732 0.083 -0.672 0.041

Cluster_10 -0.2662 0.111 -2.399 0.016 -0.484 -0.049

==============================================================================

Omnibus: 572.607 Durbin-Watson: 1.986

Prob(Omnibus): 0.000 Jarque-Bera (JB): 434.187

Skew: -0.175 Prob(JB): 5.22e-95

Kurtosis: 2.605 Cond. No. 34.8

==============================================================================

Notes:

[1] Standard Errors assume that the covariance matrix of the errors is correctly specified.

OLS Regression Results

====================================================================================================

Dep. Variable: spending_more_time_with_loved_ones_adj_z R-squared: 0.001

Model: OLS Adj. R-squared: 0.001

Method: Least Squares F-statistic: 5.369

Date: Tue, 11 Apr 2023 Prob (F-statistic): 2.25e-07

Time: 20:05:26 Log-Likelihood: -54689.

No. Observations: 37303 AIC: 1.094e+05

Df Residuals: 37293 BIC: 1.095e+05

Df Model: 9

Covariance Type: nonrobust

==============================================================================

coef std err t P>|t| [0.025 0.975]

------------------------------------------------------------------------------

Intercept 0.0283 0.007 3.981 0.000 0.014 0.042

Cluster_1 -0.0692 0.016 -4.465 0.000 -0.100 -0.039

Cluster_3 -0.0594 0.014 -4.174 0.000 -0.087 -0.032

Cluster_4 -0.0799 0.029 -2.801 0.005 -0.136 -0.024

Cluster_5 -0.1580 0.061 -2.588 0.010 -0.278 -0.038

Cluster_6 -0.0175 0.069 -0.253 0.800 -0.153 0.118

Cluster_7 -0.0438 0.062 -0.703 0.482 -0.166 0.078

Cluster_8 -0.1792 0.074 -2.413 0.016 -0.325 -0.034

Cluster_9 -0.4248 0.183 -2.326 0.020 -0.783 -0.067

Cluster_10 0.0283 0.111 0.254 0.799 -0.190 0.247

==============================================================================

Omnibus: 2724.274 Durbin-Watson: 1.986

Prob(Omnibus): 0.000 Jarque-Bera (JB): 3495.294

Skew: -0.668 Prob(JB): 0.00

Kurtosis: 3.681 Cond. No. 34.8

==============================================================================

Notes:

[1] Standard Errors assume that the covariance matrix of the errors is correctly specified.
